# [3 + 2] Cycloadditions of Tertiary Amine *N*‑Oxides and Azoarenes as a Route to Substituted 1,2,4-Triazolidines

**DOI:** 10.1021/acsorginorgau.5c00098

**Published:** 2025-12-05

**Authors:** Nicholas A. Frankos, Malavika S. Nair, Aiden M. Lane, Megan M. Glista, Joshua K. Graber, Abbigail E. F. Black, Trista G. L. X. Newman, Elias R. Griffin, Kiera M. Luca, Eric J. Chartier, David B. Heisler, Thomas D. Montgomery

**Affiliations:** Department of Chemistry and Biochemistry, 6613 Duquesne University, 600 Forbes Ave. Pittsburgh, Pennsylvania 15282, United States

**Keywords:** 1,2,4-triazolidine, azoarene, cycloaddition, tertiary amine *N*-oxide, antibacterial

## Abstract

We have developed a synthesis of 29 novel 1,2,4-triazolidines using tertiary amine *N*-oxides and a wide range of substituted azoarenes. Our method utilizes a base-mediated [3 + 2] cycloaddition, starting from either commercially available or easily accessible precursors to generate triazolidines in yields up to 99%. Density functional theory calculations were performed in parallel to the experimental work to provide insights into the reactivity patterns and the overall mechanism. Finally, preliminary biological data are included on the antibacterial properties of these compounds.

## Graphical abstract

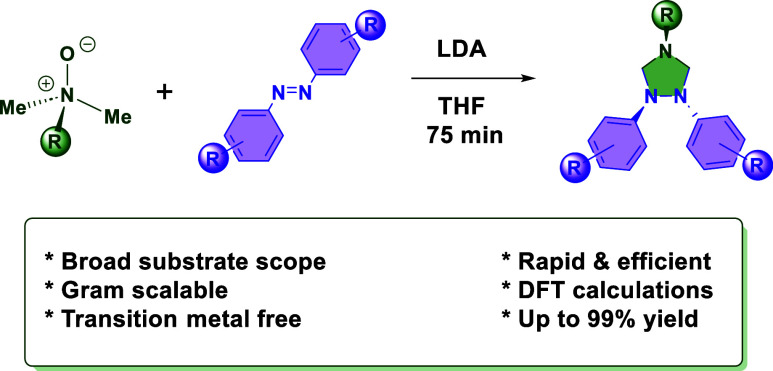

## Introduction

Nitrogen-containing heterocycles (*N*-heterocycles) are a ubiquitous class of molecules that frequently exhibit medicinal properties, making them of significant interest to both the synthetic and pharmaceutical communities.^1,2^ While many families of *N*-heterocycles, such as indoles,^3–5^ pyrroles,^6^ and piperidines,^7^ have seen significant attention, 1,2,4-triazolidines have not been widely investigated. The first syntheses of 1,2,4-triazolidines were reported by Schmitz^8^ and Strating^9,10^ via a condensation reaction between hydrazines and aldehydes (Scheme 1A). Early efforts from McCarthy and Murphy,^11^ Heine,^12^ Grigg,^13^ and Mlostoń^14^ demonstrated the possibility of accessing these compounds via cycloaddition reactions. Following these initial reports, several groups have utilized either transition-metal catalysis^15–17^ or electrochemical methods^18,19^ to synthesize triazolidines (Scheme 1B).^20^ More recently, Yang et al.^21,22^ and Dell’Amico et al.^23^ have developed effective photochemical routes for synthesizing 1,2,4-triazolidines. Yang’s 2021 article utilized a decarboxylative approach using *N*-aryl glycine and azoarenes in the presence of methylene blue to afford 1,2,4-triazolidines in excellent yields.^21^ They later expanded on this work to generate 1,2,4-triazolidines using rose bengal (Scheme 1C).^22^ Taking inspiration from these prior works, we decided to develop an orthogonal, transition-metal-free approach to synthesize 1,2,4-triazolidines utilizing *N*-oxides and azoarenes as readily available precursors.

**Scheme 1:**
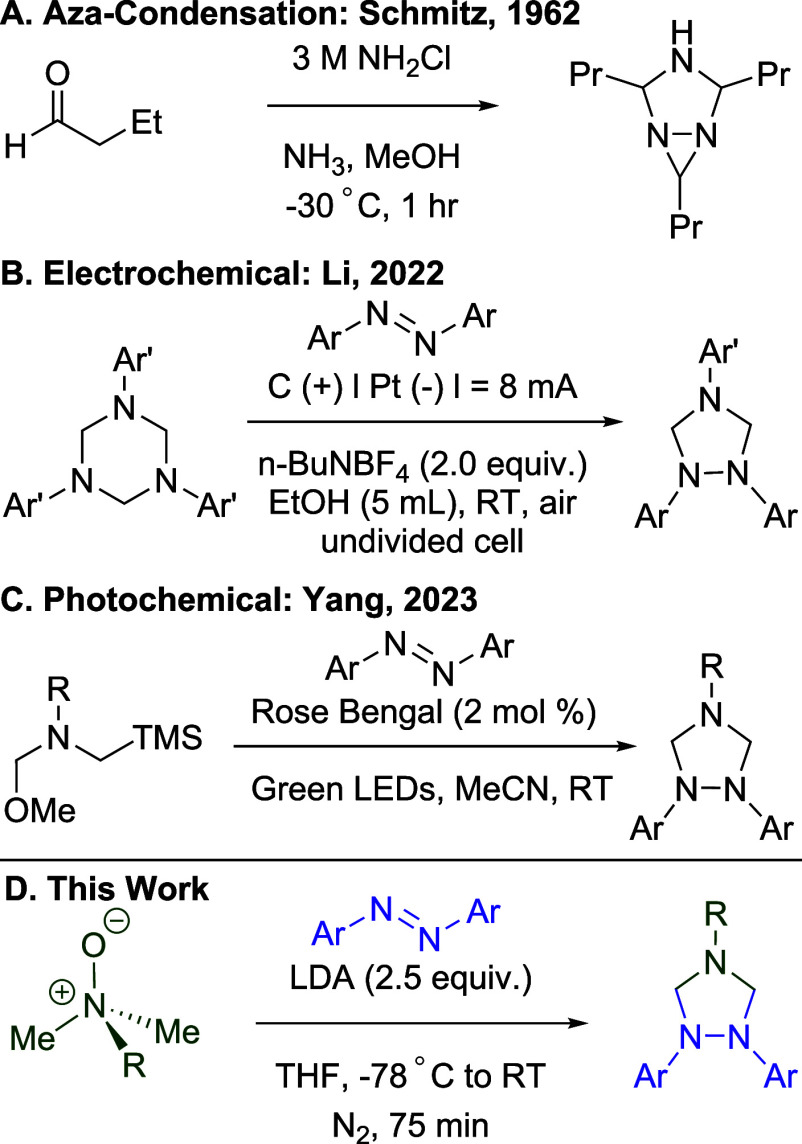
1,2,4-Trazolidine Syntheses

Roussi et al. first reported on how tertiary amine *N*-oxides can serve as effective precursors for forming azomethine ylides and generated a range of pyrrolidines through a [3 + 2] cycloaddition with suitable dipolarophiles.^24–27^ Subsequent work by Davoren^28^ and Williams^29^ expanded this chemistry, generating 3,4-bisubstituted pyrrolidines and pyrroles, respectively. Prior work in our group has expanded the scope of *N*-heterocycles that this chemistry can generate to include ethylene diamines^30^ and azanorbornanes.^31^ Given that both alkenes and imines serve as competent dipolarophiles, we hypothesized that azoarenes would be similarly tolerated in this reaction. Azoarenes, or closely related compounds, have frequently served as one of the reagents when synthesizing 1,2,4-triazolidines.^11–19,21,22^ This is unsurprising, given how azoarenes represent a natural synthon for creating 1,2,4-triazolidines. More generally, azoarenes have been widely used in other *N*-heterocyclic forming reactions^32,33^ including triazoles,^34–38^ indazoles,^39–50^ carbazoles,^51,52^ and others.^53–56^

Despite the comparatively small number of literature examples for the synthesis of 1,2,4-triazolidines, this family of molecules exhibits properties of significant interest to both the biomedical and materials science communities. Specifically, molecules containing the 1,2,4-triazolidine core have shown to act as antifungal,^57,58^ antiviral,^59–61^ antibacterial,^62,63^ and anticancer agents.^17^ Additionally, Michael and co-workers reported 1,2,4-triazolidine-containing compounds to have anticorrosive properties.^64,65^ Given the potential versatility of these compounds, it is surprising that more methods do not exist to access these understudied but highly promising *N*-heterocyclic cores. In this context, we aim to bridge this gap by developing a comprehensive synthetic methodology that enables the generation of a wide spectrum of both *C*2 symmetric and asymmetric 1,2,4-triazolidines.

## Results and Discussion

### Reaction Optimization

To test this, we combined trimethylamine *N*-oxide (TMAO) and lithium diisopropylamide (LDA) in our group’s previously developed reaction conditions to generate the presumed azomethine ylide intermediate.^25,30,31,66,67^ This was then reacted with azobenzene to generate 1,2,4-triazolidine **3a** in a 4% yield (Table 1, entry 1). We hypothesized that this low yield was likely due to an acid-initiated fragmentation pathway, similarly to when silyl imines were used as dipolarophiles.^30^ Forgoing acidic or basic workup produced triazolidine **3a** in 45% isolated yield (Table 1, entry 2). Subsequent reaction optimization (Table 1) examined solvent concentration and equivalents of LDA, ultimately producing triazolidine **3a** in a 94% yield after 75 min (Table 1, entry 5).

**Table 1:**
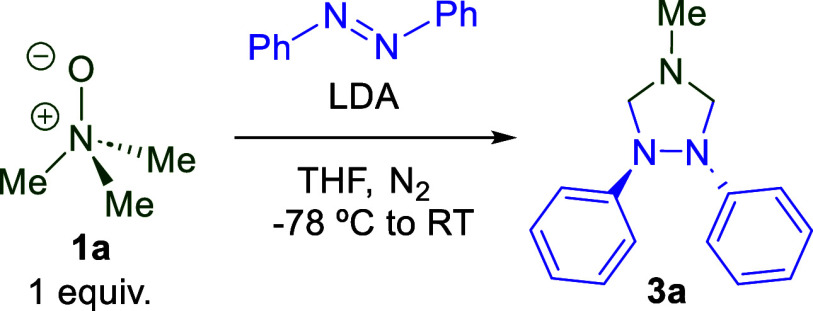
Optimization of Reaction Conditions^a^

entry	LDA [equiv]	THF [M]	time (min)	yield (%)^b^
1	3	0.1	60	4^c^
2	3	0.1	60	45
3	1	0.1	75	48
4	2	0.1	75	63
**5**	2.5	0.1	75	94
6	2.5	0.08	75	55
7	2.5	0.15	75	74
8	2.5	0.1	60	78
9	2.5	0.1	120	53

a Reactions were conducted on a 0.8 mmol scale.

b Yields were calculated from ^1^H nuclear magnetic resonance (NMR) spectra using 1,3,5-trimethoxybenzene (TMB) as an internal standard.

c Isolated yield after acid workup.

### Substrate Scope

We then generated 13 *C*2-symmetric and 10 *C*2-asymmetric azoarenes via a one-step synthesis from commercially available starting materials. Symmetric azoarenes were formed through manganese-mediated oxidative homocoupling of the corresponding anilines.^68,69^ Asymmetric azoarene starting materials were generated using a Baeyer–Mills condensation of nitrosobenzene and corresponding aniline.^70,71^ These azoarenes were then used as substrates under the optimized reaction conditions to establish a substrate scope for the formation of 1,2,4-triazolidines (Scheme 2).

**Scheme 2:**
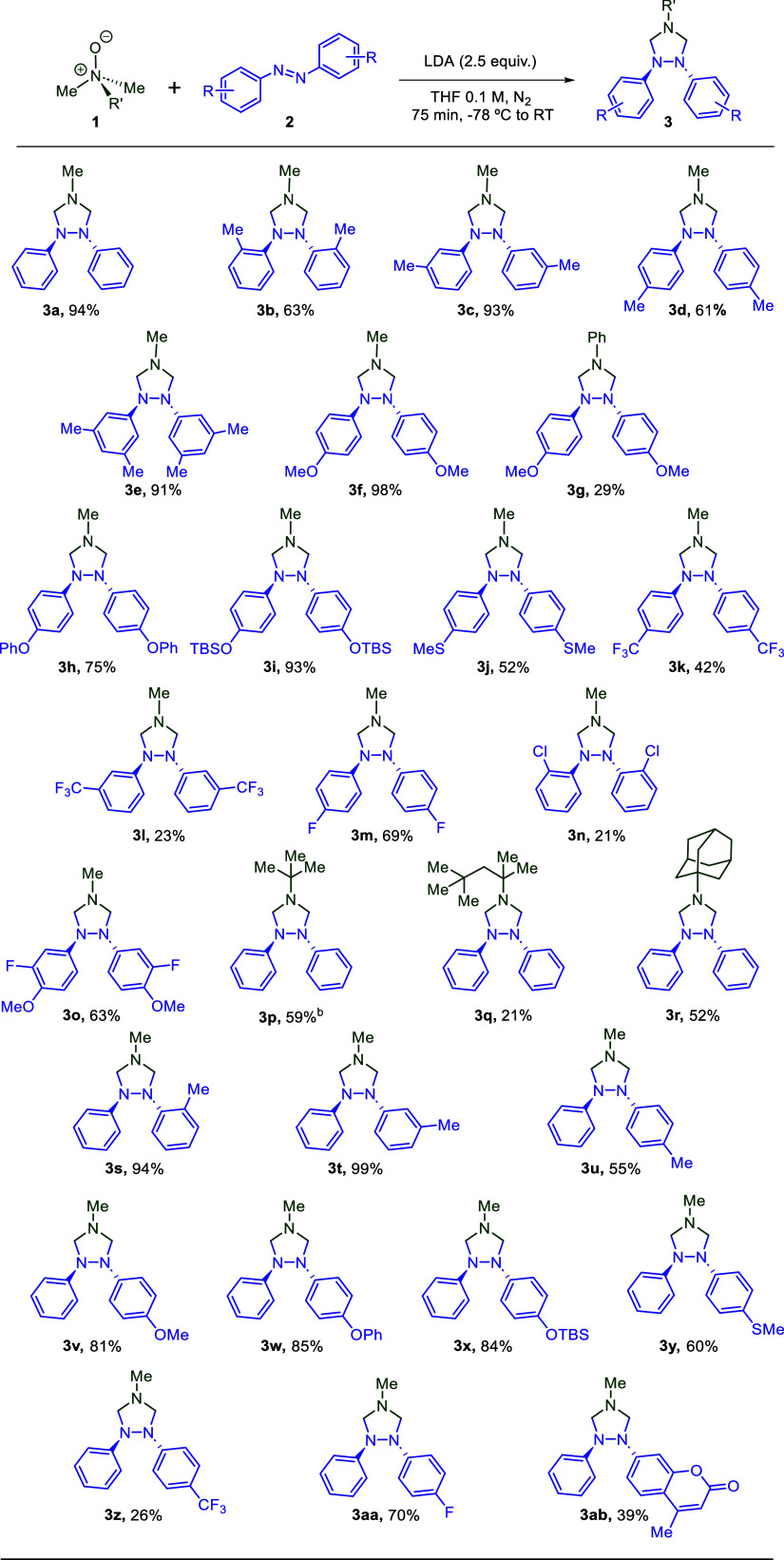
Substrate Scope for 1,2,4-Triazolidines^a^

a Reactions carried out in a 0.4 mmol scale. Conditions: *N*-Oxide (2.0 equiv), LDA (2.5 equiv), THF (0.1 M), azoarene (1.0 equiv), and from −78 °C to room temperature (RT). Isolated yields are reported.
b Isolated as an intractable product mixture with a minor hydrazine product (See the Supporting Information entry)

We initiated our studies by varying the substitution patterns on the aromatic rings to investigate reactivity trends for the *C*2 symmetric products. Introduction of methyl groups at the *ortho* (**3b**) and *meta* (**3c**) positions furnished the corresponding triazolidine products in modest to excellent yields. The diminished yield of **3b** was likely the result of both methyl groups’ proximity to the nitrogen–nitrogen double bond, which would have sterically hindered the ylide during the cycloaddition reaction. In contrast, the para-substituted derivative **3d** provided diminished yields. The addition of electronically neutral steric bulk to all meta sites on the aromatic rings did not hinder the cycloaddition, affording product **3e** in an excellent yield. Symmetric para-substituted methoxy derivative **3f** was obtained in excellent yield; however, the yield was greatly diminished upon the substitution of the methyl group at the 4 position of the triazolidine moiety with a mildly electron-withdrawing phenyl ring (**3g**), revealing the sensitivity of our chemistry to the electronics of the azomethine ylide. Phenoxy (**3h**) and siloxy (**3i**) triazolidines were obtained in high yields, further illustrating the positive effect of electron-rich substituents on the azoarene. Notably, the bis-siloxy-substituted triazolidine (**3i**) could be synthesized on a gram scale in 75% isolated yield, demonstrating the scalability of the transformation. *Para*-thio analogue **3j** was afforded in moderate yield, showing a similar positional effect as was observed in **3d**. Electron-poor substituents **3k**, **3l**, and **3m** were isolated in fair to moderate yields, indicating decent tolerance of fluoro substituents. Remarkably, **3n** was isolated, albeit in poor yields, but nonetheless demonstrating tolerance of our chemistry to nonfluoro halogen substituents. This was an unexpected result given that nonfluoro halogens typically display instability under these reaction conditions, likely due to lithium-halogen exchange or ortho-lithiation.^72–74^

To further explore the electronic tolerance of this methodology, **3o** was synthesized in a fair yield. Substitution of the parent methyl group at the 4 position of the triazolidine moiety with a *tert*-butyl substituent had a moderately negative effect on the yield (**3p**), indicating that our chemistry was sensitive to the steric bulk of the azomethine ylide as well as the electronics thereof. Further increasing the steric bulk at position 4 of the triazolidine ring, through the addition of a *tert*-octyl group at this position (**3q**) had a strong negative effect on reaction outcome despite its slightly more electron-donating nature compared to the methyl (**3a**) and *tert*-butyl (**3p**) analogues. To further investigate substitution at this position, we added a sterically demanding adamantyl group (**3r**) to the 4 position, which gave a similarly modest yield as **3p**.

*C*2 asymmetric **3s**, **3t**, and **3u** demonstrated comparable reactivity trends with respect to those of their *C*2 symmetric analogues. We were able to generate **3s** and **3t** in excellent yields, whereas **3u** was generated in a similarly modest yield as **3d**. As was observed with the symmetric products, electron-rich **3v**, **3w**, and **3x** were all isolated in good yield. For the *para*-thiomethyl analogue **3y**, a similar outcome was observed for the symmetric variant **3j**. Similarly, for the symmetric system, the strongly electron-withdrawing trifluoromethyl substituent afforded the corresponding triazolidine **3z** in fair yield. Interestingly, **3aa** was tolerated similarly to **3m** by our chemistry, further indicating a good tolerance to fluorinated substituents. The ester-containing product **3ab** was formed in a modest yield.

### Mechanistic Studies

Following the establishment of a substrate scope, we turned our attention to a plausible mechanism for this reaction. Originally, Roussi and co-workers suggested that this reaction proceeded through deprotonation and loss of lithium monoxide anion, generating an iminium intermediate (Scheme 3A). This iminium is then deprotonated by a second equivalent of LDA to yield an azomethine ylide intermediate.^27,75^ In a different report, Roussi and co-workers also suggested that this reaction may proceed through a diradical pathway, which could then form cyclic products such as **3a** through a stepwise mechanism (Scheme 3B).^76^ Our prior computational analysis of this reaction indicated that the azomethine ylide is likely formed directly from the *N*-oxide via a multi-ion bridge intermediate following the loss of lithium oxide, as opposed to moving through an iminium intermediate.^66,67^ The resulting azomethine ylide then undergoes a concerted asynchronous [3 + 2] cycloaddition to generate **3a** (Scheme 3C).

**Scheme 3:**
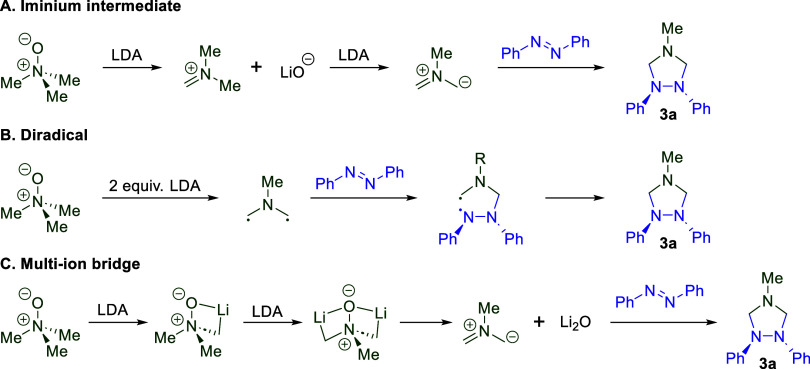
Proposed Reaction Mechanisms

To determine the most likely mechanism, we first attempted to trap any radical intermediates using TEMPO and BHT (Table 2).^77–79^ When both TEMPO and BHT were added, we saw good conversion of the starting material to the expected triazolidine product (**3a**), though with significantly decreased isolated yields. These results were further complicated by the controls (Table 2, entries 3–7) where we removed TMAO from the reaction mixture. When just LDA and azobenzene were combined, we observed minor amounts of azobenzene decomposition but could recover most of the starting material (Table 2, entry 3). When azobenzene was combined with TEMPO and BHT in the absence of LDA, no reaction was observed (Table 2, entries 4 and 5). However, when either TEMPO or BHT was added in the presence of LDA, the azobenzene completely decomposed after 75 min (Table 2, entries 6 and 7). This suggested that the lower isolated yields in Table 2 (entries 1 and 2) could be attributed to either decomposition of the azobenzene starting material or radial inhibition.

**Table 2:**
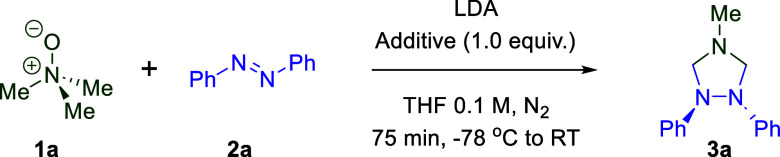
Mechanistic Experiments

entry	equiv **1a**	equiv LDA	additive	yield
1	1	2.5	TEMPO	(>80)^a^, 20%
2	1	2.5	BHT	(>80)^a^, 45%
3	0	2.5	none	minor decomposition
4	0	0	BHT	NR
5	0	0	TEMPO	NR
6	0	2.5	TEMPO	decomposition
7	0	2.5	BHT	decomposition

a Conversion based off the unreacted starting material.

Given that intermolecular radical trapping was inconclusive, we synthesized *N*-oxide **4** with a methylene cyclopropyl appendage (Scheme 4). We hypothesized that if there was a significant radical character in the precyclization intermediate, intramolecular cyclopropyl ring opening should outcompete the intermolecular reaction with azobenzene.^80^ Using our standard conditions, we were able to form the 2-cyclopropyl-substituted triazolidine **5** in low yields as the sole product, suggesting that the azomethine ylide is the major mechanistic pathway (Scheme 3). Finally, we attempted to form triazolidine **5** starting from Eschenmoser’s salt **6**, an analog for the postulated iminium intermediate (Scheme 5). This did not produce any amount of cycloaddition product after 75 min.

**Scheme 4:**
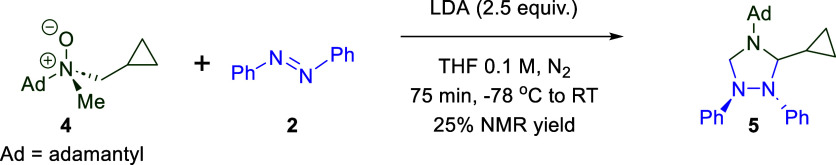
Cyclopropyl *N*-Oxide Cycloaddition

**Scheme 5:**
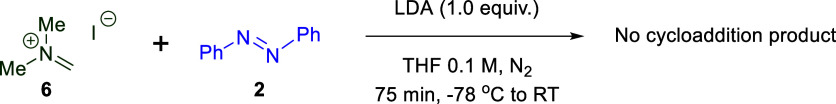
Attempted [3 + 2] Reaction Using Eschenmoser’s Salt

### DFT Calculations

The experimental data agree with our computational analysis comparing the diradical and azomethine ylide pathways (Figure 1), which shows that the diradical is 30.4 kcal/mol higher in free energy than the azomethine ylide. Quantum calculations were carried out using the M06-2X density functional^81^ with the Dunning’s correlation consistent maug-cc-pv­[D,T,Q] basis sets.^82^ Free energies were corrected using Whitesides’ free volume model for translational entropy.^83^ Based on our experimental results and computational data,^66,67^ we suggest that the reaction likely proceeds through formation of an azomethine ylide via a multi-ion bridge. The azomethine ylide then reacts with an available dipolarophile, such as azobenzene, through a concerted asynchronous cycloaddition reaction (Figure 1, Blue path).

**Figure 1:**
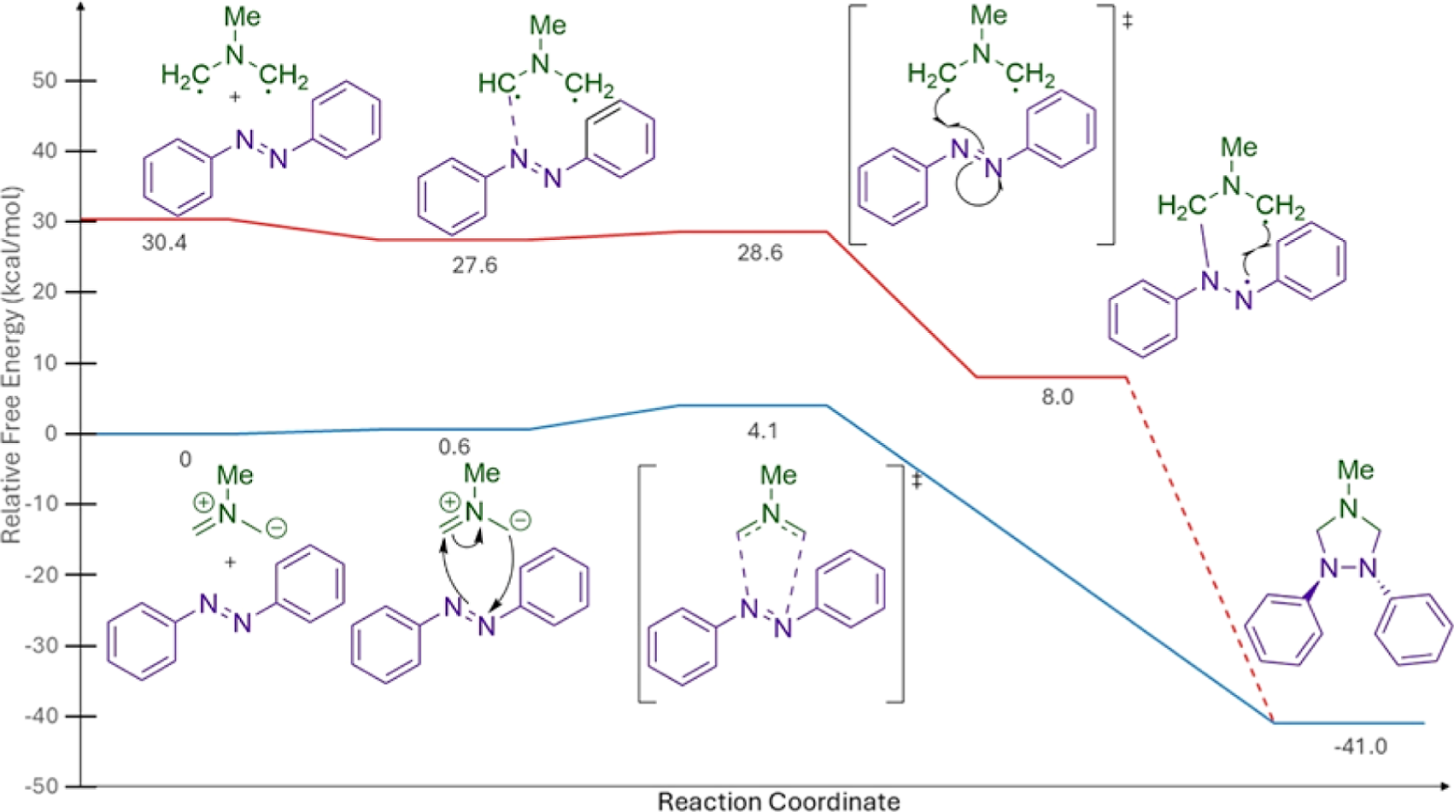
Free volume-corrected free energy diagram^a^ ^a^[3 + 2] cycloaddition (single, blue) versus diradical mechanism (triplet, red) in kcal/mol using maug-cc-pv­[D,T,Q]­z extrapolated to the complete basis set (CBS).^64^

### Biological Studies

Recent work has highlighted the potential of 1,2,4-triazolidines as antimicrobial scaffolds.^85^ Multiple investigations have identified 1,2,4-triazolidine derivatives as potent antiviral compounds, with effective concentrations in the low nanomolar to micromolar ranges.^60,86–89^ Kanagarajan et al. synthesized novel 1,2,4-triazolidines that demonstrated antifungal activities against *Aspergillus niger* and *Candida albicans* as well as antibacterial properties against *Bacillus subtilis* at concentrations as low as 6.25 μg/mL.^90^ Triazolidine-containing antibiotics have been synthesized that are potent against both Gram-negative and Gram-positive pathogens, with ng/mL to μg/mL minimum inhibitory concentrations.^85^ Most of these antibiotics require the addition of large, bulky substitutions or fusion to additional cyclic structures for activity.

For our substrate scope, we obtained preliminary data on the antibacterial potential of these compounds against common food-borne Gram-positive *Listeria monocytogenes*, and Gram-negative pathogens *Salmonella typhimurium* (*S*Tm), *Escherichia coli* (*E. coli*), and *Shigella flexneri* (Table 3). Kirby–Bauer disk assays were performed using a logarithmic, 2-fold dilution of each compound to determine the minimum amount of compound that inhibited bacterial growth.^91^ We found that **3a**, **3b**, **3f**, and **3v** were broadly antibacterial with detectable zones of inhibition against all four pathogens (Table 3).

**Table 3:** Antibacterial Potential^a^ of Trazolidines^b^

compound	*Listeria*	*S*Tm	*Shigella*	*E. coli*
**3a**	200	50	100	200
**3b**	200	100	100	100
**3d**	^c^	-	200	-
**3e**	-	200	100	-
**3f**	200	100	50	100
**3m**	200	200	100	-
**3s**	-	50	50	50
**3t**	-	100	50	-
**3u**	-	200	100	-
**3v**	200	50	50	100
**3x**	-	-	200	-
**3z**	-	-	200	-
**3aa**	-	200	100	-
**3ab**	-	100	-	-
chloramphenicol	2.5	1.25	0.25	2.5

a Potential is defined as the presence of a zone of inhibition at the indicated mg of compound.

b Compounds showing no activity to any strains are omitted for clarity but included in the Supporting Information.

c “-” Indicates that no zone of inhibition was observed up to the highest amount (200 μg) tested.

Interestingly, **3s** inhibited the growth of Gram-negative, but not the Gram-positive, pathogens (Table 3). Switching the methyl group from the ortho position to the meta (**3c**, **3e**, and **3t**) or para (**3d** and **3u**) position either reduced or eliminated the ability of these compounds to kill most of the pathogens (Table 3). Both **3f** and **3v** retained antibiotic activity, while **3g**, **3h**, **3i**, **3j**, **3k**, **3l**, **3n**, **3o**, **3p**, **3q**, **3r**, **3w**, and **3y** exhibited no activity (see the Supporting Information). Additionally, substitution of the methyl group at the 4 position of the triazolidine ring with a phenyl group (**3f** and **3g**) also eliminated the activity of the compound against all tested pathogens. Para-fluoro-substituted compounds (**3m** and **3aa**) showed modest activity. Finally, ester-containing triazolidine **3ab** showed some activity against *S*Tm only. This suggests, in addition to having sterically small, electron neutral, or donating groups at the ortho/para positions of the azoarene portion of the molecule, that the substituent at the 4 position of the triazolidine ring contributes to the antibacterial properties of these molecules. We performed the same assays with the broad-spectrum antibiotic chloramphenicol as a positive control and point of comparison (Table 3). As expected, chloramphenicol inhibited bacterial growth at a concentration of 2.5 mg or less, significantly lower than even our most active compounds. Future studies will continue from compounds **3s** and **3v**, which showed the highest activities.

## Conclusions

In summary, we developed a [3 + 2] cycloaddition reaction between tertiary amine *N*-oxides and azoarenes to produce substituted 1,2,4-triazolidines in modest to excellent yields. We generated 29 novel substrates and showed reasonable functional group tolerance to both sterics and electronics while making use of easily prepared or commercially available starting materials. Mechanistic investigations suggest that the reaction proceeds through an azomethine ylide that is not formed from iminium deprotonation. Preliminary biological testing indicates that four of these compounds exhibit antibacterial activity against both Gram-positive and Gram-negative bacteria, providing a basis for further optimization to improve their efficacy to be comparable to other broad-spectrum antibiotics such as chloramphenicol. This method provides a powerful route toward accessing this family of *N*-heterocycles and enables new lines of investigation.

## Experimental Methods

All materials were used as purchased from MilliporeSigma, Thermo Fisher Scientific, TCI, Ambeed, or Oakwood Chemical, unless otherwise noted. Tetrahydrofuran (THF) was dried by a column of activated alumina via an Inert PurSolv Solvent System and subsequently stored over activated 4 Å molecular sieves. Tertiary amine *N*-oxides were stored under rigorous anhydrous conditions in a desiccator with Drierite and phosphorus pentoxide. −78 °C cooling baths were achieved using dry ice in acetone. Solutions of LDA were titrated using salicylaldehyde phenylhydrazone before use.^92^

### NMR Spectroscopy

NMR spectra were recorded on a Bruker AVANCE 400 or AVANCE II 500 MHz spectrometer. ^1^H NMR spectra were calibrated from standard TMS (δ 0.00) or solvent resonance (CDCl_3_: δ 7.27, MeOD: δ 3.31). ^13^C NMR spectra were calibrated from solvent resonance (CDCl_3_: δ 77.16, MeOD: δ 49.00).

### Mass Spectrometry

High-resolution mass spectrometric data was recorded on an Agilent Technologies 6530 Accurate-Mass QTOF LC/MS equipped with an Agilent Technologies 1200 series LC system.

### Infrared Spectrometry

Infrared (IR) spectral analysis was performed on a Thermo Scientific Everest ATR.

### Reaction Monitoring and Purification

Reactions monitored by thin layer chromatography (TLC) used TLC silica gel 60 F_254_ and were visualized under a 4-W 254/365 nm UV lamp. Flash column chromatography (FCC) (EtOAc/Hex) was performed using a Biotage Isolera One Flash Chromatography instrument with a 10 or 25 g Biotage Sfär Silica D-Duo 60 μm column. An IKA heating mantel was used as the heat source for transformations that required heating.

### Cautionary Note Regarding Lithium Diisopropylamide Usage

LDA is pyrophoric and highly corrosive. Proper personal protective equipment including eye protection, gloves, and a lab coat should be worn at all times while working with LDA. In this paper, LDA was handled under an inert atmosphere using the Schlenk technique to prevent exothermic reactions with air or moisture.

### General Procedure A for the Preparation of Tertiary Amines

A magnetic stir bar and formic acid (3.0 mL, 80 mmol, 8 equiv) were added to a round-bottom flask immersed in an ice/water bath cooled to 0 °C and set stirring. Primary amine (10 mmol, 1 equiv) was then added over the course of 10 min. Upon complete addition, the mixture was heated to 70 °C for 10 min, after which 37% aqueous formaldehyde solution (3.3 mL, 33 mmol, 3.3 equiv) was added. A reflux condenser was then fixed to the round-bottom flask, and the reaction mixture was heated to 100 °C for 1.5 h. The reaction mixture was removed from heat and allowed to cool to room temperature (RT), and the aqueous solution was washed with Et_2_O three times. The aqueous layer was then basified with 1 M NaOH_(aq)_ (pH > 10) and extracted three times with Et_2_O. The organic layers were then combined, dried with Na_2_SO_4_, filtered, and concentrated by rotary evaporation to yield pure product.

### General Procedure B for the Preparation of Tertiary Amine *N*-Oxides

The corresponding tertiary amine was dissolved in methanol (2 M) in a round-bottomed flask containing a magnetic stir bar. The round-bottomed flask was then sealed with a rubber septum and vented with a needle. The mixture was then set to stir. Over the course of 10 min, 30% H_2_O_2(aq)_ (3 equiv) was added via a syringe through the septum. The reaction mixture was then allowed to stir at RT and monitored by TLC or ^1^H NMR. After reaction completion, volatiles were removed by rotary evaporation at 40 °C for 30 min, and the residue was further dried under Schlenk line vacuum for 8 h to yield the pure product. Attention: It is critical for subsequent reactions that water is completely removed.

### General Procedure C for the Preparation of Symmetric Diazenes

1.0 g of aniline was dissolved in 50 mL of toluene, and to this solution, 10 equiv of activated MnO_2_ was added. The mixture was then heated to 90 °C and monitored by TLC. The reaction was run until the starting material was consumed by TLC (1–16 h). Following the consumption of the starting material, the reaction mixture was filtered through a plug of silica, and the silica plug was washed with hexanes. The solvent was removed by rotary evaporation, and the resulting mixture was purified by FCC.

### General Procedure D for the Preparation of Asymmetric Diazenes

To a solution of nitrosobenzene in ethanol (5 mL) and glacial acetic acid (0.3 mL) was added aniline (1.0 equiv). The reaction was stirred for 12 h. The reaction mixture was then diluted with ethanol and water, followed by ethyl acetate extraction. This organic layer was dried over Na_2_SO_4_, and ethyl acetate was gently removed under reduced pressure. Purification by FCC was carried out as needed.

### General Procedure E for the Preparation of Symmetric Triazolidines 3a–3r

Dry tertiary amine *N*-oxide (0.5 mmol, 1.0 equiv) was added to an oven-dried test tube charged with a magnetic stir bar. The test tube was then sealed, purged, and flushed with dry nitrogen. 5.0 mL (0.1 M) of dry THF was then added to the test tube via a dry syringe. The reaction test tube was stirred at ambient temperature until the *N*-oxide was completely dissolved. The reaction tube was then immersed in an acetone/dry ice bath and cooled to −78 °C. 1.6 M LDA (2.5 equiv) in THF was then added dropwise over the course of 10 min via a syringe. Immediately after LDA addition was completed, azobenzene (37.0 mg, 0.2 mmol, 0.5 equiv) was dissolved in 1.0 mL (0.2 M) of dry THF and added to the test tube dropwise over 1 min. Following addition, the reaction flask was removed from the cold bath, allowed to warm to ambient temperature, and stirred for a total of 75 min; the reaction mixture was quenched with DI H_2_O and extracted three times with Et_2_O (10 mL). The organic layer was dried with Na_2_SO_4_, filtered, and concentrated by rotary evaporation. The product was then purified using FCC with a normal phase gradient on silica gel.

### General Procedure F for the Preparation of Asymmetric Triazolidines **3s–3ab**

Dry triethylamine *N*-oxide (TMAO) (60.0 mg, 0.8 mmol, 1.0 equiv) was added to an oven-dried test tube charged with a magnetic stir bar. The test tube was then sealed, purged, and flushed with dry nitrogen. 6.0 mL (0.133 M) of THF was then added to the test tube via a dry syringe, and the mixture was set stirring. The reaction test tube was stirred at ambient temperature until TMAO was completely dissolved. It was then immersed in an acetone/dry ice bath and cooled to −78 °C. 1.6 M LDA in THF was then added dropwise over the course of 10 min via a syringe. Immediately after LDA addition was completed, azobenzene (73.0 mg, 0.4 mmol, 0.5 equiv) dissolved in 1.0 mL (0.1 M) of dry THF was added to the test tube dropwise over ca. 1 min. Following complete addition, the reaction was removed from the cold bath and allowed to warm to ambient temperature and stirred for 75 min. At the end of the reaction time, the reaction mixture was concentrated by rotary evaporation. The crude residue was then purified using FCC using a normal phase gradient on silica gel.

### Gram-Scale Synthesis of 1,2-Bis­(4-((*tert*-butyldimethylsilyl)­oxy)­phenyl)-4-methyl-1,2,4-triazolidine (**3i**)

Triazolidine was prepared according to general procedure E using trimethylamine *N*-oxide (341 mg, 4.52 mmol, 1.0 equiv), (E)-1,2-bis­(4-((*tert*-butyldimethylsilyl)­oxy)-phenyl)­diazene (1.10 g, 2.26 mmol, 0.5 equiv), 1.47 M LDA (7.69 mL, 11.3 mmol, 2.5 equiv), and dry THF (22.6 mL, 0.2 M). This was followed by purification by FCC on silica gel (EtOAc in Hexanes mobile phase) to isolate the product as a yellow oil (75% yield, 852 mg, 1.704 mmol).

### Bacterial Strains

*Salmonella enterica* Typhimurium SL1344 effectorless,^93^ *E. coli* DH5α, and *S. flexneri* M90T^94^ were grown using Lysogeny Broth media. *L. monocytogenes* 10403S Δhly^95^ was grown using Brain Heart Infusion media. All strains were gifts from Neal Alto (University of Texas Southwestern Medical School) or Dan Portnoy (University of California-Berkley).

### Zone of Inhibition Assays

Inhibition of bacterial growth on solid medium was performed as previously described.^91^ Briefly, lag phase bacteria were spread across a solid agarose medium, and then a 4 mm Whatman filter disk containing the indicated amount of compound, or 2 mL of DMSO, was placed on top of the agarose. After 16–18 h, the zone of growth inhibition, or the area around the disk without bacterial growth, was manually measured using calipers.

## General Computational Methods

The quantum chemistry method of meta-hybrid density functional theory (DFT)^96^ was carried out at the Center for Computational Sciences (CCS) at Duquesne University using Gaussian 16.^97^ The M06-2X functional^81^ with Dunning’s maug-cc-pv­[D,T,Q]­z basis sets^82^ were used to calculate electronic, enthalpic, and free energies for both ground and transition structures. These energies were then extrapolated to the CBS limit, which is not a basis set but rather an extrapolated estimate of a result using an infinitely large basis set.^84^ The procedure removes any error from the linear combination of atomic orbitals approximation. Unrestricted M06-2X was used for triplet state calculations, and the spin expectation values of these calculations are given. The use of M06-2X, developed by Truhlar and co-workers, has been reported to be accurate to within 1.2 kcal/mol for reaction barriers and within 0.37 kcal/mol of noncovalent interaction energies.^81^ Vibrational frequency calculations were used to confirm all stationary points as either minima or transition structures and to provide thermodynamic corrections for the enthalpies and free energies. The free volume method developed by Whitesides et al.^83^ was used to correct for translational entropy. A standard state of 1 M was assumed for all of the species.

## Supplementary Material





## Data Availability

The data underlying this study are available in the published article and its Supporting Information.

## Notes

FAIR Data available includes the primary NMR FID files for compounds **2o**, **2ab**, **3a**-**3ab**, **4**, **5**, **SI**-**6**, **SI**-**8**, **SI**-**9**, and **SI**-**10** (ZIP)
Detailed experimental procedures, compound characterization data, copies of NMR spectra for new compounds, computational data, and biological data (PDF)
