# Supplementary figures and images for: [3 + 2] Cycloadditions of Tertiary Amine N‑Oxides and Azoarenes as a Route to Substituted 1,2,4-Triazolidines
Source: ACS Org Inorg Au. 2025 Dec 5;6(1):119–29. doi: 10.1021/acsorginorgau.5c00098 (PMC12879182; doi:10.1021/acsorginorgau.5c00098)

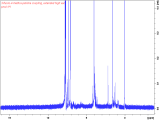

Supplement: Supplementary file 1 [file gg5c00098_si_001.zip › FID for Publication/2o (E)-1,2-bis(3-fluoro-4-methoxyphenyl)diazene/2o_1H/pdata/1/thumb.png]

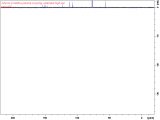

Supplement: Supplementary file 1 [file gg5c00098_si_001.zip › FID for Publication/2o (E)-1,2-bis(3-fluoro-4-methoxyphenyl)diazene/2o_13C/pdata/1/thumb.png]

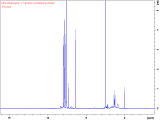

Supplement: Supplementary file 1 [file gg5c00098_si_001.zip › FID for Publication/2ab (E)-4-methyl-7-(phenyldiazenyl)-2H-chromen-2-one/2ab_1H/pdata/1/thumb.png]

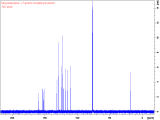

Supplement: Supplementary file 1 [file gg5c00098_si_001.zip › FID for Publication/2ab (E)-4-methyl-7-(phenyldiazenyl)-2H-chromen-2-one/2ab_13C/pdata/1/thumb.png]

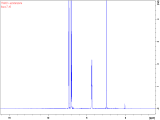

Supplement: Supplementary file 1 [file gg5c00098_si_001.zip › FID for Publication/3a 4-methyl-1,2-diphenyl-1,2,4-triazolidine/3a_1H/pdata/1/thumb.png]

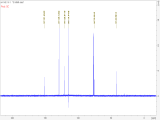

Supplement: Supplementary file 1 [file gg5c00098_si_001.zip › FID for Publication/3a 4-methyl-1,2-diphenyl-1,2,4-triazolidine/3a_13C/pdata/1/thumb.png]

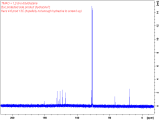

Supplement: Supplementary file 1 [file gg5c00098_si_001.zip › FID for Publication/3b 4-methyl-1,2-di-o-tolyl-1,2,4-triazolidine/3b_13C/pdata/1/thumb.png]

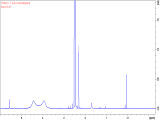

Supplement: Supplementary file 1 [file gg5c00098_si_001.zip › FID for Publication/3b 4-methyl-1,2-di-o-tolyl-1,2,4-triazolidine/3b_1H/pdata/1/thumb.png]

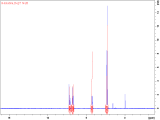

Supplement: Supplementary file 1 [file gg5c00098_si_001.zip › FID for Publication/3c 4-methyl-1,2-di-m-tolyl-1,2,4-triazolidine/3c_1H/pdata/1/thumb.png]
