# Supplementary material for: [3 + 2] Cycloadditions of Tertiary Amine N‑Oxides and Azoarenes as a Route to Substituted 1,2,4-Triazolidines
Source: ACS Org Inorg Au. 2025 Dec 5;6(1):119–29. doi: 10.1021/acsorginorgau.5c00098 (PMC12879182; doi:10.1021/acsorginorgau.5c00098)
Supplement: Supplementary file 2 [file gg5c00098_si_002.pdf]

# [3+2] Cycloadditions of Tertiary Amine N-Oxides and Azoarenes as a Route to Substituted 1,2,4-Triazolidines

## Supporting Information

Nicholas A. Frankos, Malavika S. Nair, Aiden M. Lane, Megan M. Glista, Joshua K. Graber, Abbigail E.F. Black, Trista G.L.X. Newman, Elias R. Griffin, Kiera M. Luca, Eric J. Chartier, David B. Heisler, and Thomas D. Montgomery*

Department of Chemistry and Biochemistry, Duquesne University, Pittsburgh Pennsylvania 15282, United States

Experimental General Information S2

Compound Characterization S6

$^{1}H$, $^{13}C$, and $^{19}F$ NMR Spectra S34

Biological Studies S78

Computational Data S81

References S119

## Experimental General Information

Nuclear magnetic resonance (NMR) spectra were measured on a Bruker AVANCE™ 400 MHz or AVANCE™ II 500 MHz spectrometer. $^{1}H$ NMR spectra were calibrated from standard TMS (δ 0.00) or solvent resonance ($CDCl_3$: δ 7.27, MeOD: δ 3.31). $^{13}C$ NMR spectra were calibrated from solvent resonance ($CDCl_3$: δ 77.16, MeOD: δ 49.00). High-resolution mass spectrometric analysis (HRMS) was measured on an Agilent Technologies 6530 Accurate-Mass QTOF LC/MS equipped with the Agilent Technologies 1200 series LC system. Infrared (IR) spectral analysis was performed on a Thermo Scientific Everest ATR. Reactions monitored by thin layer chromatography (TLC) used TLC silica gel 60 $F_{254}$ and visualized under a 4-Watt 254/365 nm UV lamp. Flash column chromatography (FCC) (EtOAc/Hex) was performed using a Biotage Isolera One Flash Chromatography instrument with a 10 g or 25 g Biotage® Sfär Silica D- Duo 60 µm column. An IKA heating mantel was used as the heat source for transformations that required heating.

## Materials

All materials were used as purchased from MilliporeSigma, Thermo Fisher Scientific, TCI, Ambeed, or Oakwood Chemical, unless otherwise noted. Tetrahydrofuran (THF) was dried by a column of activated alumina via an Inert PurSolv Solvent System and was subsequently stored over activated 4 Å molecular sieves. Tertiary amine *N*-oxides were stored under rigorous anhydrous conditions in a desiccator with Drierite and phosphorus pentoxide. -78 °C cooling baths were achieved using dry ice in acetone. Solutions of lithium diisopropylamide (LDA) were titrated using salicylaldehyde phenylhydrazone before use.[1]

## Cautionary Note Regarding Lithium Diisopropylamide Usage

LDA is pyrophoric and highly corrosive. Proper personal protective equipment (PPE) including eye protection, gloves, and a lab coat, should be worn at all times while working with LDA. In this paper, LDA was handled under inert atmosphere using Schlenk technique to prevent exothermic reactions with air or moisture.

## General synthetic procedures

### General Procedure A for the preparation of tertiary amines SI-3, and SI-7, SI-10

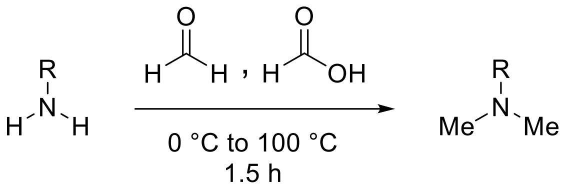


A magnetic stir bar and formic acid (3.0 mL, 80 mmol, 8 equiv.) were added to a round bottom flask immersed in an ice/water bath cooled to 0 °C and set stirring. Primary amine (10 mmol, 1 equiv.) was then added over the course of 10 minutes. Upon complete addition, the mixture was heated to 70 °C for 10 minutes, after which 37 % aqueous formaldehyde solution (3.3 mL, 33 mmol, 3.3 equiv.) was added. A reflux condenser was then fixed to the round bottom flask, and the reaction mixture was heated to 100 °C for 1.5 h. The reaction mixture was removed from heat and allowed to cool to RT, the aqueous solution was washed with $Et_2O$ three times. The aqueous layer was then basified with 1 M $NaOH_{(aq)}$ (pH > 10) and extracted three times with $Et_2O$. The organic layers were then combined, dried with $Na_2SO_4$, filtered, and concentrated by rotary evaporation to yield pure product.

### General Procedure B for the preparation of tertiary amine *N*-oxides SI-4, SI-6, SI-8, and SI-11 via $H_2O_2$

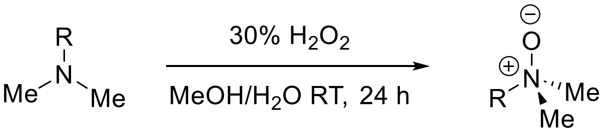


The corresponding tertiary amine was dissolved in methanol (2 M) in a round bottom flask containing a magnetic stir bar. The round bottom flask was then sealed with a rubber septum and vented with a needle. The mixture was then set to stir. Over the course of 10 minutes, 30% $H_2O_{2(aq)}$ (3 equiv.) was added via syringe through the septum. The reaction mixture was then allowed to stir at RT and monitored by TLC or $^1H$ NMR. After reaction completion, volatiles were removed by rotary evaporation at 40 °C for 30 min, the residue was further dried by Schlenk line vacuum for 8 h to yield pure product. **Attention**: It is critical for subsequent reactions that water is completely removed.

### General procedure C for the preparation of symmetric diazenes 2b-2o

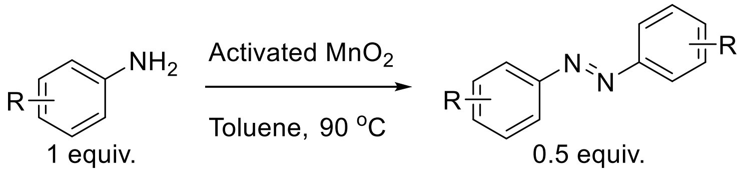


1.0 g of aniline was dissolved in 50 mL of toluene, to this solution 10 equiv. of activated $MnO_2$ was added. The mixture was then heated to 90 °C and monitored by TLC. The reaction was run until starting material was consumed by TLC (1-16 hours). Following the consumption of starting material, the reaction mixture was filtered through a plug of silica, and the silica plug was washed with hexanes. The solvent was removed by rotary evaporation and the resulting mixture was purified by FCC.

### General procedure D for the preparation of asymmetric diazenes 2s-2ab

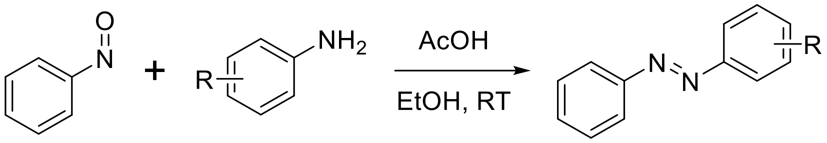


To a solution of nitrosobenzene in ethanol (5 mL) and glacial acetic acid (0.3 mL), the aniline (1.0 equiv.) was added. The reaction was stirred for 12 h. The reaction mixture was then diluted with ethanol and water followed by ethyl acetate extraction. This organic layer was dried over $Na_2SO_4$, and the ethyl acetate was gently removed under reduced pressure. Purification by FCC was carried out as needed.

### General procedure E for the preparation of Symmetric Triazolidines 3a-3r

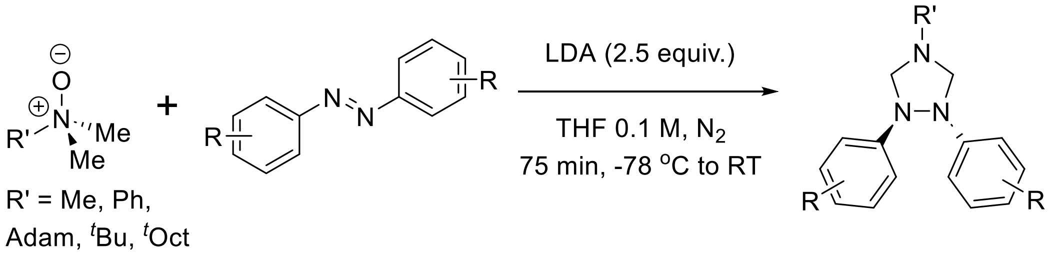


Dry tertiary amine *N*-oxide (0.5 mmol, 1.0 equiv.) was added to an oven dried test tube charged with a magnetic stir bar. The test tube was then sealed, purged, and flushed with dry nitrogen. 5.0 mL (0.1 M) of dry THF was then added to the test tube via a dry syringe. The reaction test tube was stirred at ambient temperature until *N*-oxide was completely dissolved. The reaction tube was then immersed in an acetone/dry ice bath and cooled to -78 °C. 1.6 M LDA (2.5 equiv.) in THF was then added dropwise over the course of 10 minutes via syringe. Immediately after LDA addition was completed, azobenzene (37.0 mg, 0.2 mmol, 0.5 equiv.) was dissolved in 1.0 mL (0.2 M) dry THF and added to the test tube dropwise over 1 minute. Following addition, the reaction flask was removed from the

cold bath and allowed to warm to ambient temperature and stirred for a total of 75 minutes, the reaction mixture was quenched with DI $H_2O$ and extracted three times with $Et_2O$ (10 mL). The organic layer was dried with $Na_2SO_4$, filtered, and concentrated by rotary evaporation. The product was then purified using FCC using a normal phase gradient on silica gel.

**General procedure F for the preparation of Asymmetric Triazolidines 3s-3ab**

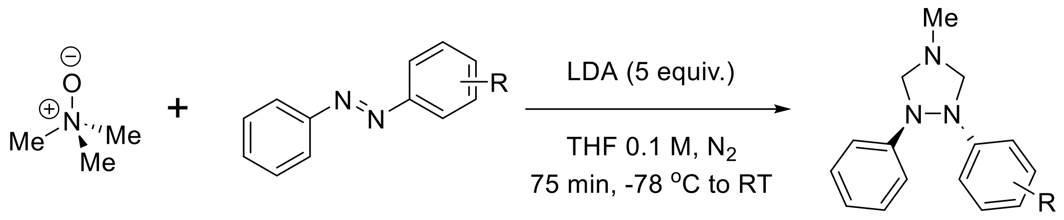


Dry triethylamine *N*-oxide (TMAO) (60.0 mg, 0.8 mmol, 1.0 equiv.) was added to an oven dried test tube charged with a magnetic stir bar. The test tube was then sealed, purged, and flushed with dry nitrogen. 6.0 mL (0.133 M) of THF was then added to the test tube via dry syringe and the mixture was set stirring. The reaction test tube was stirred at ambient temperature until TMAO was completely dissolved. It was then immersed in an acetone/dry ice bath and cooled to -78 °C. 1.6 M LDA in THF was then added dropwise over the course of 10 minutes via syringe. Immediately after LDA addition was completed, azobenzene (73.0 mg, 0.4 mmol, 0.5 equiv.) dissolved in 1.0 mL (0.1 M) dry THF was added to the test tube dropwise over circa 1 minute. Following complete addition, the reaction was removed from the cold bath and allowed to warm to ambient temperature and stirred for 75 minutes. At the end of the reaction time, the reaction mixture was concentrated by rotary evaporation. The crude residue was then purified using FCC using a normal phase gradient on silica gel.

## Compound Characterization

### (E)-4-(phenyldiazenyl)phenol (SI-1)

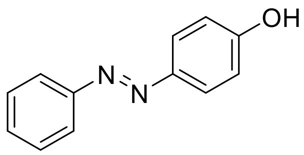

Prepared according to General Procedure D. A round bottom flask was charged with nitrosobenzene (390 mg, 3.64 mmol, 1.2 equiv.), EtOH (15 mL), and a magnetic stir bar. The mixture was stirred until the nitrosobenzene was completely solubilized; at which point, glacial acetic acid (0.9 mL) and 4-aminophenol (460 mg, 4.2 mmol, 1.4 equiv.) were added sequentially. The round bottom flask was then sealed with a rubber septum and vented. The reaction mixture then stirred for 24 h, after which it was quenched with 22.5 mL DI $H_2O$, extracted with $Et_2O$ and purified by FCC on silica gel (EtOAc/hexanes mobile phase) to isolate the product as an orange solid (70% yield, 510 mg, 2.458 mmol). $^1H$ NMR matched literature spectra.[2]

### 4-((*tert*-butyldimethylsilyl)oxy)aniline (SI-2)

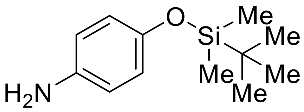

A round bottom flask was charged with 4-aminophenol (540 mg, 4.95 mmol, 1 equiv.), THF (10 mL), imidazole (1.0 g, 15 mmol, 3.0 equiv.), *tert*-butylchlorodimethylsilane (1.1 g, 7.5 mmol, 1.5 equiv.), and a stir bar. The round bottom flask was then sealed with a rubber septum and vented. The reaction mixture was then stirred for 4 h, after which it was quenched with saturated sodium bicarbonate solution. The phases were separated, and the aqueous phase was extracted three times with $Et_2O$, and purified by FCC on silica gel (EtOAc/hexanes mobile phase) to isolate the product (46 % yield, 510 mg, 2.3 mmol). $^1H$ NMR matched literature spectra.[3]

### ***N*,*N*,2,4,4-pentamethylpentan-2-amine (SI-3)**

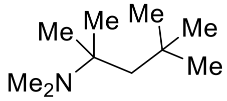

Prepared according to General Procedure A. A round bottom flask was charged with formic acid (15 mL, 400 mmol, 8 equiv.) and a stir bar. The flask was then cooled to 0 °C after which *tert*-octylamine (8.0 mL, 50 mmol, 1 equiv.) was added slowly over 10 min. The round bottom flask was then removed from the cold bath and heated to 70 °C for a total of 10 minutes, at which point a 37 % aqueous solution of formaldehyde (16.4 mL, 165 mmol, 3.3 equiv.) was added. A reflux condenser was then fixed to the round bottom flask and the reaction mixture was heated to 100 °C for 1.5 h, then it was removed from heat. After it cooled to room temperature, the crude reaction mixture was then washed with $Et_2O$. The aqueous layer from this extraction was basified with 1 M $NaOH_{(aq)}$ until it turned milky white. The aqueous layer was then extracted three times with $Et_2O$, dried with $Na_2SO4$, and filtered to afford pure product (80 % yield, 6.29 g, 40.0 mmol). $^1H$ NMR matched literature spectra.[4]

### ***N*,*N*,2,4,4-pentamethylpentan-2-amine oxide (SI-4)**

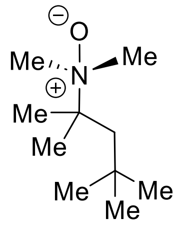

Prepared according to General Procedure B. *N*,*N*,2,4,4-pentamethylpentan-2-amine (4.4 g, 28.0 mmol, 1 equiv.) was dissolved in methanol (14.0 mL, 2 M) in a round bottom flask containing a magnetic stir bar. The round bottom flask was then sealed with a rubber septum and vented with a needle. The mixture was then set to stir. Over the course of 10 minutes, 30% $H_2O_2$ (6.6 mL, 84 mmol, 3 equiv.) was added via syringe through the septum. The reaction mixture was then allowed to stir at RT for 24 h. After that, the reaction mixture was concentrated by rotary evaporation at 40 °C for 30 min, then placed on Schlenk vacuum for 8 h to yield product (44 % yield, 2.13 g, 12.3 mmol). Yield includes small amounts of intractable Cope elimination product, 2,4,4-trimethylpentene, as indicated in $^{13}C$ NMR spectrum (Figure S2).

$^{1}H$ NMR (400 MHz, $CDCl_3$) δ 3.16 (s, 6H), 1.87 (s, 2H), 1.54 (s, 6H), 1.08 (s, 9H).

$^{13}C$ {$^{1}H$} NMR (101 MHz, $CDCl_3$) δ 52.0, 51.6, 31.7, 30.0, 25.3, 22.1.

IR: 2954, 2827, 2715, 1581, 1354, $cm^{-1}$

HRMS (ESI) m/z: $[M+H]^+$ Calc'd for $C_{10}H_{24}NO$ 174.1852; Found 174.1849

### ***N*,*N*,2-trimethylpropan-2-amine (SI-5)**

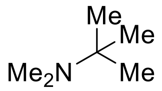

Product was prepared according to the literature.[5] A round bottom flask was charged with formic acid (4.52 mL, 120 mmol, 4 equiv.) and a stir bar. The flask was then cooled to 0 °C after which *tert*-butylamine (3.15 mL, 30 mmol, 1 equiv.) was added slowly over 10 min. The round bottom flask was then removed from the cold bath and heated to 90 °C, at which point a 37 % aqueous solution of formaldehyde (2.76 mL, 75 mmol, 2.5 equiv.) was added. The reaction mixture was allowed to reflux at 100 °C for 1.5 h, it was then removed from heat and let cool down to ambient temperature. Product was distilled as an azeotrope with water and taken to the oxidation step without further purification. (35 % yield, 1.06 g, 10.5 mmol). $^{1}H$ NMR matched literature spectra.[6]

***N*,*N*,2-trimethylpropan-2-amine oxide (SI-6)**

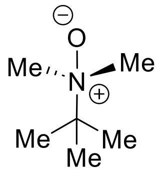

Prepared according to General Procedure B. *N*,*N*,2-trimethylpropan-2-amine (3.31 g, 32.7 mmol, 1.0 equiv.) was dissolved in methanol (16.35 mL, 2 M) in a round bottom flask containing a magnetic stir bar. The round bottom flask was then sealed with a rubber septum and vented with a needle. The mixture was then set to stir. Over the course of 10 minutes, 30% $H_2O_2$ (7.61 mL, 98.181 mmol, 3.0 equiv.) was added via syringe through the septum. The reaction mixture was then allowed to stir at RT for 24 h. After that, the reaction mixture was concentrated by rotary evaporation at 40 °C for 30 min, then placed on Schlenk vacuum for 8 h to yield pure product (35 % yield, 1.34 g, 11.4 mmol).

$^1$H NMR (400 MHz, $CDCl_3$) δ 3.11(s, 6H), 1.41 (s, 9H).

$^{13}$C {$^1$H} NMR (101 MHz, $CDCl_3$) δ 73.4, 52.6, 24.2.

IR: 2983, 2594, 1403, 1369, 1264 cm$^{-1}$

HRMS (ESI) m/z: $[M+H]^+$ Calc'd for $C_6H_{16}NO$ 118.1226; Found 118.1228

***N*,*N*-dimethyladamantan-1-amine (SI-7)**

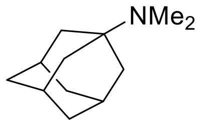


Prepared according to General Procedure A. A round bottom flask was charged with formic acid (6.03 mL, 160 mmol, 8 equiv.) and a stir bar The flask was then cooled to 0 °C after which adamantylamine (2.781 g, 20 mmol, 1equiv.) was added slowly over 10 min. The round bottom flask was then removed from the cold bath and heated to 90 °C, at which point a 37 % aqueous solution of formaldehyde (6.58 mL, 66 mmol, 3.3 equiv.) was added. The reaction mixture was allowed to reflux at 90 °C for 1.5 h then it was removed from heat. After it cooled to room temperature, the crude reaction mixture was then washed with $Et_2O$. The aqueous layer from this extraction was basified with 1 M $NaOH_{(aq)}$ until noticeable precipitate. The aqueous layer was then extracted three times with $Et_2O$, dried with $Na_2SO_4$, and filtered to afford pure product (62 % yield, 2.05 g, 11.43 mmol). $^1$H NMR matched literature spectra.[6]

**_N,N_-dimethyladamantan-1-amine oxide (SI-8)**

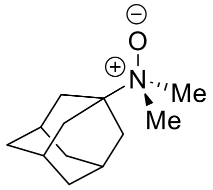

*N*,*N*-dimethyladamantan-1-amine (2.05 g, 11.43 mmol, 1 equiv.) was added to a round bottom flask and charged with a magnetic stir bar. The starng material was dissolved in dichloromethane (110 mL, 0.1M), and cooled to 0 °C in an ice water bath. After stirring at 0 °C for 20 minutes 70% mCPBA (2.81 g, 11.4 mmol, 1 equiv.) was added portionwise over 2 minutes. The round bottom flask was then sealed with a rubber septum, vented with a needle, and allowed to stir at 0 °C for 1 hour. After 1-hour $Na_2CO_3$ (3.62 g, 34.2 mmol, 3 equiv.) was added and the reaction was allowed to stir for an addition hour. The reaction mixture was then filtered using a Buchner funnel and the filtrate was concentrated by rotary evaporation at 23°C before being dried on Schlenk line vacuum. The product was sufficiently pure and was used without other purification (95% yield, 2.131 g, 10.91 mmol).

$^1$H NMR (500 MHz, $CDCl_3$) δ 3.17 (s, 1H), 2.28 (s, 3H), 2.10 (s, 6H), 1.76 – 1.57 (m, 6H).

$^{13}$C {$^1$H} NMR (126 MHz, $CDCl_3$) δ 73.1, 51.7, 35.7, 35.6, 30.0.

IR: 2906, 2849, 1361, 1287, 1054 cm$^{-1}$

HRMS (ESI) m/z: [M+H]$^+$ Calc'd for $C_{12}H_{22}NO$ 196.1696; Found 196.1713

**_N_-(cyclopropylmethyl)adamantan-1-amine (SI-9)**

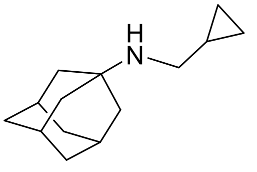

DCM (5 mL, 1 M), Adamantylamine (756 mg, 5.0 mmol, 1.0 equiv.), and cyclopropanecarbaldehyde (0.52 mL, 5 mmol, 1.0 equiv.), and $Na_2SO4$ (710 mg, 5 mmol, 1.0 equiv.) were added to a round bottom flask along with a magnetic stir bar. The reaction mixture was allowed to stir for 2 h at RT. After that, methanol (15 mL, 0.3 M) and $NaBH_4$ (189 mg, 5 mmol, 1.0 equiv.) were added sequentially and the mixture was stirred for an additional 1.5 h. The reaction mixture was then concentrated *in vacuo*, washed with deionized water, and extracted three times with $Et_2O$ to yield pure product that was taken on to the next step without further purification.

$^1$H NMR (400 MHz, $CDCl_3$) δ 2.44 (d, *J* = 6.9 Hz, 2H), 2.07 (s, 3H), 1.73 – 1.56 (m, 12H), 0.99 – 0.88 (m, 1H), 0.48 (q, *J* = 5.7 Hz, 2H), 0.09 (q, *J* = 4.6 Hz, 2H).

$^{13}$C {$^1$H} NMR (101 MHz, $CDCl_3$) δ 50.1, 45.9, 42.8, 36.8, 29.6, 12.1, 3.5.

IR: 2905, 2849, 1365, 1344, 1308 cm$^{-1}$

HRMS (ESI) m/z: [M+H]$^+$ Calc'd for $C_{14}H_{24}N$ 206.1903; Found 206.1917

**N-(cyclopropylmethyl)-N-methyladamantan-1-amine (SI-10)**

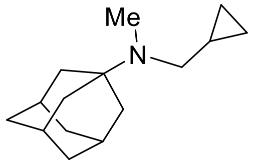

Prepared according to General Procedure A. A round bottom flask was charged with formic acid (1.58 mL, 41.84 mmol, 8 equiv.) and a stir bar. The flask was then cooled to 0 °C after which *N*-(cyclopropylmethyl)adamantan-1-amine (1.063 g, 5.23 mmol, 1 equiv.) was added slowly over 10 min. The round bottom flask was then removed from the cold bath and heated to 90 °C, at which point a 37 % aqueous solution of formaldehyde (1.718 mL, 17.25 mmol, 3.3 equiv.) was added. The reaction mixture was allowed to reflux at 90 °C for 1.5 h and was then removed from heat. After it cooled to room temperature, the crude reaction mixture was washed with $Et_2O$. The aqueous layer from this extraction was basified with 1 M $NaOH_{(aq)}$ until noticeable precipitate. The aqueous layer was then extracted three times with $Et_2O$, dried with $Na_2SO_4$, and filtered to afford pure product (72 % yield, over 2 steps, 770 mg, 3.49 mmol).

$^1$H NMR (400 MHz, $CDCl_3$) δ 2.45 – 2.22 (m, 5H), 2.09 (s, 3H), 1.77 – 1.51 (m, 12H), 0.93 – 0.79 (m, 1H), 0.53 (q, *J* = 5.5 Hz, 2H), 0.11 (q, *J* = 4.7 Hz, 2H).

$^{13}$C {$^1$H} NMR (126 MHz, $CDCl_3$) δ 54.2, 53.9, 38.6, 36.9, 33.9, 29.7, 11.0, 4.2.

IR: 2905, 2849, 1365, 1344, 1308 cm$^{-1}$

HRMS (ESI) m/z: $[M+H]^+$ Calc'd for $C_{15}H_{26}N$ 220.2060; Found 220.2065

**N-(cyclopropylmethyl)-N-methyladamantan-1-amine oxide (4)**

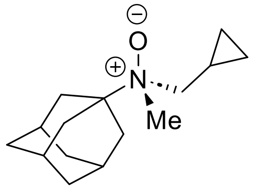

Prepared according to General Procedure B. *N*-(cyclopropylmethyl)-N-methyladamantan-1-amine (766.5 mg, 3.49 mmol, 1 equiv.) was added to a round bottom flask with a magnetic stir bar. Methanol (2 mL, 2 M), and 30% $H_2O_2$ (0.812 mL, 10.48 mmol, 3 equiv.) were added sequentially to the round bottom flask. The round bottom flask was then sealed with a rubber septum, vented with a needle, and allowed to stir for 5 h at RT. After that, the reaction mixture was concentrated *in vacuo* and the product was taken onto the next step without further purification. (73 % yield, 599.6 mg, 2.547 mmol)

$^1$H NMR (400 MHz, $CDCl_3$) δ 3.63 (dd, *J* = 11.9, 3.4 Hz, 1H), 3.09 (s, 3H), 2.51 (dd, *J* = 11.9, 9.4 Hz, 1H), 2.26 (s, 3H), 2.18 – 2.05 (m, 6H), 1.68 (q, *J* = 12.5 Hz, 7H), 0.85 – 0.77 (m, 1H), 0.76 – 0.68 (m, 1H), 0.36 – 0.28 (m, 1H), 0.28 – 0.20 (m, 1H).

$^{13}$C {$^1$H} NMR (126 MHz, $CDCl_3$) δ 74.0, 66.2, 47.0, 36.0, 35.6, 30.2, 7.7, 5.8, 2.2.

IR: 2905, 2849, 1365, 1344, 1308 cm$^{-1}$

HRMS (ESI) m/z: $[M+H]^+$ Calc'd for $C_{15}H_{26}NO$ 236.2009; Found 236.2015

**(E)-1,2-di-*o*-tolyldiazene (2b)**

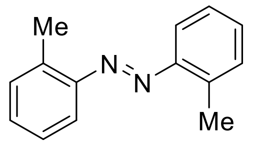


Prepared according to General Procedure C. A round bottom flask was charged with *o*-toluidine (1.0 mL, 9.33 mmol, 1 equiv.), toluene (25 mL), activated $MnO_2$ (4.15 g, 46.65 mmol, 5 equiv.), and a stir bar. The reaction mixture was then heated to 90 °C until starting material was consumed (monitored by TLC). The reaction mixture was then filtered through a silica plug using hexane as the rinsing solvent. The crude reaction mixture was then purified by FCC on silica gel (EtOAc/hexanes mobile phase) to isolate the product as a dark orange solid (62% yield, 618 mg, 2.93 mmol). $^1$H NMR matched literature spectra.[8]

**(E)-1,2-di-*m*-tolyldiazene (2c)**

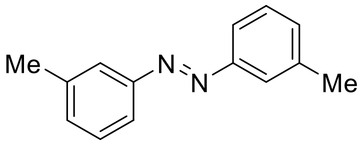


Prepared according to General Procedure C. A round bottom flask was charged with *m*-toluidine (1.0 mL, 9.33 mmol, 1 equiv.), toluene (50 mL), activated $MnO_2$ (8.21 g, 93.2 mmol, 10 equiv.), and a stir bar. The reaction mixture was then heated to 90 °C until starting material was consumed (monitored by TLC). The reaction mixture was then filtered through a silica plug using hexane as the rinsing solvent. The crude reaction mixture was then purified by FCC on silica gel (EtOAc/hexanes mobile phase) to isolate the product as a red solid (75% yield, 738 mg, 3.51 mmol). $^1$H NMR matched literature spectra.[9]

**(E)-1,2-di-*p*-tolyldiazene (2d)**

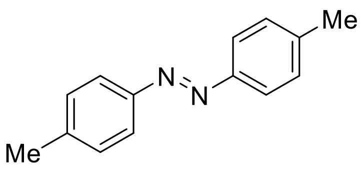


Prepared according to General Procedure C. A round bottom flask was charged with *p*-toluidine (1.0 mg, 9.33 mmol, 1 equiv.), toluene (25 mL), activated $MnO_2$ (3.43 g, 37.32 mmol, 4 equiv.), and a stir bar. The reaction mixture was then heated to 90 °C until starting material was consumed (monitored by TLC). The reaction mixture was then filtered through a silica plug using hexane as the rinsing solvent. The crude reaction mixture was then purified by FCC on silica gel (EtOAc/hexanes mobile phase) to isolate the product as a yellow solid (66% yield, 654 mg, 4.745 mmol). $^1$H NMR matched literature spectra.[8]

**(E)-1,2-bis(3,5-dimethylphenyl)diazene (2e)**

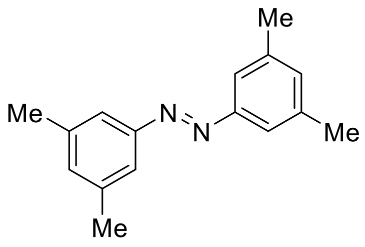


Prepared according to General Procedure C. A round bottom flask was charged with 3,5-dimethylaniline (1.0 mL, 8.25 mmol, 1 equiv.), toluene (50 mL), activated $MnO_2$ (7.19 g, 82.52 mmol, 10 equiv.), and a stir bar. The reaction mixture was then heated to 90 °C until starting material was consumed (monitored by TLC). The reaction mixture was then filtered through a silica plug using hexane as the rinsing solvent. The crude reaction mixture was then purified by FCC on silica gel (EtOAc/hexanes mobile phase) to isolate the product as a bright orange solid (57% yield, 541 mg, 2.269 mmol). $^1$H NMR matched literature spectra.[10]

**(E)-1,2-bis(4-methoxyphenyl)diazene (2f)**

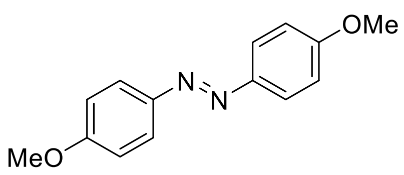


Prepared according to General Procedure C. A round bottom flask was charged with 4-methoxyaniline (1.0 g, 8.12 mmol, 1 equiv.), toluene (50 mL), activated $MnO_2$ (7.07 g, 81.2 mmol, 10 equiv.), and a stir bar. The reaction mixture was then heated to 90 °C until starting material was consumed (monitored by TLC). The reaction mixture was then filtered through a silica plug using hexane as the rinsing solvent. The crude reaction mixture was then purified by FCC on silica gel (EtOAc/hexanes mobile phase) to isolate the product as a yellow solid (63% yield, 647 mg, 2.67 mmol). $^1$H NMR matched literature spectra.[11]

**(E)-1,2-bis(4-phenoxyphenyl)diazene (2h)**

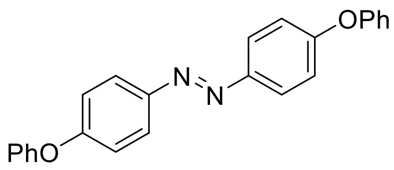


Prepared according to General procedure C. A round bottom flask was charged with 4-phenoxyaniline (1.0 g, 5.39 mmol, 1 equiv.), toluene (50 mL), activated $MnO_2$ (4.70 g, 93.2 mmol, 10 equiv.), and a stir bar. The reaction mixture was then heated to 90 °C until starting material was consumed (monitored by TLC). The reaction mixture was then filtered through a silica plug using hexane as the rinsing solvent. The crude reaction mixture was then purified by FCC on silica gel (EtOAc/hexanes mobile phase) to isolate the product as dark yellow solid (53% yield, 530 mg, 1.446 mmol). $^1$H NMR matched literature spectra.[12]

**(E)-1,2-bis(4-((*tert*-butyldimethylsilyl)oxy)phenyl)diazene (2i)**

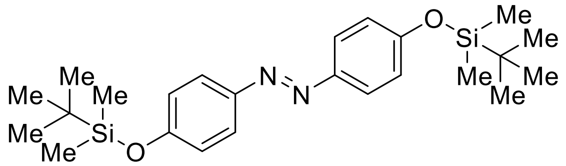


Prepared according to General procedure C. A round bottom flask was charged with 4-((*tert*-butyldimethylsilyl)oxy)aniline (155.1 mg, 0.693 mmol, 1 equiv.), toluene (1.5 mL), activated $MnO_2$ (0.3 g, 3.46 mmol, 5 equiv.), and a stir bar. The reaction mixture was then heated to 90 °C until starting material was consumed (monitored by TLC). The reaction mixture was then filtered through a silica plug using hexane as the rinsing solvent. The crude reaction mixture was then purified by FCC on silica gel (EtOAc/hexanes mobile phase) to isolate the product (78% yield, 119.8 mg, 0.271 mmol). $^1$H NMR matched literature spectra.[13]

**(E)-1,2-bis(4-(methylthio)phenyl)diazene (2j)**

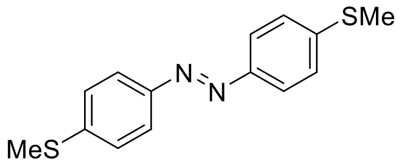


Prepared according to General procedure C. A round bottom flask was charged with 4-(methylthio)aniline (0.89 mL, 7.18 mmol, 1 equiv.), toluene (50 mL), activated $MnO_2$ (6.27 g, 71.8 mmol, 10 equiv.), and a stir bar. The reaction mixture was then heated to 90 °C until starting material was consumed by TLC. The reaction mixture was then filtered through silica gel, and the silica plug was washed with hexanes. The crude reaction mixture was then purified by FCC on silica gel (EtOAc/hexanes mobile phase) to isolate the product as a dark red solid (56% yield, 556 mg, 2.026 mmol). $^1$H NMR matched literature spectra.[14]

**(E)-1,2-bis(4-(trifluoromethyl)phenyl)diazene (2k)**

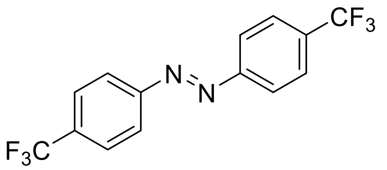


Prepared according to General procedure C. A round bottom flask was charged with 4-aminobenzotrifluoride (0.78 mL, 6.21 mmol, 1 equiv.), toluene (25 mL), activated $MnO_2$ (2.78 g, 31.05 mmol, 5 equiv.), and a stir bar. The reaction mixture was then heated to 90 °C until starting material was consumed (monitored by TLC). The reaction mixture was then filtered through a silica plug using hexane as the rinsing solvent. The crude reaction mixture was then purified by FCC on silica gel (EtOAc/hexanes mobile phase) to isolate the product as a bright orange solid (28% yield, 281 mg, 0.883 mmol). $^1$H NMR matched literature spectra.[14]

**(E)-1,2-bis(3-(trifluoromethyl)phenyl)diazene (2l)**

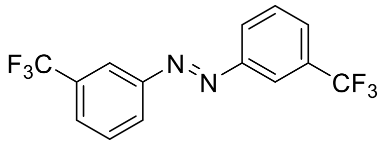


Prepared according to General procedure C. A round bottom flask was charged with 3-aminobenzotrifluoride (0.78 mL, 6.21 mmol, 1 equiv.), toluene (50 mL), activated $MnO_2$ (5.42 g, 62.1 mmol, 10 equiv.), and a stir bar. The reaction mixture was then heated to 90 °C until starting material was consumed (monitored by TLC). The reaction mixture was then filtered through a silica plug using hexane as the rinsing solvent. The crude reaction mixture was then purified by FCC on silica gel (EtOAc/hexanes mobile phase) to isolate the product as a bright orange solid (27% yield, 534 mg, 1.68 mmol). $^1$H NMR matched literature spectra.[15]

**(E)-1,2-bis(4-fluorophenyl)diazene (2m)**

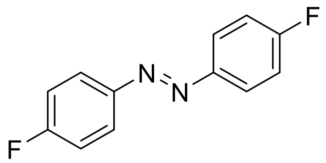


Prepared ccording to General procedure C. A round bottom flask was charged with 4-fluoroaniline (0.85 mL, 8.9 mmol, 1 equiv.), toluene (50 mL), activated $MnO_2$ (7.74 g, 89 mmol, 10 equiv.), and a stir bar. The reaction mixture was then heated to 90 °C until starting material was consumed (monitored by TLC). The reaction mixture was then filtered through a silica plug using hexane as the rinsing solvent. The crude reaction mixture was then purified by FCC on silica gel (EtOAc/hexanes mobile phase) to isolate the product as an orange solid (56% yield, 538 mg, 2.466 mmol). $^1$H NMR matched literature spectra.[14]

**(E)-1,2-bis(2-chlorophenyl)diazene (2n)**

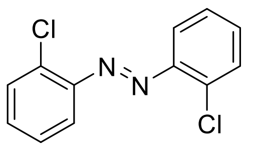


Prepared according to General procedure C. A round bottom flask was charged with 2-chloroaniline (0.84 mL, 7.83 mmol, 1 equiv.), toluene (25 mL), activated $MnO_2$ (3.84 g, 44.17 mmol, 5 equiv.), and a stir bar. The reaction mixture was then heated to 90 °C until starting material was consumed by TLC. The reaction mixture was then filtered through silica gel, and the silica plug was washed with hexanes. The crude reaction mixture was then purified by FCC on silica gel (EtOAc/hexanes mobile phase) to isolate the product as a red solid (36% yield, 363 mg, 1.446 mmol). $^1$H NMR matched literature spectra.[8]

**(E)-1,2-bis(3-fluoro-4-methoxyphenyl)diazene (2o)**

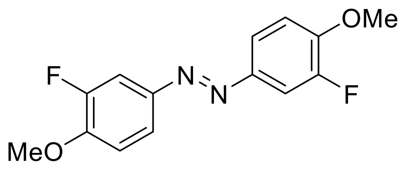

Prepared according to General procedure C. A round bottom flask was charged with 3-fluoro-4-methoxyaniline (1.0 g, 7.09 mmol, 1 equiv.), toluene (50 mL), activated $MnO_2$ (6.17 g, 70.89 mmol, 7.09 equiv.), and a stir bar. The reaction mixture was then heated to 90 °C until starting material was consumed (monitored by TLC). The reaction mixture was then filtered through a silica plug using hexane as the rinsing solvent. The crude reaction mixture was then purified by FCC on silica gel (EtOAc/hexanes mobile phase) to isolate the product as a yellow solid (72% yield, 714 mg, 2.566 mmol).

$^1$H NMR (400 MHz, $CDCl_3$) δ 7.74 (d, J = 8.7 Hz, 2H), 7.67 (dd, J = 12.2, 2.2 Hz, 2H), 7.08 (t, J = 8.7 Hz, 2H), 3.98 (s, 6H).

$^{13}$C {$^1$H} NMR (101 MHz, $CDCl_3$) δ 152.7 (d, J = 248.4 Hz), 150.12 (d, J = 11.5 Hz), 146.50 (d, J = 5.3 Hz), 122.87 (d, J = 3.0 Hz), 112.44 (d, J = 2.3 Hz), 107.49 (d, J = 19.4 Hz), 56.37.

$^{19}$F NMR (376 MHz, $CDCl_3$) δ -133.6.

IR: 1611, 1440, 1217, 1098, 734 cm$^{-1}$

HRMS (ESI) m/z: $[M+H]^+$ Calc'd for $C_{14}H_{13}F_2N_2O_2$ 279.0940; Found 279.0961

**(E)-1-phenyl-2-(*o*-tolyl)diazene (2s)**

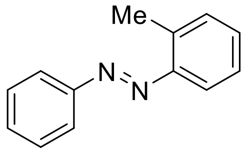

Prepared according to General Procedure D. A round bottom flask was charged with nitrosobenzene (385.8 mg, 3.6 mmol, 1.2 equiv.), EtOH (15 mL), and a magnetic stir bar. The mixture was stirred until the nitrosobenzene was completely solubilized; at which point, glacial acetic acid (0.9 mL) and *o*-toluidine (0.57 mL, 4.2 mmol, 1.4 equiv.) were added sequentially. The round bottom flask was then sealed with a rubber septum and vented. The reaction mixture then stirred for 24 h at RT, after which it was quenched with 22.5 mL DI $H_2O$, extracted with $Et_2O$ and purified by FCC on silica gel (EtOAc/hexanes mobile phase) to isolate the product as red oil (90% yield, 635.0 mg, 3.326 mmol). $^1$H NMR matched literature spectra.[16]

**(E)-1-phenyl-2-(*m*-tolyl)diazene (2t)**

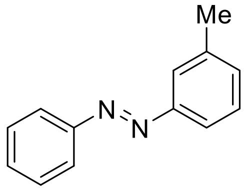


Prepared according to General Procedure D. A round bottom flask was charged with nitrosobenzene (385.7 mg, 3.6 mmol, 1.2 equiv.), EtOH (15 mL), and a magnetic stir bar. The mixture was stirred until the nitrosobenzene was completely solubilized; at which point, glacial acetic acid (0.3 mL) and *m*-toluidine (0.57 mL, 4.2 mmol, 1.4 equiv.) were added sequentially. The round bottom flask was then sealed with a rubber septum and vented. The reaction mixture then stirred for 24 h, after which it was quenched with 22.5 mL DI $H_2O$, extracted with $Et_2O$ and purified by FCC on silica gel (EtOAc/hexanes mobile phase) to isolate the product as red oil (94% yield, 669.0 mg, 3.409 mmol). $^1H$ NMR matched literature spectra.[16]

**(E)-1-phenyl-2-(*p*-tolyl)diazene (2u)**

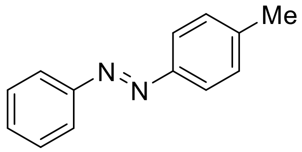


Prepared according to General Procedure D. A round bottom flask was charged with nitrosobenzene (128.5 mg, 1.2 mmol, 1.2 equiv.), EtOH (5 mL), and a magnetic stir bar. The mixture was stirred until the nitrosobenzene was completely solubilized; at which point, glacial acetic acid (0.3 mL) and *p*-toluidine (150.8 mg, 1.4 mmol, 1.4 equiv.) were added sequentially. The round bottom flask was then sealed with a rubber septum and vented. The reaction mixture then stirred for 24 h, after which it was quenched with 7.5 mL DI $H_2O$, extracted with $Et_2O$ and purified by FCC on silica gel (EtOAc/hexanes mobile phase) to isolate the product as an orange solid (99% yield, 234.0 mg, 1.192 mmol). $^1H$ NMR matched literature spectra.[16]

**(E)-1-(4-methoxyphenyl)-2-phenyldiazene (2v)**

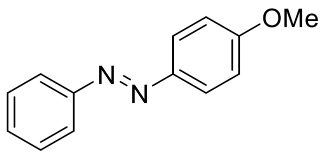


Prepared according to General Procedure D. A round bottom flask was charged with nitrosobenzene (129.5 mg, 1.2 mmol, 1.2 equiv.), EtOH (5 mL), and a magnetic stir bar. The mixture was stirred until the nitrosobenzene was completely solubilized; at which point, glacial acetic acid (0.3 mL) and 4-methoxyaniline (173.2 mg, 1.4 mmol, 1.4 equiv.) were added sequentially. The round bottom flask was then sealed with a rubber septum and vented. The reaction mixture then stirred for 24 h, after which it was quenched with 7.5 mL DI $H_2O$, extracted with $Et_2O$ and purified by FCC on silica gel (EtOAc/hexanes mobile phase) to isolate the product as a red solid (96% yield, 737.0 mg, 3.472 mmol). $^1H$ NMR matched literature spectra.[16]

**(E)-1-(4-phenoxyphenyl)-2-phenyldiazene (2w)**

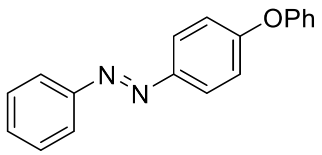

Prepared according to General Procedure D. A round bottom flask was charged with nitrosobenzene (385.7 mg, 3.6 mmol, 1.2 equiv.), EtOH (15 mL), and a magnetic stir bar. The mixture was stirred until the nitrosobenzene was completely solubilized; at which point, glacial acetic acid (0.9 mL) and 4-phenoxyaniline (778.2 mg, 4.2 mmol, 1.4 equiv.) were added sequentially. The round bottom flask was then sealed with a rubber septum and vented. The reaction mixture then stirred for 24 h, after which it was quenched with 22.5 mL DI $H_2O$, extracted with $Et_2O$ and purified by FCC on silica gel (EtOAc/hexanes mobile phase) to isolate the product as a red solid (90% yield, 845.0 mg, 3.080 mmol). $^1$H NMR matched literature spectra.[17]

**(E)-1-(4-((*tert*-butyldimethylsilyl)oxy)phenyl)-2-phenyldiazene (2x)**

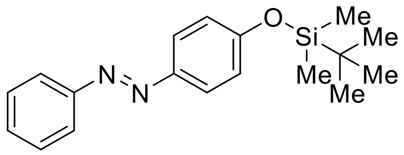

A round bottom flask was charged with (E)-4-(phenyldiazenyl)phenol (501 mg, 2.52 mmol, 1 equiv.), THF (15 mL), imidazole (450.6 mg, 7.5 mmol, 3.0 equiv.), *tert*-butylchlorodimethylsilane (565.2 mg, 3.75 mmol, 1.5 equiv.), and a stir bar. The round bottom flask was then sealed with a rubber septum and vented. The reaction mixture was then stirred for 3 h, after which it was quenched with excess sodium bicarbonate, extracted three times with $Et_2O$, and purified by FCC on silica gel (EtOAc/hexanes mobile phase) to isolate the product as dark red oil (79 % yield, 612 mg, 1.958 mmol). $^1$H NMR matched literature spectra.[18]

**(E)-1-(4-(methylthio)phenyl)-2-phenyldiazene (2y)**

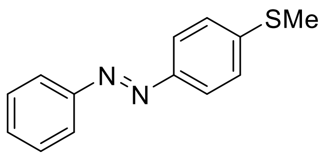

Prepared according to General Procedure D. A round bottom flask was charged with nitrosobenzene (358.8 mg, 3.6 mmol, 1.2 equiv.), EtOH (15 mL), and a magnetic stir bar. The mixture was stirred until the nitrosobenzene was completely solubilized; at which point, glacial acetic acid (0.9 mL) and 4-(methylthio)aniline (0.53 mL, 4.2 mmol, 1.4 equiv.) were added sequentially. The round bottom flask was then sealed with a rubber septum and vented. The reaction mixture then stirred for 24 h, after which it was quenched with 22.5 mL DI $H_2O$, extracted with $Et_2O$ and purified by FCC on silica gel (EtOAc/hexanes mobile phase) to isolate the product as a yellow oil (87% yield, 713 mg, 3.123 mmol). $^1$H NMR matched literature spectra.[11]

**(E)-1-phenyl-2-(4-(trifluoromethyl)phenyl)diazene (2z)**

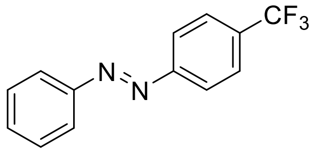


Prepared according to General Procedure D. A round bottom flask was charged with nitrosobenzene (535.9 mg, 5.0 mmol, 1.2 equiv.), EtOH (25 mL), and a magnetic stir bar. The mixture was stirred until the nitrosobenzene was completely solubilized; at which point, glacial acetic acid (2.0 mL) and 4-(trifluoromethyl)aniline (0.88 mL, 7.0 mmol, 1.4 equiv.) were added sequentially. The round bottom flask was then sealed with a rubber septum and vented. The reaction mixture then stirred for 24 h, after which it was quenched with 37.5 mL DI $H_2O$, extracted with $Et_2O$ and purified by FCC on silica gel (EtOAc/hexanes mobile phase) to isolate the product as a yellow solid (49% yield, 548 mg, 2.190 mmol). $^1H$ NMR matched literature spectra.[11]

**(E)-1-(4-fluorophenyl)-2-phenyldiazene (2aa)**

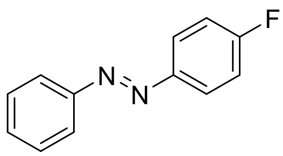


Prepared according to General Procedure D. A round bottom flask was charged with nitrosobenzene (385.8 mg, 3.6 mmol, 1.2 equiv.), EtOH (15 mL), and a magnetic stir bar. The mixture was stirred until the nitrosobenzene was completely solubilized; at which point, glacial acetic acid (0.9 mL) and 4-fluoroaniline (0.4 mL, 4.2 mmol, 1.4 equiv.) were added sequentially. The round bottom flask was then sealed with a rubber septum and vented. The reaction mixture then stirred for 24 h, after which it was quenched with 22.5 mL DI $H_2O$, extracted with $Et_2O$ and purified by FCC on silica gel (EtOAc/hexanes mobile phase) to isolate the product as a yellow solid (94% yield, 677.0 mg, 3.381 mmol). $^1H$ NMR matched literature spectra.[11]

**(E)-4-methyl-7-(phenyldiazenyl)-2H-chromen-2-one (2ab)**

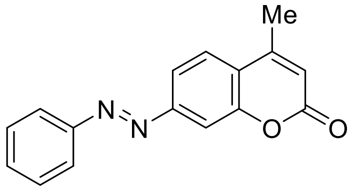

Prepared according to General Procedure D. A round bottom flask was charged with nitrosobenzene (128.5 mg, 1.2 mmol, 1.2 equiv.), EtOH (5 mL), and a magnetic stir bar. The mixture was stirred until the nitrosobenzene was completely solubilized; at which point, glacial acetic acid (0.3 mL) and 7-amino-4-methyl-2H-chromen-2-one (245.3 mg, 1.4 mmol, 1.4 equiv.) were added sequentially. The round bottom flask was then sealed with a rubber septum and vented. The reaction mixture was heated to 85 °C and stirred for 24 h, after which it was quenched with 7.5 mL DI $H_2O$, extracted with $Et_2O$ and purified by FCC on silica gel (EtOAc/hexanes mobile phase) to isolate the product as a dark orange solid (5% yield, 15.0 mg, 0.086 mmol)

$^1$H NMR (400 MHz, $CDCl_3$) δ 7.95 (dd, *J* = 7.6, 2.1 Hz, 2H), 7.89 – 7.81 (m, 2H), 7.72 (d, *J* = 8.3 Hz, 1H), 7.59 – 7.46 (m, 3H), 6.35 (d, *J* = 1.3 Hz, 1H), 2.48 (s, 3H).

$^{13}$C {$^1$H} NMR (101 MHz, $CDCl_3$) δ 160.0, 153.6, 153.4, 151.7, 151.2, 131.3, 128.6, 124.6, 122.6, 121.0, 118.1, 115.3, 110.5, 18.2.

IR: 3023, 2979, 1712, 1591, 1242 cm$^{-1}$

HRMS (ESI) m/z: $[M+H]^+$ Calc'd for $C_{16}H_{13}N_2O_2$ 265.0972; Found 265.0981

**4-methyl-1,2-diphenyl-1,2,4-triazolidine (3a)**

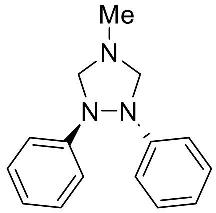

Triazolidine was prepared according to the general procedure E using trimethylamine *N*-oxide (35.5 mg, 0.5 mmol, 1.0 equiv.), azobenzene (45.5 mg, 0.25 mmol, 0.5 equiv.), 1.6 M LDA (0.39 mL, 0.625 mmol, 2.5 equiv.), dry THF (5.0 mL, 0.1 M). This was followed by purification by FCC on silica (EtOAc/hexanes mobile phase) to isolate product as a yellow oil (94% yield, 56 mg, 0.235 mmol).

$^1$H NMR (400 MHz, $CDCl_3$) δ 7.28 – 7.21 (m, 4H), 6.96 (d, *J* = 7.8 Hz, 4H), 6.89 (t, *J* = 7.3 Hz, 2H), 4.45 – 4.08 (m, 4H), 2.38 (s, 3H).

$^{13}$C {$^1$H} NMR (126 MHz, $CDCl_3$) δ 151.5, 129.1, 120.5, 114.4, 75.3, 42.5.

IR: 3023, 2951, 1591, 1282, 942 cm$^{-1}$

HRMS (ESI) m/z: $[M+H]^+$ Calc'd for $C_{15}H_{18}N_3$ 240.1495; Found 240.1508

**4-methyl-1,2-di-*o*-tolyl-1,2,4-triazolidine (3b)**

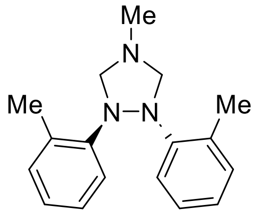


Triazolidine was prepared according to the general procedure E using trimethylamine *N*-oxide (35.6 mg, 0.5 mmol, 1.0 equiv.), (E)-1,2-di-*o*-tolyl diazene (52.6 mg, 0.25 mmol, 0.5 equiv.), 1.6 M LDA (0.39 mL, 0.625 mmol, 2.5 equiv.), and dry THF (5.0 mL, 0.1 M). This was followed by purification by FCC on silica (EtOAc/hexanes mobile phase) to isolate product as a yellow oil (63% yield, 42 mg, 0.158 mmol). Yield includes minor amount of intractable hydrazine side product as indicated in $^{1}$H NMR spectrum (Figure S20).

$^{1}$H NMR (400 MHz, $CDCl_3$) δ 7.61 (d, *J* = 8.1 Hz, 1H), 7.17 – 7.01 (m, 4H), 6.90 (t, *J* = 7.3 Hz, 2H), 4.39 (s, 2H), 3.90 (s, 2H), 2.47 (s, 3H), 2.41 (s, 6H).

$^{13}$C {$^{1}$H} NMR (126 MHz, $CDCl_3$) δ 150.9, 131.1, 128.2, 126.5, 122.6, 117.9, 77.7, 41.8, 19.4.

IR: 2949, 1501, 1238, 941, 759 $cm^{-1}$

HRMS (ESI) m/z: $[M+H_3O]^+$ Calc'd for $C_{17}H_{24}N_3O$ 286.1914; Found 286.1920

**4-methyl-1,2-di-*m*-tolyl-1,2,4-triazolidine (3c)**

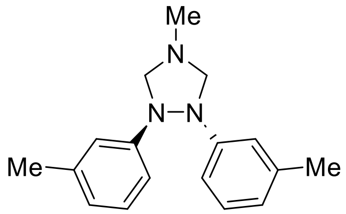


Triazolidine was prepared according to the general procedure E using trimethylamine *N*-oxide (35.5 mg, 0.5 mmol, 1.0 equiv.), (E)-1,2-di-*m*-tolyl diazene (52.6 mg, 0.25 mmol, 0.5 equiv.), 1.6 M LDA (0.39 mL, 0.625 mmol, 2.5 equiv.), and dry THF (5.0 mL, 0.1 M). This was followed by purification by FCC on silica gel (EtOAc/hexanes mobile phase) to isolate product as a yellow oil (93% yield, 62.2 mg, 0.233 mmol).

$^{1}$H NMR (400 MHz, $CDCl_3$) δ 7.13 (t, *J* = 7.7 Hz, 2H), 6.82 – 6.75 (m, 4H), 6.72 (d, *J* = 7.4 Hz, 2H), 4.32 (d, *J* = 8.2 Hz, 2H), 4.23 (d, *J* = 7.8 Hz, 2H), 2.39 (s, 3H), 2.30 (s, 6H).

$^{13}$C {$^{1}$H} NMR (126 MHz, $CDCl_3$) δ 151.7, 138.9, 129.0, 121.4, 115.0, 111.7, 75.4, 42.4, 21.8.

IR: 2950, 1584, 1302, 995, 910 $cm^{-1}$

HRMS (ESI) m/z: $[M+H]^+$ Calc'd for $C_{17}H_{22}N_3$ 268.1808; Found 268.1823

**4-methyl-1,2-di-*p*-tolyl-1,2,4-triazolidine (3d)**

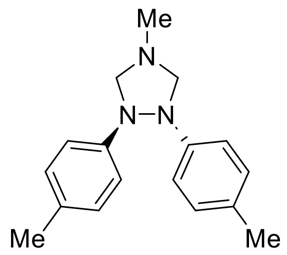


Triazolidine was prepared according to the general procedure E using trimethylamine *N*-oxide (35.6 mg, 0.5 mmol, 1.0 equiv.), (E)-1,2-di-*p*-tolyl diazene (52.6 mg, 0.25 mmol, 0.5 equiv.), 1.6 M LDA (0.39 mL, 0.625 mmol, 2.5 equiv.), and dry THF (5.0 mL, 0.1 M). This was followed by purification by FCC on silica gel (EtOAc/hexanes mobile phase) to isolate product as a yellow oil (61% yield, 40.8 mg, 0.153 mmol).

$^{1}$H NMR (400 MHz, $CDCl_3$) δ 7.05 (d, *J* = 8.1 Hz, 4H), 6.87 (d, *J* = 8.5 Hz, 4H), 4.26 (d, *J* = 13.2 Hz, 4H), 2.38 (s, 3H), 2.27 (s, 6H).

$^{13}$C {$^{1}$H} NMR (126 MHz, $CDCl_3$) δ 149.4, 129.7, 129.6, 114.4, 75.5, 42.5, 20.5.

IR: 2920, 1452, 1290, 943, 908 $cm^{-1}$

HRMS (ESI) m/z: $[M+H]^+$ Calc'd for $C_{17}H_{22}N_3$ 268.1808; Found 268.1834

**1,2-bis(3,5-dimethylphenyl)-4-methyl-1,2,4-triazolidine (3e)**

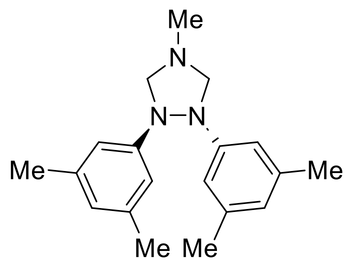


Triazolidine was prepared according to the general procedure E using trimethylamine *N*-oxide (35.6 mg, 0.5 mmol, 1.0 equiv.), (E)-1,2-bis(3,5-dimethylphenyl) diazene (59.6 mg, 0.25 mmol, 0.5 equiv.), 1.6 M LDA (0.39 mL, 0.625 mmol, 2.5 equiv.), and dry THF (5.0 mL, 0.1 M). This was followed by purification by FCC on silica gel (EtOAc/hexanes mobile phase) to isolate product as a yellow oil (91% yield, 67.2 mg, 0.228 mmol).

$^{1}$H NMR (400 MHz, $CDCl_3$) δ 6.61 (s, 4H), 6.55 (s, 2H), 4.28 (d, *J* = 8.1 Hz, 2H), 4.18 (d, *J* = 7.7 Hz, 2H), 2.38 (s, 3H), 2.26 (s, 12H).

$^{13}$C {$^{1}$H} NMR (126 MHz, $CDCl_3$) δ 151.9, 138.8, 122.5, 112.3, 75.4, 42.3, 21.7.

IR: 1916, 1593, 1312, 944, 909 $cm^{-1}$

HRMS (ESI) m/z: $[M+H]^+$ Calc'd for $C_{19}H_{26}N_3$ 296.2121; Found 296.2134

**1,2-bis(4-methoxyphenyl)-4-methyl-1,2,4-triazolidine (3f)**

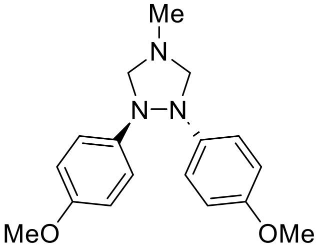

Triazolidine was prepared according to the general procedure E using trimethylamine *N*-oxide (35.6 mg, 0.5 mmol, 1.0 equiv.), (E)-1,2-bis(4-methoxyphenyl) diazene (60.6 mg, 0.25 mmol, 0.5 equiv.), 1.6 M LDA (0.39 mL, 0.625 mmol, 2.5 equiv.), and dry THF (5.0 mL, 0.1 M). This was followed by purification by FCC on silica gel (EtOAc/Hexanes mobile phase) to isolate product as a yellow oil (98% yield, 73.0 mg, 0.245 mmol).

$^1$H NMR (400 MHz, $CDCl_3$) δ 6.95 – 6.90 (m, 4H), 6.84 – 6.78 (m, 4H), 4.22 (s, 3H), 3.75 (s, 6H), 2.39 (s, 3H).

$^{13}$C {$^1$H} NMR (126 MHz, $CDCl_3$) δ 154.1, 145.7, 115.6, 114.5, 76.0, 55.7, 42.5.

IR: 2844, 1501, 1288, 1237, 823, 641 cm$^{-1}$

HRMS (ESI) m/z: $[M+H_3O]^+$ Calc'd for $C_{17}H_{24}N_3O_3$ 318.1812; Found 318.1820

**1,2-bis(4-methoxyphenyl)-4-phenyl-1,2,4-triazolidine (3g)**

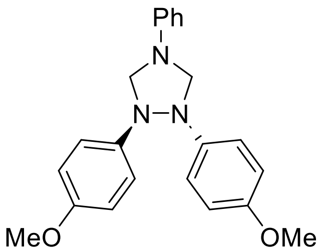

Triazolidine was prepared according to the general procedure E using *N*,*N*-dimethylaniline *N*-oxide (137.9 mg, 1.0 mmol, 1.0 equiv.), (E)-1,2-bis(4-methoxyphenyl) diazene (122.6 mg, 0.5 mmol, 0.5 equiv.), 1.6 M LDA (0.86 mL, 1.25 mmol, 5.0 equiv.), and dry THF (5.0 mL, 0.1 M). This was followed by purification by FCC on silica (EtOAc/hexanes mobile phase) to isolate product as an orange solid (29% yield, 54.0 mg, 0.15 mmol)

$^1$H NMR (500 MHz, $CDCl_3$) δ 7.21 (t, *J* = 7.8 Hz, 2H), 7.13 (d, *J* = 9.0 Hz, 4H), 6.83 (d, *J* = 9.0 Hz, 4H), 6.75 (t, *J* = 7.3 Hz, 1H), 6.56 (d, *J* = 8.5 Hz, 2H), 4.74 (s, 2H), 4.66 (s, 2H), 3.75 (s, 6H).

$^{13}$C {$^1$H} NMR (126 MHz, $CDCl_3$) δ 154.9, 145.2, 144.2, 129.4, 118.1, 116.8, 114.5, 113.2, 67.6, 55.7.

IR: 2922, 2850, 1499, 1287, 997 cm$^{-1}$

HRMS (ESI) m/z: $[M+Na+H_2O]^+$ Calc'd for $C_{22}H_{25}N_3NaO_3$ 402.1788; Found 402.1795

**4-methyl-1,2-bis(4-phenoxyphenyl)-1,2,4-triazolidine (3h)**

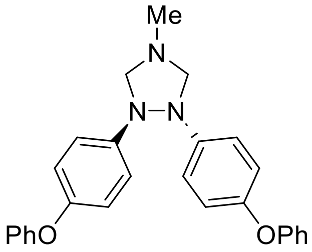


Triazolidine was prepared according to the general procedure E using trimethylamine *N*-oxide (35.6 mg, 0.5 mmol, 1.0 equiv.), (E)-1,2-bis(4-phenoxyphenyl) diazene (91.6 mg, 0.25 mmol, 0.5 equiv.), 1.6 M LDA (0.39 mL, 0.625 mmol, 2.5 equiv.), and dry THF (5.0 mL, 0.1 M). This was followed by purification by FCC on silica gel (EtOAc/hexanes mobile phase) to isolate product as a yellow oil (75% yield, 79.6 mg, 0.188 mmol).

$^1$H NMR (400 MHz, $CDCl_3$) δ 7.32 – 7.25 (m, 4H), 7.05 – 7.00 (m, 2H), 6.99 – 6.93 (m, 12H), 4.27 (s, 4H), 2.43 (s, 3H).

$^{13}$C {$^1$H} NMR (126 MHz, $CDCl_3$) δ 158.5, 150.5, 147.8, 129.6, 122.4, 120.5, 117.7, 115.7, 75.9, 42.1.

IR: 3036, 2848, 1586, 1279, 1231, 955 $cm^{-1}$

HRMS (ESI) m/z: $[M+H]^+$ Calc'd for $C_{27}H_{26}N_3O_2$ 424.2020; Found 424.1994

**1,2-bis(4-((*tert*-butyldimethylsilyl)oxy)phenyl)-4-methyl-1,2,4-triazolidine (3i)**

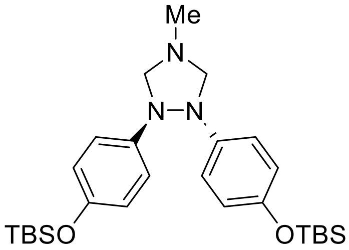


Triazolidine was prepared according to the general procedure E using trimethylamine *N*-oxide (21.0 mg, 0.28 mmol, 1.0 equiv.), (E)-1,2-bis(4-((tert-butyldimethylsilyl)oxy)-phenyl)diazene (138.0 mg, 0.14 mmol, 0.5 equiv.), 1.5 M LDA (0.22 mL, 0.35 mmol, 2.5 equiv.), and dry THF (2.5 mL, 0.1 M). This was followed by purification by FCC on silica (EtOAc/hexanes mobile phase) to isolate product as a yellow oil (93% yield, 63.8 mg, 0.128 mmol).

**Gram-scale synthesis:** Triazolidine was prepared according to the general procedure E using trimethylamine *N*-oxide (340.7 mg, 4.52 mmol, 1.0 equiv.), (E)-1,2-bis(4-((*tert*-butyldimethylsilyl)oxy)-phenyl)diazene (1.10 g, 2.26 mmol, 0.5 equiv.), 1.47 M LDA (7.69 mL, 11.3 mmol, 2.5 equiv.), and dry THF (22.6 mL, 0.2 M). This was followed by purification by FCC on silica gel (EtOAc in Hexanes mobile phase) to isolate product as a yellow oil (75% yield, 851.8 mg, 1.704 mmol).

$^1$H NMR (400 MHz, $CDCl_3$) δ 6.87 – 6.81 (m, 4H), 6.74 – 6.69 (m, 4H), 4.21 (s, 4H), 2.38 (s, 3H), 0.96 (s, 18H), 0.16 (s, 12H)

$^{13}$C {$^1$H} NMR (126 MHz, $CDCl_3$) δ149.6, 146.1, 120.3, 115.5, 76.0, 42.2, 25.7, 18.2, -4.4.

IR: 3400, 2857, 1596, 1246, 1109, 906 $cm^{-1}$

HRMS (ESI) m/z: $[M+H_3O]^+$ Calc'd for $C_{27}H_{48}N_3O_3Si_2$ 518.3229; Found 518.3254

**4-methyl-1,2-bis(4-(methylthio)phenyl)-1,2,4-triazolidine (3j)**

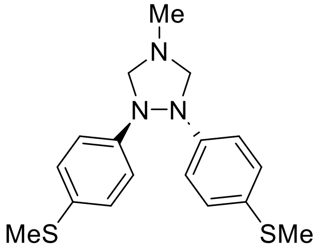


Triazolidine was prepared according to the general procedure E using trimethylamine *N*-oxide (35.6 mg, 0.5 mmol, 1.0 equiv.), (E)-1,2-bis(4-(methylthio)phenyl)diazene (68.6 mg, 0.25 mmol, 0.5 equiv.), 1.6 M LDA (0.39 mL, 0.625 mmol, 2.5 equiv.), and dry THF (5.0 mL, 0.1 M). This was followed by purification by FCC on silica (EtOAc/hexanes mobile phase) to isolate product as a yellow oil (52% yield, 43.1 mg, 0.130 mmol).

$^{1}$H NMR (400 MHz, $CDCl_3$) δ 7.25 – 7.18 (m, 4H), 6.94 – 6.86 (m, 4H), 4.28 (s, 2H), 4.26 (s, 2H), 2.43 (s, 6H), 2.39 (s, 3H).

$^{13}$C {$^{1}$H} NMR (126 MHz, $CDCl_3$) δ 149.8, 129.9, 128.6, 115.1, 75.4, 42.5, 18.0.

IR: 3079, 3024, 2917, 1589, 1290, 814, 725 cm$^{-1}$

HRMS (ESI) m/z: $[M+H]^+$ Calc'd for $C_{17}H_{22}N_3S_2$ 332.1250; Found 332.1277

**4-methyl-1,2-bis(4-(trifluoromethyl)phenyl)-1,2,4-triazolidine (3k)**

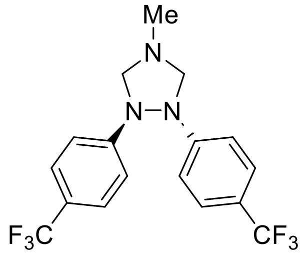


Triazolidine was prepared according to the general procedure E using trimethylamine *N*-oxide (35.6 mg, 0.5 mmol, 1.0 equiv.), (E)-1,2-bis(4-(trifluoromethyl)phenyl)diazene (79.6 mg, 0.25 mmol, 0.5 equiv.), 1.6 M LDA (0.39 mL, 0.625 mmol, 2.5 equiv.), dry THF (5.0 mL, 0.1 M). This was followed by purification by FCC on silica gel (EtOAc/hexanes mobile phase) to isolate product as a yellow oil (42% yield, 33.4 mg, 0.089 mmol).

$^{1}$H NMR (400 MHz, $CDCl_3$) δ 7.51 (d, *J* = 8.5 Hz, 4H), 6.97 (d, *J* = 8.5 Hz, 4H), 4.40 (d, *J* = 8.2 Hz, 2H), 4.32 (d, *J* = 8.3 Hz, 2H), 2.39 (s, 3H).

$^{13}$C {$^{1}$H} NMR (126 MHz, $CDCl_3$) δ 153.4, 126.6 (q, *J* = 3.7 Hz), 124.5 (q, J = 268.8 Hz), 122.9 (q, J = 32.7 Hz) 114.1, 75.2, 42.6.

$^{19}$F NMR (376 MHz, $CDCl_3$) δ -61.5

IR: 3161, 2929, 1515, 1320, 1065, 1007 cm$^{-1}$

HRMS (ESI) m/z: $[M+H]^+$ Calc'd for $C_{17}H_{16}F_6N_3$ 376.1243; Found 376.1222

**4-methyl-1,2-bis(3-(trifluoromethyl)phenyl)-1,2,4-triazolidine (3l)**

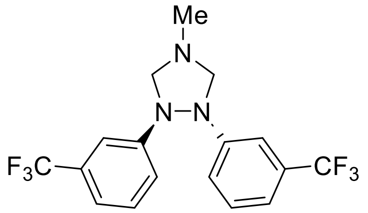


Triazolidine was prepared according to the general procedure E using trimethylamine *N*-oxide (35.6 mg, 0.5 mmol, 1.0 equiv.), (E)-1,2-bis(3-(trifluoromethyl)phenyl)-diazene (79.6 mg, 0.25 mmol, 0.5 equiv.), 1.6 M LDA (0.39 mL, 0.625 mmol, 2.5 equiv.), and dry THF (5.0 mL, 0.1 M). This was followed by purification by FCC on silica gel (EtOAc/hexanes mobile phase) to isolate product as a yellow oil (23% yield, 21.8 mg, 0.058 mmol).

$^1$H NMR (400 MHz, $CDCl_3$) δ 7.37 (t, *J* = 8.2 Hz, 2H), 7.18 (dd, *J* = 4.2, 1.8 Hz, 4H), 7.13 (d, *J* = 1.9 Hz, 1H), 7.11 (d, *J* = 2.1 Hz, 1H), 4.37 – 4.29 (m, 4H), 2.39 (s, 3H).

$^{13}$C {$^1$H} NMR (126 MHz, $CDCl_3$) δ 151.5, 131.6 (q, J = 31.2 Hz), 129.8, 124.2 (q, J = 272.2 Hz), 117.8 (q, J = 1.3 Hz), 117.7 (q, J = 3.9 Hz), 111.1 (q, J = 4.1 Hz), 75.7, 42.3.

$^{19}$F NMR (376 MHz, $CDCl_3$) δ -62.6.

IR: 3159, 2970, 1541, 1324, 1067, 995 $cm^{-1}$

HRMS (ESI) m/z: $[M+Na+H_2O]^+$ Calc'd for $C_{17}H_{17}F_6N_3NaO$ 416.1168; Found 416.1156

**1,2-bis(4-fluorophenyl)-4-methyl-1,2,4-triazolidine (3m)**

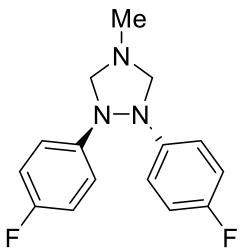


Triazolidine was prepared according to the general procedure E using trimethylamine *N*-oxide (35.6 mg, 0.5 mmol, 1.0 equiv.), (E)-1,2-bis(4-fluorophenyl) diazene (54.5 mg, 0.25 mmol, 0.5 equiv.), 1.6 M LDA (0.39 mL, 0.625 mmol, 2.5 equiv.), dry THF (5.0 mL, 0.1 M). This was followed by purification by FCC on silica gel (EtOAc/hexanes mobile phase) to isolate product as a yellow oil (69% yield, 47.5 mg, 0.173 mmol).

$^1$H NMR (500 MHz, $CDCl_3$) δ 6.98 – 6.87 (m, 8H), 4.24 (s, 4H), 2.39 (s, 3H).

$^{13}$C {$^1$H} NMR (126 MHz, $CDCl_3$) δ 157.7 (d, J = 238.5 Hz), 147.7 (d, *J* = 2.3 Hz), 115.7 (d, J = 10.7 Hz), 115.5 (d, J = 4.1 Hz), 76.0, 42.1.

$^{19}$F NMR (376 MHz, $CDCl_3$) δ -124.4.

IR: 2953, 1593, 1291, 1008, 826 $cm^{-1}$

HRMS (ESI) m/z: $[M+H_3O]^+$ Calc'd for $C_{15}H_{18}F_2N_3O$ 294.1412; Found 294.1390

**1,2-bis(2-chlorophenyl)-4-methyl-1,2,4-triazolidine (3n)**

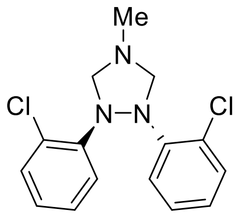


Triazolidine was prepared according to the general procedure E using trimethylamine *N*-oxide (37.9 mg, 0.5 mmol, 1.0 equiv.), (E)-1,2-bis(2-chlorophenyl)diazene (62.8 mg, 0.25 mmol, 0.5 equiv.), 1.6 M LDA (0.38 mL, 0.625 mmol, 2.5 equiv.), and dry THF (5.0 mL, 0.1 M). This was followed by purification by FCC on silica gel ((EtOAc/hexanes mobile phase) to isolate product as an orange oil (21% yield, 16.0 mg, 0.05 mmol).

$^{1}$H NMR (500 MHz, $CDCl_3$) δ 7.69 (d, *J* = 7.5 Hz, 2H), 7.33 (d, *J* = 7.9 Hz, 2H), 7.15 (t, *J* = 8.1 Hz, 2H), 6.91 (t, *J* = 8.0 Hz, 2H), 4.65 (d, *J* = 8.3 Hz, 2H), 4.14 (d, *J* = 8.1 Hz, 2H), 2.49 (s, 3H).

$^{13}$C {$^{1}$H} NMR (126 MHz, $CDCl_3$) δ 148.5, 130.3, 127.7, 124.2, 123.6, 119.5, 77.8, 42.6

IR: 3064, 2953, 1585, 1247, 937, 638 cm$^{-1}$

HRMS (ESI) m/z: [M+H+MeCN]$^+$ Calc'd for $C_{17}H_{19}Cl_2N_4$ 349.0981; Found 349.0975

**1,2-bis(3-fluoro-4-methoxyphenyl)-4-methyl-1,2,4-triazolidine (3o)**

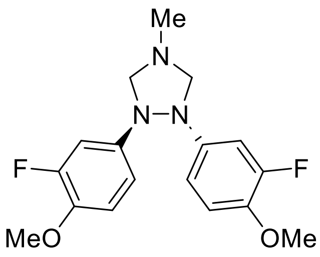


Triazolidine was prepared according to the general procedure E using trimethylamine *N*-oxide (35.6 mg, 0.5 mmol, 1.0 equiv.), (E)-1,2-bis(3-fluoro-4-methoxyphenyl) diazene (69.6 mg, 0.25 mmol, 0.5 equiv.), 1.6 M LDA (0.39 mL, 0.625 mmol, 2.5 equiv.), and dry THF (5.0 mL, 0.1 M). This was followed by purification by FCC on silica gel (EtOAc/hexanes mobile phase) to isolate product as a yellow oil (63% yield, 52.9 mg, 0.16 mmol).

$^{1}$H NMR (400 MHz, $CDCl_3$) δ 6.91 – 6.84 (m, 2H), 6.82 (d, *J* = 13.4, Hz, 2H), 6.68 – 6.62 (m, 2H), 4.19 (s, 4H), 3.84 (s, 6H), 2.40 (s, 3H).

$^{13}$C {$^{1}$H} NMR (126 MHz, $CDCl_3$) δ 153.1 (d, J = 244.6 Hz), 146.2 (d, *J* = 7.6 Hz) 141.8 (d, *J* = 10.9 Hz), 114.9 (d, *J* = 3.2 Hz), 109.6 (d, *J* = 3.2 Hz), 103.8 (d, *J* = 22.7 Hz), 76.0, 57.1, 42.2.

$^{19}$F NMR (376 MHz, $CDCl_3$) δ -133.2.

IR: 2960, 1507, 1267, 865, 758 cm$^{-1}$

HRMS (ESI) m/z: [M+Na]$^+$ Calc'd for $C_{17}H_{19}F_2N_3NaO_2$ 358.1338; Found 358.1344

**4-(*tert*-butyl)-1,2-diphenyl-1,2,4-triazolidine (3p)**

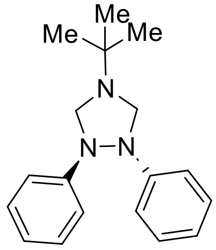

Triazolidine was prepared according to the general procedure E using *N*,*N*,2-trimethylpropan-2-amine oxide (47.7 mg, 0.4 mmol, 1.0 equiv.), azobenzene (36.7 mg, 0.2 mmol, 0.2 equiv.), 1.25 M LDA (0.80 mL, 1.0 mmol, 2.5 equiv.), and dry THF (2.0 mL, 0.2 M). This was followed by purification by FCC on silica gel (EtOAc/hexanes mobile phase) to isolate product as a yellow oil (59% yield, 33.1 mg, 0.118 mmol). Yield includes minor amount of intractable hydrazine side product as indicated in NMR spectra (Figures S54 and S55).

$^{1}$H NMR (400 MHz, $CDCl_3$) δ 7.31 – 7.24 (m, 4H), 7.05 – 6.98 (m, 4H), 6.95 – 6.88 (m, 2H), 4.31 (d, *J* = 19.2 Hz, 4H), 1.09 (s, 9H).

$^{13}$C {$^{1}$H} NMR (126 MHz, $CDCl_3$) δ 150.9, 129.0, 120.4, 114.5, 66.5, 51.9, 27.0.

IR: 2970, 2834, 1593, 1452, 795cm$^{-1}$

HRMS (ESI) m/z: $[M+H_3O]^+$ Calc'd for $C_{18}H_{26}N_3O$ 300.2070; Found 300.2077

**1,2-diphenyl-4-(2,4,4-trimethylpentan-2-yl)-1,2,4-triazolidine (3q)**

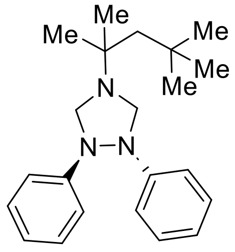

Triazolidine was prepared according to the general procedure E using *N*,*N*,2,4,4-pentamethylpentan-2-amine oxide (35.0 mg, 0.2 mmol, 1.0 equiv.), azobenzene (18 mg, 0.1 mmol, 0.5 equiv.), 1.6 M LDA (0.323 mL, 0.5 mmol, 2.5 equiv.), and dry THF (1.0 mL, 0.2 M). This was followed by purification by FCC on silica gel (EtOAc/hexanes mobile phase) to isolate product as a yellow oil (21% yield, 14.4 mg, 0.043 mmol).

$^{1}$H NMR (500 MHz, $CDCl_3$) δ 7.26 (t, *J* = 8.0 Hz, 4H), 7.00 (d, *J* = 7.8 Hz, 4H), 6.90 (t, *J* = 7.3 Hz, 2H), 4.30 (d, *J* = 17.0 Hz, 4H), 1.34 (s, 2H), 1.11 (s, 6H), 0.91 (s, 9H).

$^{13}$C {$^{1}$H} NMR (101 MHz, $CDCl_3$) δ 151.0, 128.9, 120.3, 114.7, 66.5, 55.6, 52.6, 31.7, 31.6, 26.1.

IR: 2951, 1595, 1493, 1364, 753 cm$^{-1}$

HRMS (ESI) m/z: $[M+H_3O]^+$ Calc'd for $C_{22}H_{34}N_3O$ 356.2696; Found 356.2691

**4-(adamantan-1-yl)-1,2-diphenyl-1,2,4-triazolidine (3r)**

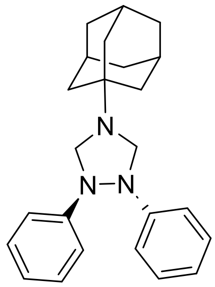

Triazolidine was prepared according to the general procedure E using *N*,*N*-dimethyladamantan-1-amine oxide (78.1 mg, 0.4 mmol, 1.0 equiv.), azobenzene (36.0 mg, 0.2 mmol, 0.5 equiv.), 1.6 M LDA (0.65 mL, 1.0 mmol, 2.5 equiv.), and dry THF (2.0 mL, 0.2 M). This was followed by purification by FCC on silica gel (EtOAc/hexanes mobile phase) to isolate product as a yellow oil (52% yield, 37.6 mg, 0.105 mmol).

$^{1}$H NMR (400 MHz, $CDCl_3$) δ 7.27 (td, *J* = 7.3, 2.1 Hz, 4H), 7.01 (dd, *J* = 8.7, 1.0 Hz, 4H), 6.95 – 6.84 (m, 2H), 4.40 (s, 2H), 4.31 (s, 2H), 2.06 (s, 3H), 1.80 – 1.41 (m, 12H).

$^{13}$C {$^{1}$H} NMR (101 MHz, CDCl3) δ 150.9, 129.0, 120.4, 114.6, 64.8, 52.2, 40.0, 36.7, 29.3.

IR: 2903, 2849, 1594, 1098, 755 cm$^{-1}$

HRMS (ESI) m/z: [M+Na+MeCN]$^+$ Calc'd for $C_{26}H_{32}N_4Na$ 423.2519; Found 423.2529

**4-methyl-1-phenyl-2-(*o*-tolyl)-1,2,4-triazolidine (3s)**

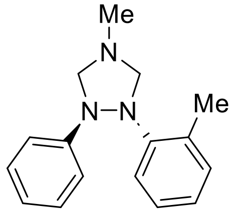


Triazolidine was prepared according to the general procedure F using trimethylamine *N*-oxide (30.0 mg, 0.4 mmol, 1.0 equiv.), (E)-1-phenyl-2-(o-tolyl)diazene (39.3 mg, 0.2 mmol, 0.5 equiv.), 1.54 M LDA (0.65 mL, 1 mmol, 5.0 equiv.), and dry THF (5.0 mL, 0.1 M). This was followed by purification by FCC on silica gel (EtOAc/hexanes mobile phase) to isolate product as a yellow oil (94% yield, 47.6 mg, 0.188 mmol).

$^{1}$H NMR (400 MHz, $CDCl_3$) δ 7.43 (dd, *J* = 8.1, 1.5 Hz, 1H), 7.25 – 7.15 (m, 3H), 7.08 (t, *J* = 7.7 Hz, 1H), 6.93 (m, 3H), 6.83 (t, *J* = 7.3 Hz, 1H), 4.72 – 4.17 (m, 2H), 4.02 (s, 2H), 2.44 (s, 6H).

$^{13}$C {$^{1}$H} NMR (126 MHz, $CDCl_3$) δ 153.1, 152.9, 133.5, 131.2, 130.2, 128.9, 125.2, 122.0, 119.9, 116.2, 79.3, 77.7, 43.9, 21.4.

IR: 3023, 2870, 1486, 1451, 1009, 735 cm$^{-1}$

HRMS (ESI) m/z: [M+H]$^+$ Calc'd for $C_{16}H_{20}N_3$ 254.1652; Found 254.1666

**4-methyl-1-phenyl-2-(*m*-tolyl)-1,2,4-triazolidine (3t)**

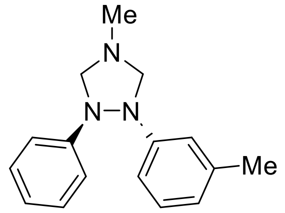

Triazolidine was prepared according to the general procedure F using trimethylamine *N*-oxide (30.0 mg, 0.4 mmol, 1.0 equiv.), (E)-1-phenyl-2-(m-tolyl)diazene (39.3 mg, 0.2 mmol, 0.5 equiv.), 1.54 M LDA (0.65 mL, 1 mmol, 5.0 equiv.), and dry THF (5.0 mL, 0.1 M). This was followed by purification by FCC on silica gel (EtOAc/hexanes mobile phase) to isolate product as a yellow oil (99% yield, 50.7 mg, 0.20 mmol).

$^1$H NMR (400 MHz, $CDCl_3$) δ 7.29 – 7.20 (m, 2H), 7.13 (t, *J* = 7.7 Hz, 1H), 6.96 (dd, *J* = 8.7, 1.2 Hz, 2H), 6.89 (t, *J* = 7.3 Hz, 1H), 6.81 – 6.69 (m, 3H), 4.33 (m, 2H), 4.24 (m, 2H), 2.38 (s, 3H), 2.30 (s, 3H).

$^{13}$C {$^1$H} NMR (126 MHz, $CDCl_3$) δ 151.6, 151.6, 137.4, 129.0, 128.9, 121.5, 120.5, 115.1, 114.5, 112.2, 75.4, 75.3, 41.6, 21.6.

IR: 3029, 2866, 1596, 1487, 1287, 734 cm$^{-1}$

HRMS (ESI) m/z: $[M+H]^+$ Calc'd for $C_{16}H_{20}N_3$ 254.1652; Found 254.1655

**4-methyl-1-phenyl-2-(*p*-tolyl)-1,2,4-triazolidine (3u)**

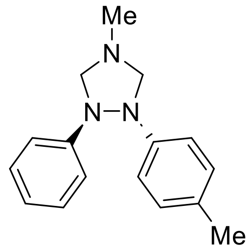

Triazolidine was prepared according to the general procedure F using trimethylamine *N*-oxide (30.0 mg, 0.4 mmol, 1.0 equiv.), (E)-1-phenyl-2-(p-tolyl)diazene (39.2 mg, 0.2 mmol, 0.5 equiv.), 1.54 M LDA (0.65 mL, 1 mmol, 5.0 equiv.), and dry THF (5.0 mL, 0.1 M). This was followed by purification by FCC on silica gel (15 % EtOAc in Hexanes) to isolate product as a yellow oil (55% yield, 27.8 mg, 0.11 mmol)

$^1$H NMR (500 MHz, $CDCl_3$) δ 7.24 (m, 2H), 7.06 (d, *J* = 8.4 Hz, 2H), 6.95 (d, *J* = 8.9 Hz, 2H), 6.89 (m, 3H), 4.29 (m, 3H), 4.22 (d, *J* = 8.4 Hz, 1H), 2.39 (s, 3H), 2.27 (s, 3H).

$^{13}$C {$^1$H} NMR (126 MHz, $CDCl_3$) δ 151.5, 149.4, 129.9, 129.7, 129.1, 120.3, 114.5, 114.3, 75.7, 75.2, 42.5, 20.5.

IR: 3024, 2859, 1507, 1289, 737 cm$^{-1}$

HRMS (ESI) m/z: $[M+H+MeCN]^+$ Calc'd for $C_{18}H_{23}N_4$ 295.1917; Found 295.1917

**1-(4-methoxyphenyl)-4-methyl-2-phenyl-1,2,4-triazolidine (3v)**

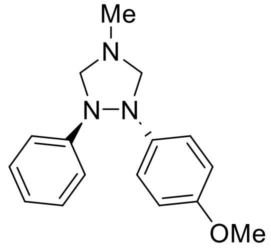

Triazolidine was prepared according to the general procedure F using trimethylamine *N*-oxide (30.0 mg, 0.4 mmol, 1.0 equiv.), (E)-1-(4-methoxyphenyl)-2-phenyldiazene (42.4 mg, 0.2 mmol, 0.5 equiv.), 1.54 M LDA (0.65 mL, 1 mmol, 5.0 equiv.), and dry THF (5.0 mL, 0.1 M). This was followed by purification by FCC on silica gel (EtOAc/hexanes mobile phase) to isolate product as a yellow oil (81% yield, 43.6 mg, 0.16 mmol).

$^1$H NMR (400 MHz, $CDCl_3$) δ 7.28 – 7.21 (m, 2H), 6.98 – 6.92 (m, 4H), 6.88 (t, J = 7.3 Hz, 1H), 6.82 (dd, J = 9.0, 2.4 Hz, 2H), 4.43 (m, 3H), 4.19 (s, 1H)., 3.76 (s, 3H), 2.40 (s, 3H).

$^{13}$C {$^1$H} NMR (126 MHz, CDCl3) δ 154.2, 151.4, 145.7, 129.1, 120.2, 115.8, 114.5, 114.2, 76.2, 75.1, 55.7, 42.3.

IR: 3028, 2970, 1497, 1218, 1073 cm$^{-1}$

HRMS (ESI) m/z: $[M+Na+H_2O]^+$ Calc'd for $C_{16}H_{21}N_3NaO_2$ 310.1526; Found 310.1544

**4-methyl-1-(4-phenoxyphenyl)-2-phenyl-1,2,4-triazolidine (3w)**

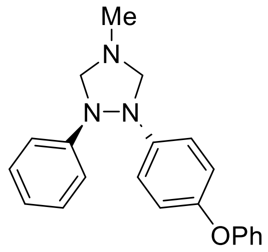

Triazolidine was prepared according to the general procedure F using trimethylamine *N*-oxide (60.0 mg, 0.8 mmol, 1.0 equiv.), (E)-1-(4-phenoxyphenyl)-2-phenyldiazene (109.7 mg, 0.4 mmol, 0.5 equiv.), 1.4 M LDA (0.70 mL, 1 mmol, 2.5 equiv.), and dry THF (5.0 mL, 0.1 M). This was followed by purification by FCC on silica gel (EtOAc/hexanes mobile phase) to isolate product as a yellow oil (85% yield, 112.0 mg, 0.34 mmol).

$^1$H NMR (400 MHz, $CDCl_3$) δ 7.32 – 7.21 (m, 4H), 7.06 – 6.87 (m, 10H), 4.31 (s, 3H), 4.24 (s, 1H)., 2.41 (s, 3H)

$^{13}$C {$^1$H} NMR (126 MHz, $CDCl_3$) δ 158.5, 151.4, 150.4, 147.9, 129.6, 129.2, 123.2, 120.6, 120.5, 117.7, 116.3, 114.4, 75.9, 75.4, 42.3.

IR: 3038, 2860, 1596, 1288, 1226, 752 cm$^{-1}$

HRMS (ESI) m/z: $[M+H]^+$ Calc'd for $C_{21}H_{22}N_3O$ 332.1757; Found 332.1755

**1-(4-((*tert*-butyldimethylsilyl)oxy)phenyl)-4-methyl-2-phenyl-1,2,4-triazolidine (3x)**

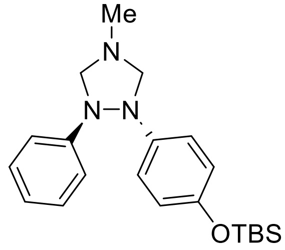

Triazolidine was prepared according to the general procedure F using trimethylamine *N*-oxide (30.0 mg, 0.4 mmol, 1.0 equiv.), (E)-1-(4-((*tert*-butyldimethylsilyl)oxy)phenyl)-2-phenyldiazene (0.13 mL, 0.2 mmol, 0.5 equiv.), 1.6 M LDA (0.62 mL, 1 mmol, 5.0 equiv.), and dry THF (5.0 mL, 0.1 M). This was followed by purification by FCC on silica (EtOAc/hexanes mobile phase) to isolate product as a yellow oil (84% yield, 62.0 mg, 0.17 mmol).

$^1$H NMR (400 MHz, , $CDCl_3$) δ 7.29 – 7.19 (m, 2H), 6.99 – 6.92 (m, 2H), 6.91 – 6.82 (m, 3H), 6.76 – 6.68 (m, 2H), 4.28 (s, 3H), 4.18 (s, 1H), 2.38 (s, 3H), 0.96 (s, 9H), 0.16 (s, 6H).

$^{13}$C {$^1$H} NMR (126 MHz, $CDCl_3$) δ 152.2, 149.8, 145.9, 128.3, 120.4, 119.5, 116.6, 114.9, 76.1, 75.2, 43.8, 25.8, 18.2, -4.4.

IR: 2954, 2930, 2857, 1498, 1247, 908, 719 cm$^{-1}$

HRMS (ESI) m/z: $[M+Na+H_2O]^+$ Calc'd for $C_{21}H_{33}N_3NaO_2Si$ 410.2234; Found 410.2263

**4-methyl-1-(4-(methylthio)phenyl)-2-phenyl-1,2,4-triazolidine (3y)**

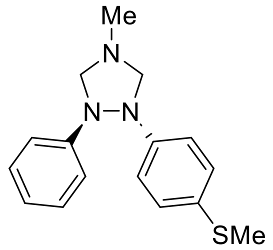

Triazolidine was prepared according to the general procedure F using trimethylamine *N*-oxide (30.0 mg, 0.4 mmol, 1.0 equiv.), (E)-1-(4-(methylthio)phenyl)-2-phenyldiazene (45.6 mg, 0.2 mmol, 0.5 equiv.), 1.54 M LDA (0.65 mL, 1 mmol, 5.0 equiv.), and dry THF (5.0 mL, 0.1 M). This was followed by purification by FCC on silica (EtOAc/hexanes mobile phase) to isolate product as a yellow oil (60% yield, 62.0 mg, 0.17 mmol).

$^1$H NMR (400 MHz, $CDCl_3$) δ 7.31 – 7.24 (m, 4H), 6.99 – 6.90 (m, 5H), 4.40 – 4.23 (m, 4H), 2.46 (s, 3H), 2.42 (s, 3H).

$^{13}$C {$^1$H} NMR (126 MHz, $CDCl_3$) δ 151.3, 150.0, 129.9, 129.1, 128.4, 120.6, 115.2, 114.4, 75.4, 75.4, 42.5, 18.1.

IR: 3060, 2859, 1488, 1288, 749, 694 cm$^{-1}$

HRMS (ESI) m/z: $[M+H]^+$ Calc'd for $C_{16}H_{20}N_3S$ 286.1373; Found 286.1391

**4-methyl-1-phenyl-2-(4-(trifluoromethyl)phenyl)-1,2,4-triazolidine (3z)**

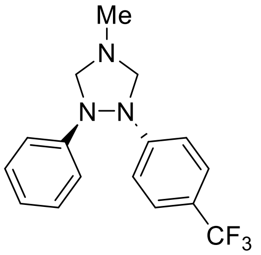

Triazolidine was prepared according to the general procedure F using trimethylamine *N*-oxide (30.0 mg, 0.4 mmol, 1.0 equiv.), (E)-1-phenyl-2-(4-(trifluoromethyl)phenyl)diazene (50.0 mg, 0.2 mmol, 0.5 equiv.), 1.54 M LDA (0.65 mL, 1 mmol, 5.0 equiv.), and dry THF (5.0 mL, 0.1 M). This was followed by purification by FCC on silica (EtOAc/hexanes mobile phase) to isolate product as a yellow oil (26% yield, 16.0 mg, 0.05 mmol).

$^{1}$H NMR (400 MHz, $CDCl_3$) δ 7.48 (d, J = 9.0 Hz, 2H), 7.28 – 7.24 (m, 2H), 7.03 – 6.89 (m, 5H), 4.46 – 4.33 (m, 3H), 4.29 – 4.22 (m, 1H), 2.39 (s, 3H).

$^{13}$C {$^{1}$H} NMR (126 MHz, $CDCl_3$) δ 153.7, 151.0, 129.2, 126.4 (q, J = 3.9 Hz), 124.7 (q, *J* = 272.2 Hz) 122.1 (q, J = 32.8 Hz), 121.1, 114.6, 113.8, 75.8, 74.7, 42.6.

$^{19}$F NMR (376 MHz, CDCl3) δ -61.3.

IR: 2945, 2807, 2163, 1595, 1317, 1108, 1007 $cm^{-1}$

HRMS (ESI) m/z: $[M+H]^+$ Calc'd for $C_{16}H_{17}F_3N_3$ 308.1369; Found 308.1344

**1-(4-fluorophenyl)-4-methyl-2-phenyl-1,2,4-triazolidine (3aa)**

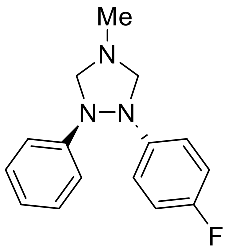

Triazolidine was prepared according to the general procedure F using trimethylamine *N*-oxide (60.0 mg, 0.8 mmol, 1.0 equiv.), (*E*)-1-(4-fluorophenyl)-2-phenyldiazene (80.0 mg, 0.4 mmol, 0.5 equiv.), 1.4 M LDA (0.70 mL, 1 mmol, 2.5 equiv.), and dry THF (5.0 mL, 0.1 M). This was followed by purification by FCC on silica gel (EtOAc/hexanes mobile phase) to isolate product as a yellow oil (70% yield, 70.0 mg, 0.28 mmol).

$^{1}$H NMR (400 MHz, $CDCl_3$) δ 7.30 – 7.19 (m, 2H), 6.99 – 6.86 (m, 7H), 4.28 (m 3H), 4.22 (m, 1H), 2.39 (s, 3H)

$^{13}$C {$^{1}$H} NMR (126 MHz, $CDCl_3$) δ 157.7 (d, J = 238.4 Hz), 151.3, 147.9 (d, J = 2.2 Hz), 129.2, 120.6, 115.7 (d, J = 7.9 Hz), 115.5, 114.3, 76.0, 75.3, 42.3.

$^{19}$F NMR (3766 MHz, CDCl3) δ -124.5.

IR: 3037, 2950, 1492, 1289, 1238, 1031 $cm^{-1}$

HRMS (ESI) m/z: $[M+H+MeCN]^+$ Calc'd for $C_{17}H_{20}FN_4$ 299.1667; Found 299.1693

**4-methyl-7-(4-methyl-2-phenyl-1,2,4-triazolidin-1-yl)-2H-chromen-2-one (3ab)**

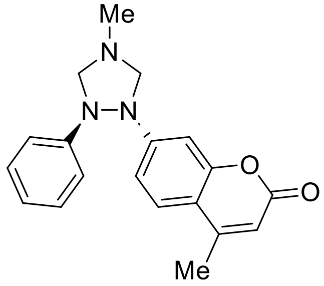


Triazolidine was prepared according to the general procedure F using trimethylamine *N*-oxide (30.0 mg, 0.4 mmol, 1.0 equiv.), (E)-4-methyl-7-(phenyldiazenyl)-2H-chromen-2-one (53.2 mg, 0.2 mmol, 0.5 equiv.), 1.4 M LDA (0.32 mL, 1 mmol, 2.5 equiv.), and dry THF (5.0 mL, 0.1 M). This was followed by purification by FCC on silica gel (EtOAc/hexanes mobile phase) to isolate the product as a yellow oil (39% yield, 25.0 mg, 0.08 mmol)

$^1$H NMR (500 MHz, $CDCl_3$) δ 7.45 (d, *J* = 8.7 Hz, 1H), 7.31 – 7.25 (t, *J* = 8 Hz, 2H), 6.98 – 6.92 (m, 3H), 6.89 (dd, *J* = 8.7, 2.3 Hz, 1H), 6.86 (d, *J* = 2.2 Hz, 1H), 6.08 (s, 1H), 4.44 (d, *J* = 8.3 Hz, 1H), 4.39 (d, *J* = 2.6 Hz, 2H), 4.28 (d, *J* = 8.1 Hz, 1H), 2.41 (s, 3H), 2.37 (s, 3H).

$^{13}$C {$^1$H} NMR (126 MHz, $CDCl_3$) δ 161.5, 155.2, 154.2, 152.6, 150.7, 129.3, 125.5, 121.4, 114.7, 113.2, 111.3, 110.8, 101.3, 76.1, 74.4, 42.7, 18.6.

IR: 1739, 1576, 1505, 1232, 1010, 734 cm$^{-1}$

HRMS (ESI) m/z: [M+H+MeCN]$^+$ Calc'd for $C_{21}H_{23}N_4O_2$ 363.1816; Found 363.1817

**4-(adamantan-1-yl)-3-cyclopropyl-1,2-diphenyl-1,2,4-triazolidine (5)**

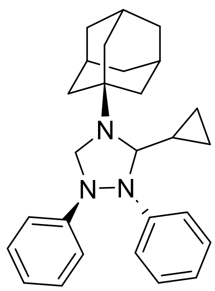


Triazolidine was prepared according to the general procedure F using *N*-(cyclopropylmethyl)-*N*-methyladamantan-1-amine *N*-oxide (81 mg, 0.34 mmol, 1.0 equiv., azobenzene (31 mg, 0.17 mmol, 0.5 equiv.), 1.55 M LDA (0.55 mL, 0.86 mmol, 2.5 equiv.), and dry THF (1.7 mL, 0.2 M). Product readily degraded during purification, yield obtained via NMR.[7] (25% yield, 17 mg, 0.043 mmol). Product **5** was highly unstable to water and silica with small amounts of phenyl hydrazine in the spectra (Figure S80).

$^1$H NMR (400 MHz, $CDCl_3$) δ 7.23 (m, 4H), 6.91 – 6.83 (m, 6H), 4.80 (d, *J* = 7.1 Hz, 1H), 4.67 (d, *J* = 5.6 Hz, 1H), 4.48 (d, *J* = 7.2 Hz, 1H), 1.98 (s, 3H), 1.60 – 1.54 (m, 6H), 1.52 – 1.49 (m, 3H) 1.40 (d, *J* = 11.8 Hz, 3H), 1.07 – 0.97 (m, 1H), 0.45 – 0.39 (m, 2H), 0.38 – 0.31 (m, 2H).

$^{13}$C {$^1$H} NMR (101 MHz, $CDCl_3$) δ 150.5, 150.2, 128.9, 128.8, 120.0, 119.4, 114.2, 114.1, 80.0, 65.0, 53.3, 41.4, 36.4, 29.5, 17.7, 2.9, 2.7.

IR: 2920, 2851, 1491, 1454, 1082, 789 cm$^{-1}$

HRMS (ESI) m/z: [M+Na]$^+$ Calc'd for $C_{27}H_{33}N_3Na$ 422.2567; Found 422.2591

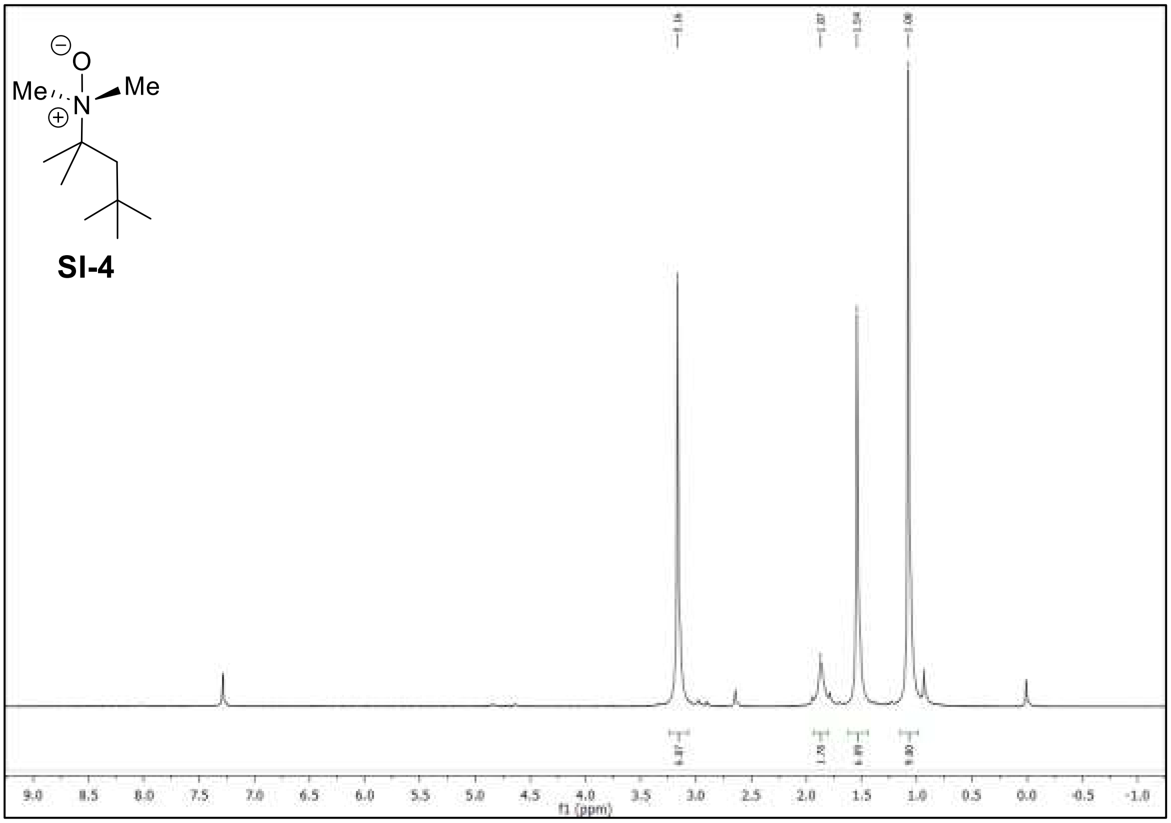


Figure S1: $^{1}$H NMR spectrum of **SI-4** (in $CDCl_3$, 400 MHz)

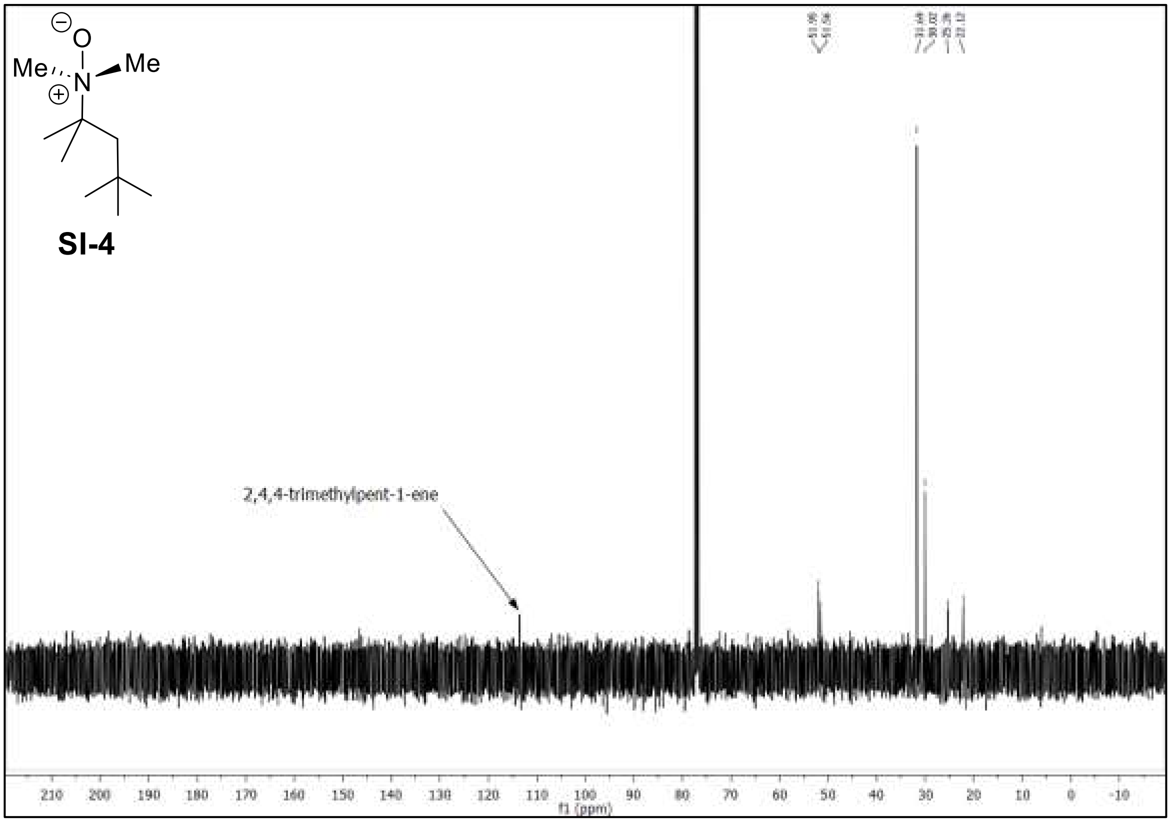


Figure S2: $^{13}C$ {$^{1}H$} NMR spectrum of **SI-4** (in $CDCl_3$, 101 MHz)

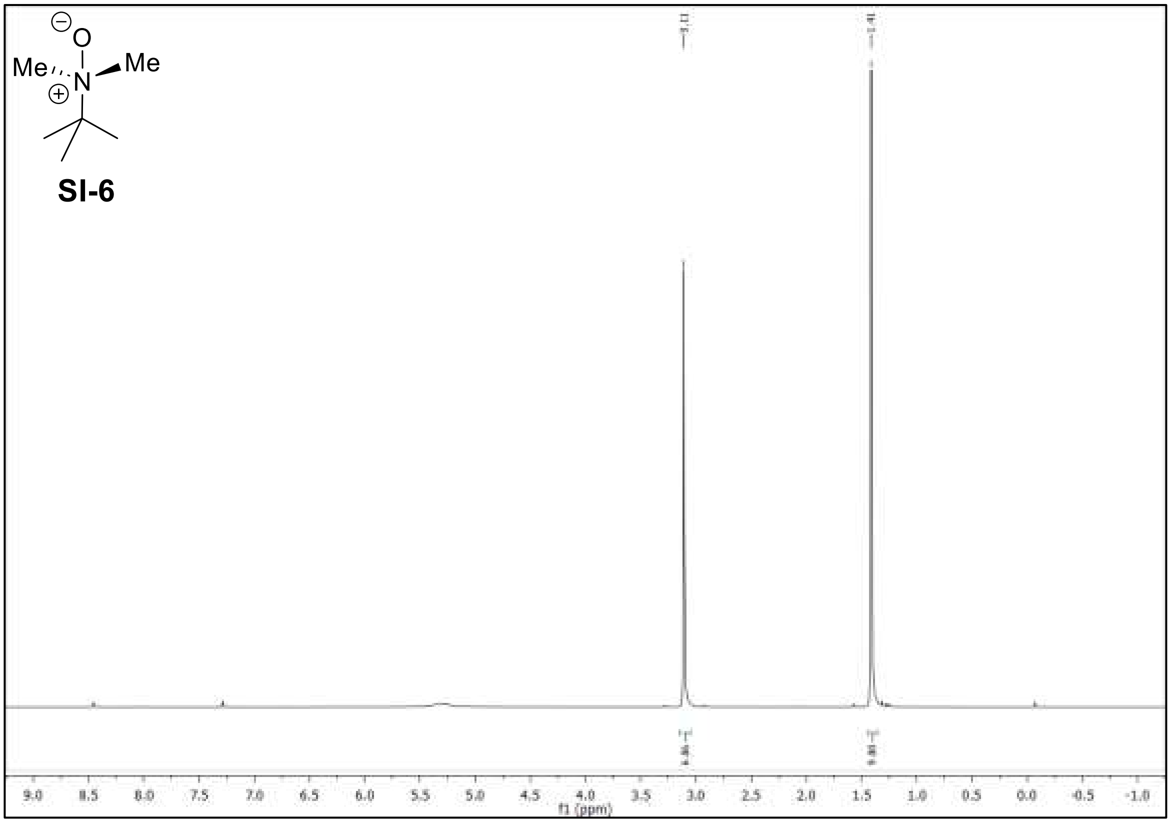

Figure S3: $^1$H NMR spectrum of **SI-6** (in $CDCl_3$, 400 MHz)

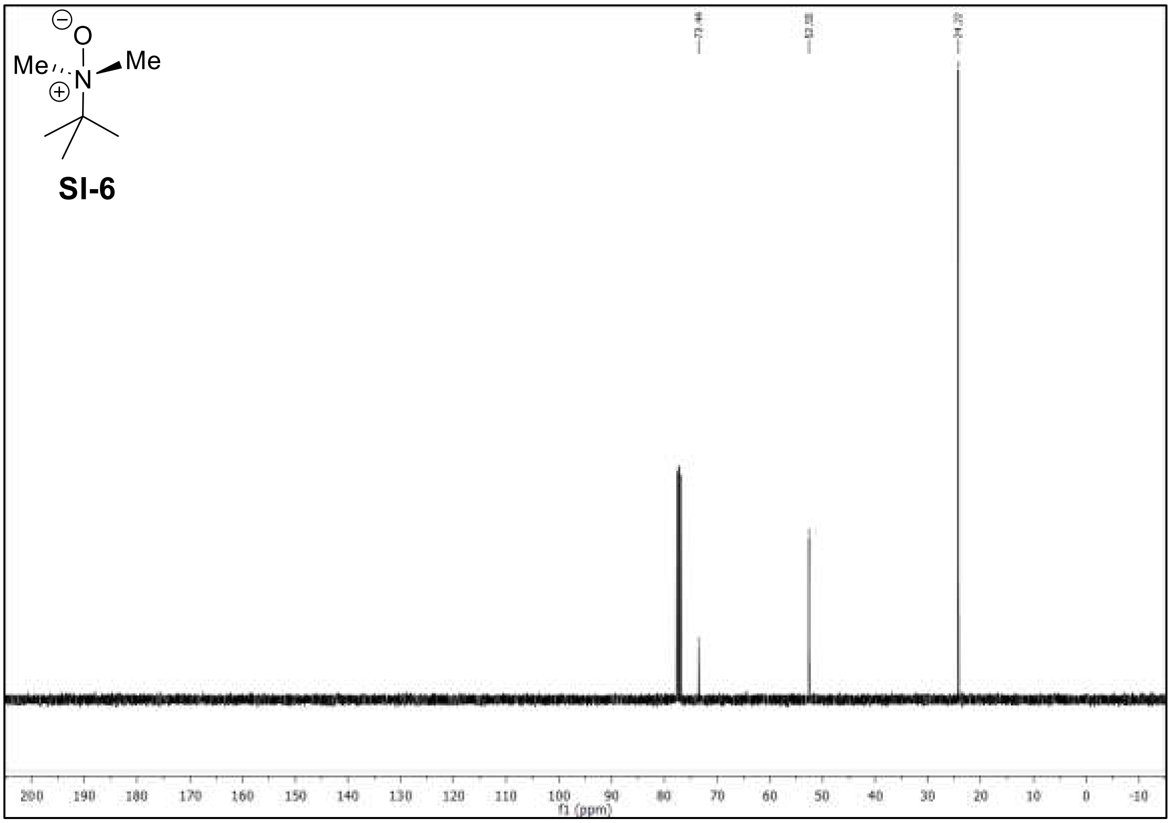

Figure S4: $^{13}C$ {$^1$H} NMR spectrum of **SI-6** (in $CDCl_3$, 101 MHz)

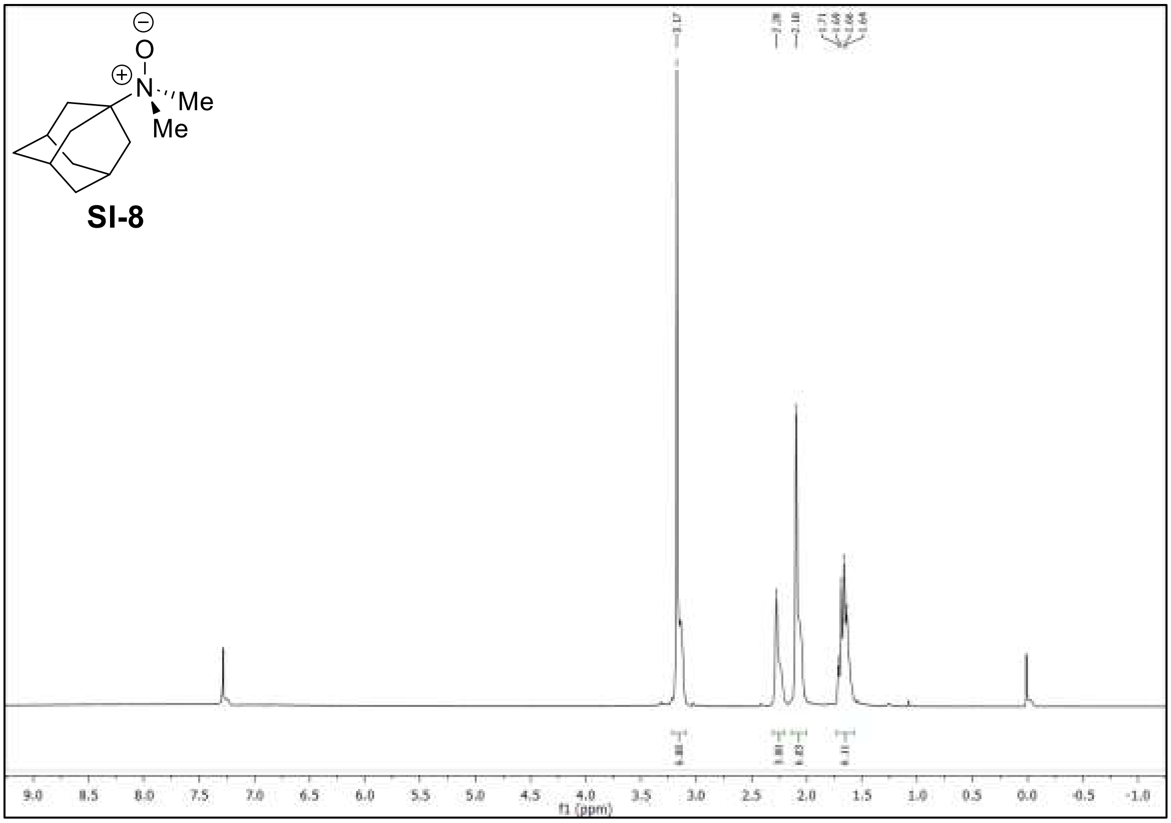


Figure S5: $^{1}H$ NMR spectrum of **SI-8** (in $CDCl_3$, 500 MHz)

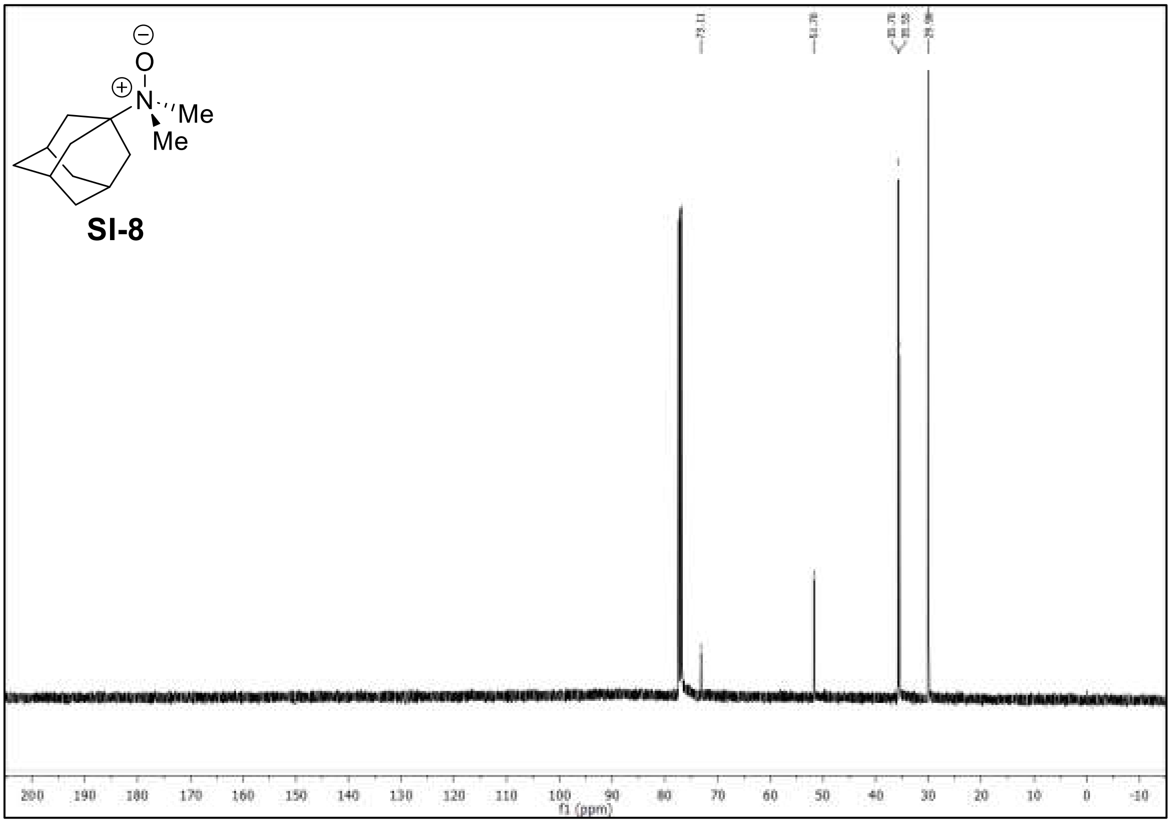


Figure S6: $^{13}C$ {$^{1}H$} NMR spectrum of **SI-8** (in $CDCl_3$, 126 MHz)

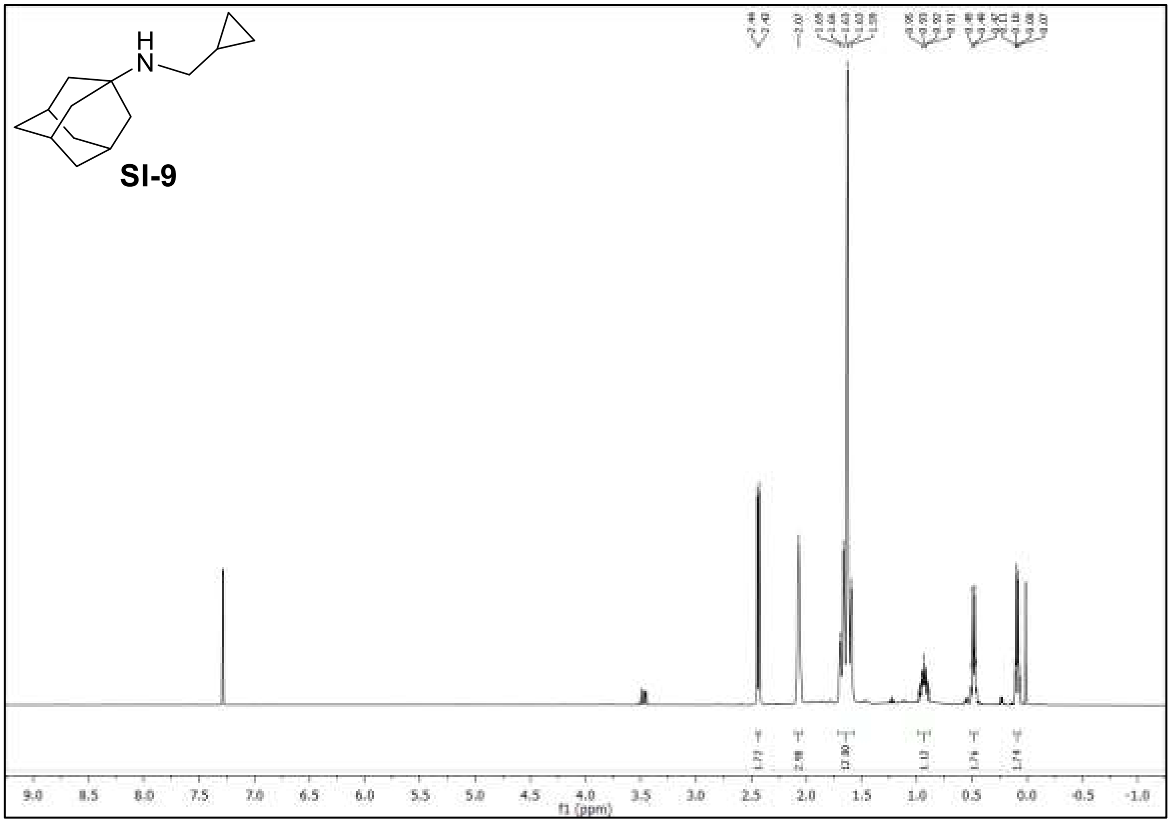


Figure S7: $^{1}H$ NMR spectrum of **SI-9** (in $CDCl_3$, 400 MHz)

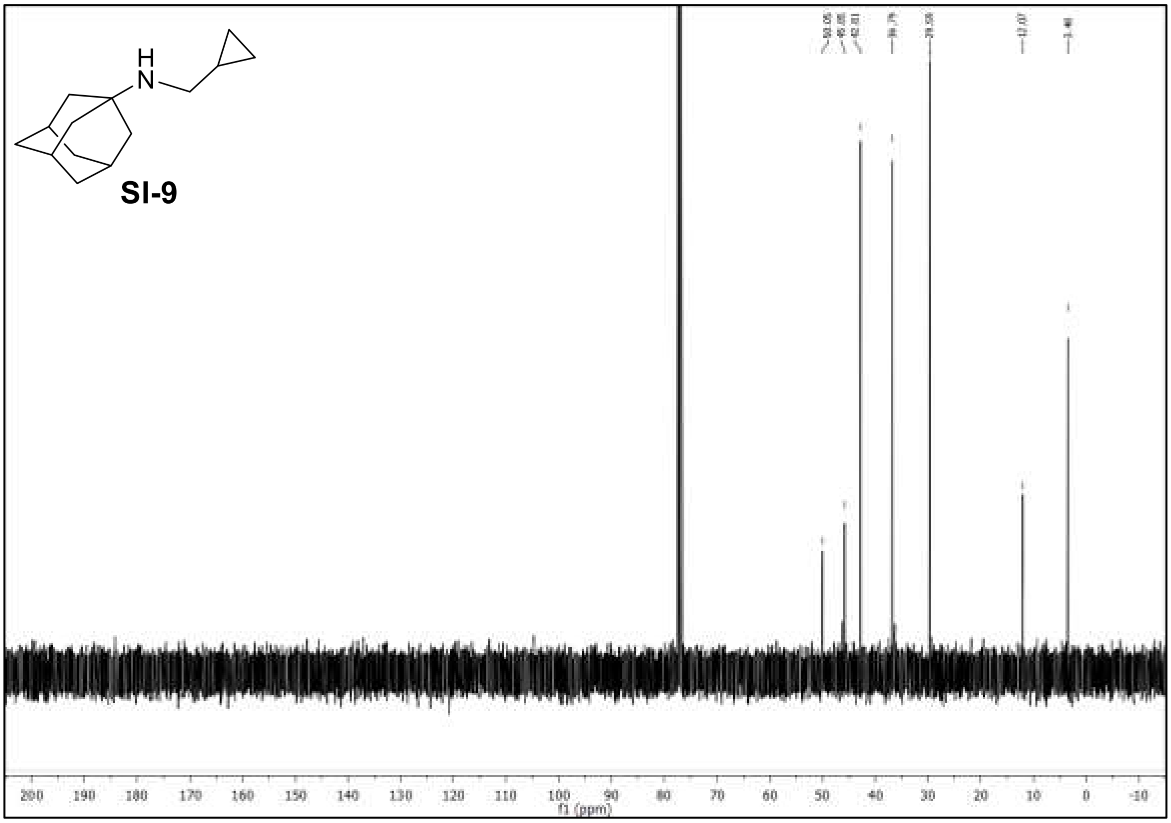


Figure S8: $^{13}C$ {$^{1}H$} NMR spectrum of **SI-9** (in $CDCl_3$, 101 MHz)

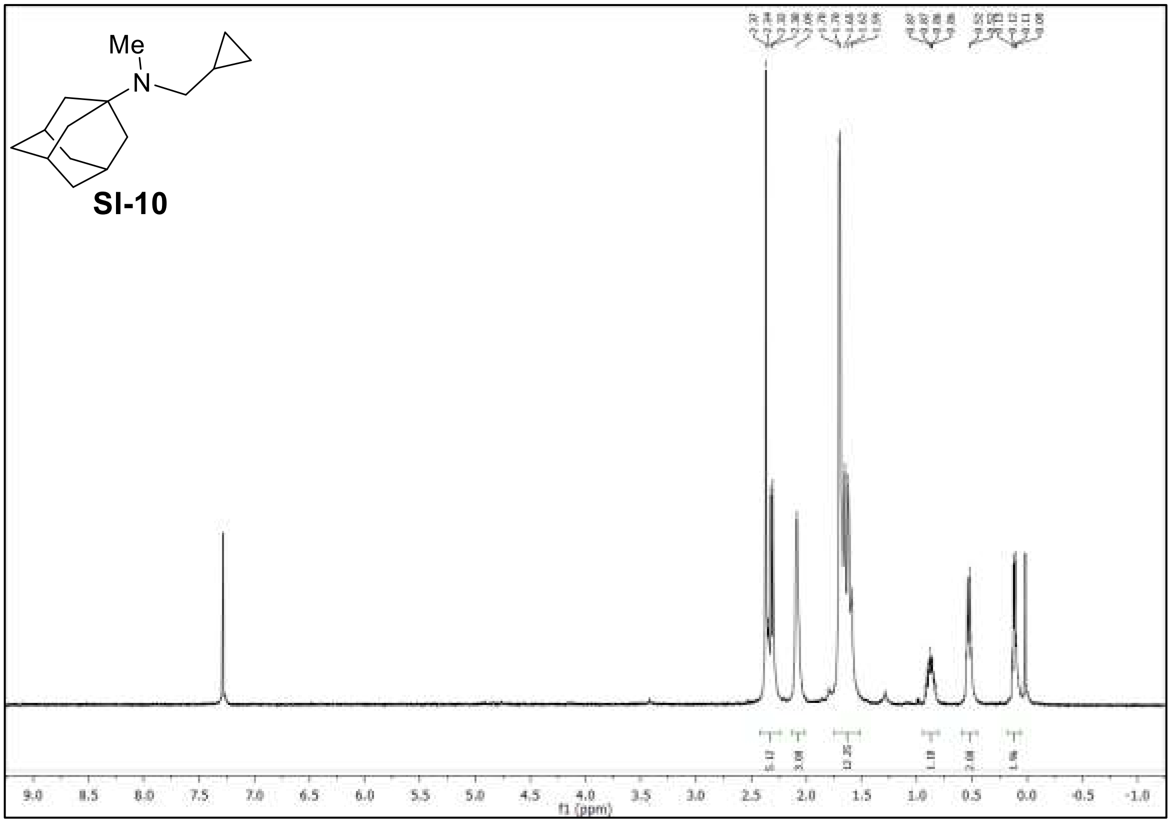


Figure S9: $^{1}H$ NMR spectrum of **SI-10** (in $CDCl_3$, 400 MHz)

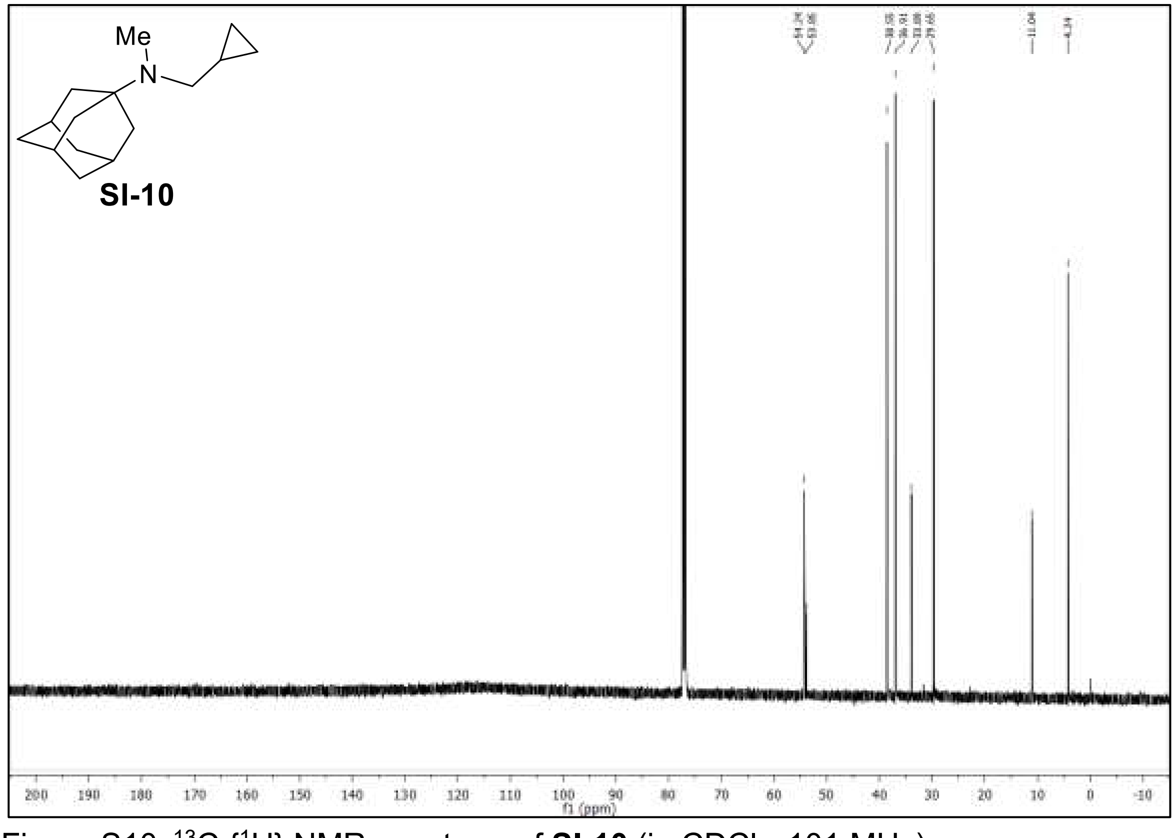


Figure S10: $^{13}C$ {$^{1}H$} NMR spectrum of **SI-10** (in $CDCl_3$, 101 MHz)

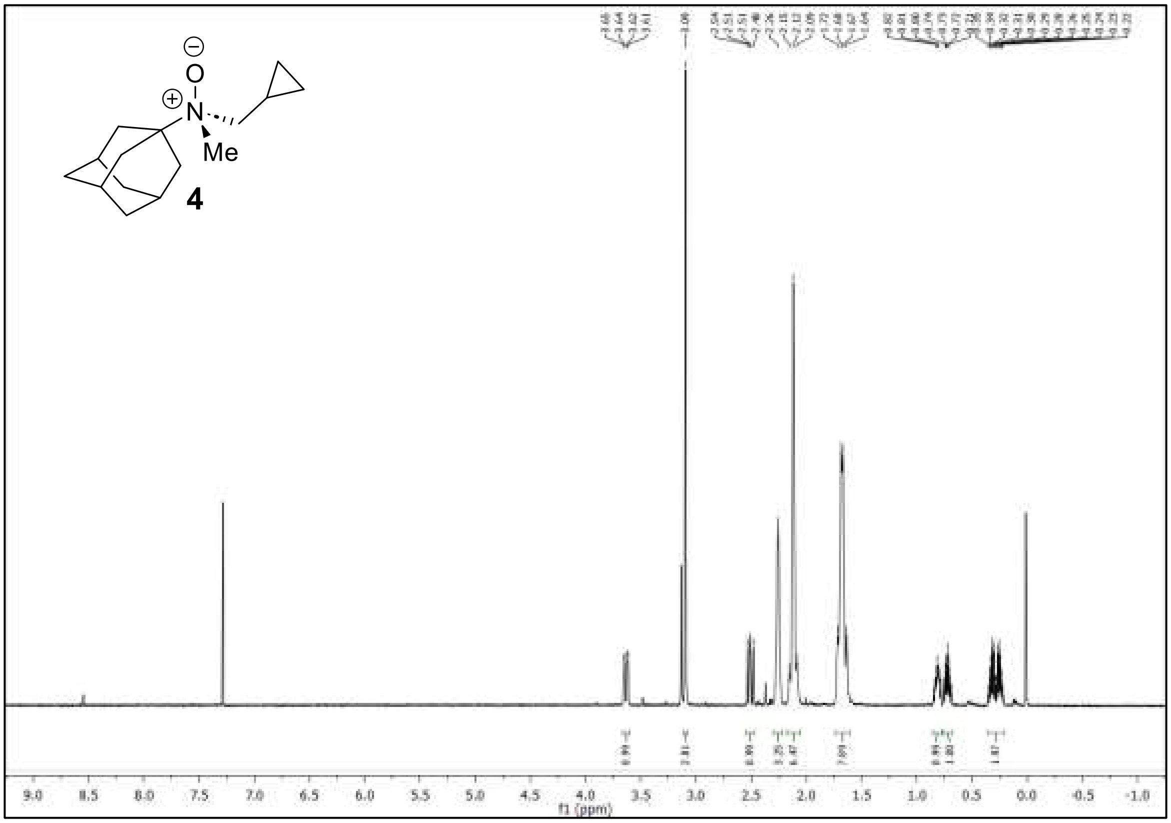

Figure S11: $^{1}H$ NMR spectrum of **4** (in $CDCl_3$, 400 MHz)

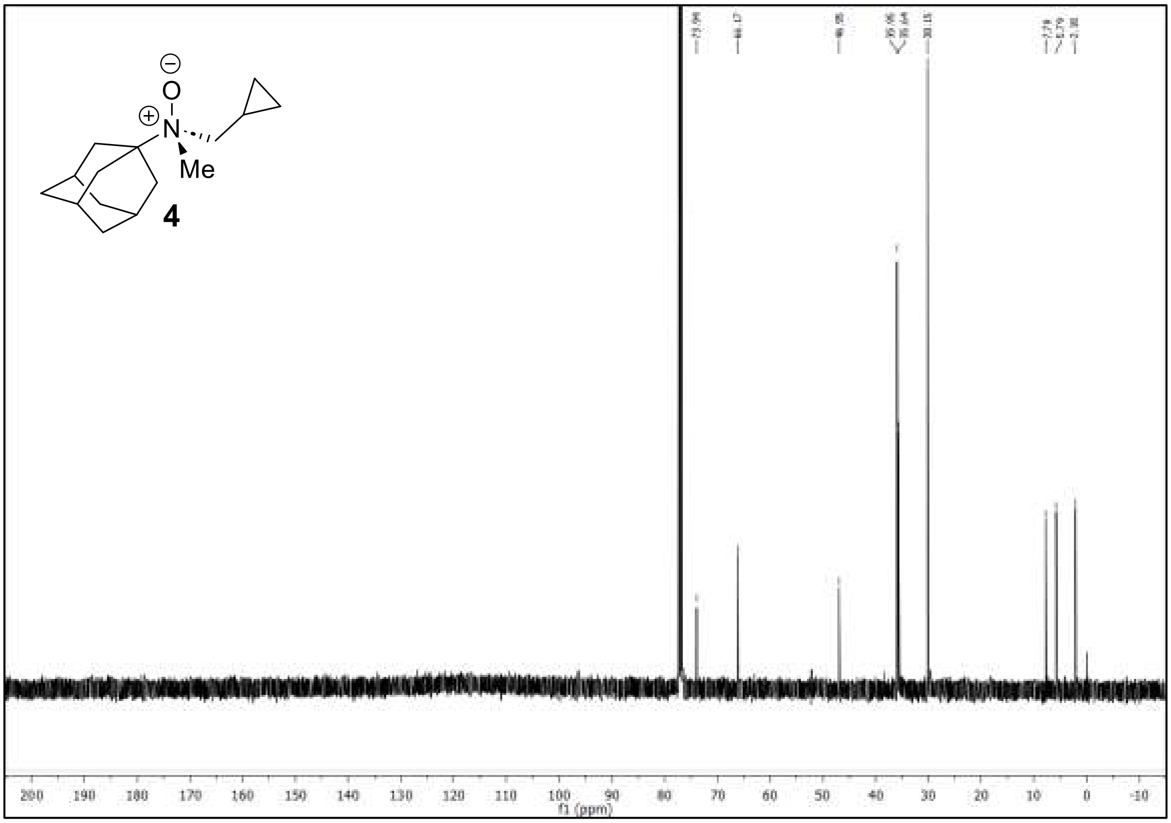

Figure S12: $^{13}C\{^{1}H\}$ NMR spectrum of **4** (in $CDCl_3$, 126 MHz)

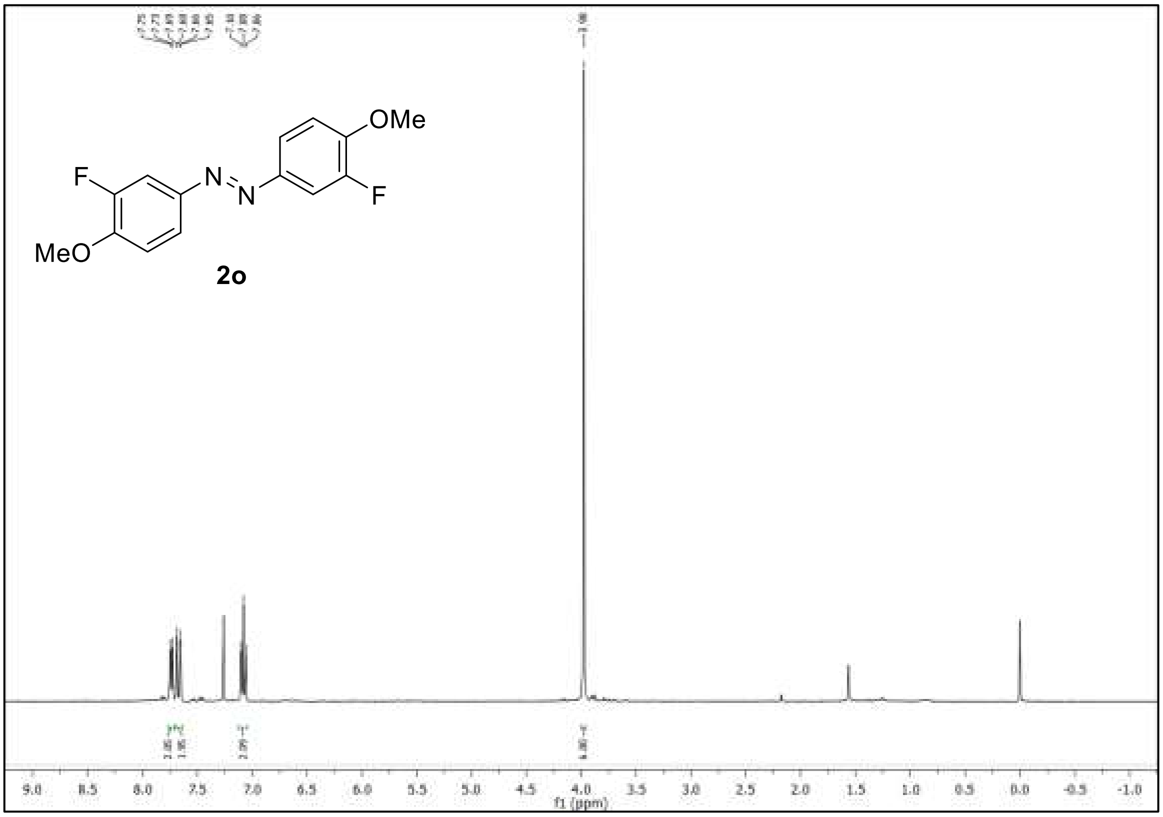

Figure S13: $^{1}H$ NMR spectrum of **2o** (in $CDCl_3$, 400 MHz)

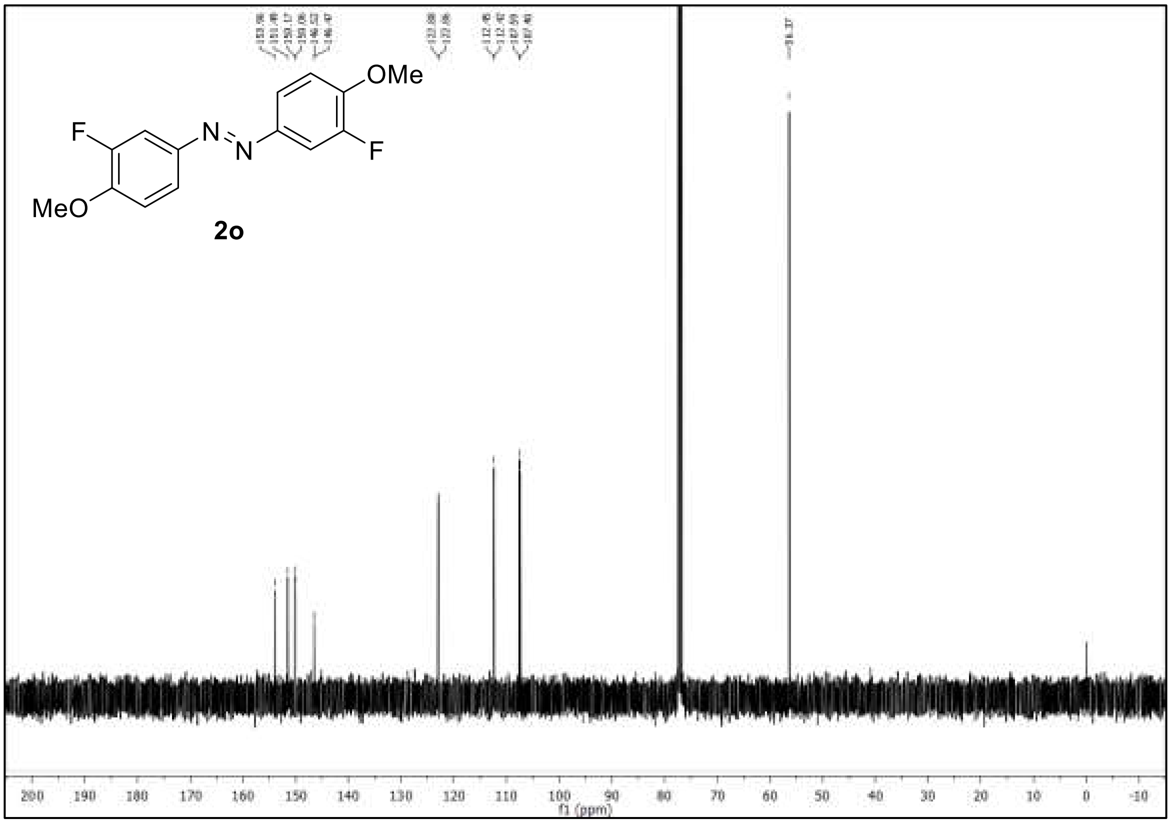

Figure S14: $^{13}C$ {$^{1}H$} NMR spectrum of **2o** (in $CDCl_3$, 101 MHz)

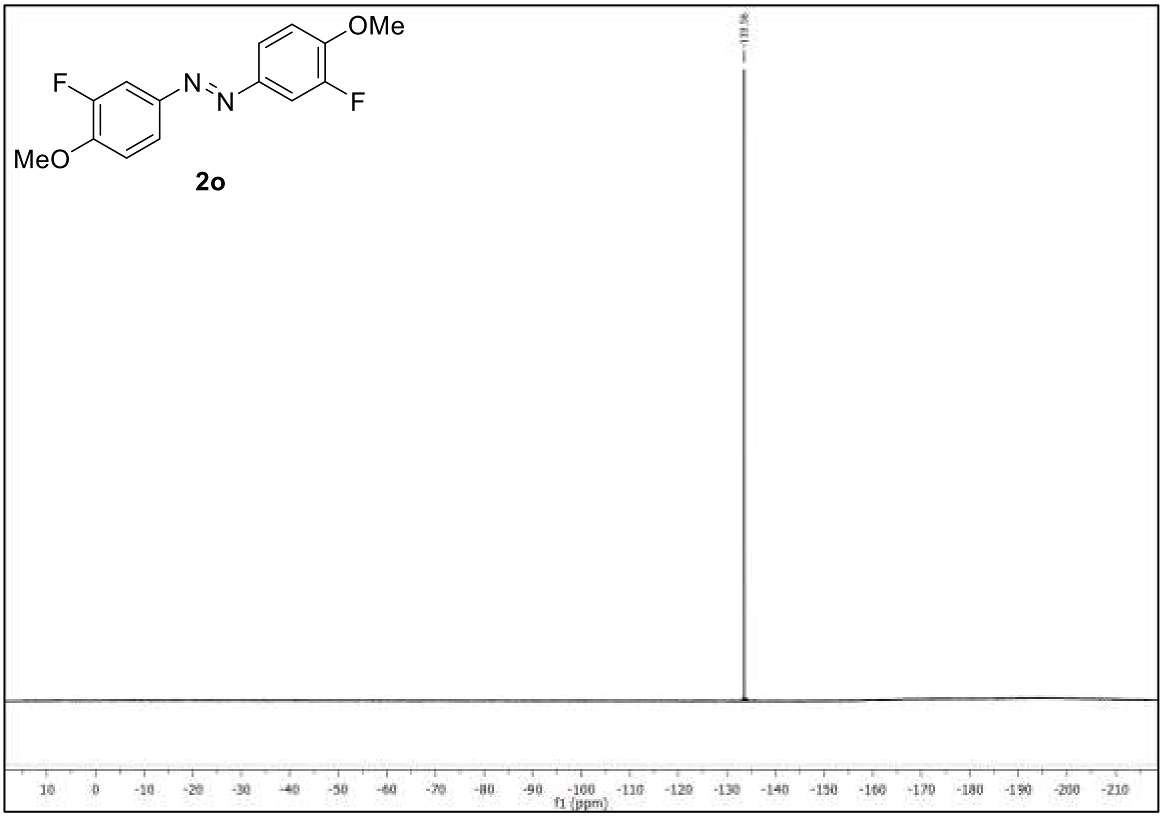

Figure S15: $^{19}$F NMR spectrum of **2o** (in $CDCl_3$, 376 MHz)

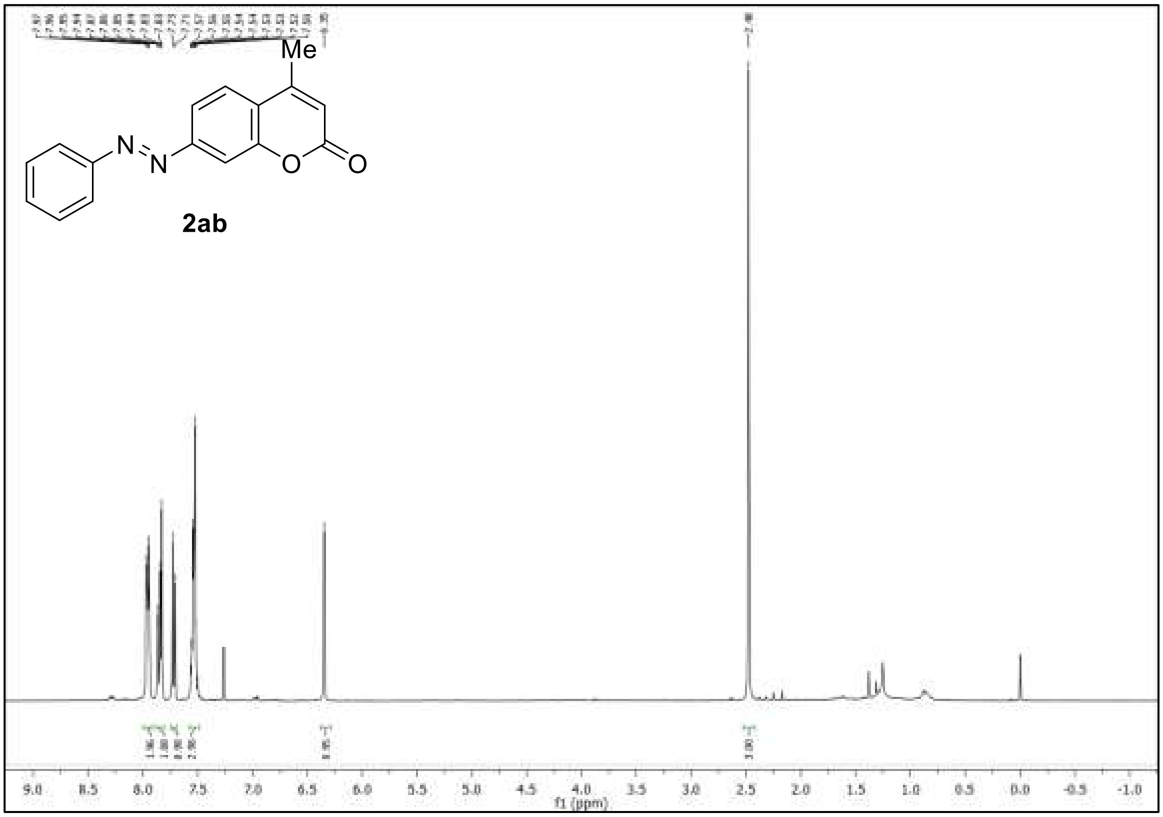

Figure S16: $^{1}H$ NMR spectrum of **2ab** (in $CDCl_3$, 400 MHz)

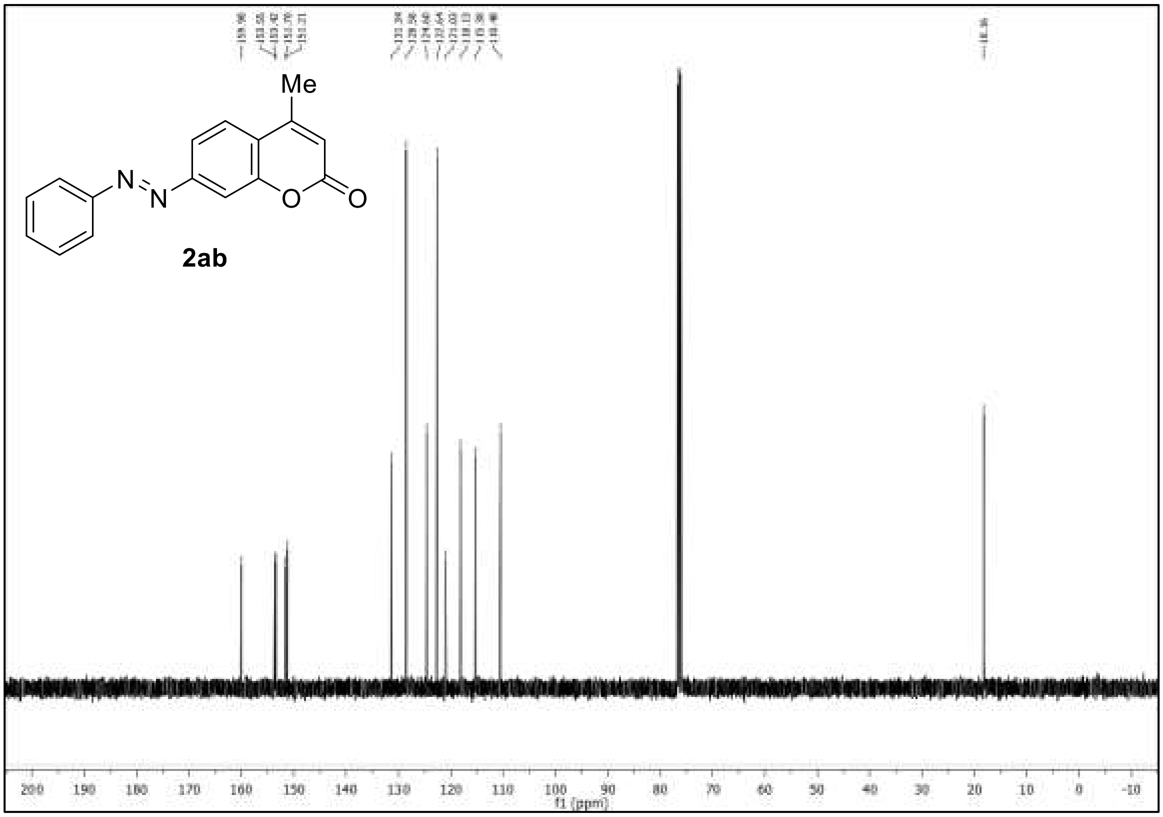

Figure S17: $^{13}C$ {$^{1}H$} NMR spectrum of **2ab** (in $CDCl_3$, 101 MHz)

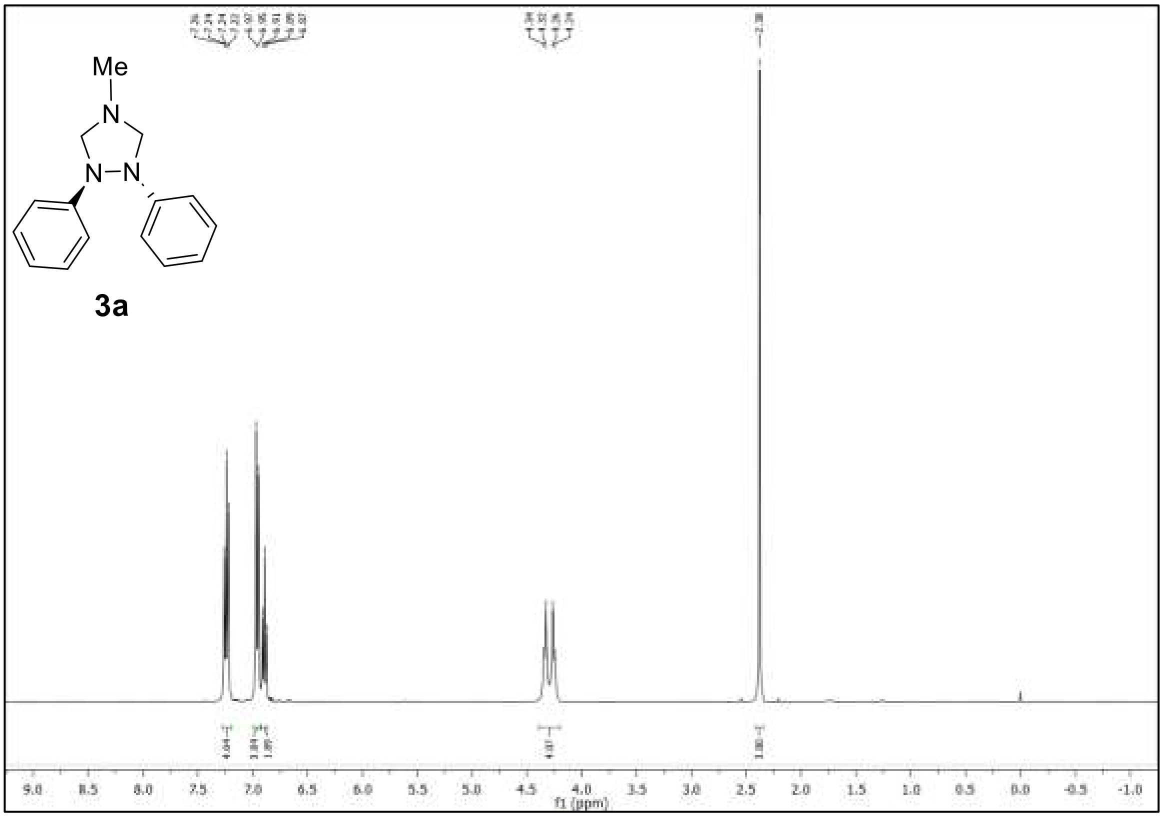

Figure S18: $^{1}$H NMR spectrum of **3a** (in $CDCl_3$, 400 MHz)

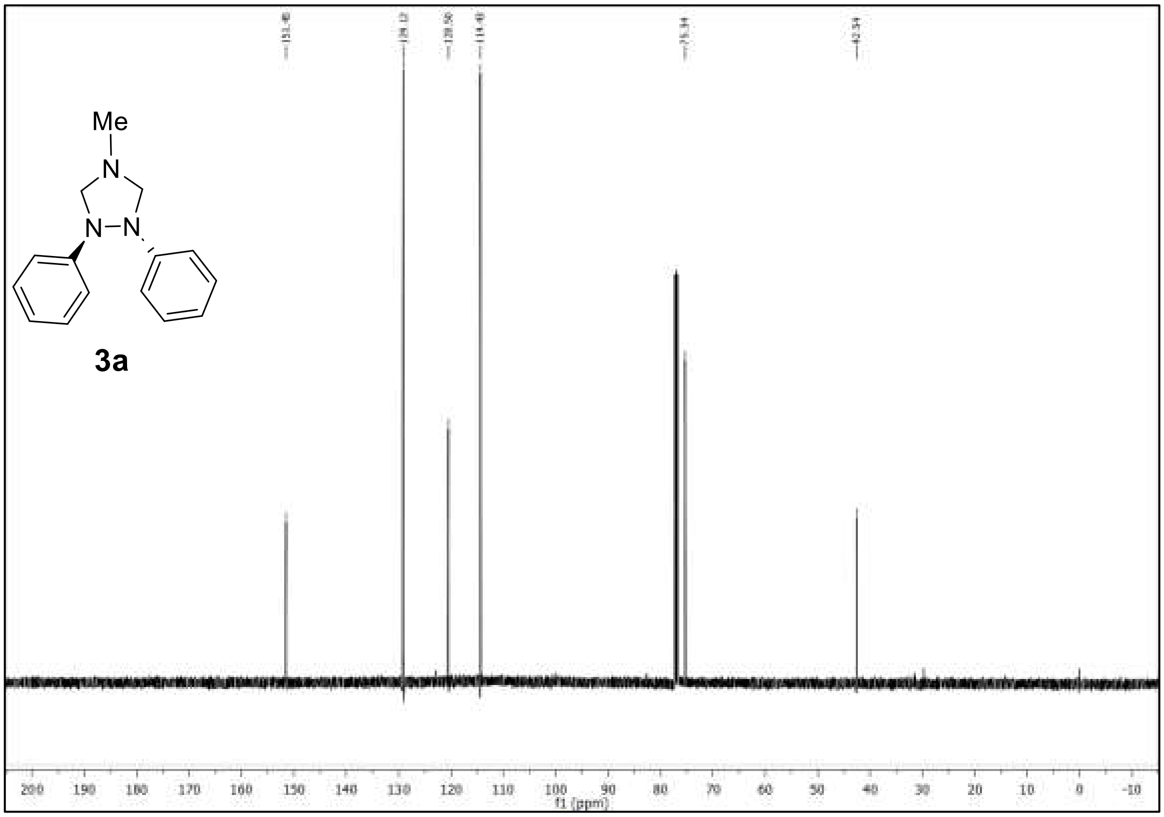

Figure S19: $^{13}$C {$^{1}$H} NMR spectrum of **3a** (in $CDCl_3$, 126 MHz)

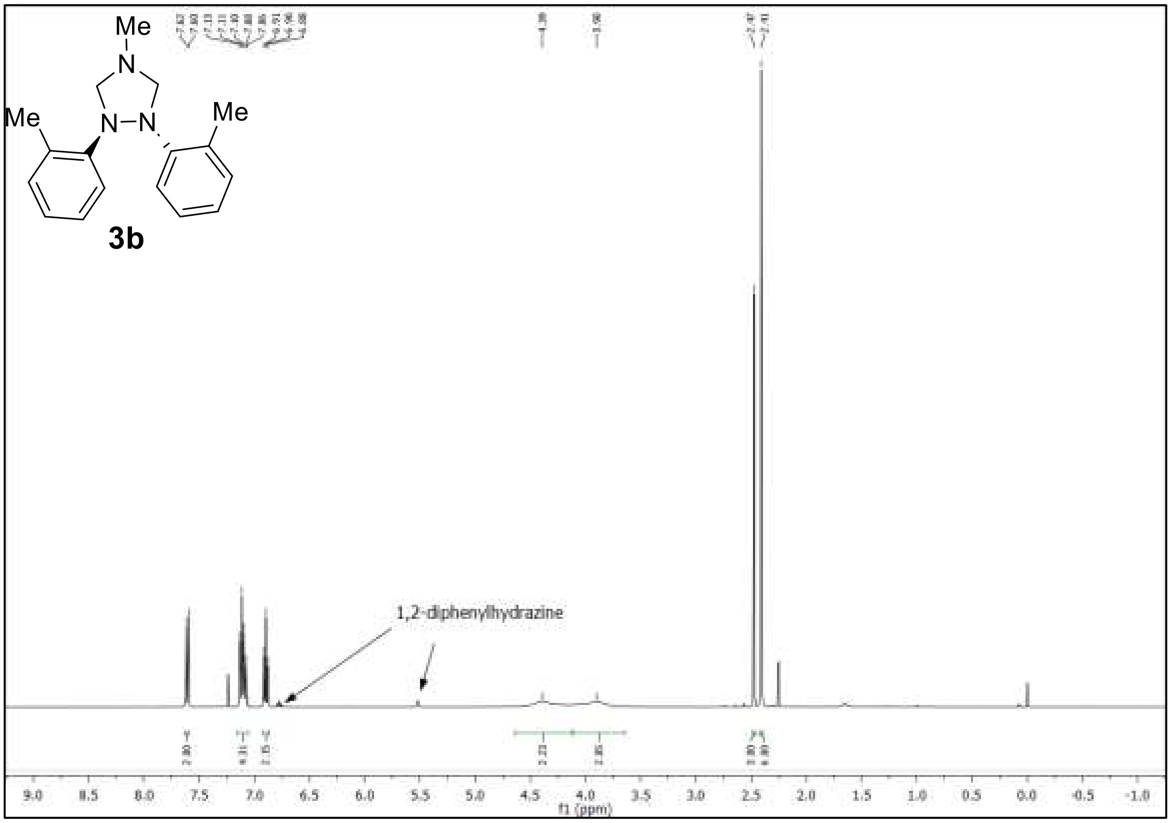


Figure S20: $^{1}H$ NMR spectrum of **3b** (in $CDCl_3$, 400 MHz)

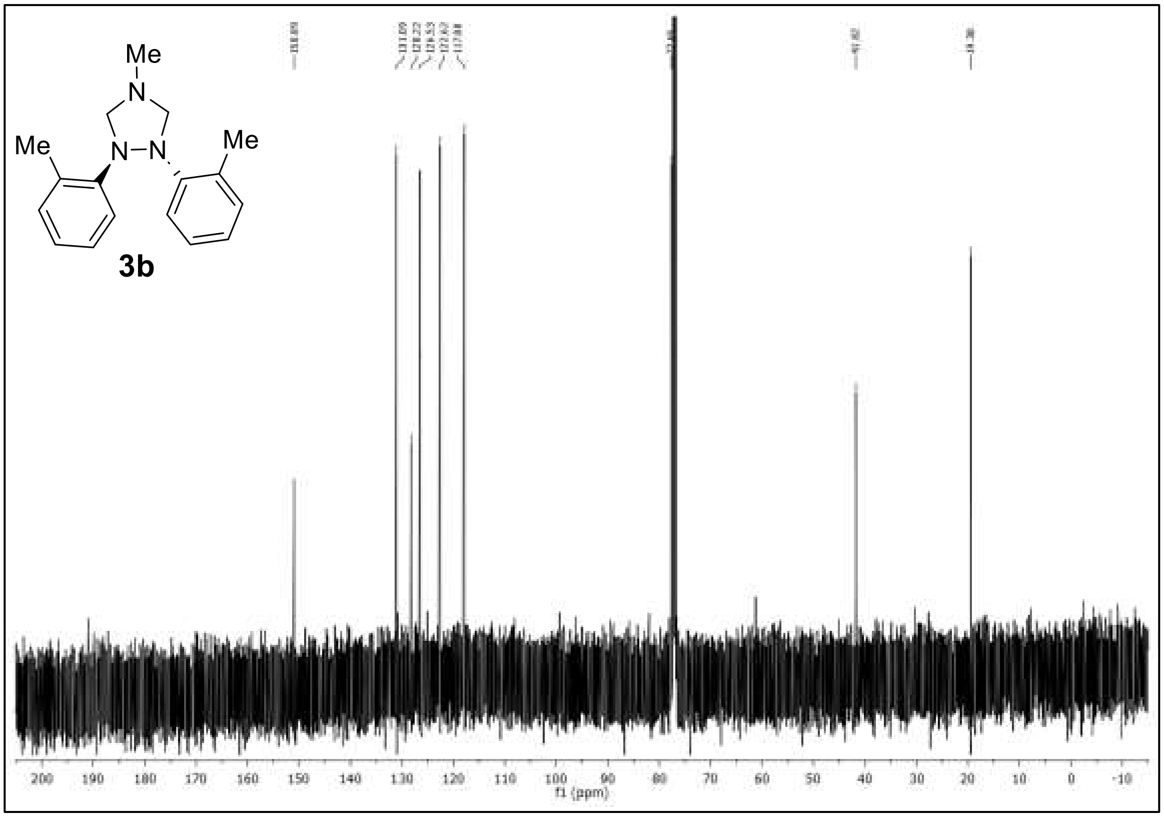


Figure S21: $^{13}C$ {$^{1}H$} NMR spectrum of **3b** (in $CDCl_3$, 126 MHz)

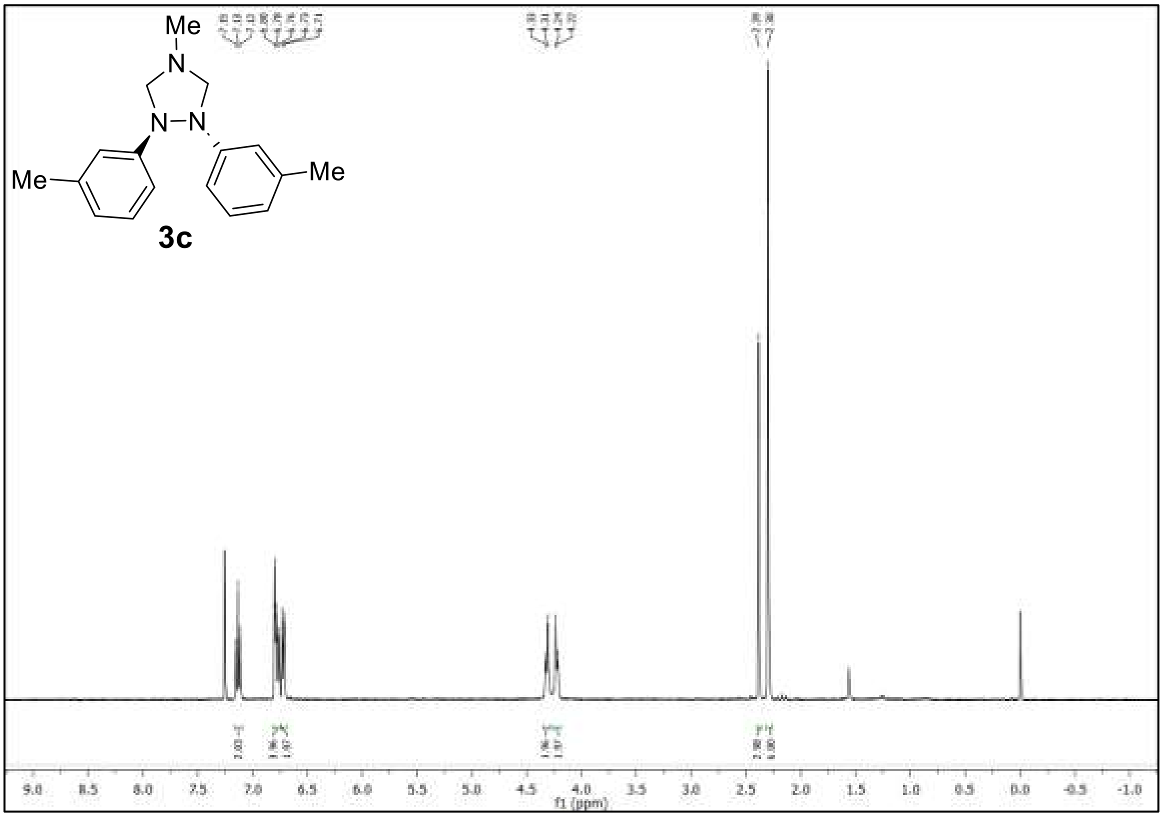

Figure S22: $^{1}H$ NMR spectrum of **3c** (in $CDCl_3$, 400 MHz)

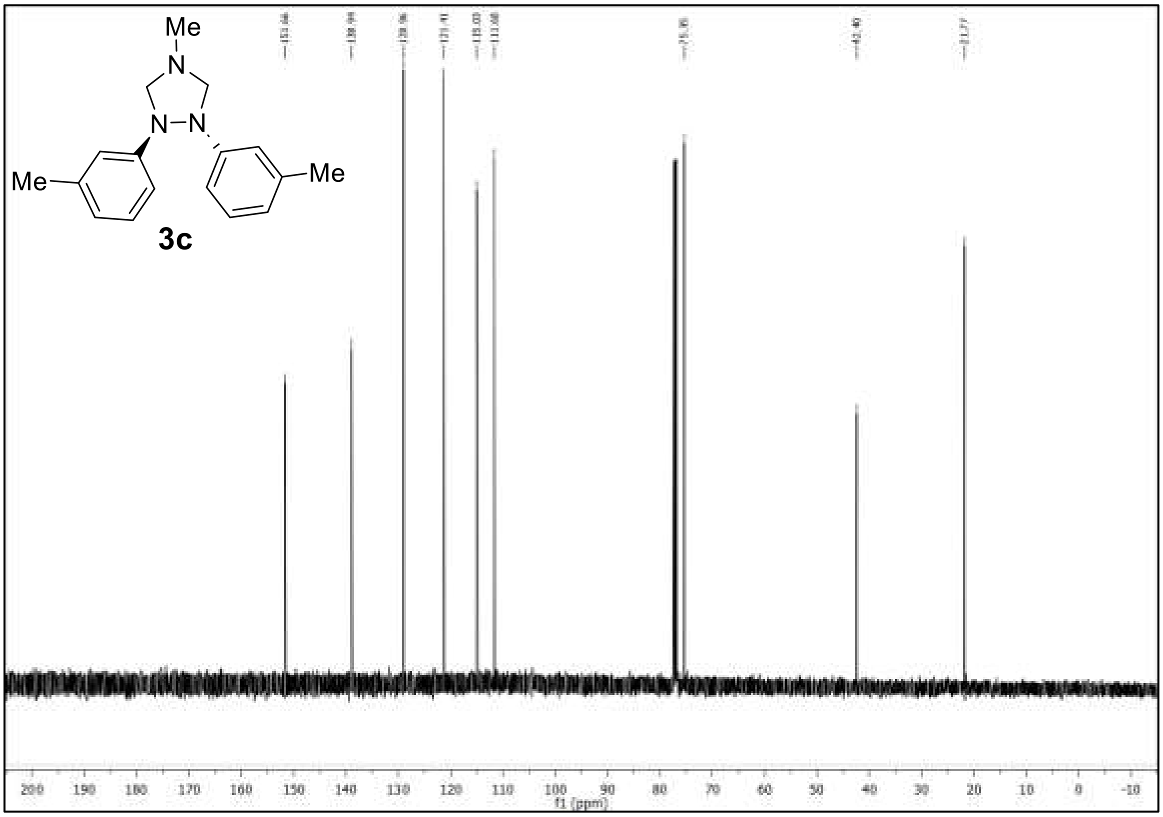

Figure S23: $^{13}C$ {$^{1}H$} NMR spectrum of **3c** (in $CDCl_3$, 126 MHz)

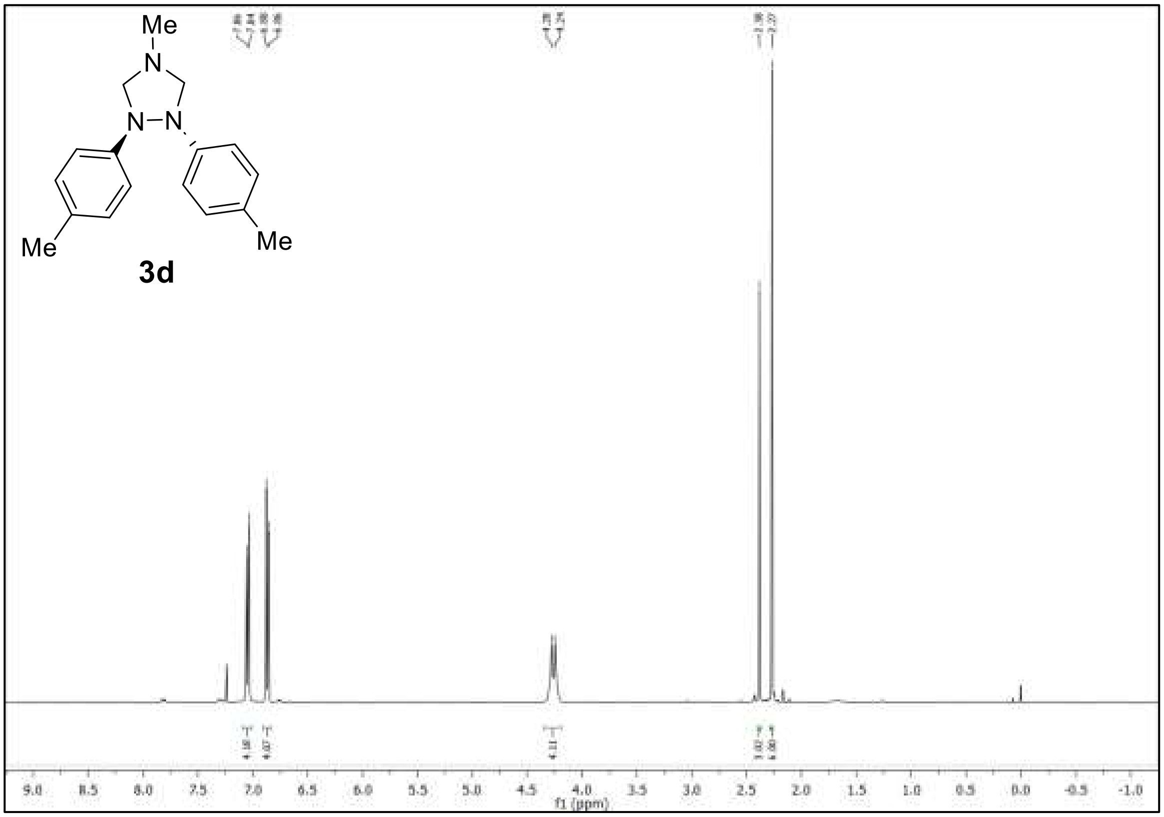

Figure S24: $^{1}H$ NMR spectrum of **3d** (in $CDCl_3$, 400 MHz)

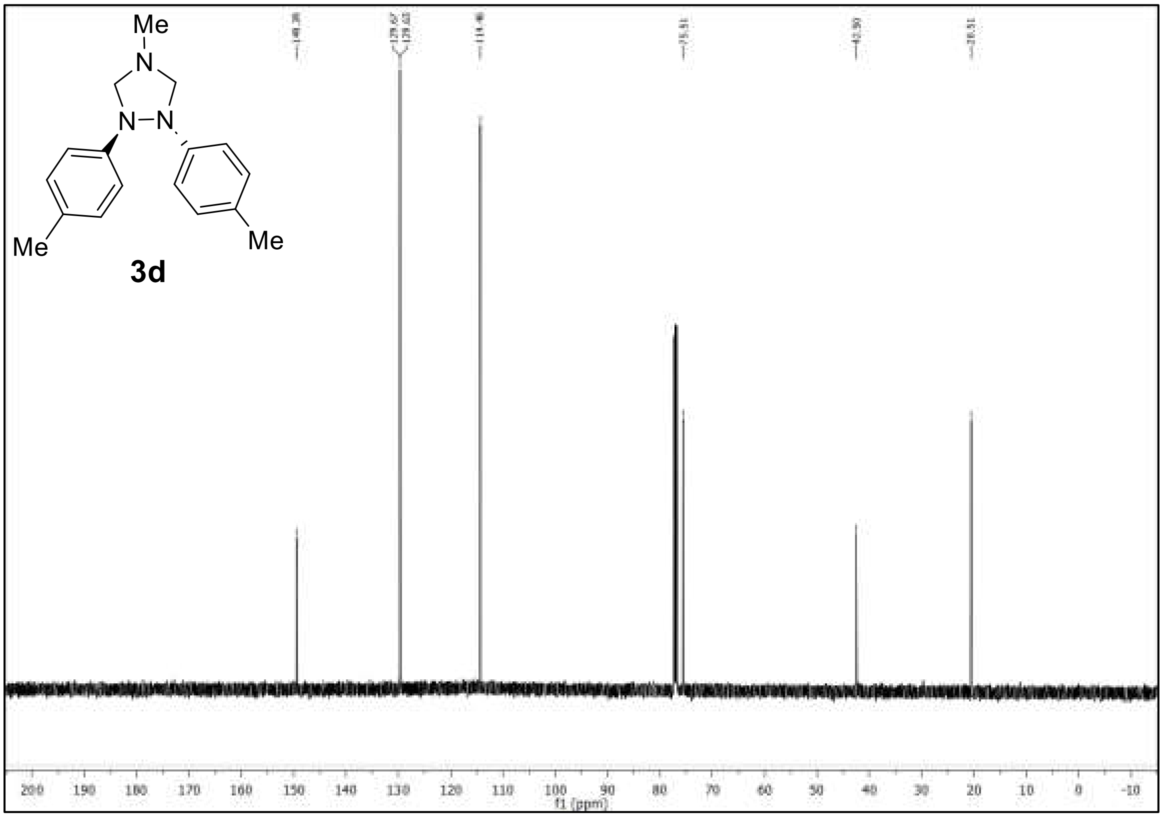

Figure S25: $^{13}C$ {$^{1}H$} NMR spectrum of **3d** (in $CDCl_3$, 126 MHz)

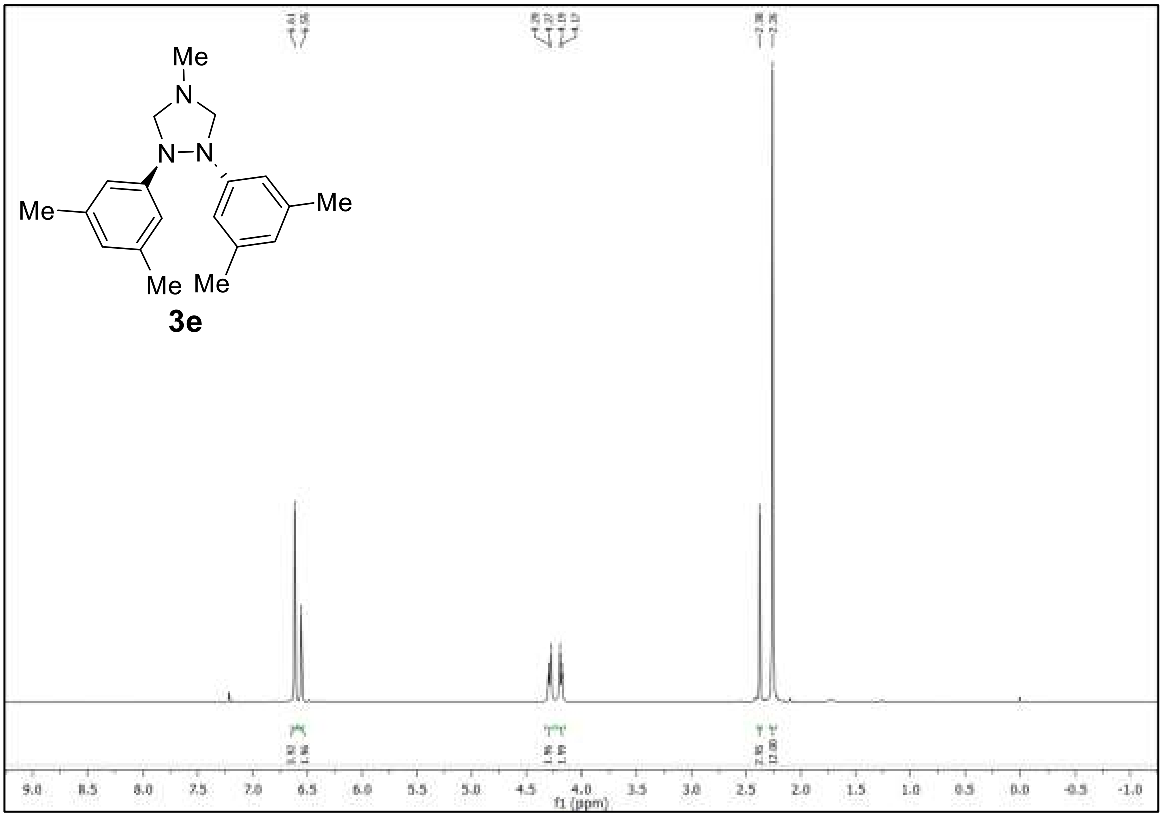


Figure S26: $^1$H NMR spectrum of **3e** (in $CDCl_3$, 400 MHz)

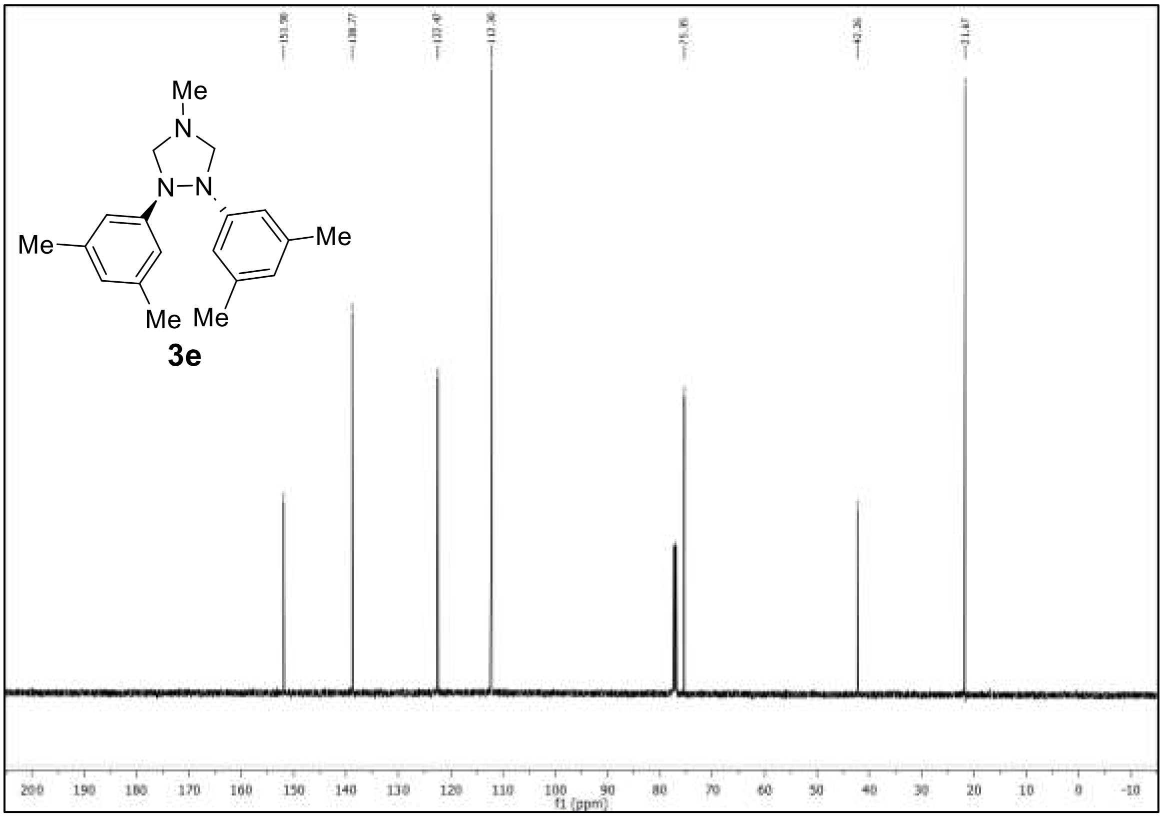


Figure S27: $^{13}$C {$^1$H} NMR spectrum of **3e** (in $CDCl_3$, 126 MHz)

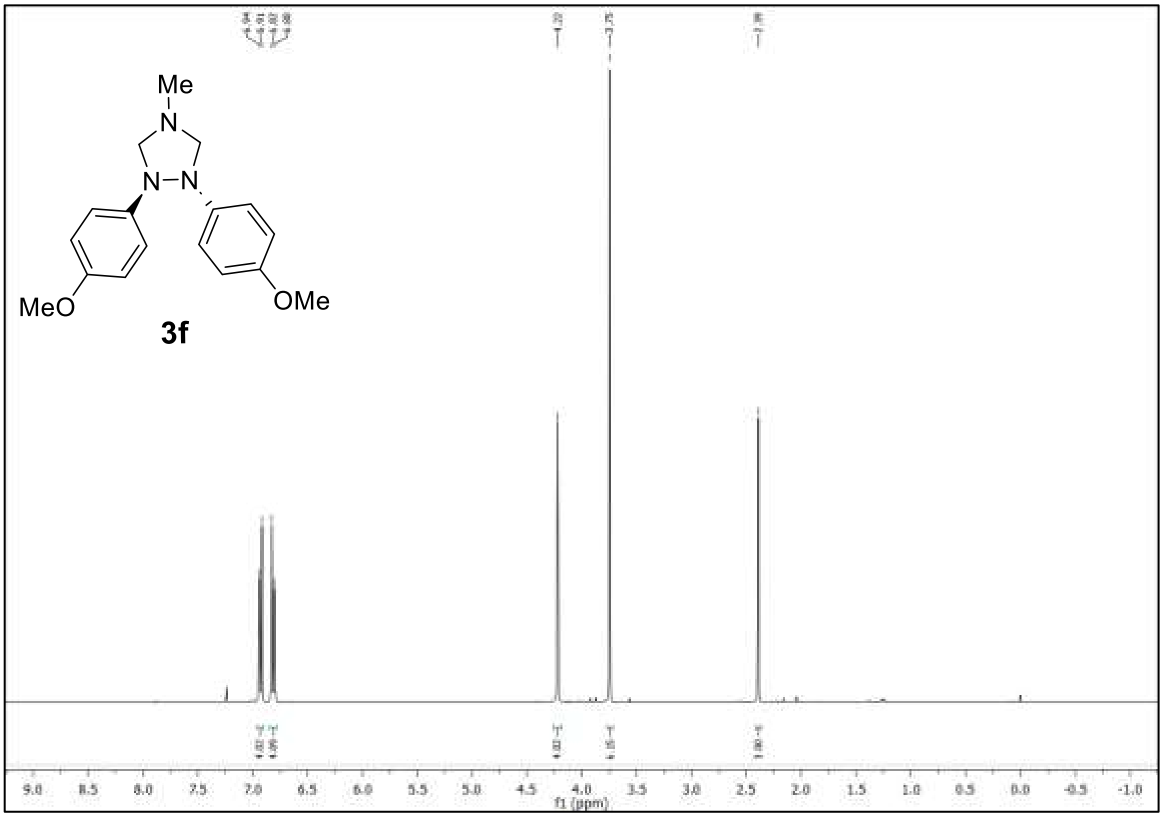


Figure S28: $^{1}H$ NMR spectrum of **3f** (in $CDCl_3$, 400 MHz)

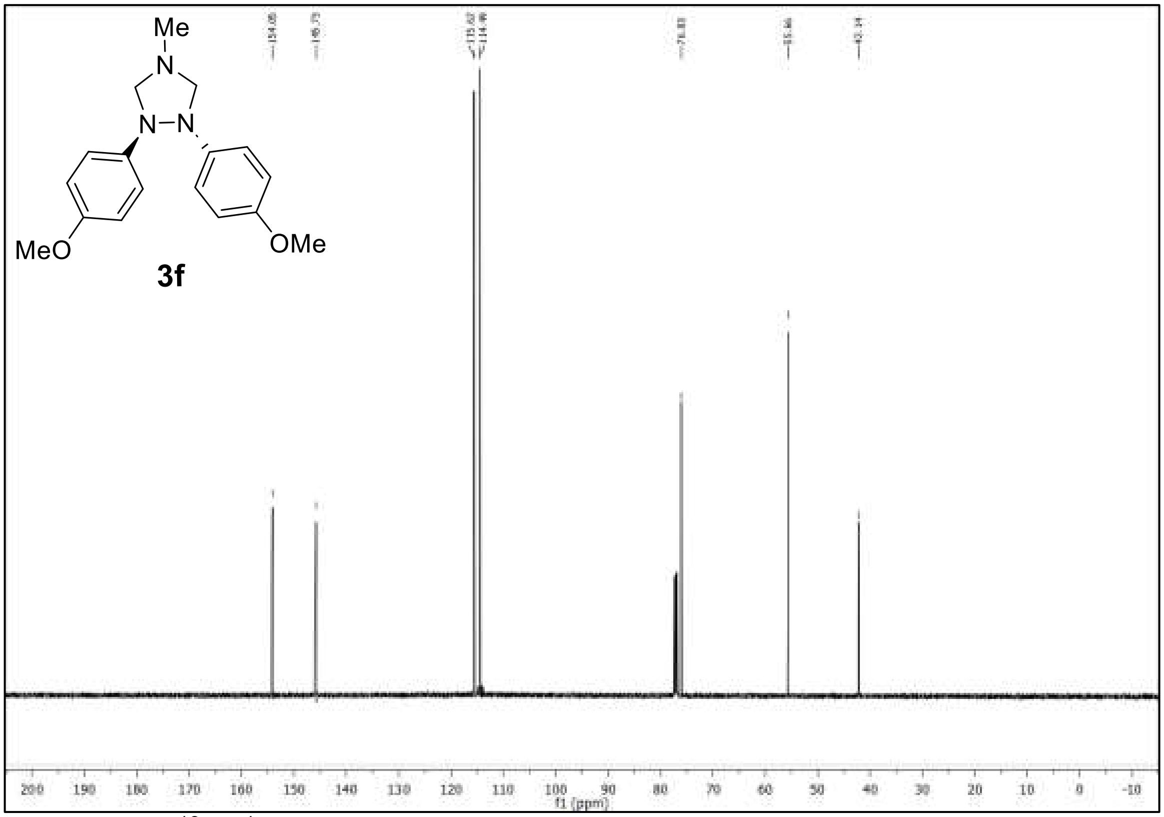


Figure S29: $^{13}C$ {$^{1}H$} NMR spectrum of **3f** (in $CDCl_3$, 126 MHz)

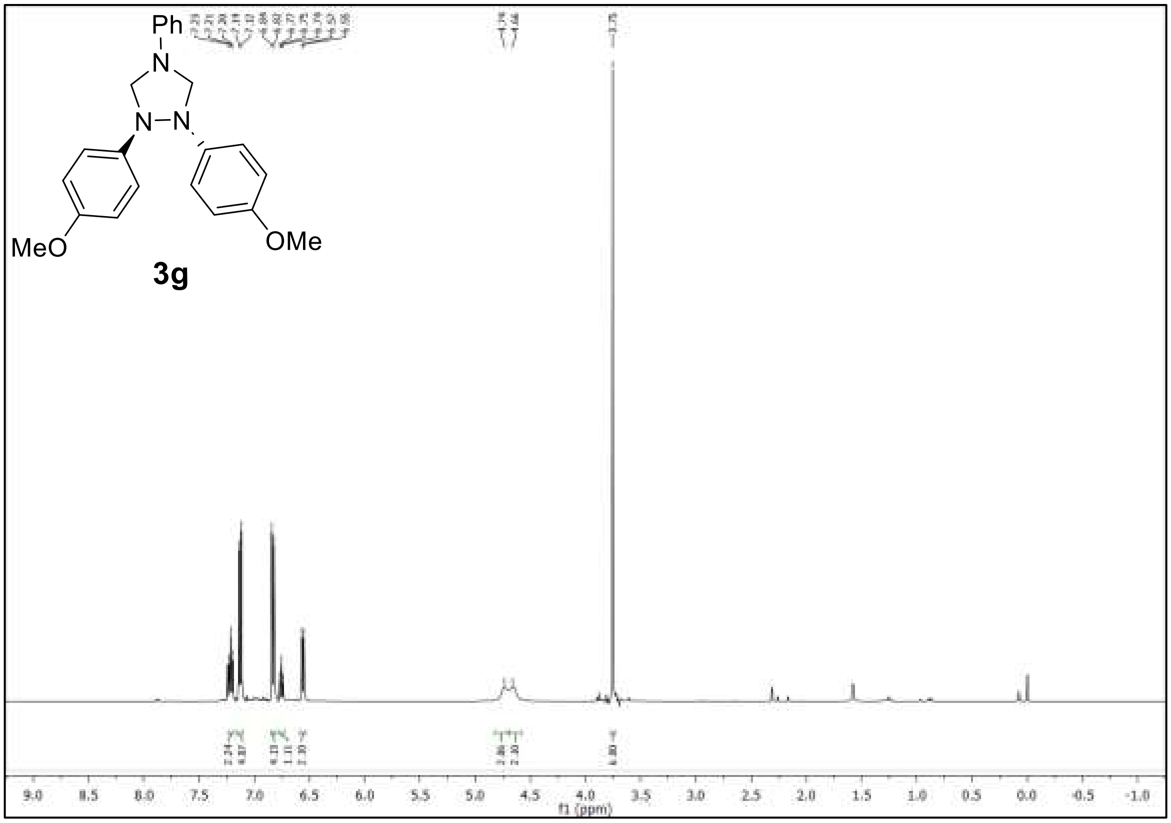

Figure S30: $^1$H NMR spectrum of **3g** (in $CDCl_3$, 500 MHz)

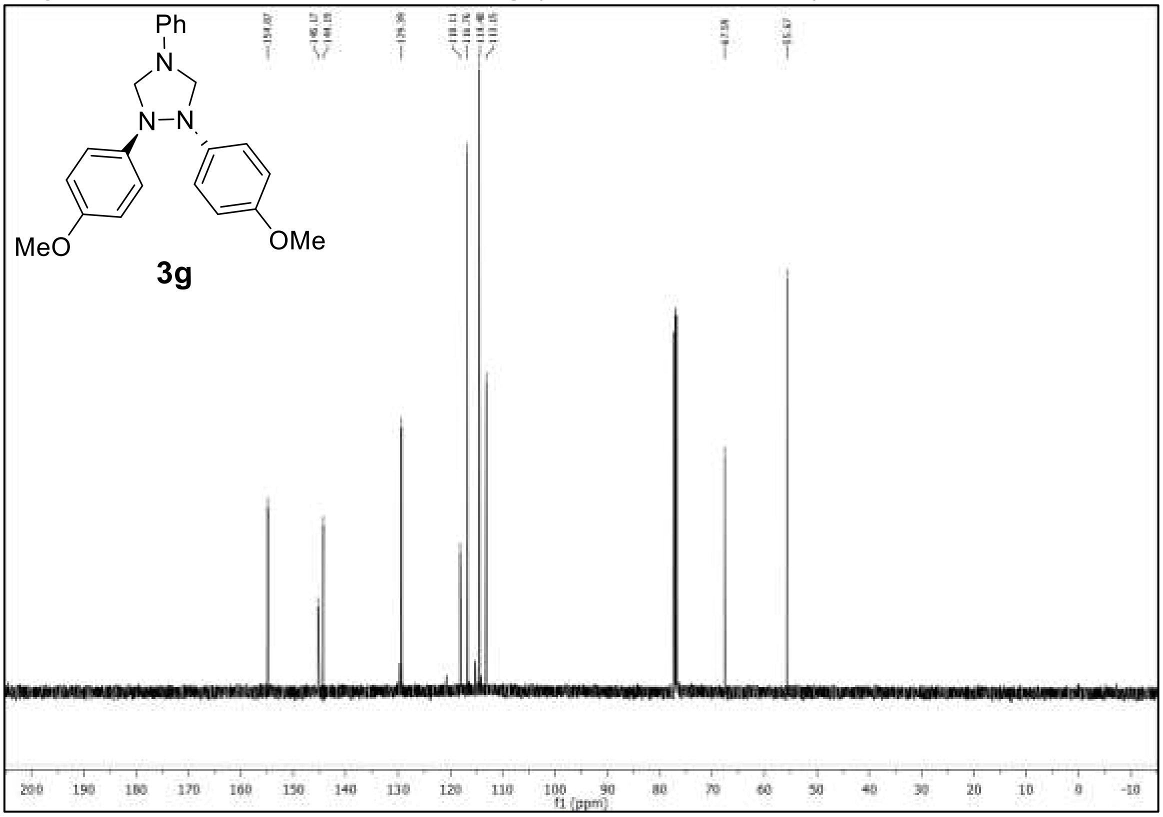

Figure S31: $^{13}C$ {$^1$H} NMR spectrum of **3g** (in $CDCl_3$, 126 MHz)

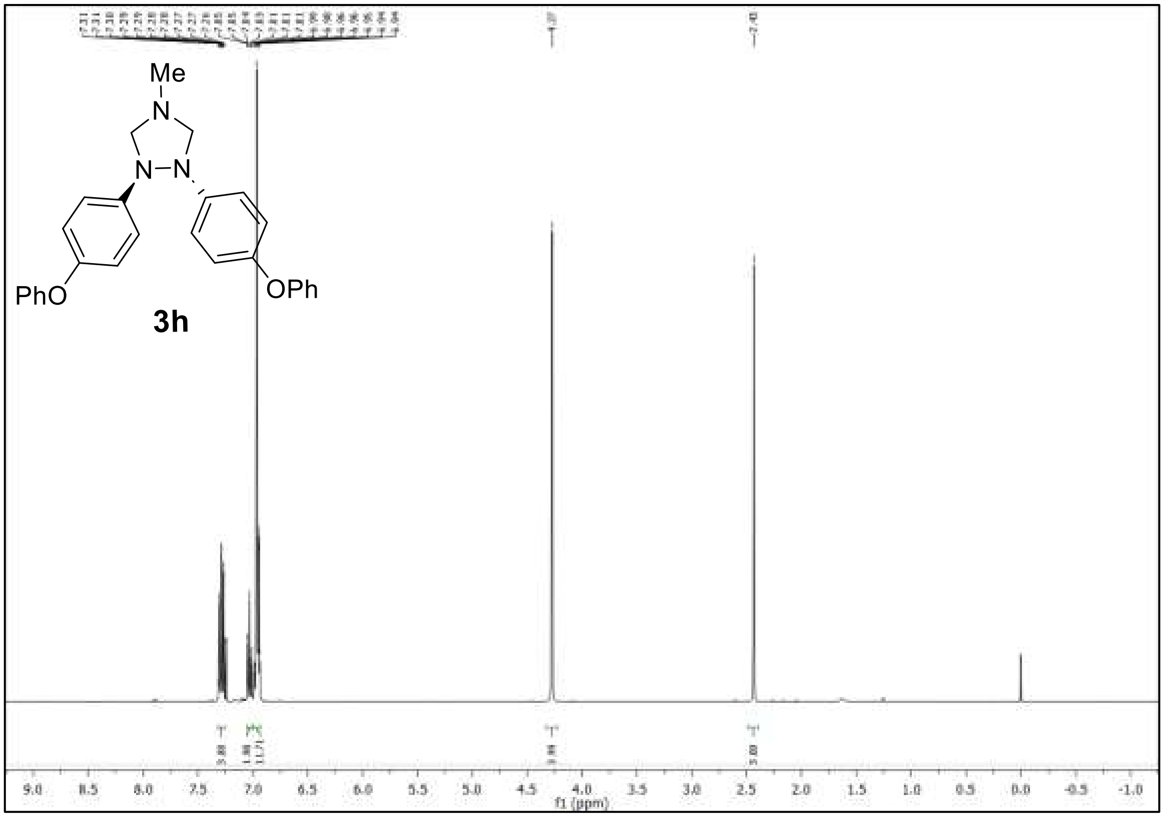

Figure S32: $^{1}H$ NMR spectrum of **3h** (in $CDCl_3$, 400 MHz)

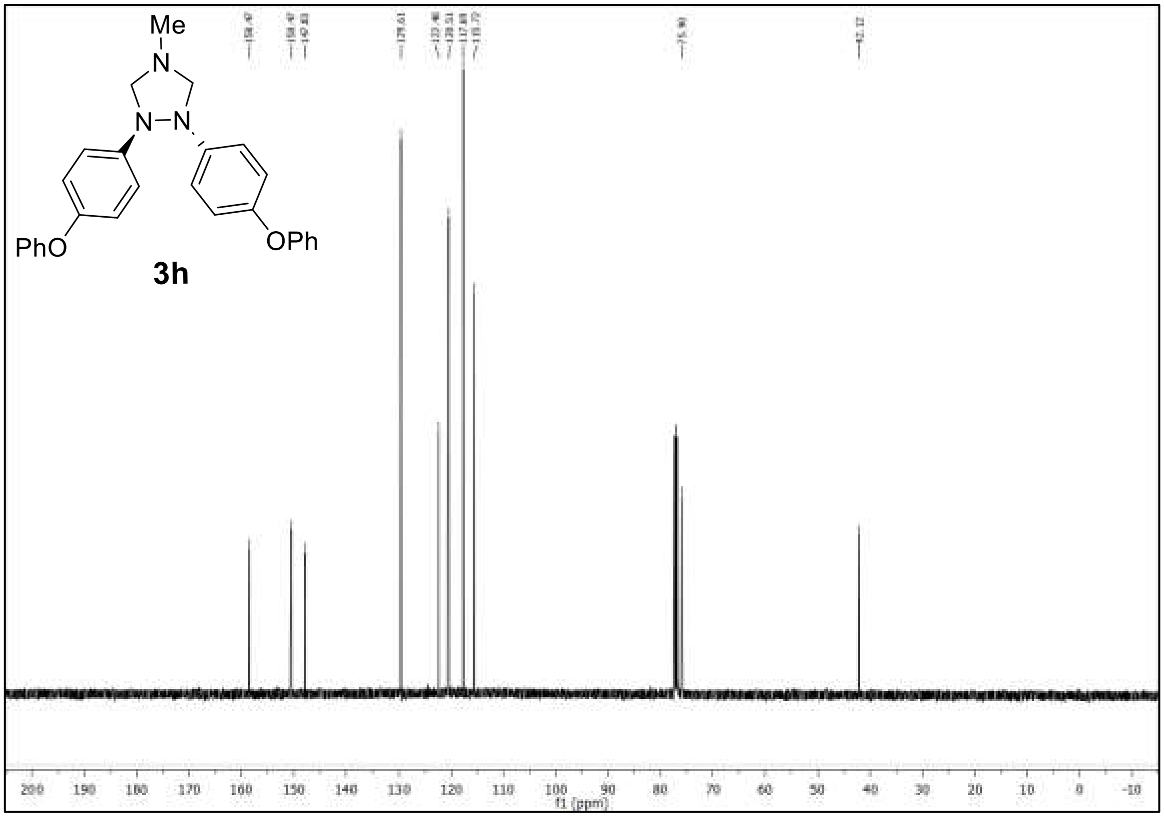

Figure S33: $^{13}C\ \{^{1}H\}$ NMR spectrum of **3h** (in $CDCl_3$, 126 MHz)

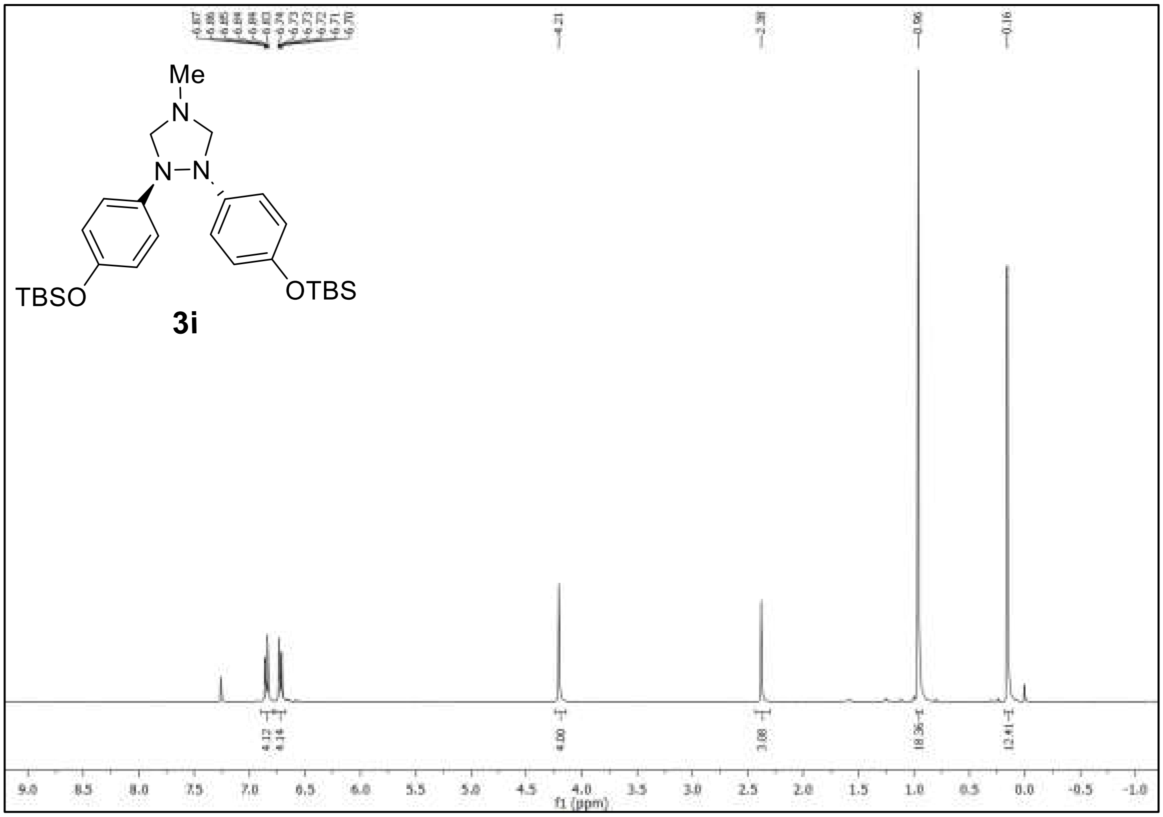


Figure S34: $^{1}$H NMR spectrum of **3i** (in $CDCl_3$, 400 MHz)

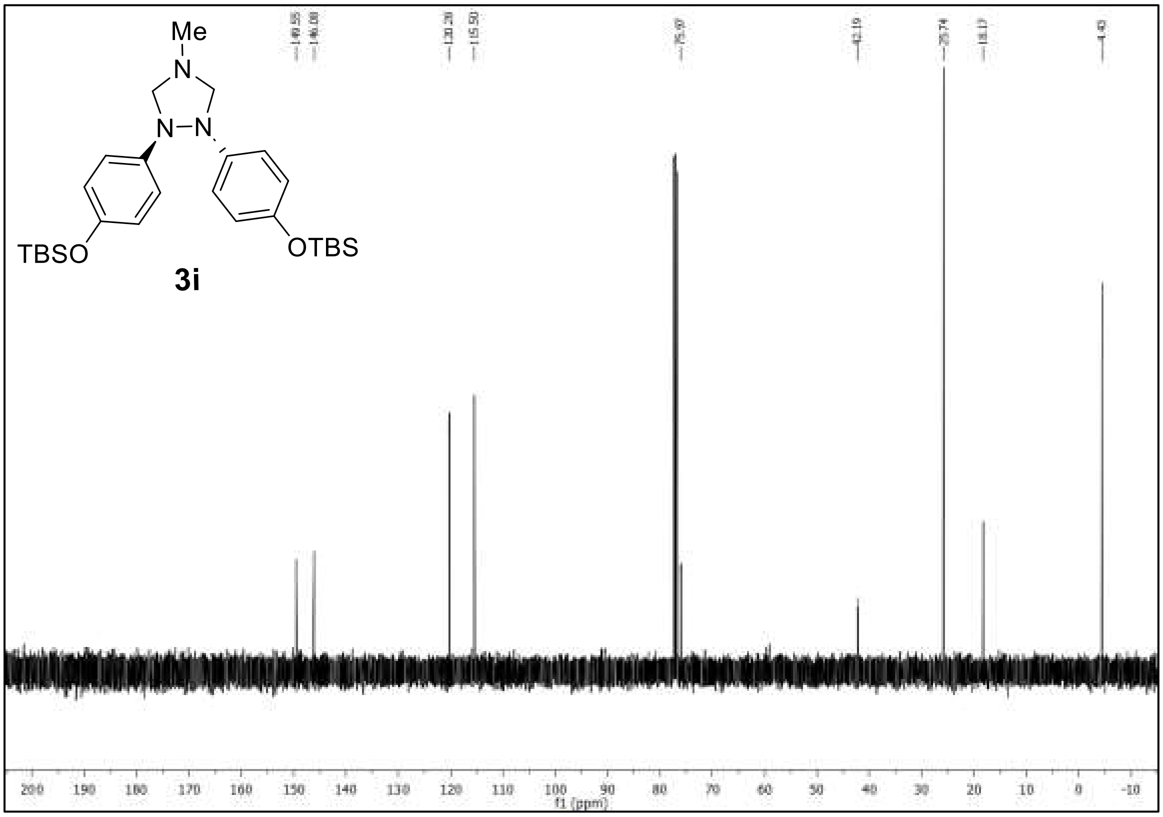


Figure S35: $^{13}$C {$^{1}$H} NMR spectrum of **3i** (in $CDCl_3$, 126 MHz)

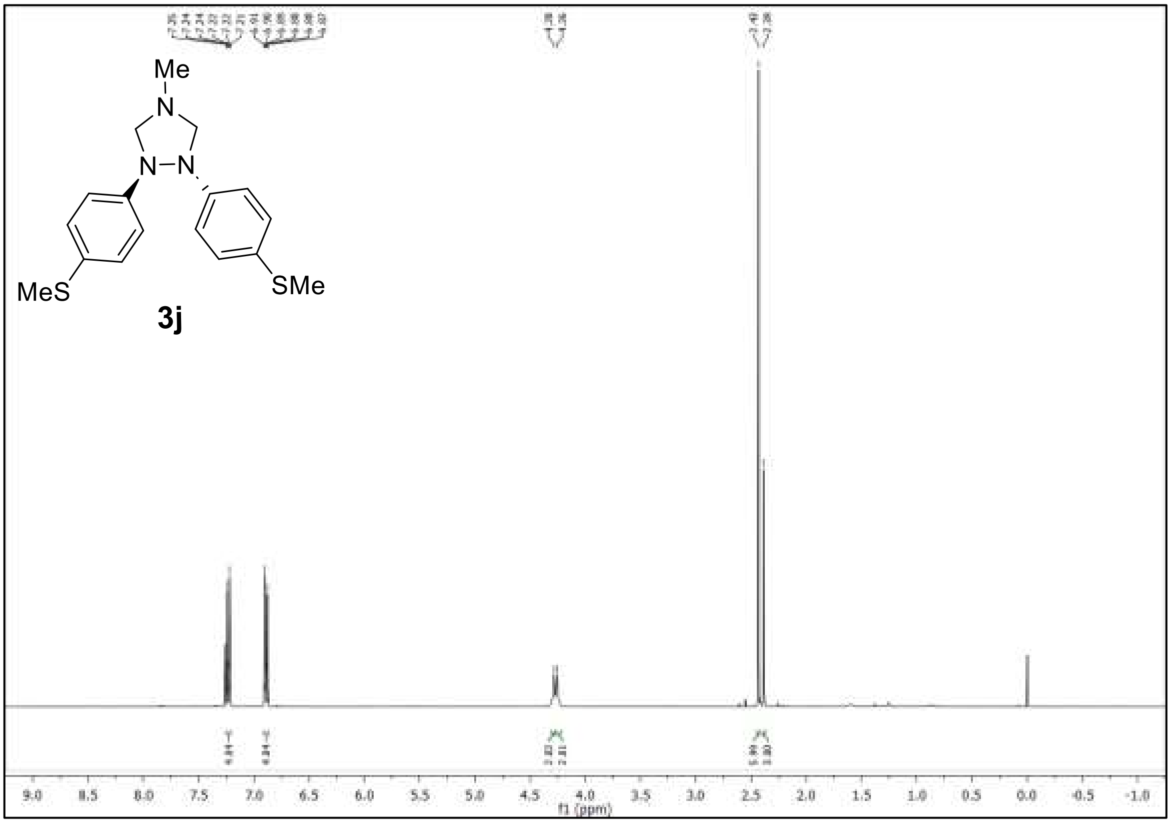

Figure S36: $^{1}H$ NMR spectrum of **3j** (in $CDCl_3$, 400 MHz)

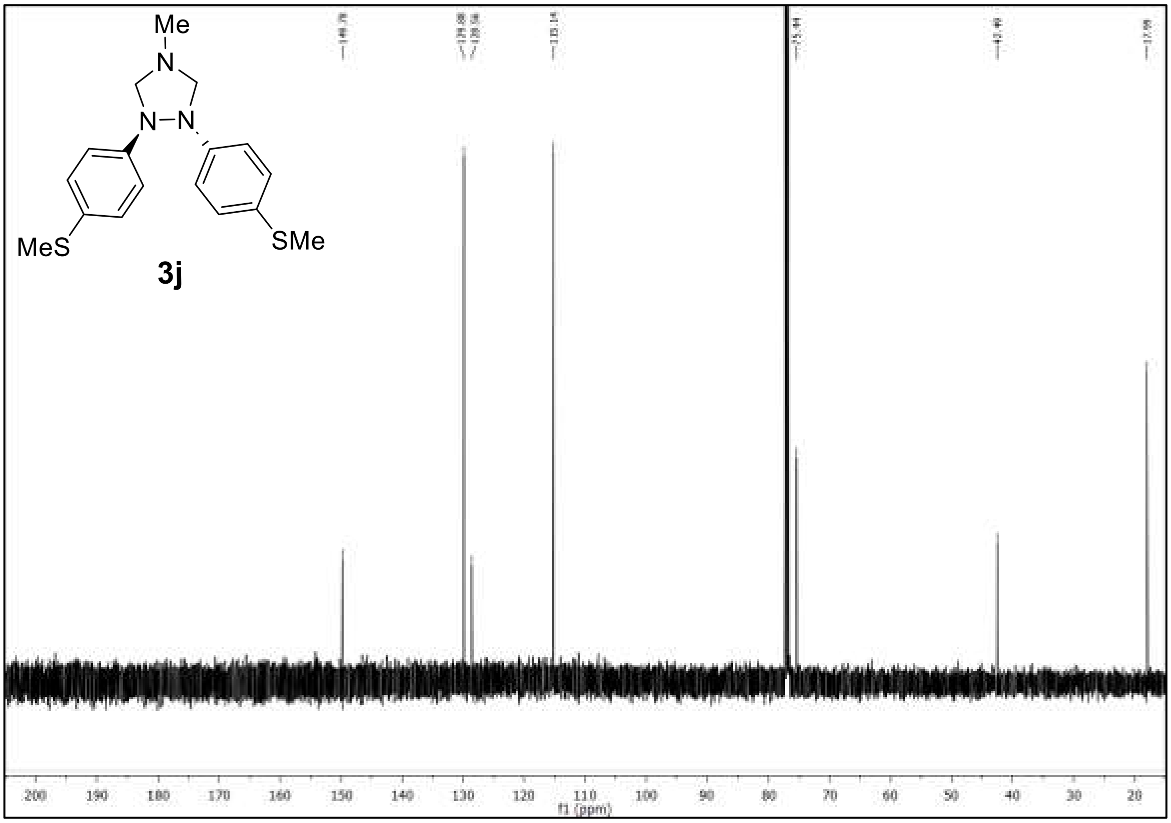

Figure S37: $^{13}C$ {$^{1}H$} NMR spectrum of **3j** (in $CDCl_3$, 126 MHz)

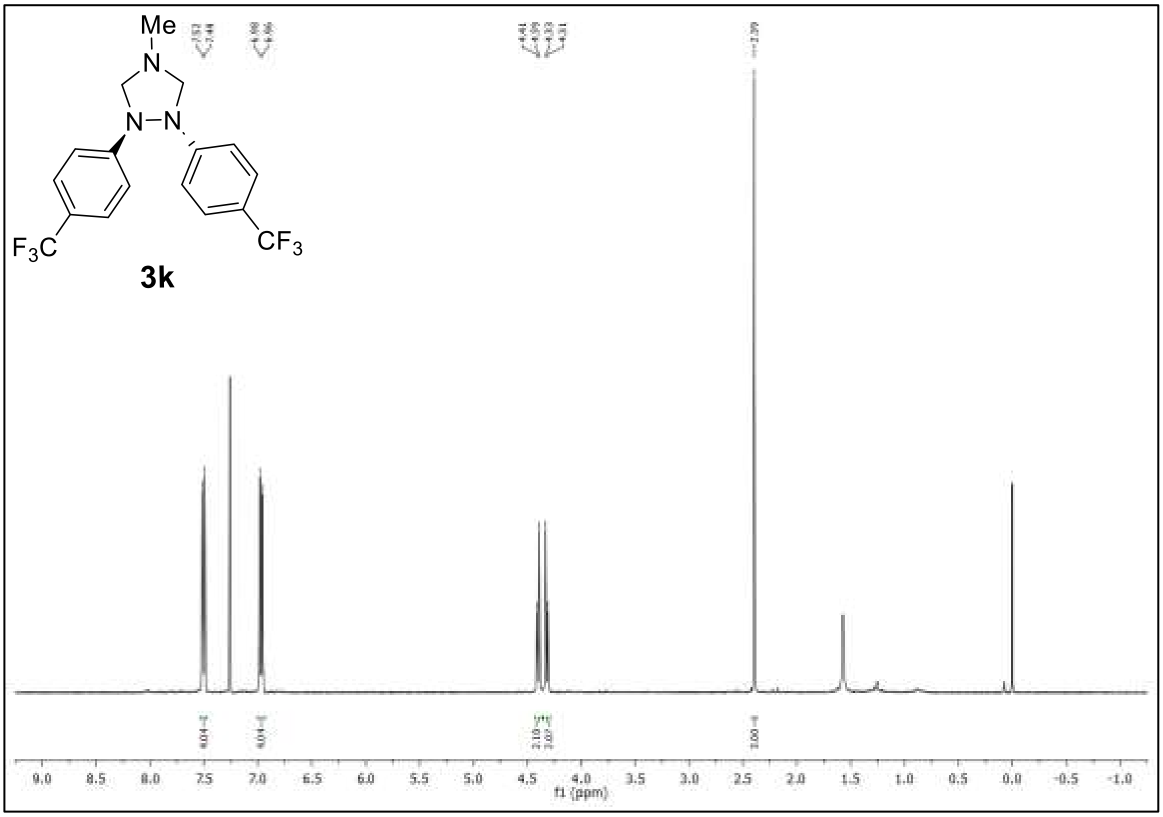


Figure S38: $^{1}H$ NMR spectrum of **3k** (in $CDCl_3$, 400 MHz)

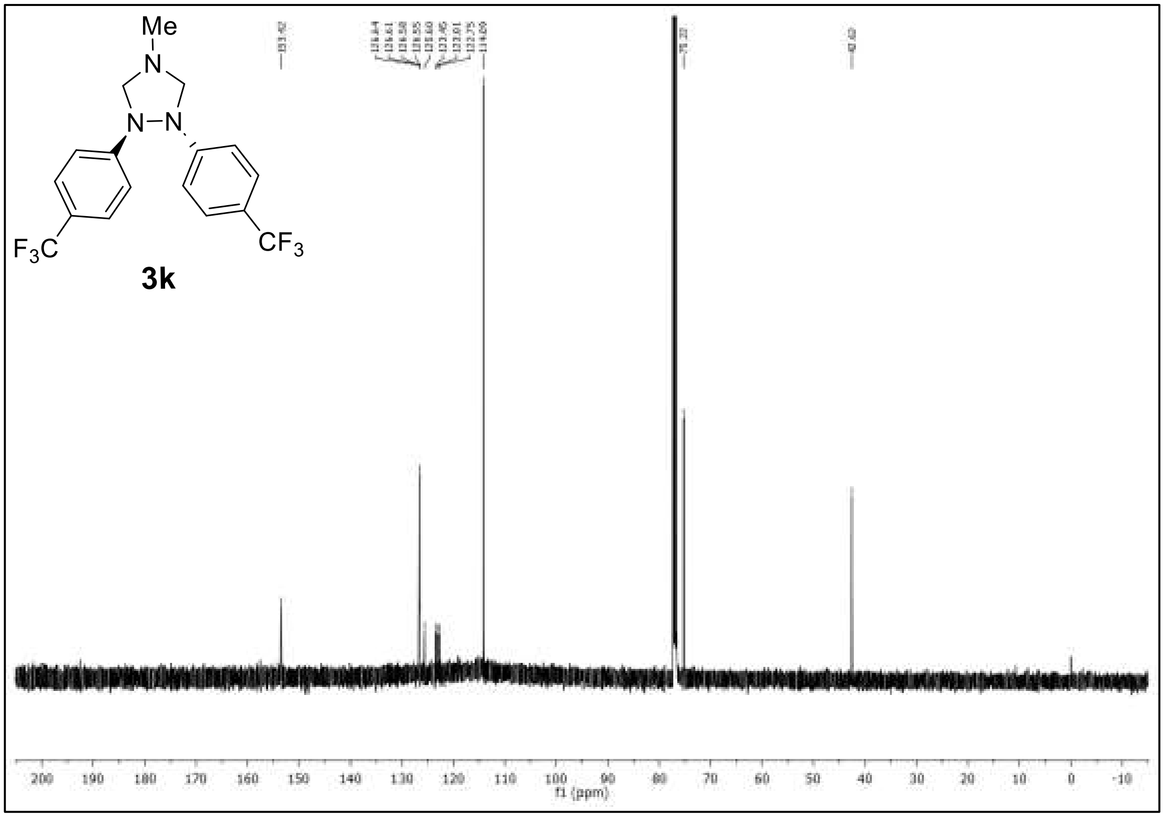


Figure S39: $^{13}C$ {$^{1}H$} NMR spectrum of **3k** (in $CDCl_3$, 126 MHz)

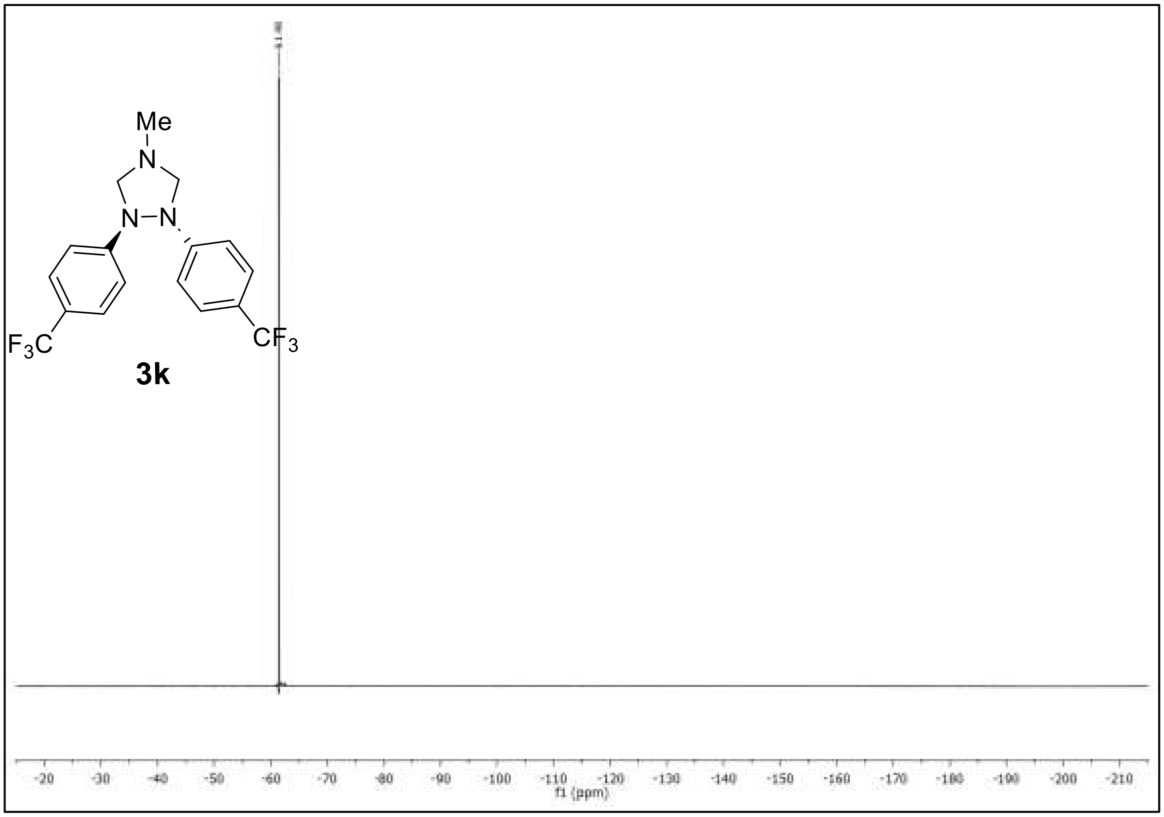


Figure S40: $^{19}F$ NMR spectrum of **3k** (in $CDCl_3$, 376 MHz)

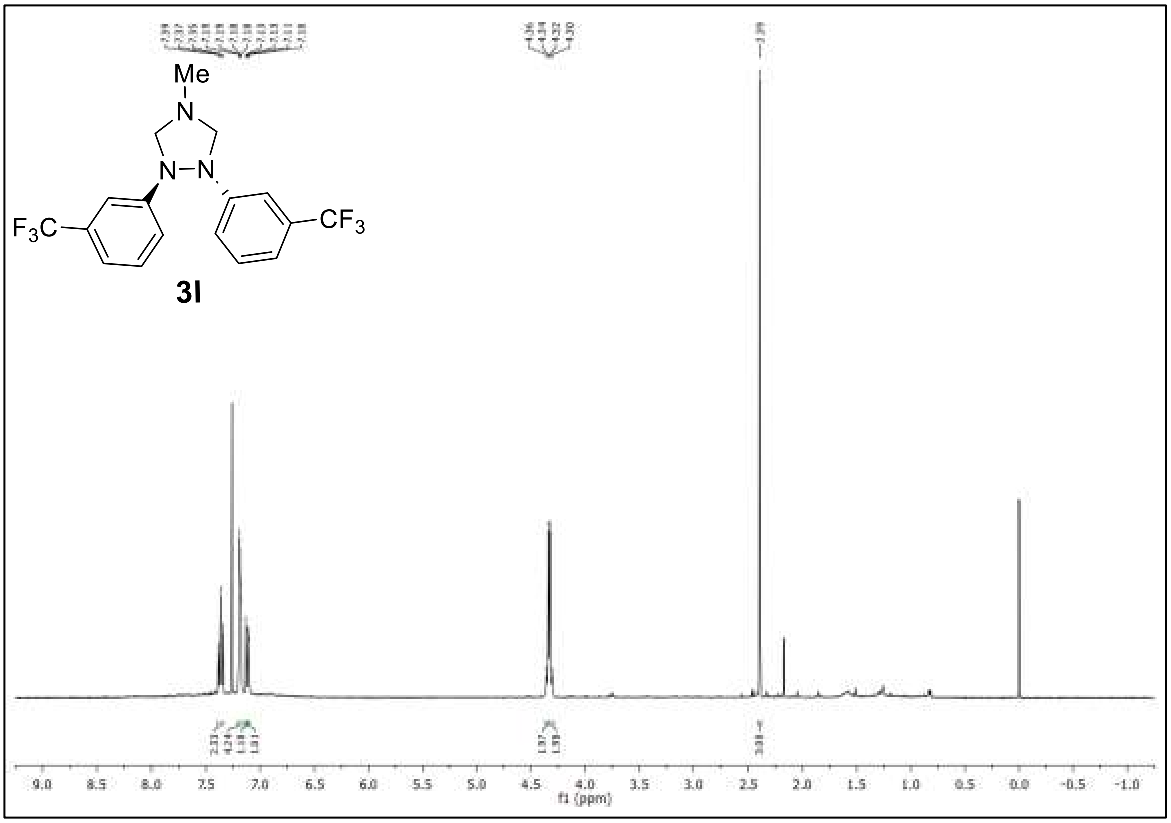

Figure S41: $^{1}H$ NMR spectrum of **3l** (in $CDCl_3$, 400 MHz)

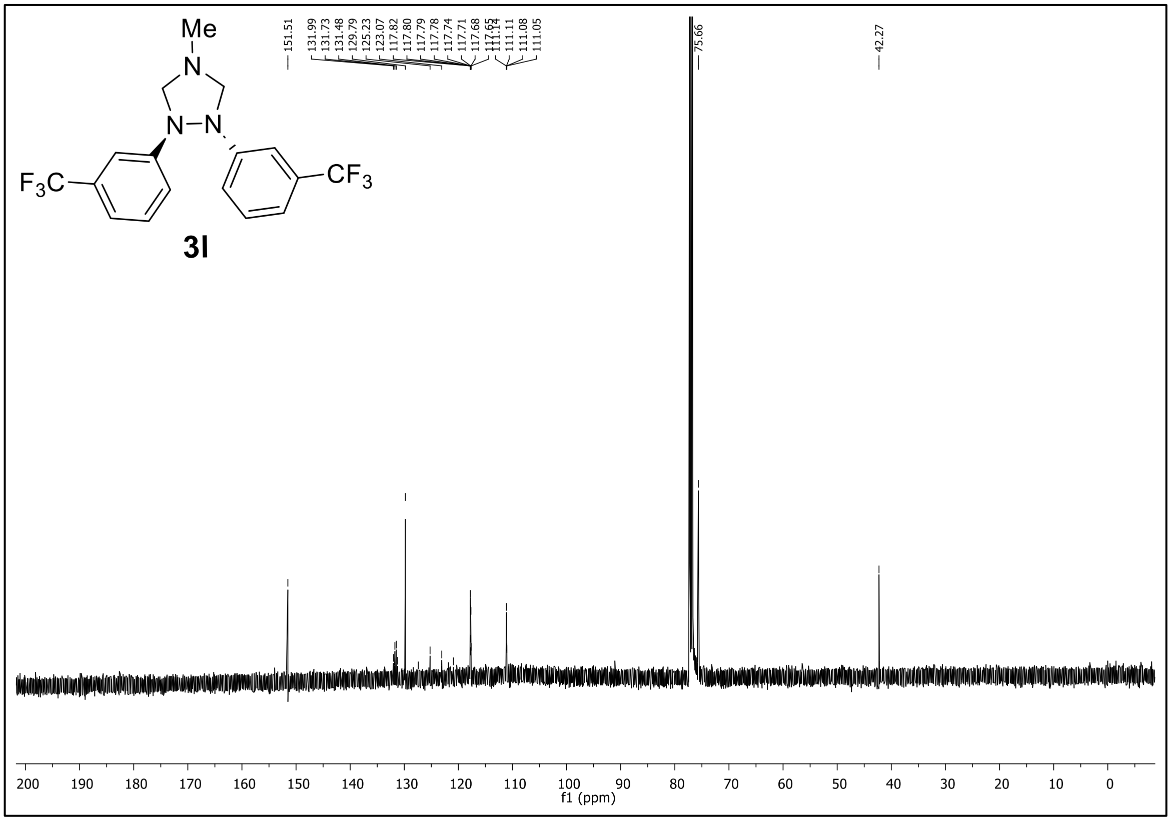

Figure 42: $^{13}C\ \{^{1}H\}$ NMR spectrum of **3l** (in $CDCl_3$, 126 MHz)

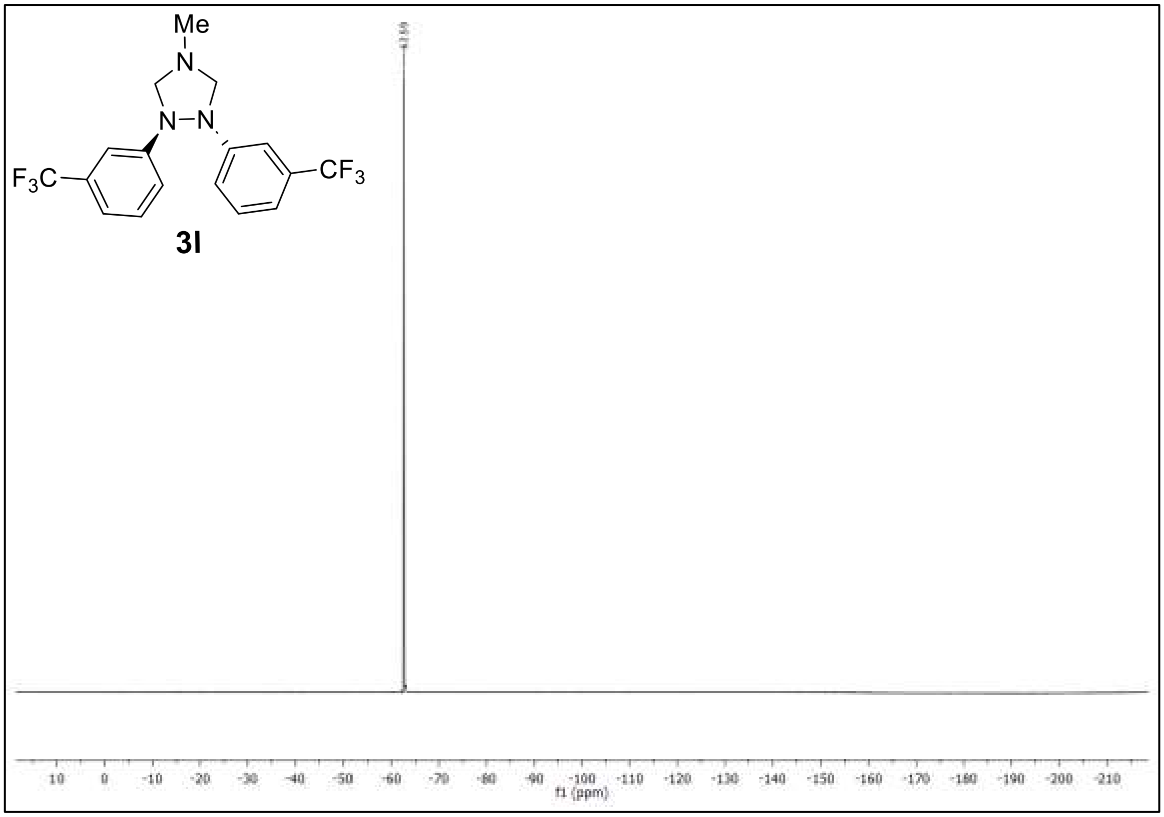


Figure S43: $^{19}$F NMR spectrum of **3l** (in $CDCl_3$, 376 MHz)

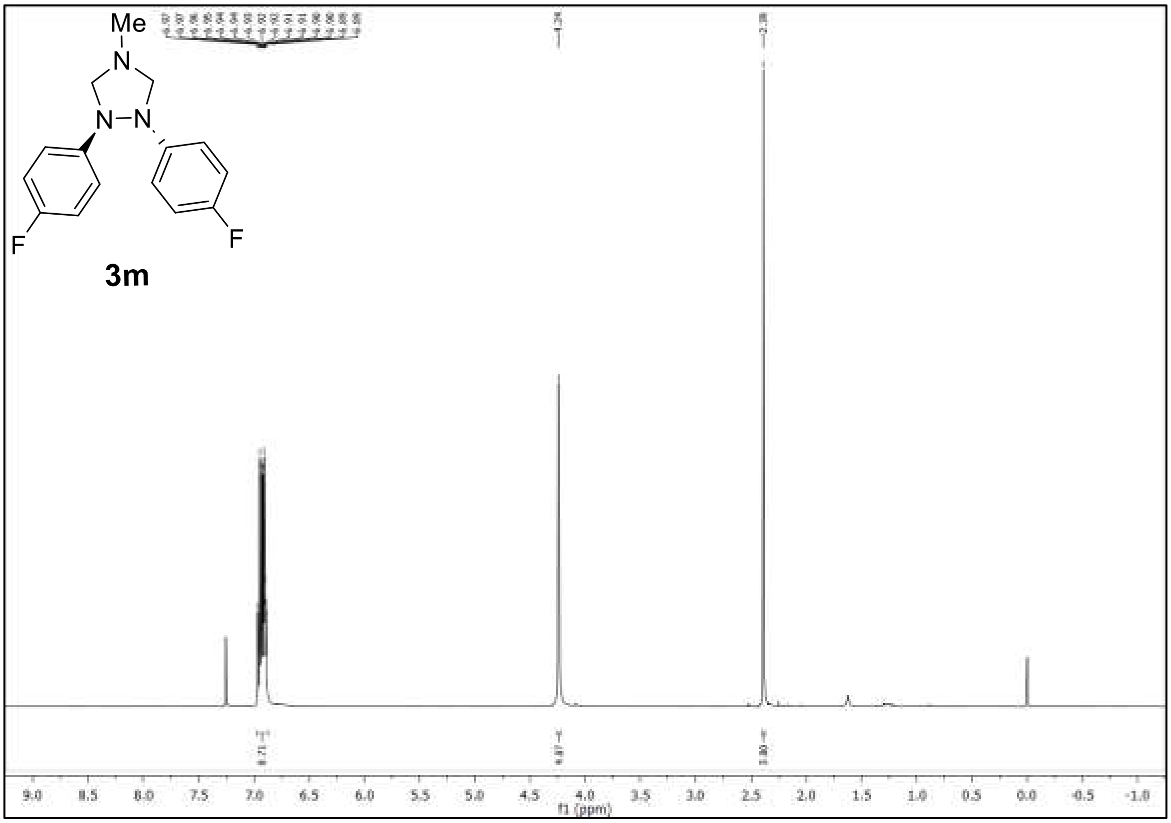


Figure S44: $^{1}$H NMR spectrum of **3m** (in $CDCl_3$, 500 MHz)

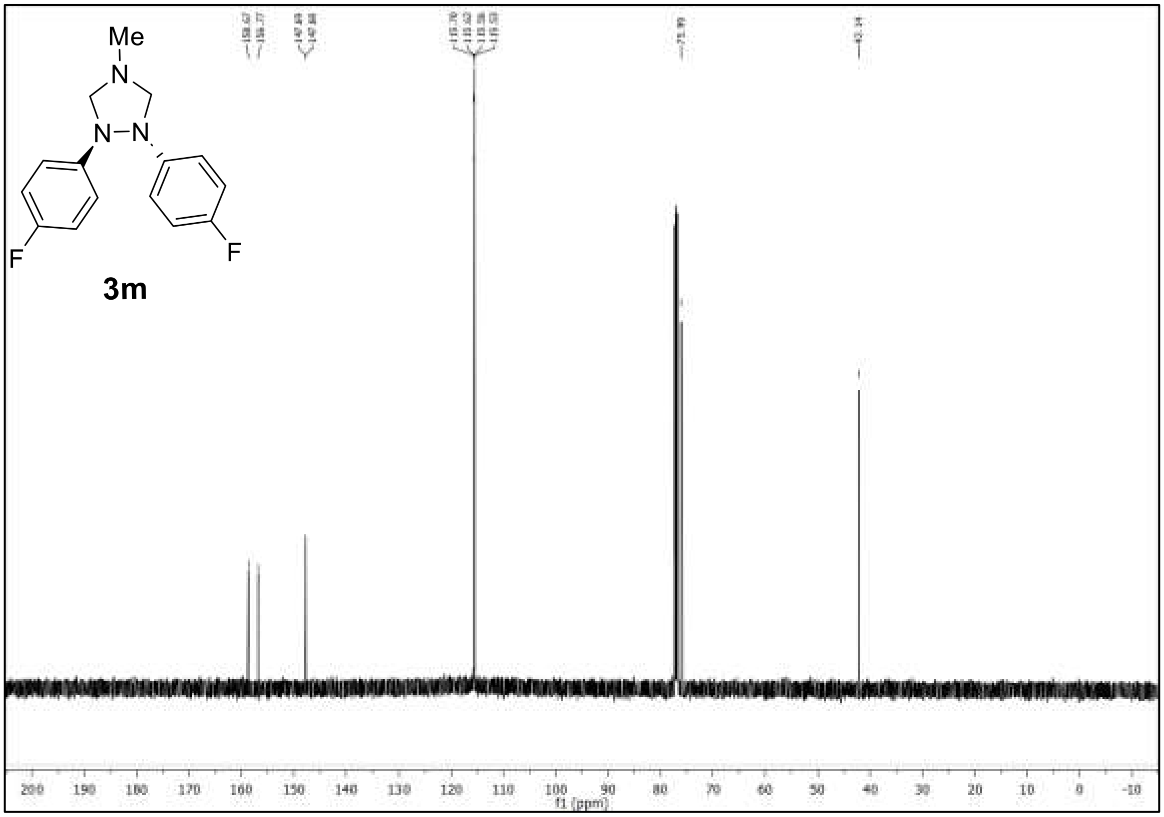


Figure S45: $^{13}$C {$^{1}$H} NMR spectrum of **3m** (in $CDCl_3$, 126 MHz)

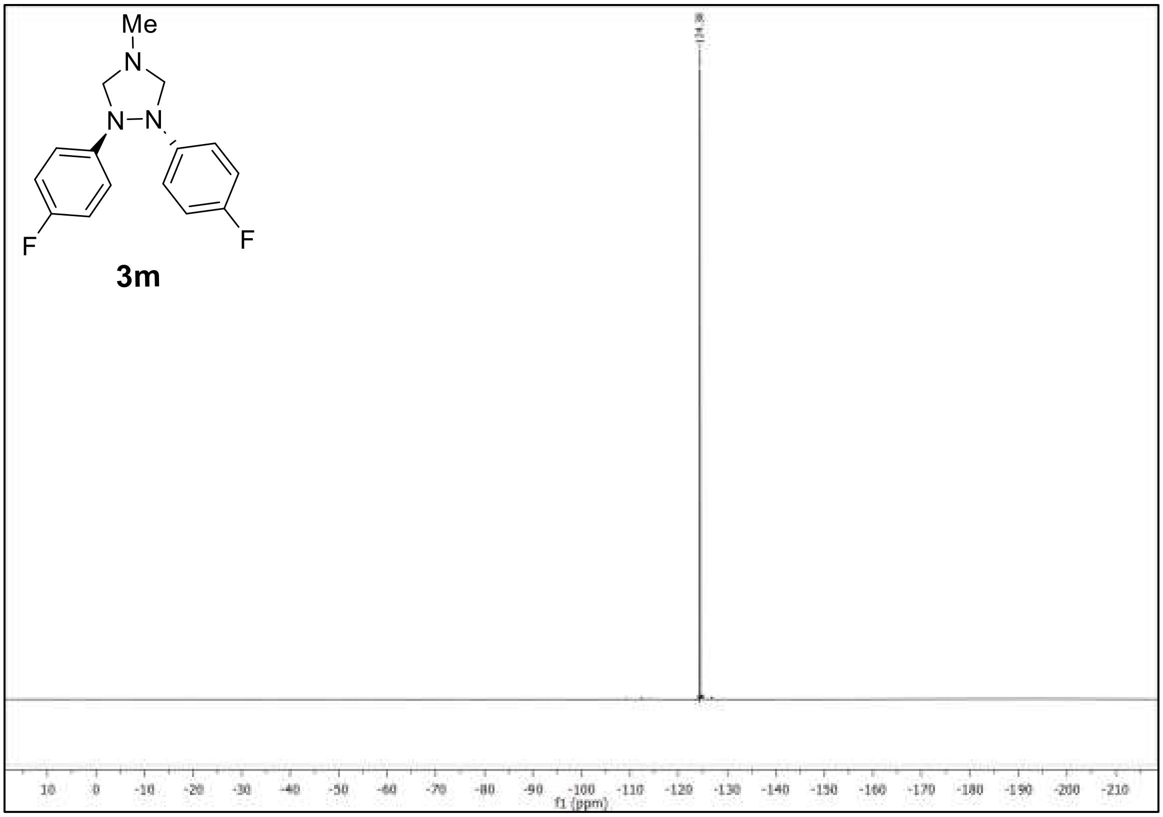

Figure S46: $^{19}$F NMR spectrum of **3m** (in $CDCl_3$, 376 MHz)

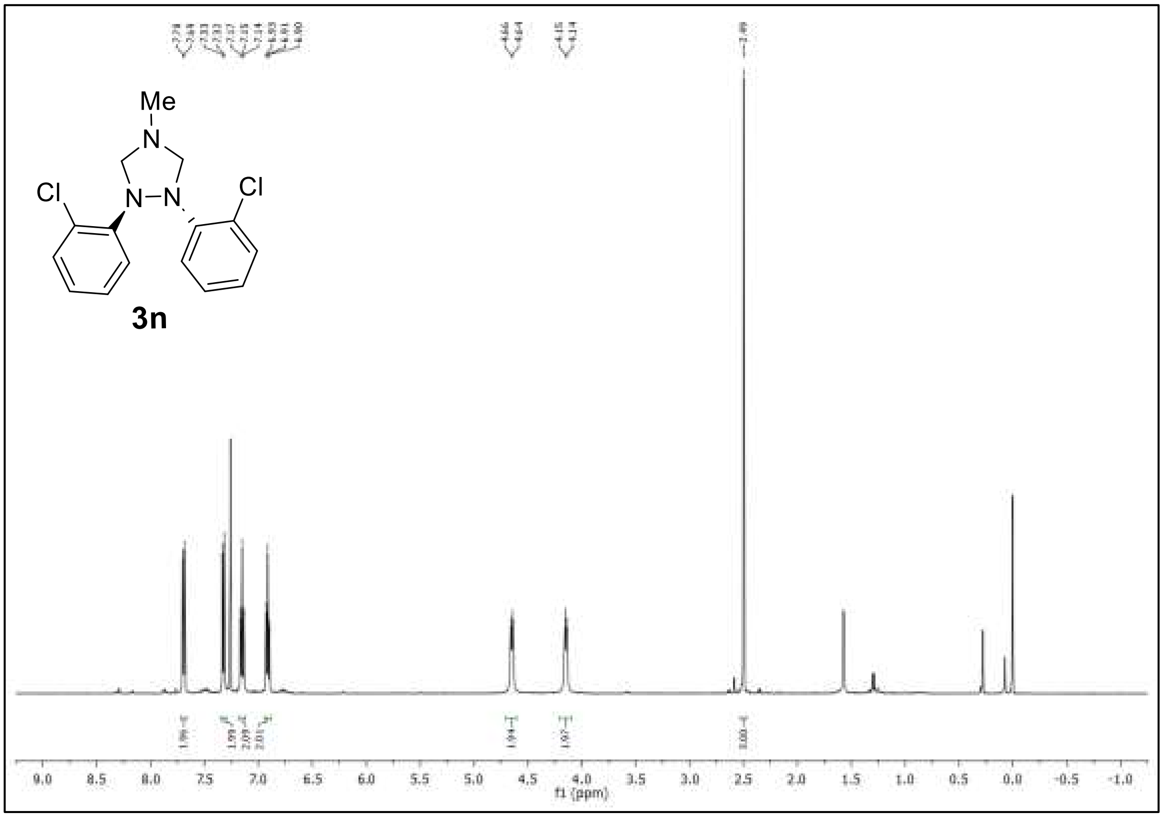


Figure S47: $^{1}H$ NMR spectrum of **3n** (in $CDCl_3$, 500 MHz)

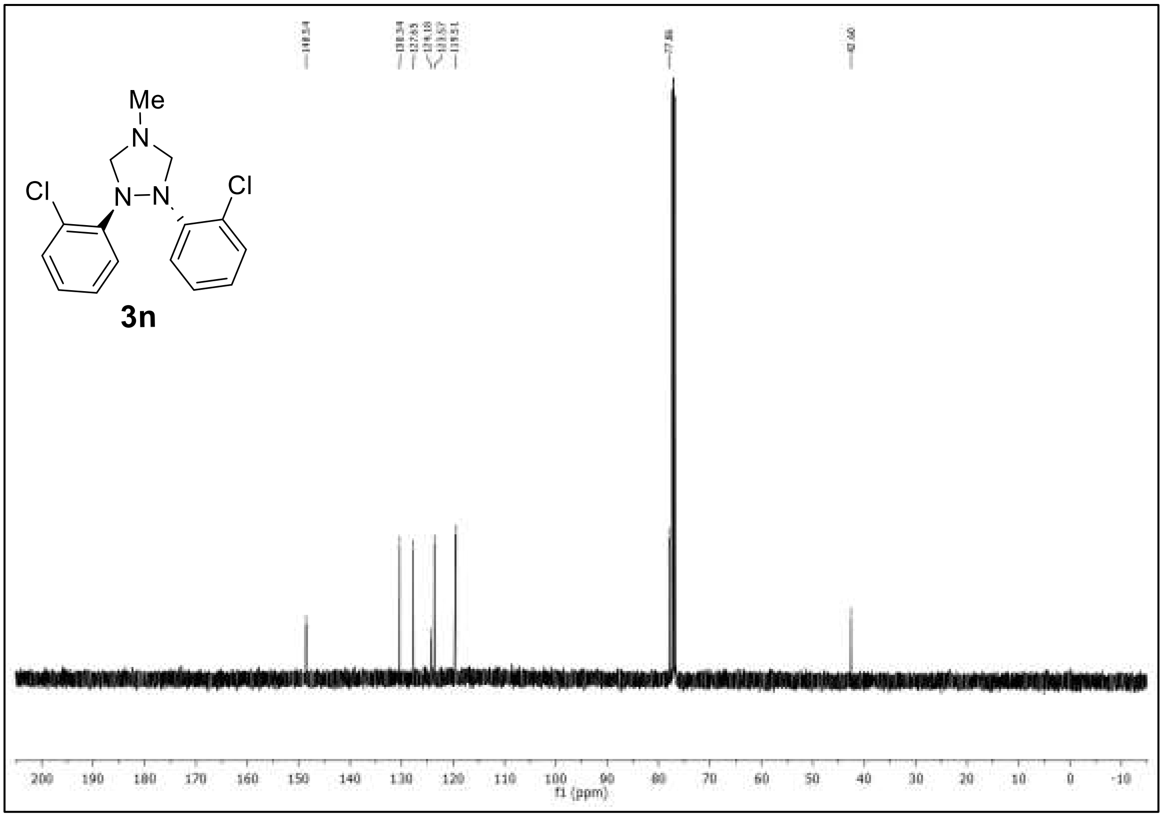


Figure S48: $^{13}C\{^{1}H\}$ NMR spectrum of **3n** (in $CDCl_3$, 126 MHz)

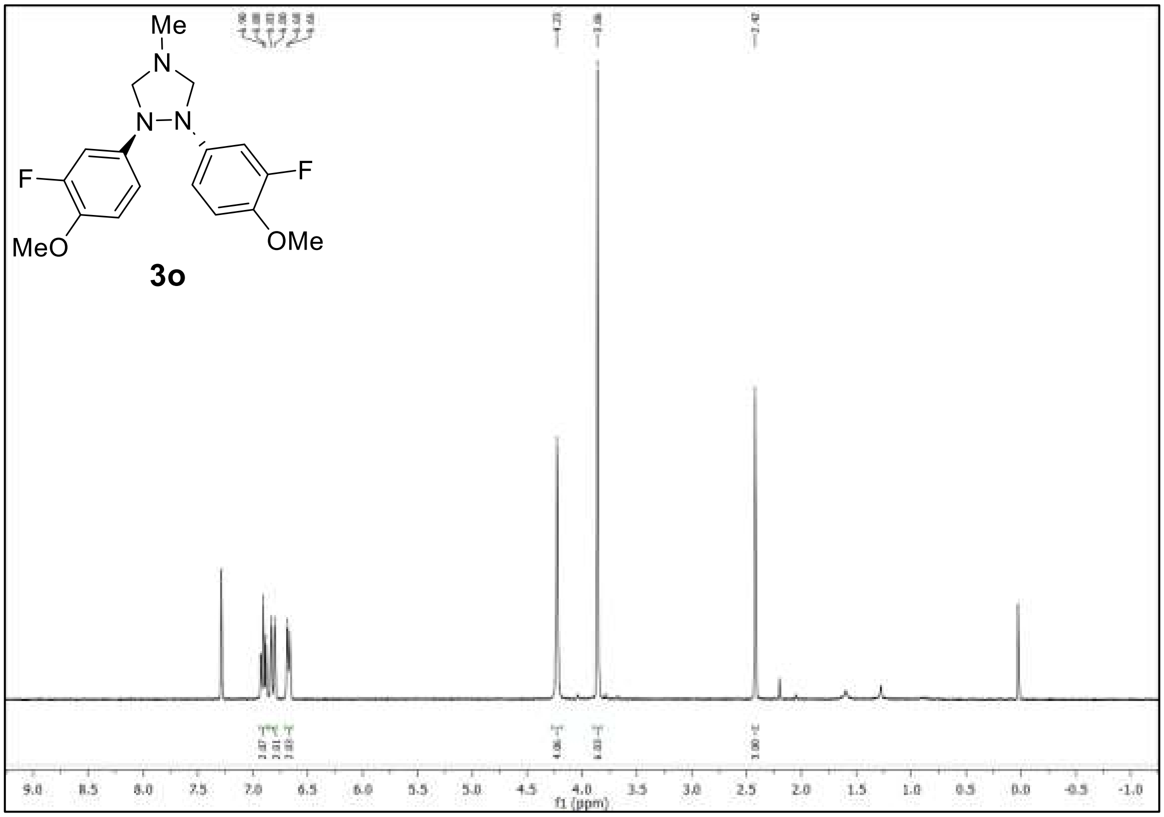

Figure S49: $^{1}H$ NMR spectrum of **3o** (in $CDCl_3$, 400 MHz)

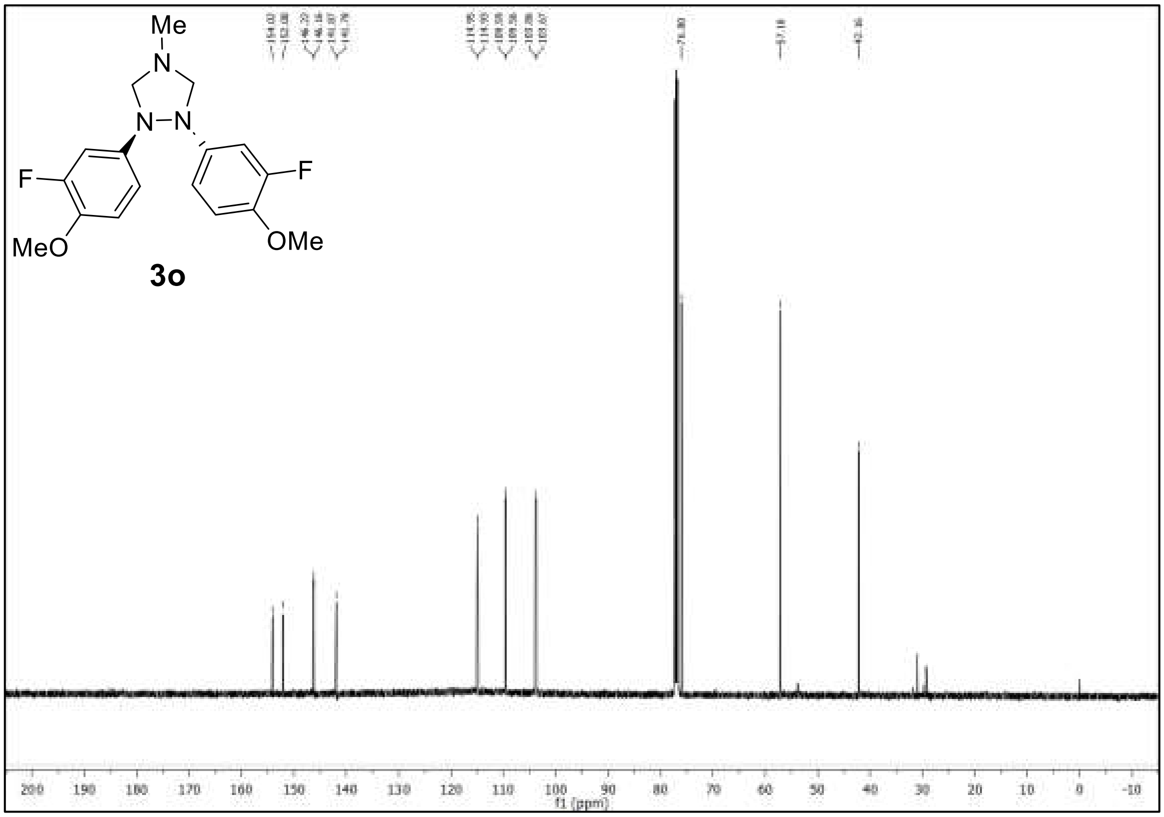

Figure S50: $^{13}C$ {$^{1}H$} NMR spectrum of **3o** (in $CDCl_3$, 126 MHz)

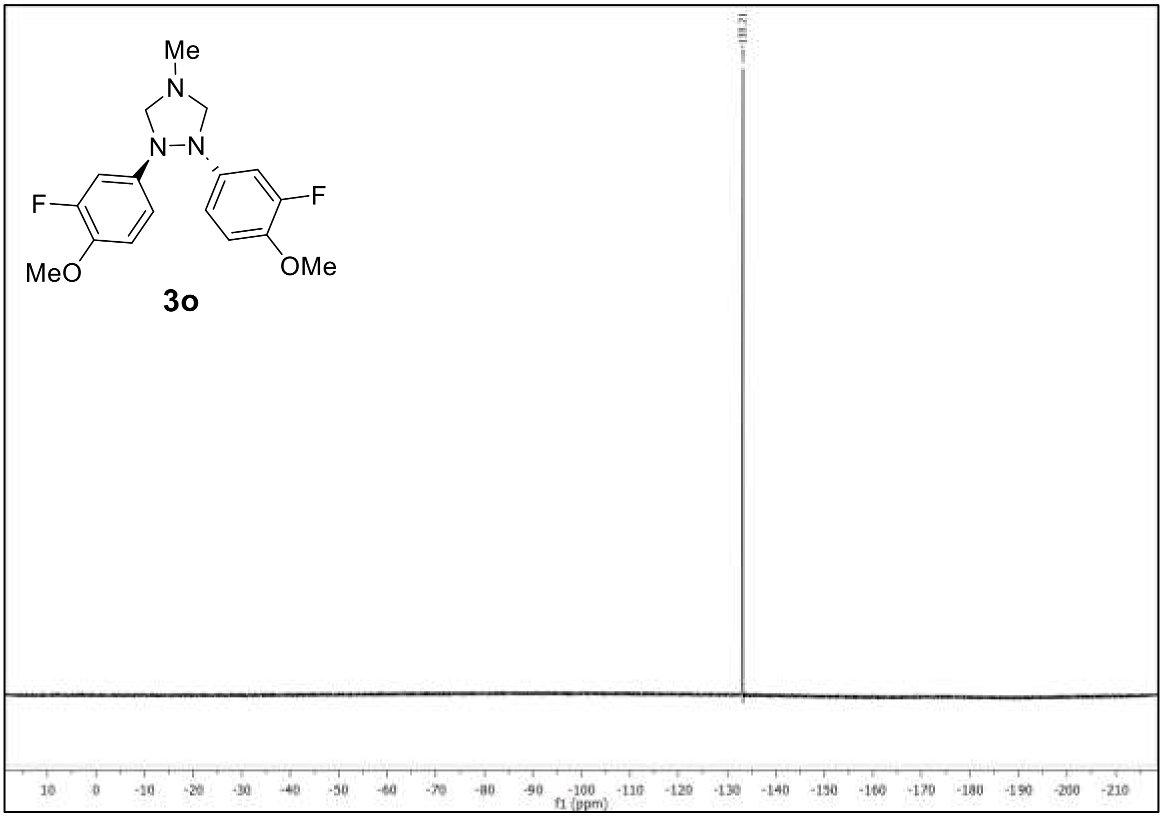


Figure S51: $^{19}$F NMR spectrum of **3o** (in $CDCl_3$, 376 MHz)

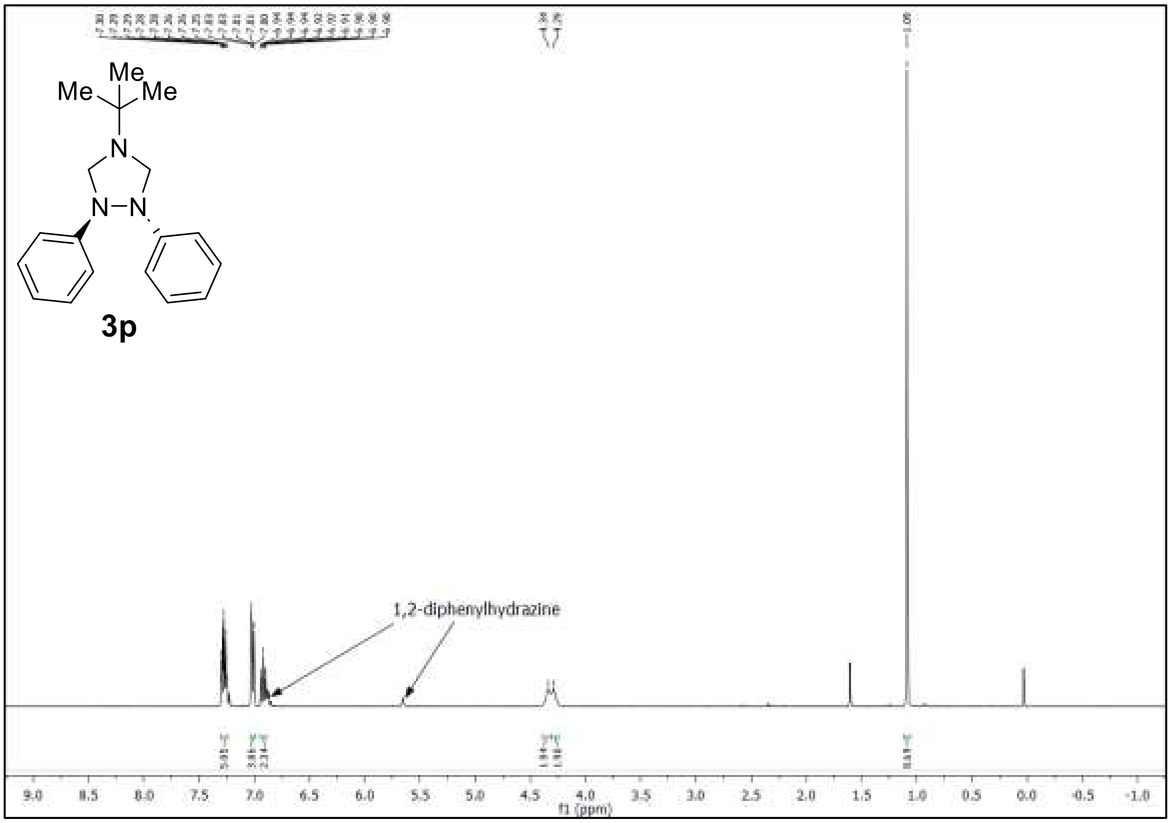


Figure S52: $^{1}H$ NMR spectrum of **3p** (in $CDCl_3$, 400 MHz)

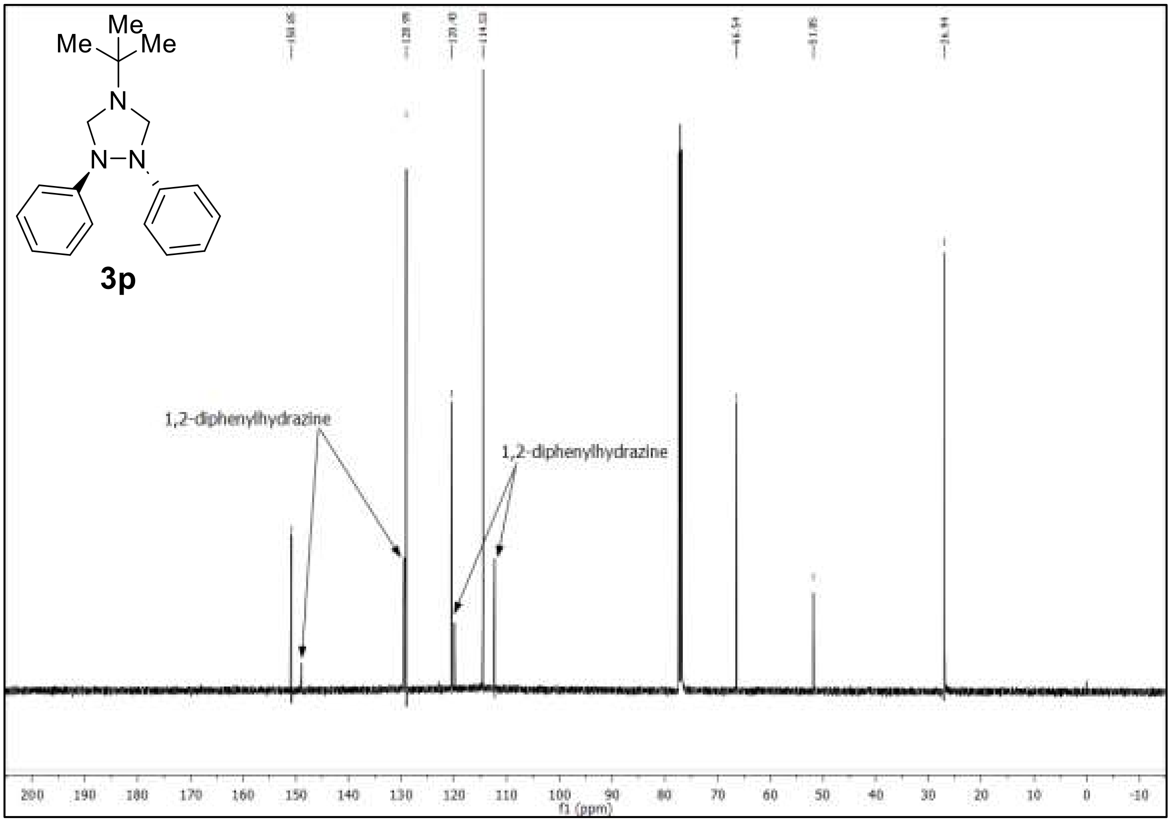


Figure S53: $^{13}C$ {$^{1}H$} NMR spectrum of **3p** (in $CDCl_3$, 126 MHz)

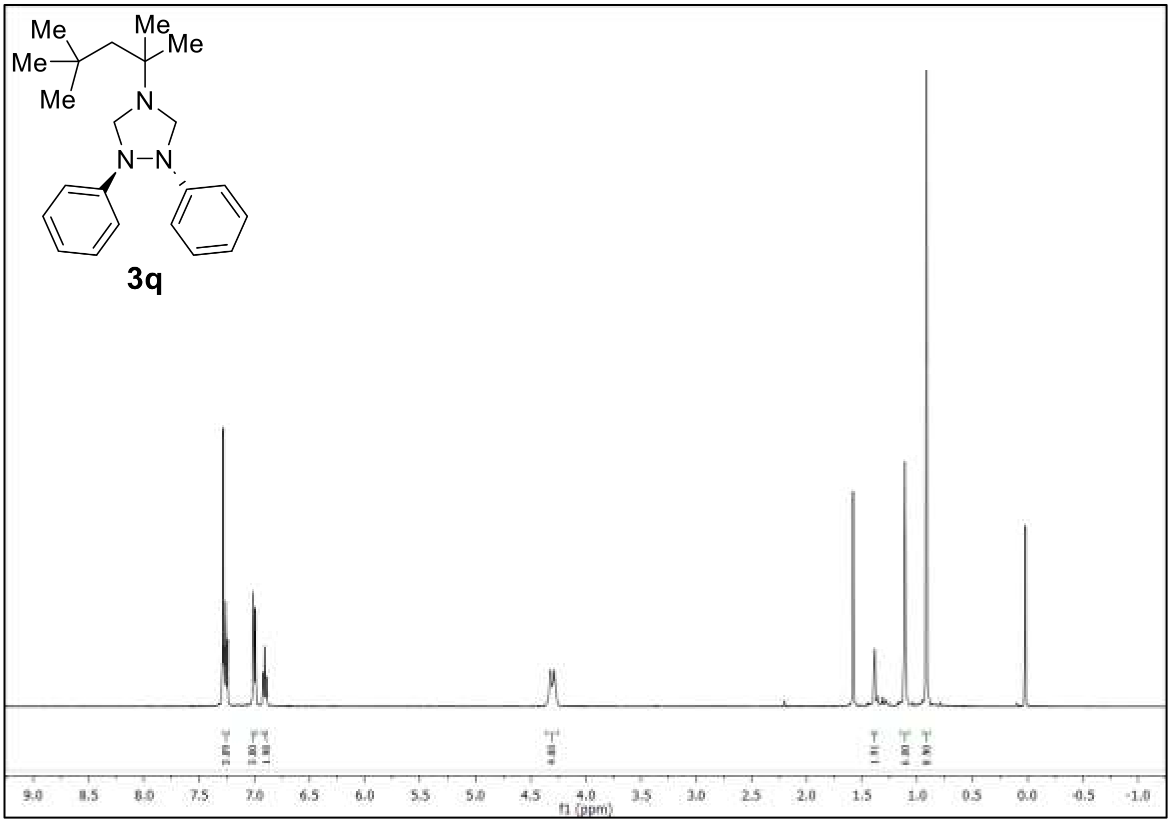

Figure S54: $^{1}$H NMR spectrum of **3q** (in $CDCl_3$, 500 MHz)

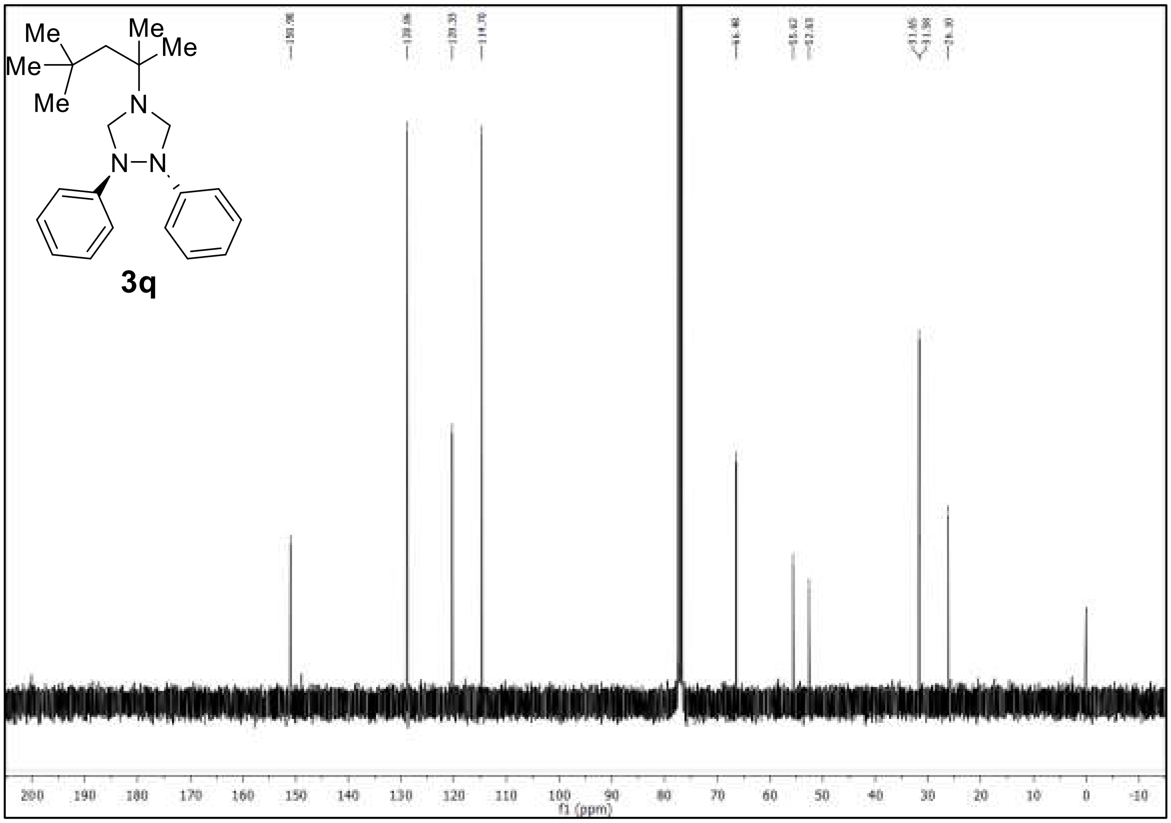

Figure S55: $^{13}C\ \{^{1}H\}$ NMR spectrum of **3q** (in $CDCl_3$, 101 MHz)

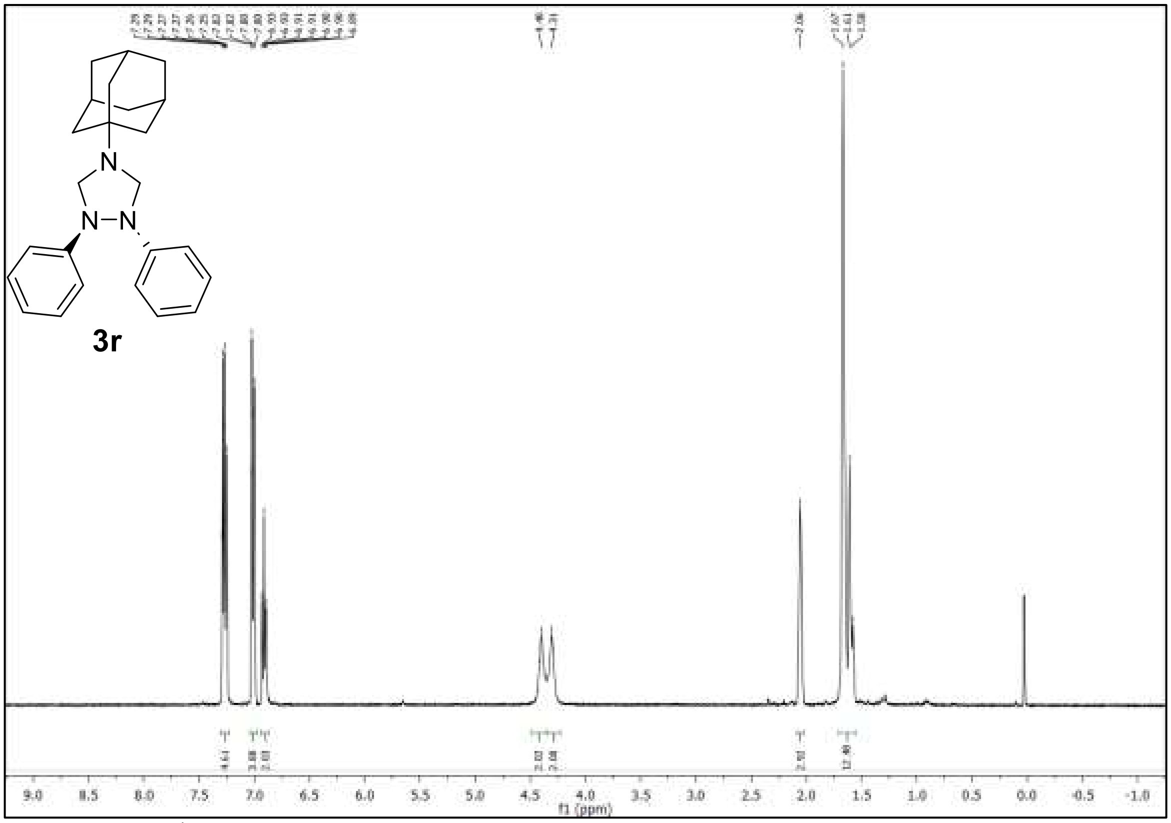

Figure S56: $^{1}H$ NMR spectrum of **3r** (in $CDCl_3$,400 MHz)

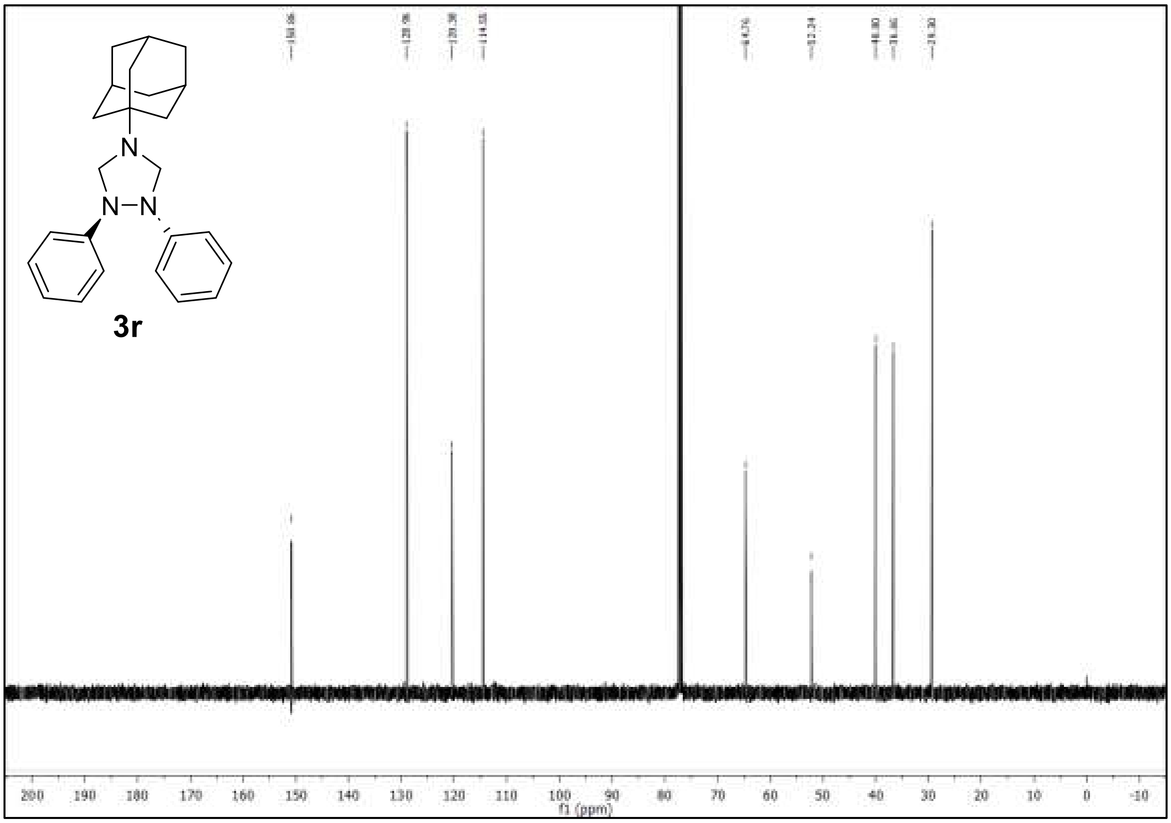

Figure S57: $^{13}C$ {$^{1}H$} NMR spectrum of **3r** (in $CDCl_3$, 101 MHz)

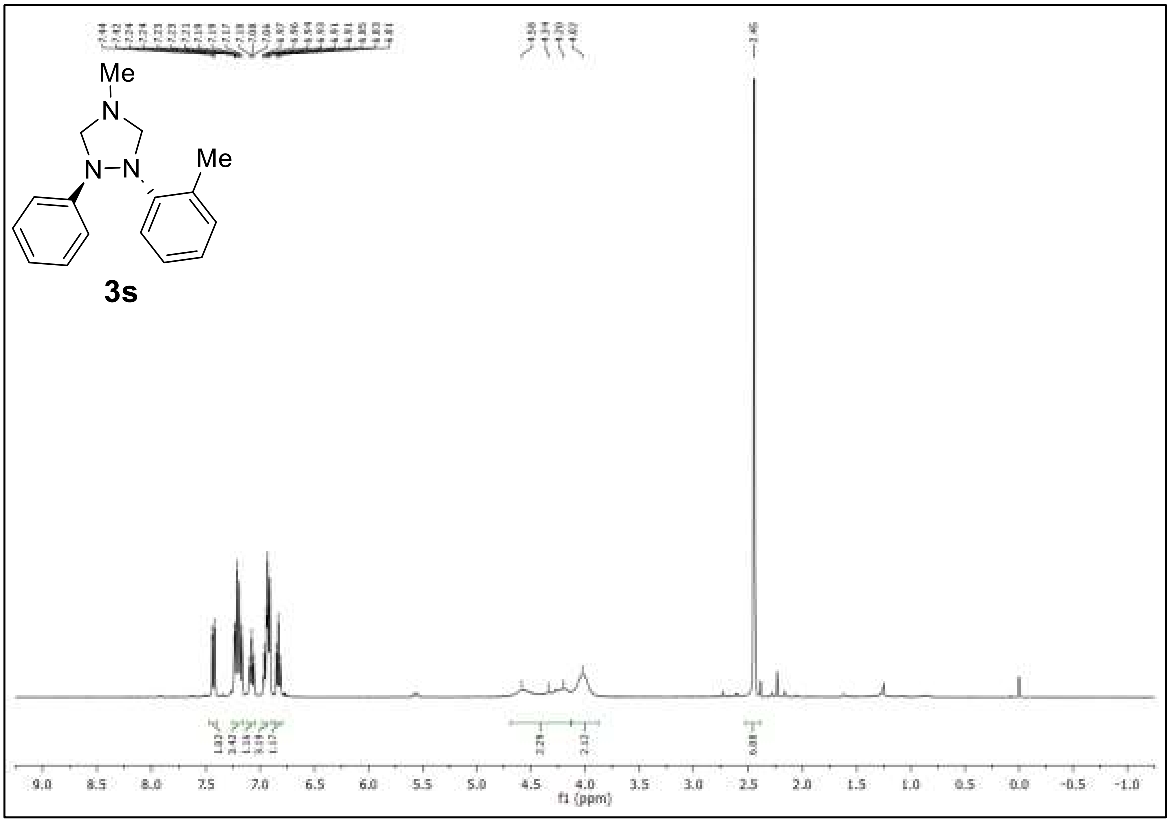


Figure S58: $^{1}$H NMR spectrum of **3s** (in $CDCl_3$, 400 MHz)

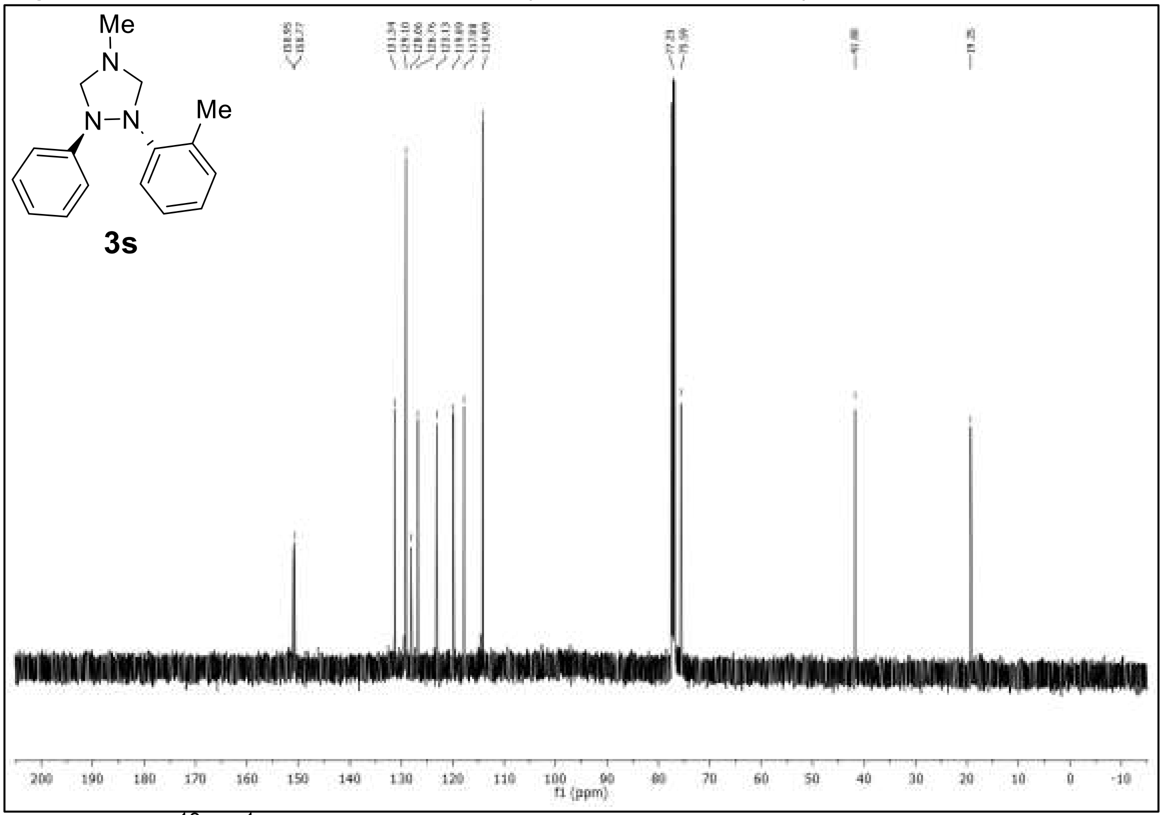


Figure S59: $^{13}$C {$^{1}$H} NMR spectrum of **3s** (in $CDCl_3$, 126 MHz)

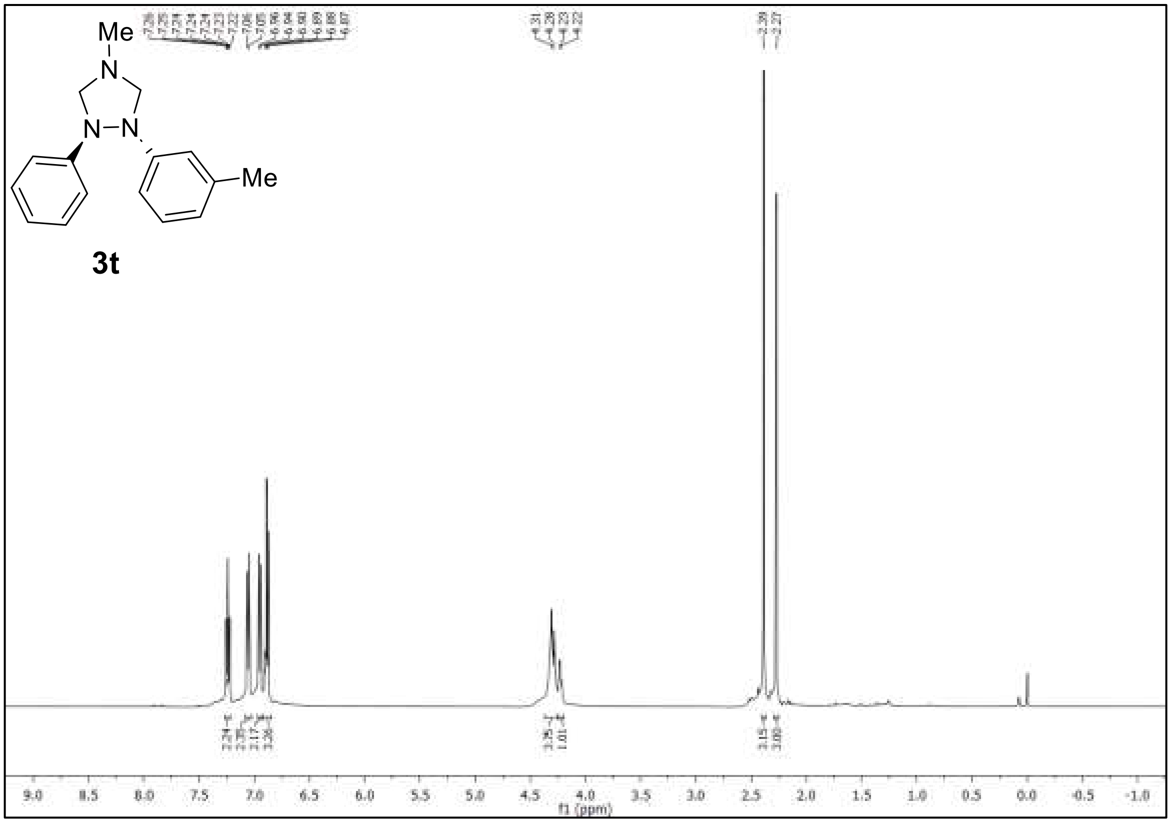


Figure S60: $^{1}H$ NMR spectrum of **3t** (in $CDCl_3$, 400 MHz)

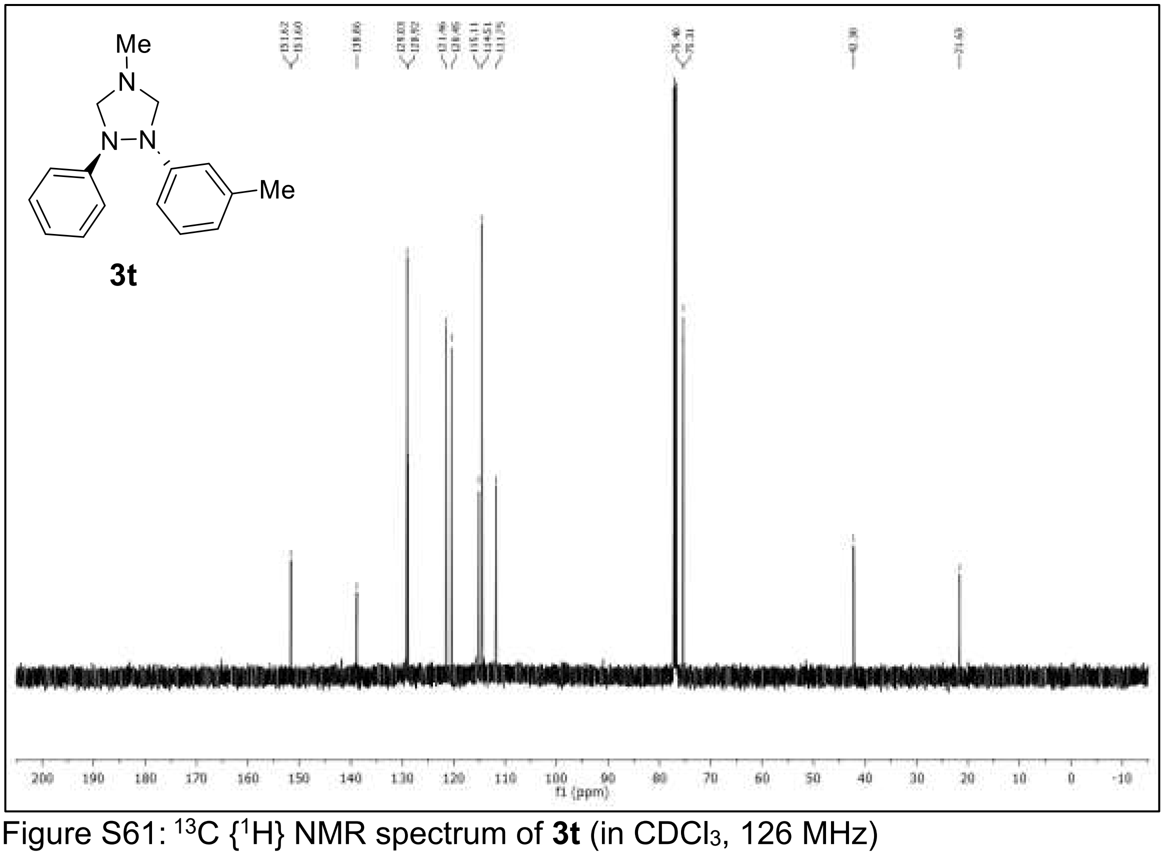


Figure S61: $^{13}C$ {$^{1}H$} NMR spectrum of **3t** (in $CDCl_3$, 126 MHz)

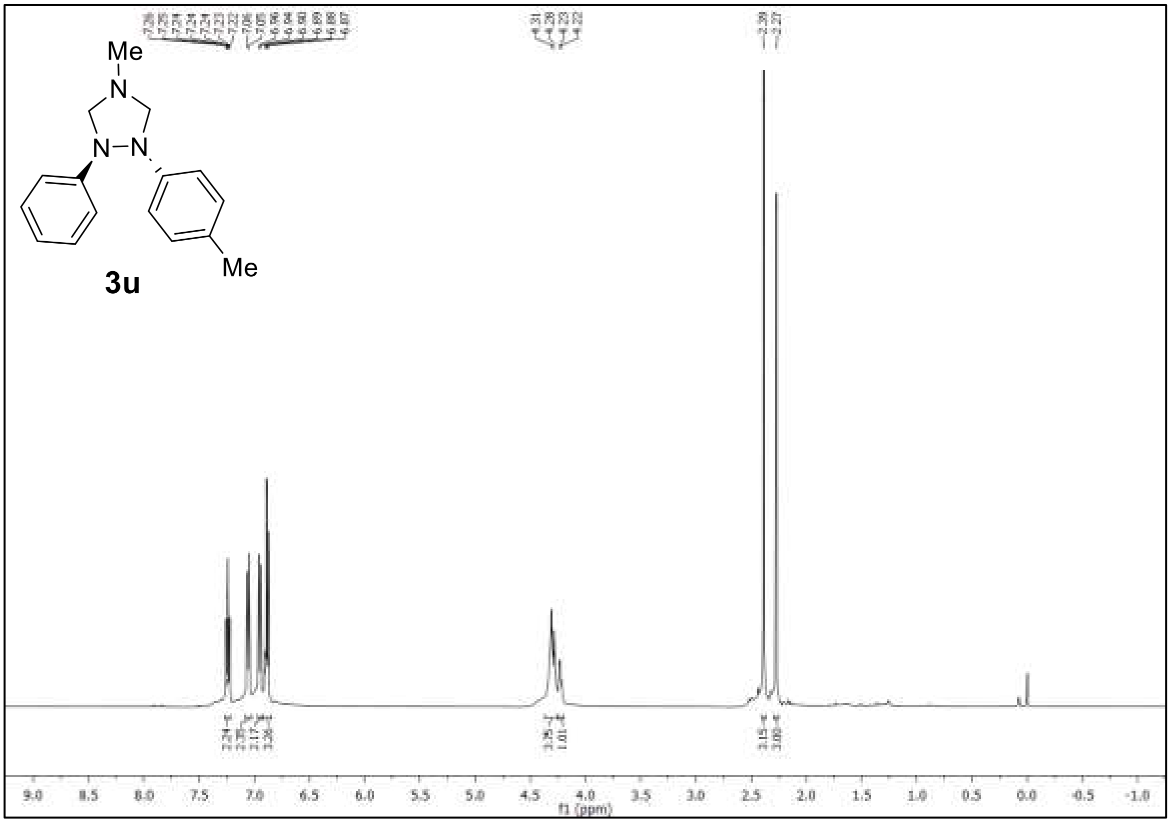

Figure S62: $^1$H NMR spectrum of **3u** (in $CDCl_3$, 400 MHz)

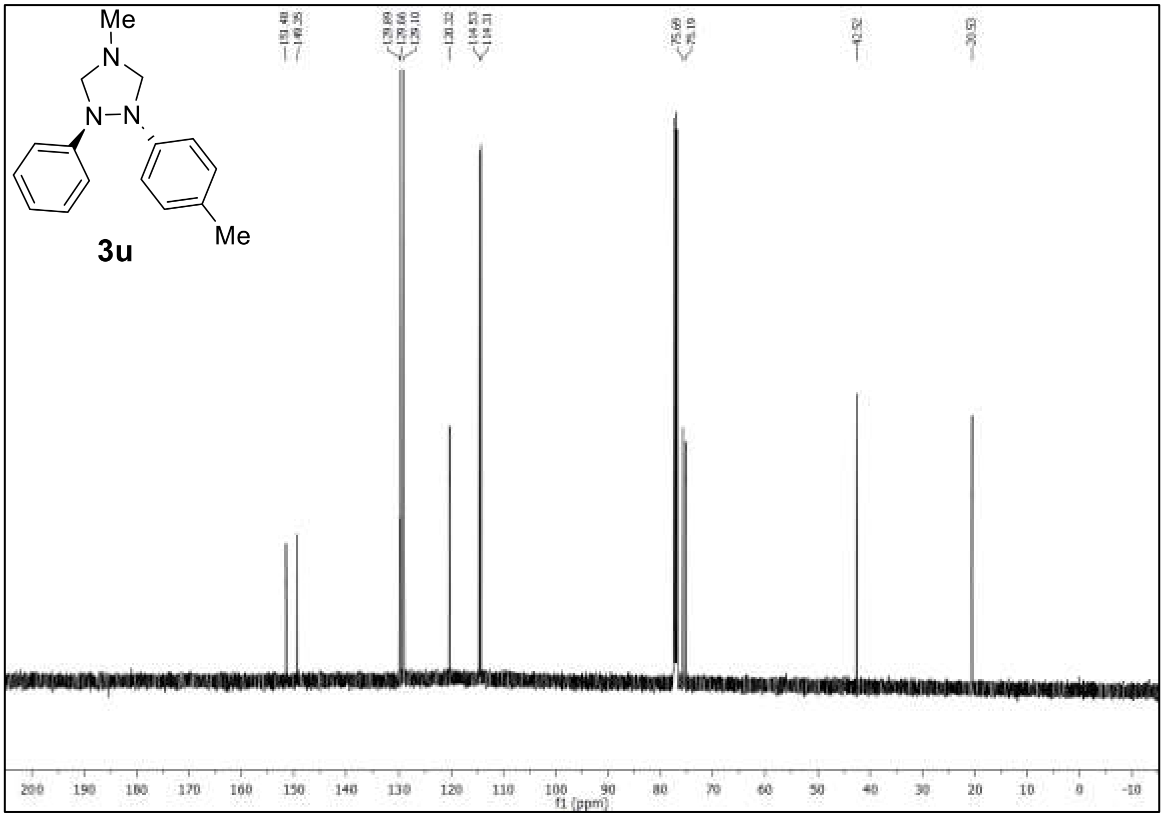

Figure S63: $^{13}C$ {$^1$H} NMR spectrum of **3u** (in $CDCl_3$, 126 MHz)

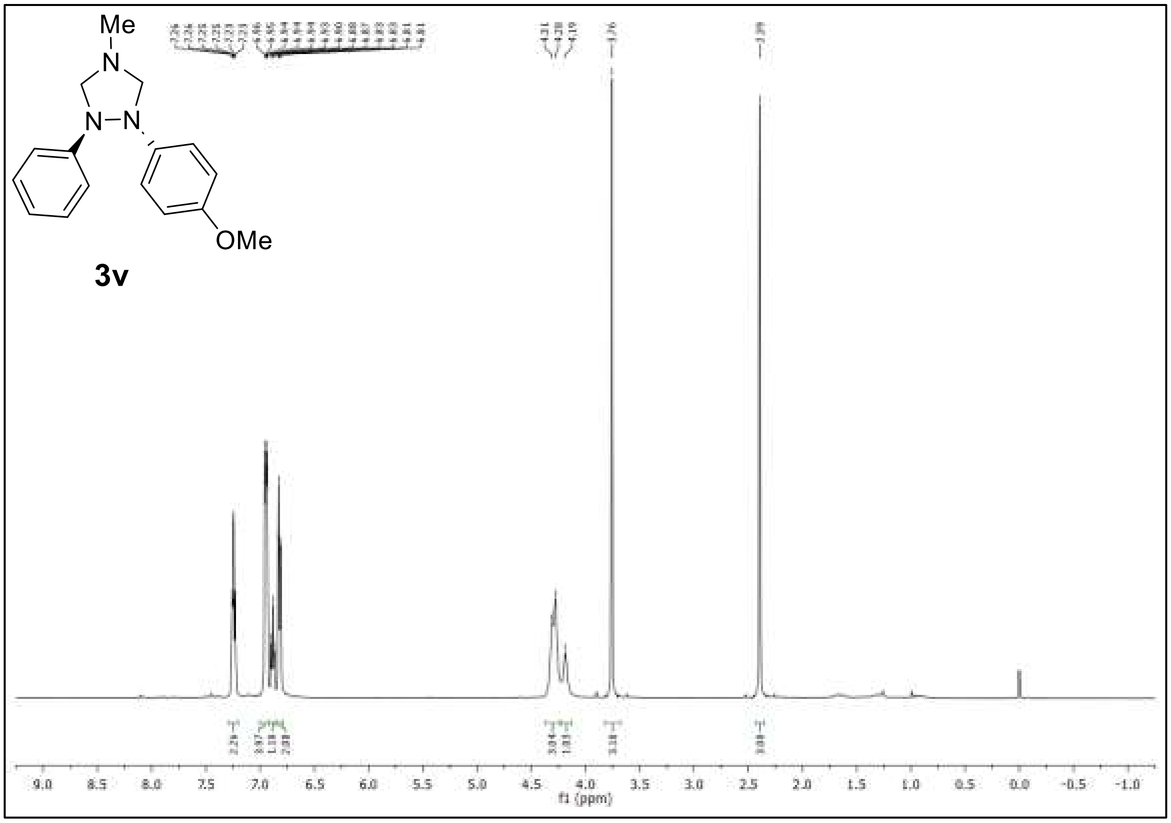


Figure S64: ¹H NMR spectrum of **3v** (in $CDCl_3$, 400 MHz)

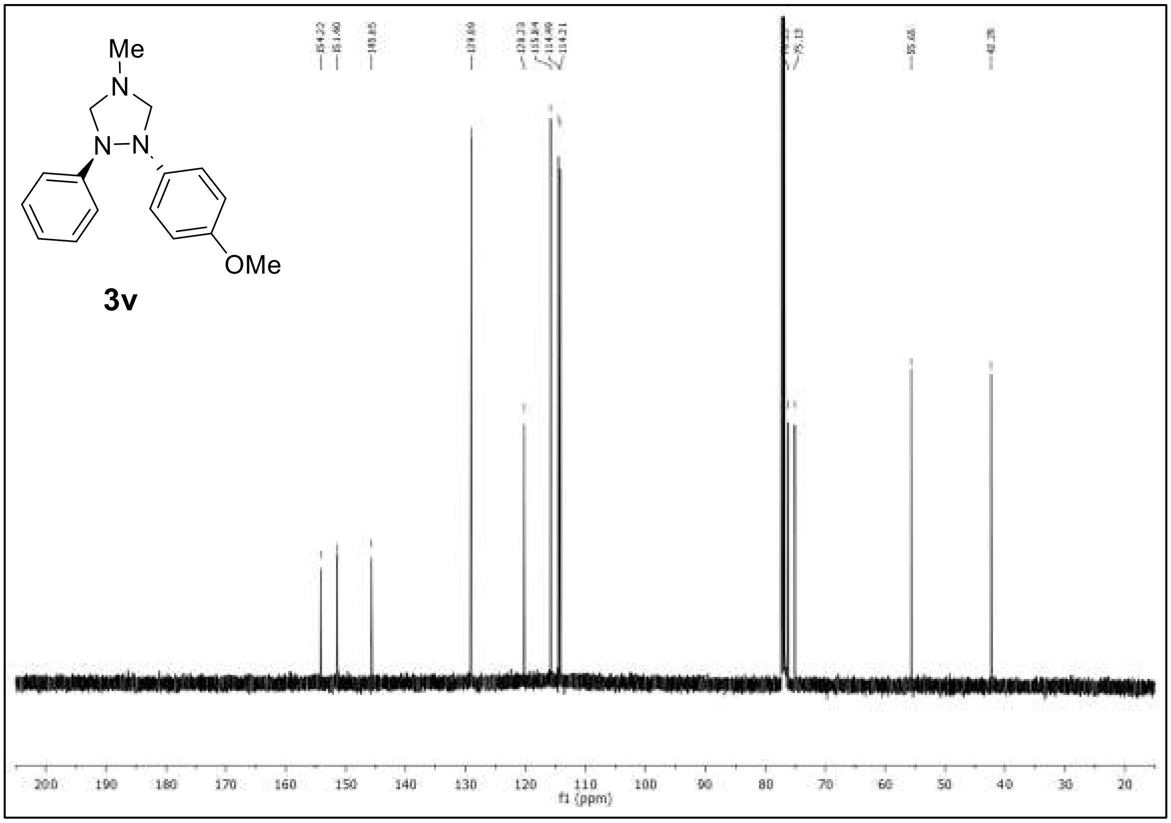


Figure S65: $^{13}C$ {$^1H$} NMR spectrum of **3v** (in $CDCl_3$, 126 MHz)

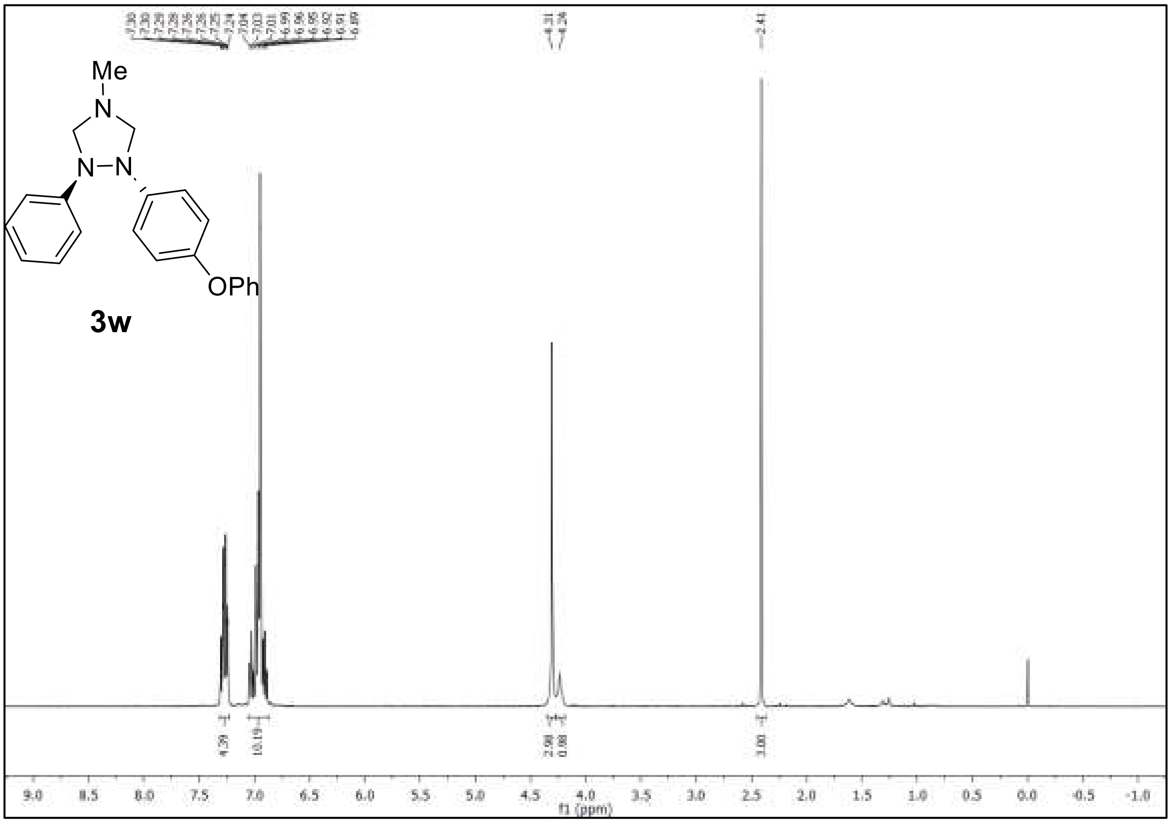

Figure S66: $^1$H NMR spectrum of **3w** (in $CDCl_3$, 400 MHz)

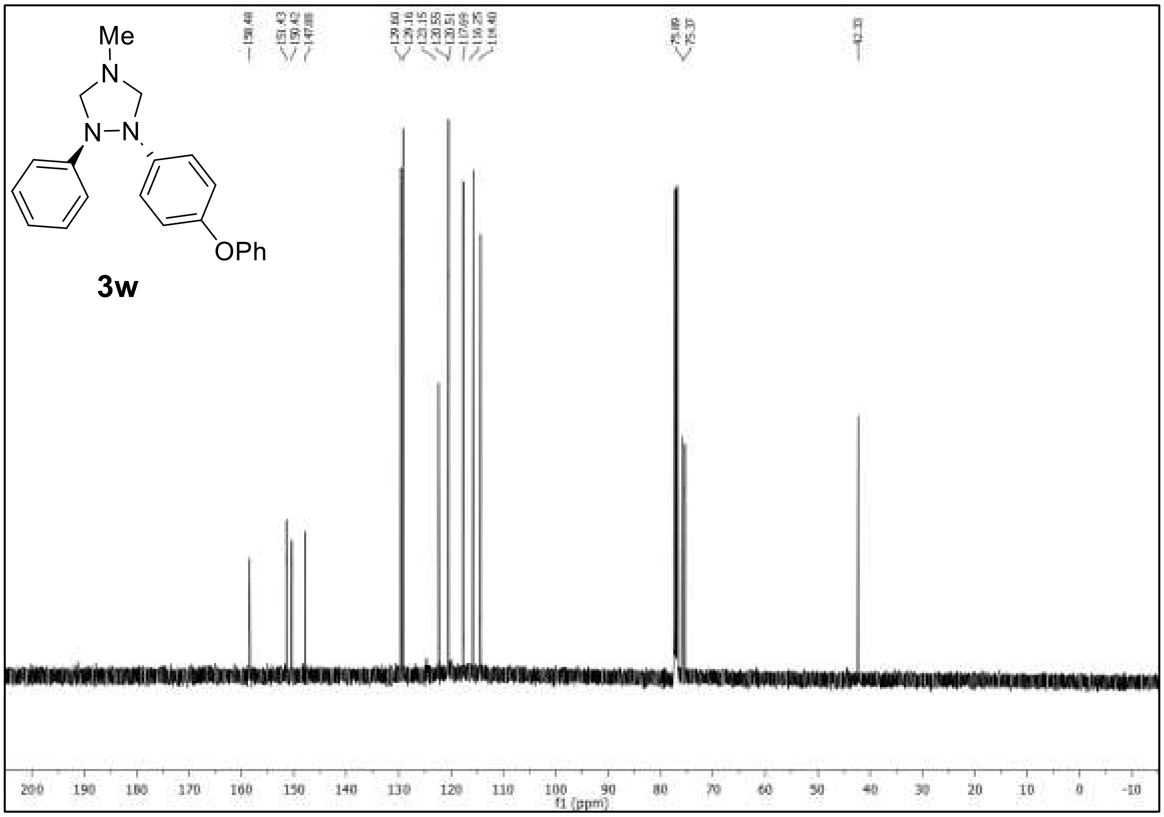

Figure S67: $^{13}C$ {$^1$H} NMR spectrum of **3w** (in $CDCl_3$, 126 MHz)

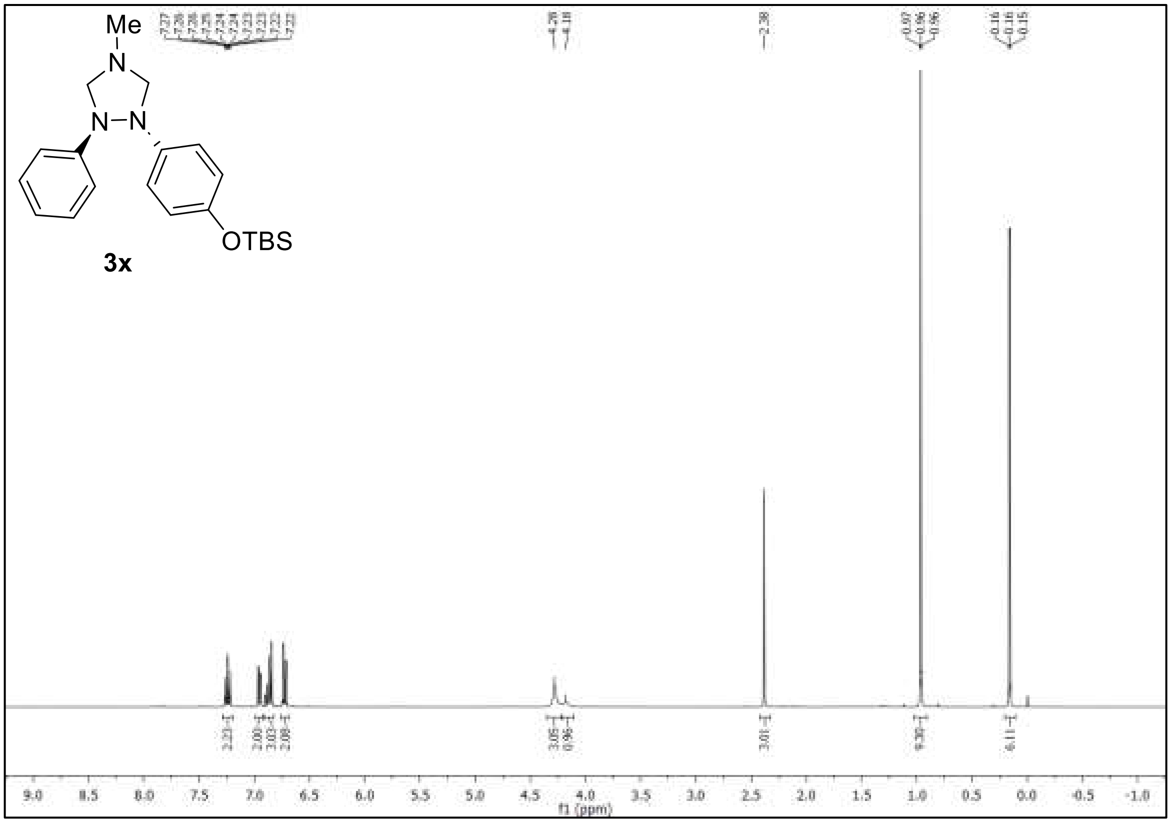

Figure S68: $^1H$ NMR spectrum of **3x** (in $CDCl_3$, 400 MHz)

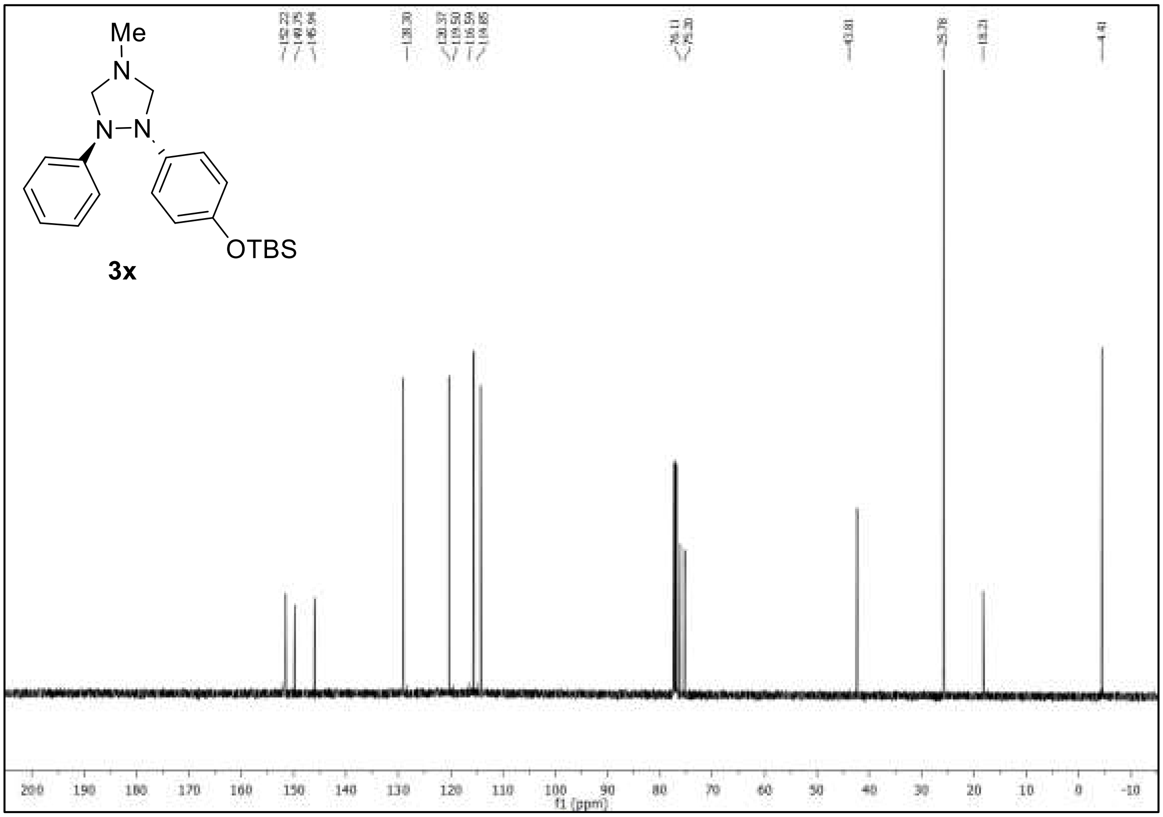

Figure S69: $^{13}C\{^1H\}$ NMR spectrum of **3x** (in $CDCl_3$, 126 MHz)

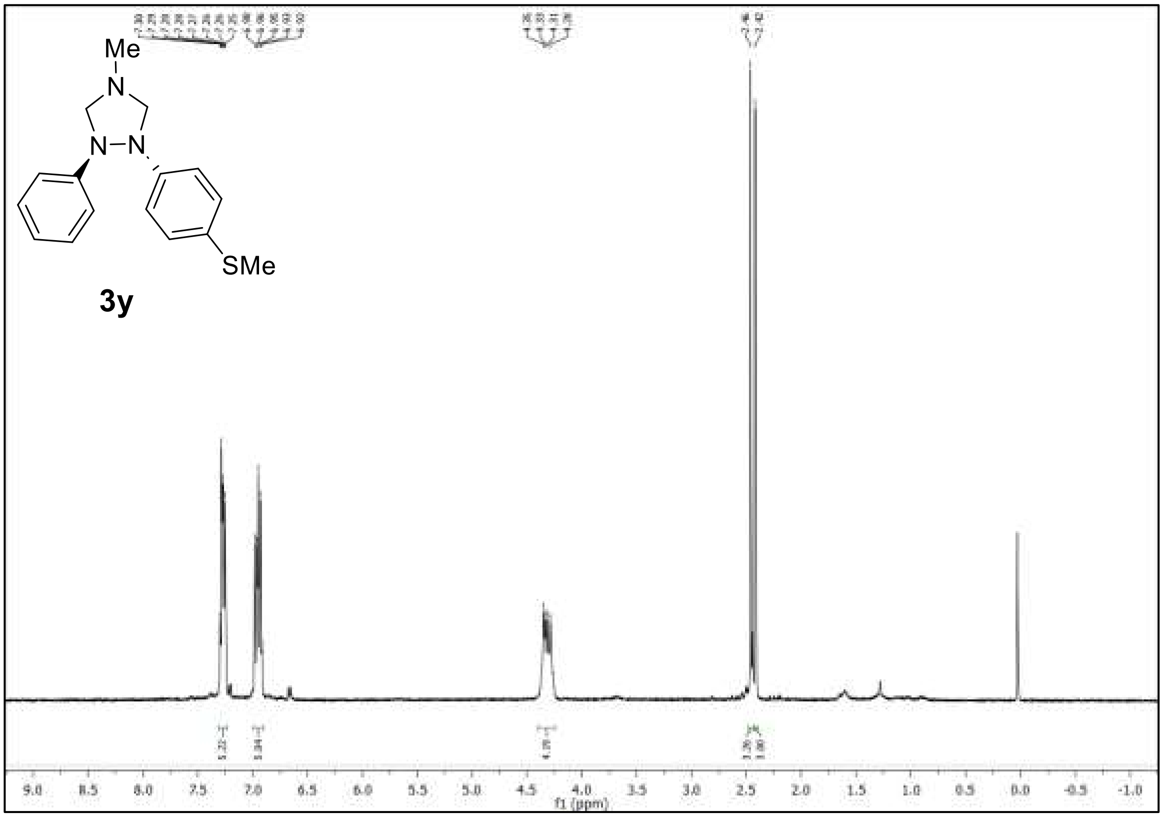


Figure S70: $^{1}H$ NMR spectrum of **3y** (in $CDCl_3$, 400 MHz)

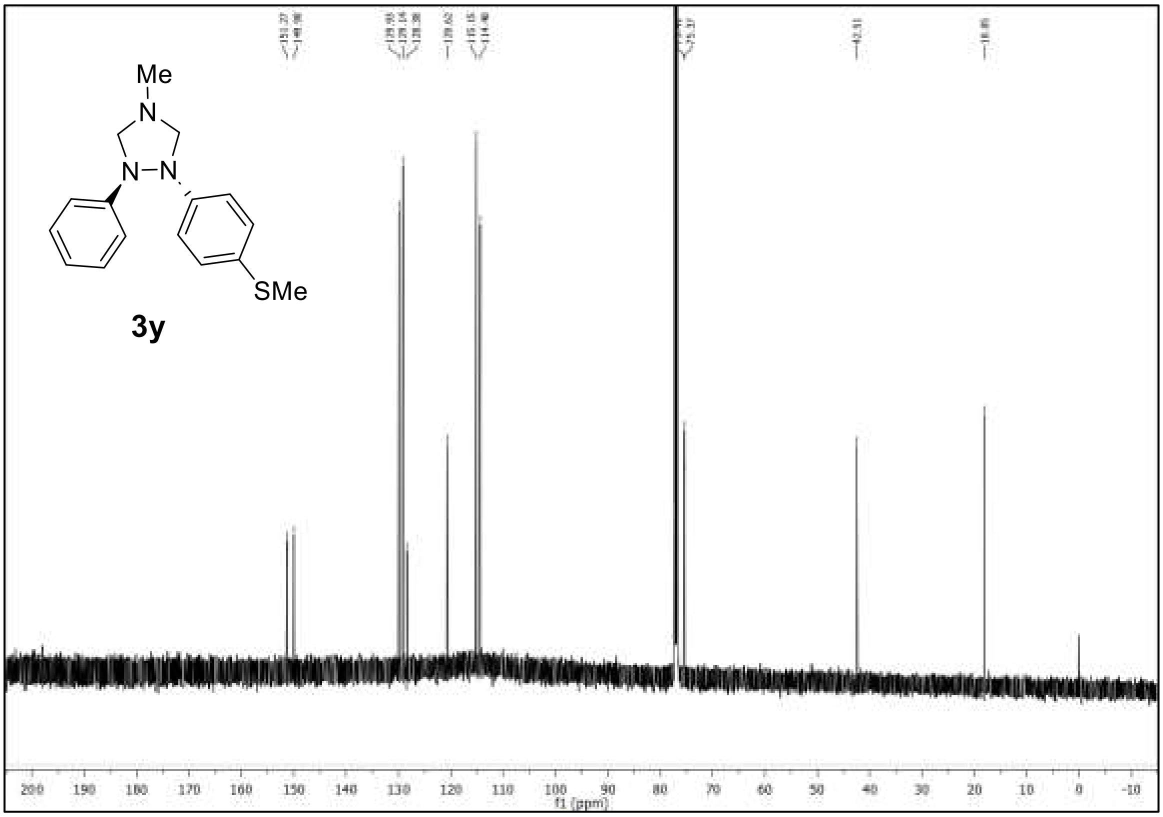


Figure S71: $^{13}C$ {$^{1}H$} NMR spectrum of **3y** (in $CDCl_3$, 126 MHz)

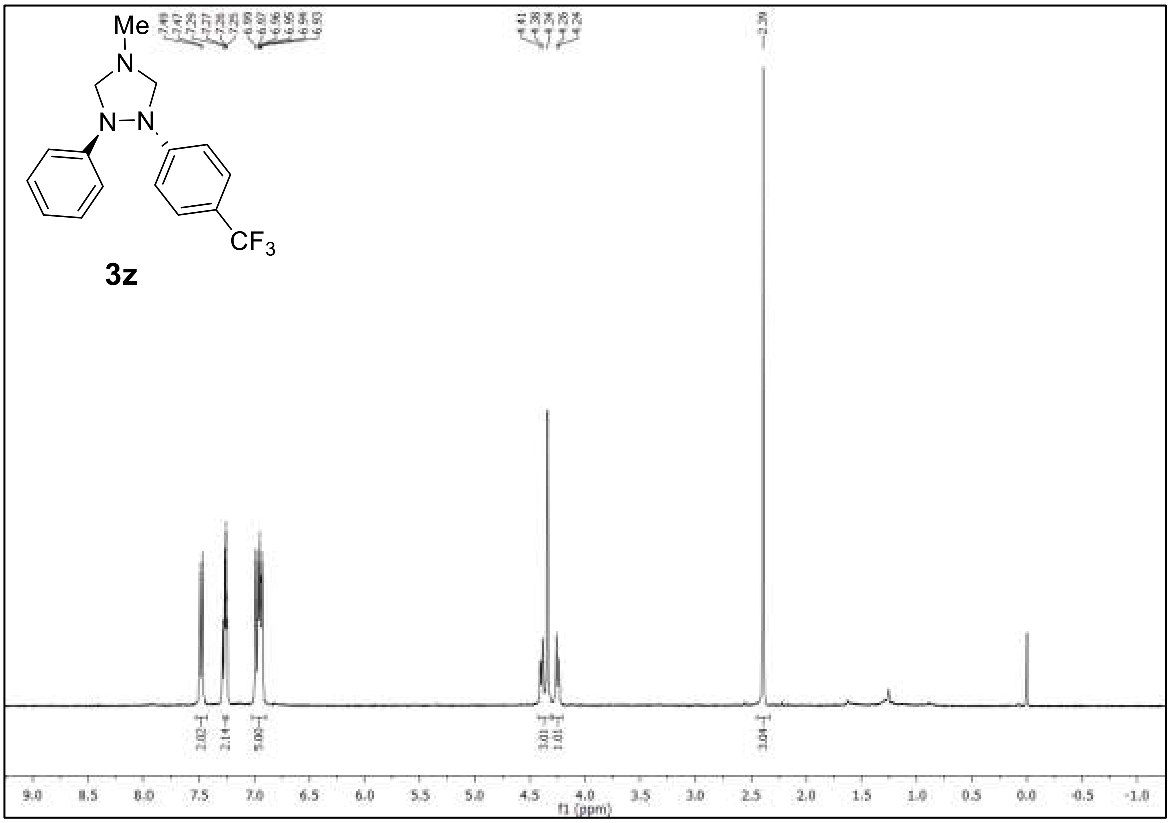


Figure S72: $^{1}H$ NMR spectrum of **3z** (in $CDCl_3$, 400 MHz)

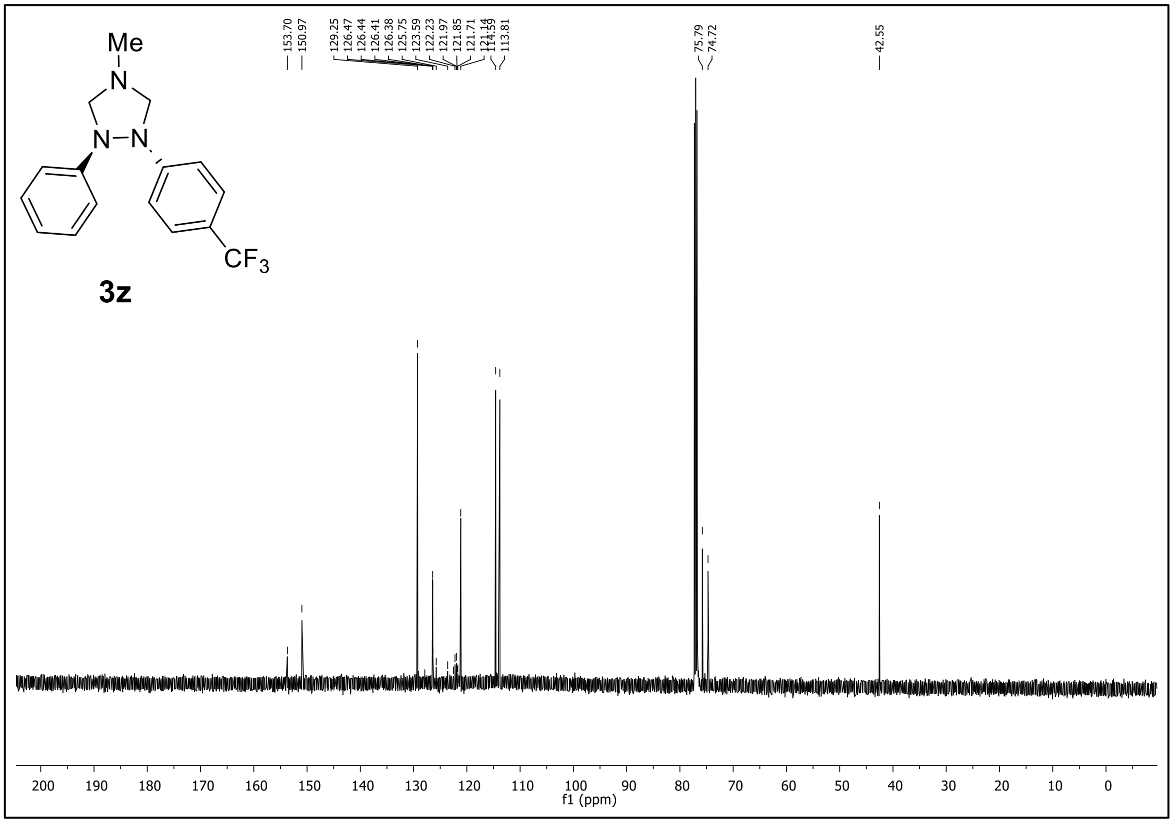


Figure S73: $^{13}C\{^{1}H\}$ NMR spectrum of **3z** (in $CDCl_3$, 126 MHz)

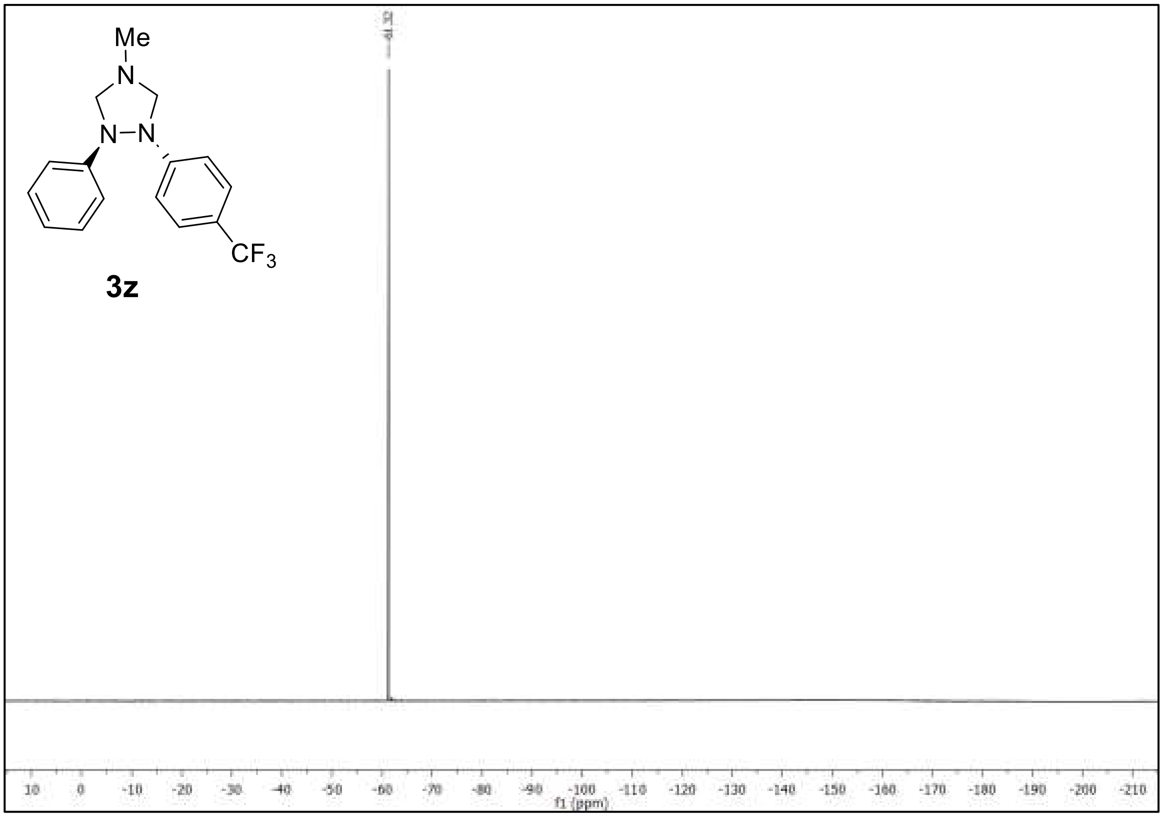

Figure S74: $^{19}F$ NMR spectrum of **3z** (in $CDCl_3$, 376 MHz)

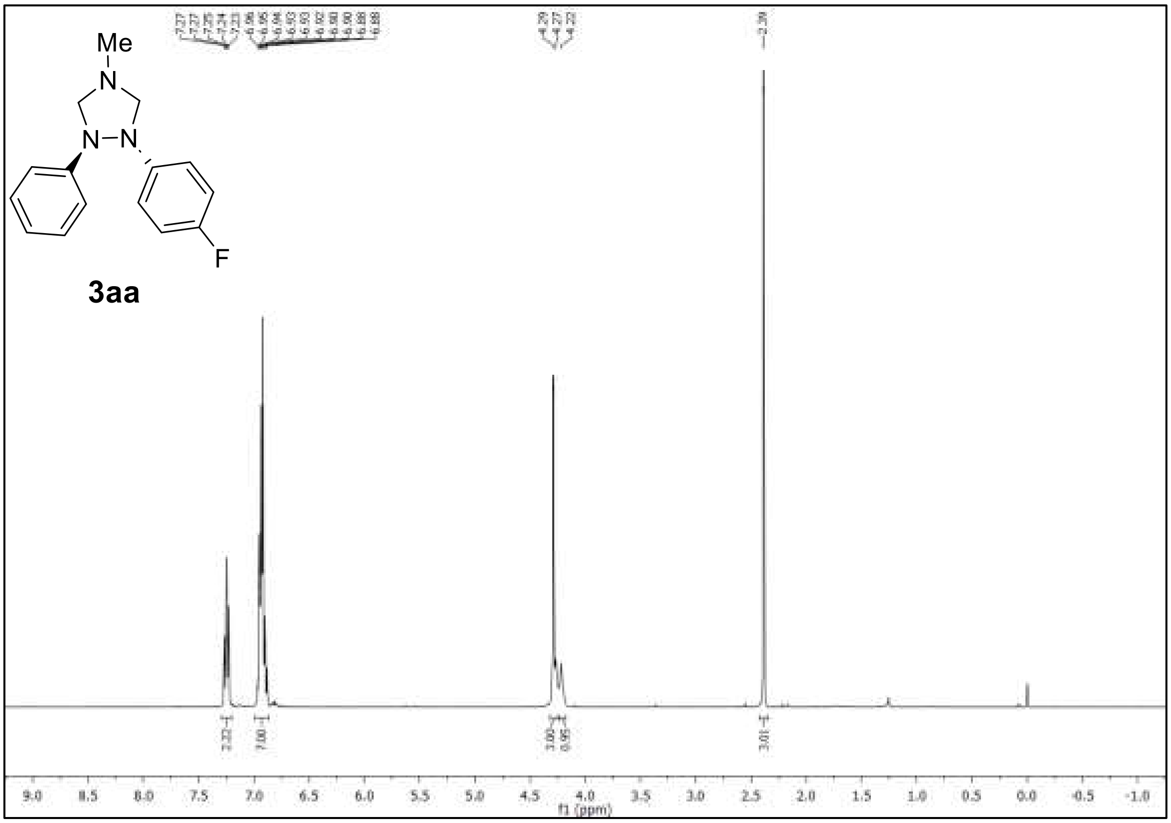

Figure S75: $^{1}H$ NMR spectrum of **3aa** (in $CDCl_3$, 400 MHz)

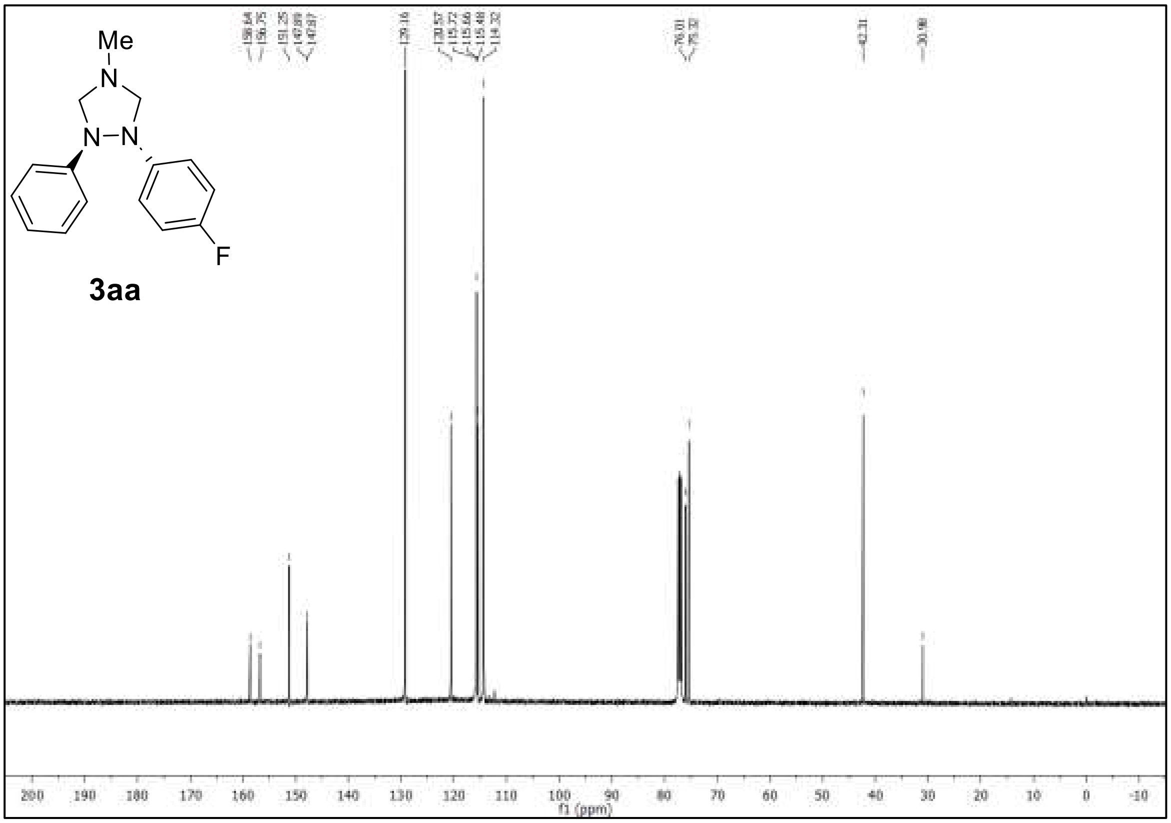

Figure S76: $^{13}C\{^{1}H\}$ NMR spectrum of **3aa** (in $CDCl_3$, 126 MHz)

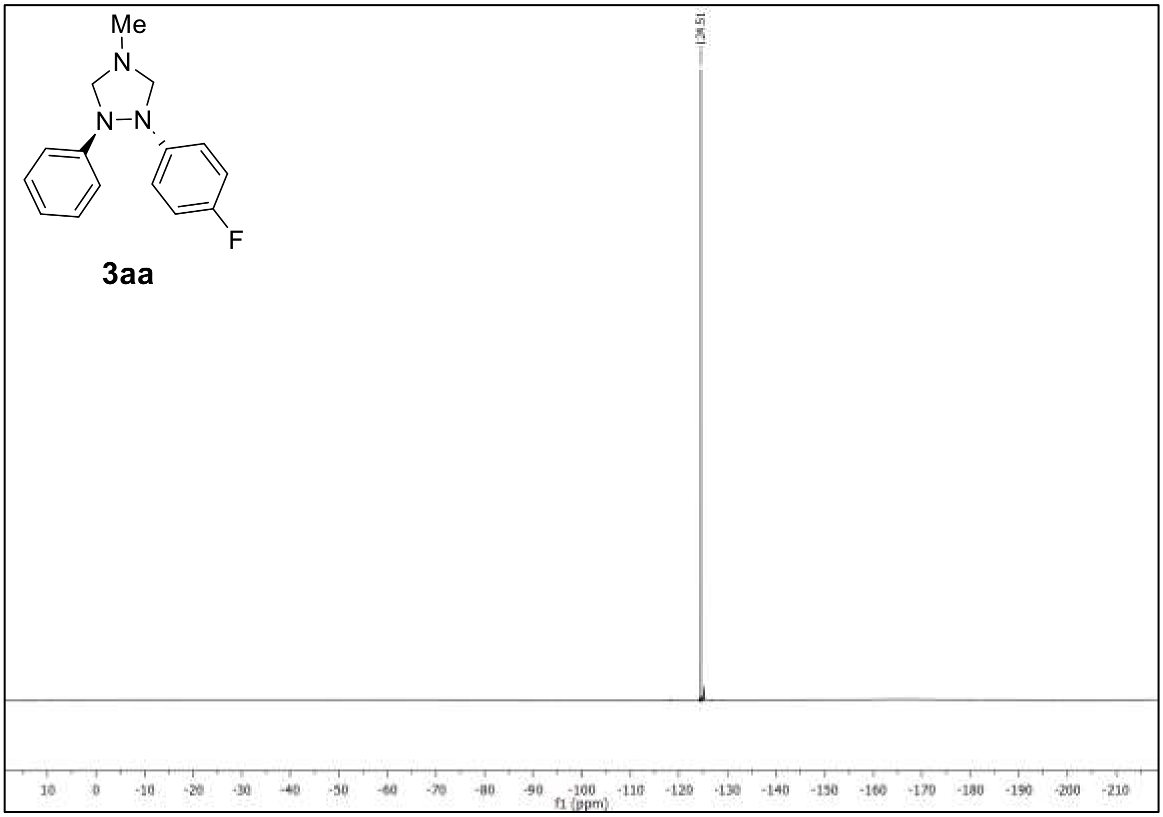


Figure S77: $^{19}$F NMR spectrum of **3aa** (in $CDCl_3$, 376 MHz)

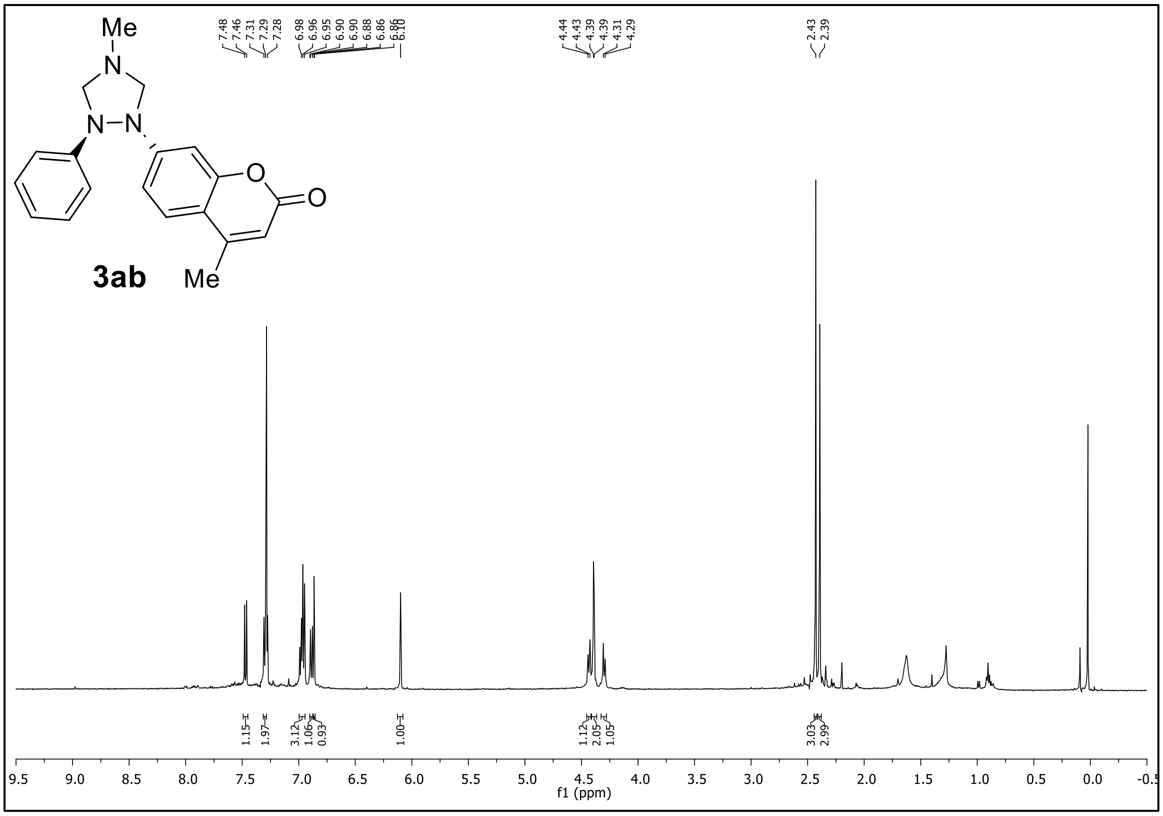


Figure S78: $^{1}H$ NMR spectrum of **3ab** (in $CDCl_3$, 500 MHz)

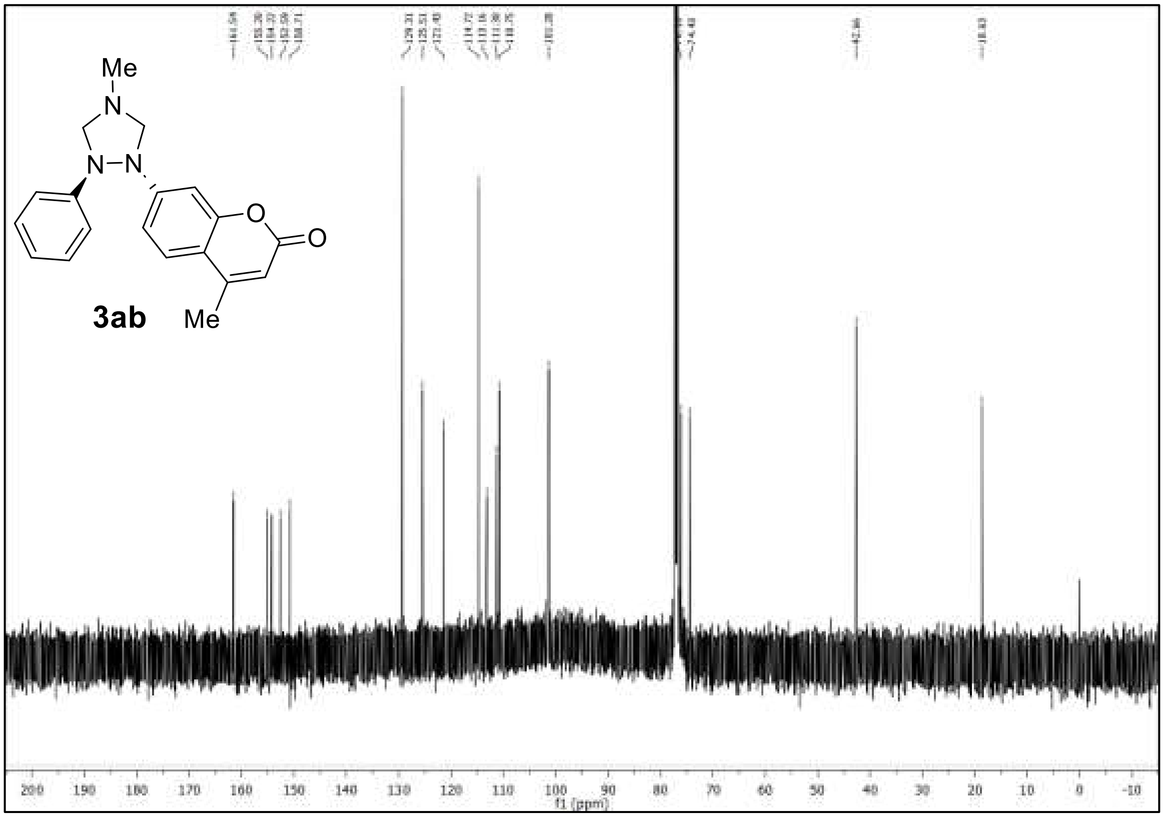


Figure S79: $^{13}C$ {$^{1}H$} NMR spectrum of **3ab** (in $CDCl_3$, 126 MHz)

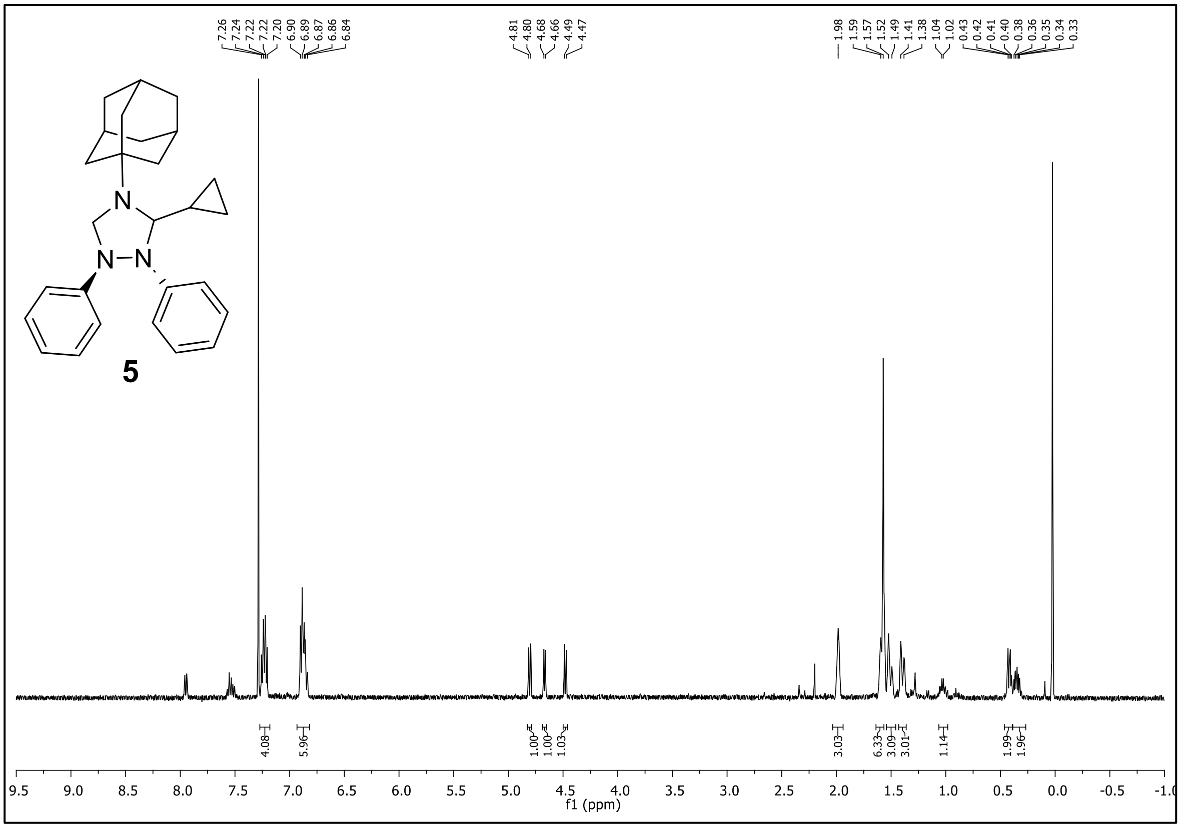


Figure S80: $^{1}H$ NMR spectrum of **5** (in $CDCl_3$, 400 MHz)

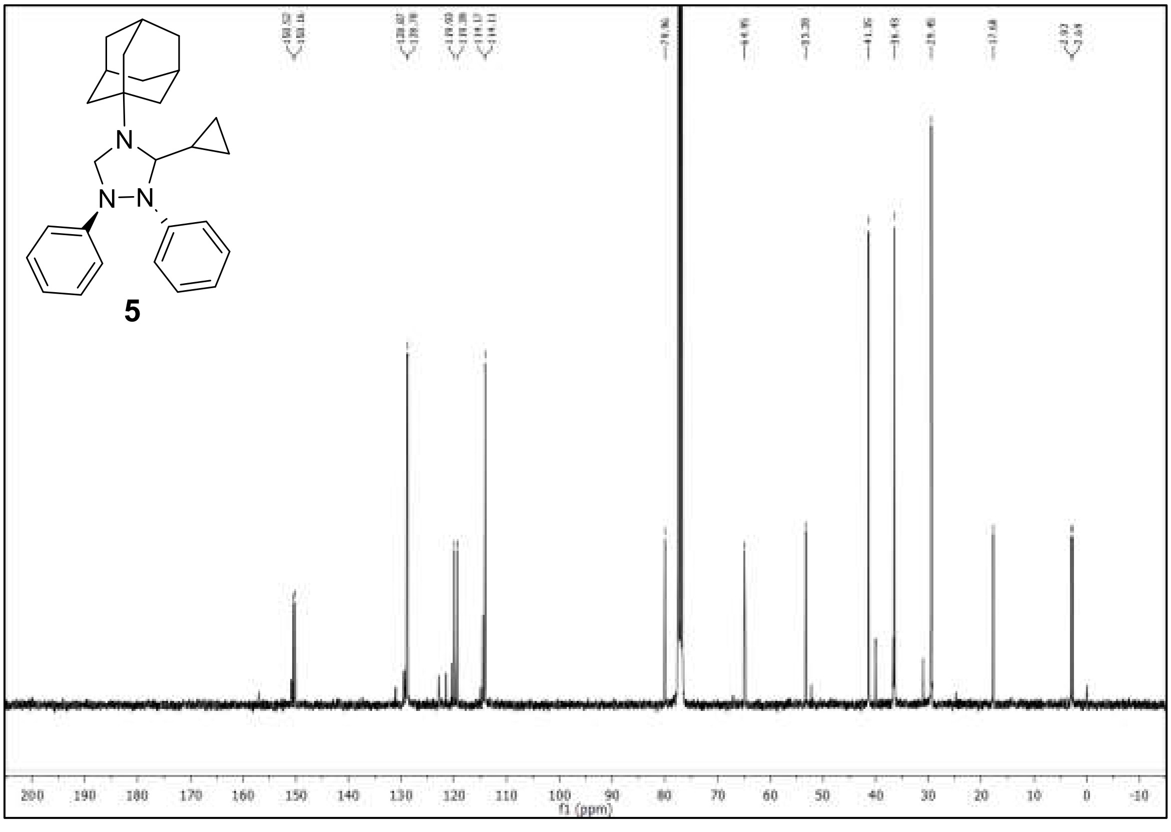


Figure S81 $^{13}C$ {$^{1}H$} NMR spectrum of **5** (in $CDCl_3$, 101 MHz)

## Biological Studies

**Bacterial strains:** *Salmonella enterica* Typhimurium SL1344 effectorless,[19] *Escherichia coli* DH5α, and *Shigella flexneri* M90T[20] were grown using Lysogeny Broth (LB) media. *Listeria monocytogenes* 10403S *Δhly*[21] was grown using Brain Heart Infusion (BHI) media. All strains were gifts from Dr. Neal Alto (University of Texas Southwestern Medical School) or Dr. Dan Portnoy (University of California-Berkley).

**Zone of Inhibition Assays:** Inhibition of bacterial growth on solid medium was performed as previously described.[22] Briefly, lag phase bacteria were spread across a solid agarose medium and then a 4 mm Whatman filter disk containing the indicated amount of compound, or 2 mL of DMSO, was placed on top of the agarose. After 16-18 h, the zone of growth inhibition, or the area around the disk without bacterial growth, was manually measured using calipers.

**Table S1. Full table for antibacterial potential[a] of triazolidines**

| Compound | *Listeria* | *S*Tm | *Shigella* | *e. Coli* |
|---|---|---|---|---|
| 3a | 200 | 50 | 100 | 200 |
| 3b | 200 | 100 | 100 | 100 |
| 3c | -[b] | - | - | - |
| 3d | - | - | 200 | - |
| 3e | - | 200 | 100 | - |
| 3f | 200 | 100 | 50 | 100 |
| 3g | - | - | - | - |
| 3h | - | - | - | - |
| 3i | - | - | - | - |
| 3j | - | - | - | - |
| 3k | - | - | - | - |
| 3l | - | - | - | - |
| 3m | 200 | 200 | 100 | - |
| 3n | - | - | - | - |
| 3o | - | - | - | - |
| 3p | - | - | - | - |
| 3q | - | - | - | - |
| 3r | - | - | - | - |
| 3s | - | 50 | 50 | 50 |
| 3t | - | 100 | 50 | - |
| 3u | - | 200 | 100 | - |
| 3v | 200 | 50 | 50 | 100 |
| 3w | - | - | - | - |
| 3x | - | - | 200 | - |
| 3y | - | - | - | - |
| 3z | - | - | 200 | - |
| 3aa | - | 200 | 100 | - |
| 3ab | - | 100 | - | - |
| chloramphenicol | 2.5 | 1.25 | 0.25 | 2.5 |

[a]Potential is defined as the presence of a zone of inhibition at the indicated μg of compound. [b]"-" indicates that no zone of inhibition was observed up to the highest amount (200 μg) tested.

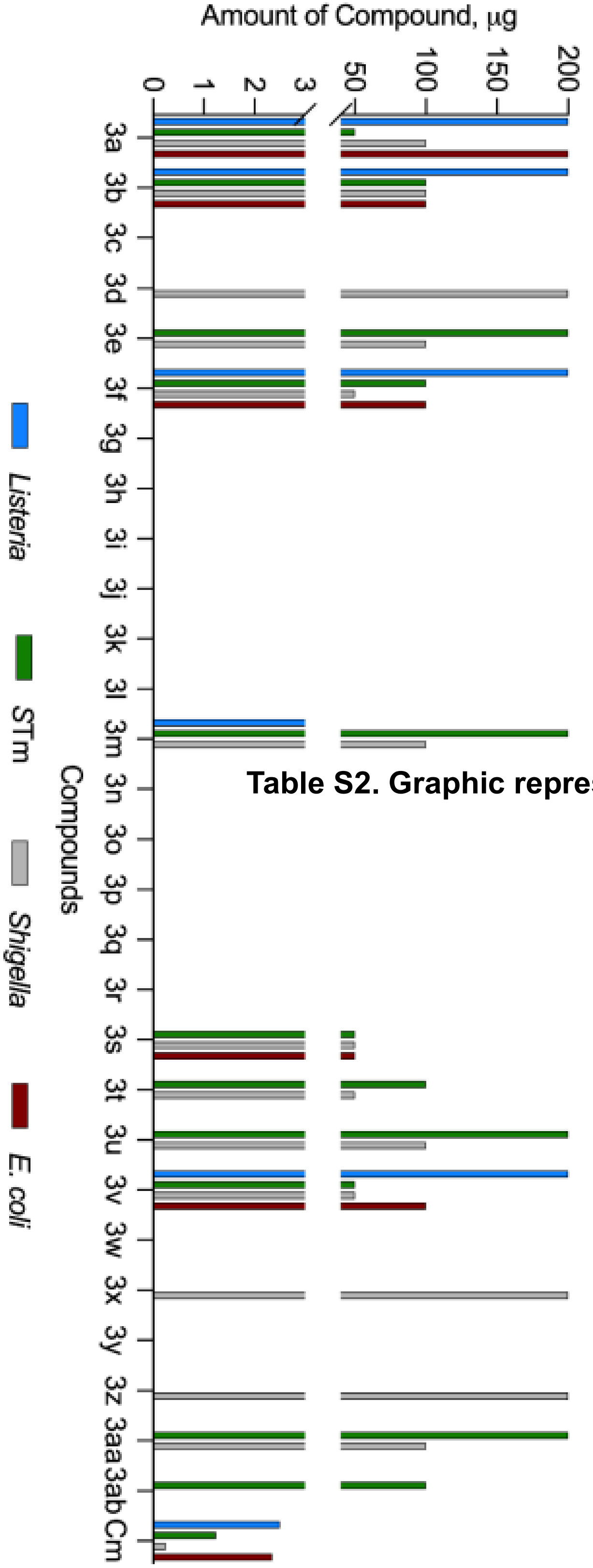


**Table S2. Graphic representation of biological data**

## Computational Data

**General Methods**:
The quantum chemistry method of meta-hybrid density functional theory (DFT)[23] was carried out at the Center for Computational Sciences (CCS) at Duquesne University using Gaussian 16.[24] The M06-2X functional[25] with Dunning's maug-cc-pv[D,T,Q]z basis sets[26] were used to calculate electronic, enthalpic, and free energies for both ground and transition structures. These energies were then extrapolated to the complete basis set (CBS) limit, which is not a basis set, but rather an extrapolated estimate of a result using an infinitely large basis set.[27] The procedure removes any error from the linear combination of atomic orbitals approximation. Unrestricted M06-2X was used for triplet state calculations, and the spin expectation value of these calculations are given. The use of M06-2X, developed by Truhlar and co-workers, has been reported to be accurate to within 1.2 kcal/mol for reaction barriers and within 0.37 kcal/mol of non-covalent interaction energies.[25] Vibrational frequency calculations were used to confirm all stationary points as either minima or transition structures and to provide thermodynamic corrections for enthalpies and free energies. The free volume method developed by Whitesides *et. al.*[28] was used to correct for translational entropy. A standard state of 1 M was assumed for all species.

**Table S3.** Absolute energies (Hartree) and entropies (cal/mol K) for the stationary points involved in the [3+2] cycloaddition or competing biradical addition mechanism using M06-2x and the listed basis sets with PCM (THF). The free volume correction to the entropy and free energy are shown. The coordinated ground state refers to a ground state containing both the azomethine ylide and *trans*-azobenzene. Free volume corrections are applied assuming 1 M species concentration. Energies are extrapolated to the complete basis set (CBS) limit.

| Species | $E_{elec}$ | $E_{298}$ | $H_{298}$ | $S_{298}$ | $G_{298}$ | $S_{298, FV}$ | $G_{298, FV}$ |
|---|---|---|---|---|---|---|---|
| *Singlet state* | | | | | | | |
| **Azomethine ylide** | | | | | | | |
| maug-cc-pvDz | -173.125552214 | -173.024571 | -173.023627 | 70.670 | -173.057204 | 51.811 | -173.048244 |
| maug-cc-pvTz | -173.178488699 | -173.077031 | -173.076087 | 70.442 | -173.109556 | 51.584 | -173.100596 |
| maug-cc-pvQz | -173.190091993 | -173.088618 | -173.087674 | 70.533 | -173.121186 | 51.675 | -173.112226 |
| maug CBS | -173.193349337 | -173.091903 | -173.090959 | 70.609 | -173.124507 | 51.752 | -173.115548 |
| ***trans*-azobenzene** | | | | | | | |
| maug-cc-pvDz | -572.594400813 | -572.391781 | -572.390836 | 107.606 | -572.441963 | 88.748 | -572.433003 |
| maug-cc-pvTz | -572.722257927 | -572.519201 | -572.518257 | 106.895 | -572.569046 | 88.037 | -572.560086 |
| maug-cc-pvQz | -572.756237755 | -572.553130 | -572.552186 | 106.787 | -572.602924 | 87.930 | -572.593964 |
| maug CBS | -572.768537095 | -572.565443 | -572.564499 | 106.789 | -572.615238 | 87.931 | -572.606278 |
| ***cis*-azobenzene** | | | | | | | |
| maug-cc-pvDz | -572.576189039 | -572.373891 | -572.372946 | 104.289 | -572.422497 | 85.431 | -572.413537 |
| maug-cc-pvTz | -572.704688440 | -572.501906 | -572.500962 | 104.136 | -572.550440 | 85.278 | -572.541480 |
| maug-cc-pvQz | -572.738683177 | -572.535874 | -572.534929 | 104.095 | -572.584388 | 85.238 | -572.575428 |
| maug CBS | -572.750911591 | -572.548143 | -572.547197 | 104.081 | -572.596649 | 85.224 | -572.587689 |
| **Coordinated ground state** | | | | | | | |

| | | | | | | | |
|---|---|---|---|---|---|---|---|
| maug-cc-pvDz | -745.735459184 | -745.428692 | -745.427748 | 133.176 | -745.491024 | 114.318 | -745.482064 |
| maug-cc-pvTz | -745.914731865 | -745.606973 | -745.606029 | 132.655 | -745.669058 | 113.798 | -745.660098 |
| maug-cc-pvQz | -745.959959859 | -745.652185 | -745.651241 | 132.782 | -745.714329 | 113.922 | -745.705369 |
| maug CBS | -745.975220228 | -745.667546 | -745.666602 | 132.939 | -745.729766 | 114.082 | -745.720806 |
| **Transition state – [3+2] cycloaddition** | | | | | | | |
| maug-cc-pvDz | -745.733956727 | -745.427457 | -745.426513 | 125.593 | -745.486186 | 106.735 | -745.477226 |
| maug-cc-pvTz | -745.913066948 | -745.605573 | -745.604629 | 125.222 | -745.664126 | 106.365 | -745.655166 |
| maug-cc-pvQz | -745.958247084 | -745.650687 | -745.649743 | 124.933 | -745.709103 | 106.076 | -745.700143 |
| maug CBS | -745.973488205 | -745.665990 | -745.665046 | 124.748 | -745.724317 | 105.890 | -745.715357 |
| **1,2,4-triazolidine (3a)** | | | | | | | |
| maug-cc-pvDz | -745.811515493 | -745.500115 | -745.499171 | 123.257 | -745.557734 | 104.399 | -745.548774 |
| maug-cc-pvTz | -745.989814442 | -745.677351 | -745.676407 | 122.738 | -745.734723 | 103.879 | -745.725763 |
| maug-cc-pvQz | -746.035454847 | -745.722993 | -745.722049 | 122.535 | -745.780269 | 103.677 | -745.771309 |
| maug CBS | -746.051157164 | -745.738823 | -745.737879 | 122.432 | -745.796051 | 103.575 | -745.787091 |
| *Triplet state* | | | | | | | |
| **Azomethine ylide** | | | | | | | |
| maug-cc-pvDz | -173.074330993 | -172.975826 | -172.974882 | 73.458 | -173.009784 | 54.600 | -173.000824 |
| maug-cc-pvTz | -173.126622494 | -173.027870 | -173.026926 | 73.663 | -173.061925 | 54.804 | -173.052965 |
| maug-cc-pvQz | -173.137844204 | -173.039058 | -173.038113 | 73.609 | -173.073088 | 54.754 | -173.064128 |
| maug CBS | -173.140910370 | -173.042122 | -173.041176 | 73.564 | -173.076129 | 54.707 | -173.067169 |
| **Coordinated ground state** | | | | | | | |
| maug-cc-pvDz | -745.688496512 | -745.383606 | -745.382662 | 138.056 | -745.448257 | 119.199 | -745.439297 |
| maug-cc-pvTz | -745.867979334 | -745.562278 | -745.561334 | 138.599 | -745.627187 | 119.742 | -745.618227 |
| maug-cc-pvQz | -745.912790919 | -745.607015 | -745.606071 | 138.613 | -745.671930 | 119.755 | -745.662970 |
| maug CBS | -745.927701884 | -745.621958 | -745.621014 | 138.561 | -745.686849 | 119.704 | -745.677889 |
| **Transition state – single bond formation** | | | | | | | |
| maug-cc-pvDz | -745.688364950 | -745.384687 | -745.383743 | 134.092 | -745.447454 | 115.234 | -745.438494 |
| maug-cc-pvTz | -745.867945540 | -745.563250 | -745.562306 | 132.958 | -745.625479 | 114.101 | -745.616519 |
| maug-cc-pvQz | -745.912775274 | -745.608063 | -745.607118 | 132.755 | -745.670195 | 113.899 | -745.661235 |
| maug CBS | -745.927689503 | -745.623078 | -745.622132 | 132.726 | -745.685194 | 113.868 | -745.676234 |
| **Stepwise addition intermediate** | | | | | | | |
| maug-cc-pvDz | -745.725279034 | -745.417721 | -745.416777 | 131.183 | -745.479107 | 112.327 | -745.470147 |
| maug-cc-pvTz | -745.903935598 | -745.595472 | -745.594528 | 131.558 | -745.657035 | 112.700 | -745.648075 |
| maug-cc-pvQz | -745.949348538 | -745.640849 | -745.639905 | 131.578 | -745.702422 | 112.721 | -745.693462 |
| maug CBS | -745.964826466 | -745.656404 | -745.655460 | 131.551 | -745.717964 | 112.694 | -745.709004 |

**Table S4.** Relative energies (Hartree) and entropies (cal/mol K) for all the stationary points along the reaction pathway using M06-2x and the listed basis sets with PCM (THF) compared to the singlet separated ground state consisting of infinitely separated azomethine ylide and *trans*-azobenzene. The coordinated ground state refers to a ground state containing both the azomethine ylide and *trans*-azobenzene. The free volume correction to the entropy and free energy are shown.

| Species | $E_{elec}$ | $E_{298}$ | $H_{298}$ | $S_{298}$ | $G_{298}$ | $S_{298, FV}$ | $G_{298, FV}$ |
|---|---|---|---|---|---|---|---|
| ***Singlet states*** | | | | | | | |
| **Coordinated ground state** | | | | | | | |
| maug-cc-pvDz | -9.7 | -7.7 | -8.3 | -45.100 | 5.1 | -26.242 | -0.5 |
| maug-cc-pvTz | -8.8 | -6.7 | -7.3 | -44.682 | 6.0 | -25.823 | 0.4 |
| maug-cc-pvQz | -8.6 | -6.5 | -7.1 | -44.538 | 6.1 | -25.682 | 0.5 |
| maug CBS | -8.4 | -6.4 | -7.0 | -44.459 | 6.3 | -25.602 | 0.6 |
| **Transition state – [3+2] cycloaddition** | | | | | | | |
| maug-cc-pvDz | -8.8 | -7.0 | -7.6 | -52.683 | 8.1 | -33.825 | 2.5 |
| maug-cc-pvTz | -7.7 | -5.9 | -6.5 | -52.115 | 9.1 | -33.256 | 3.5 |
| maug-cc-pvQz | -7.5 | -5.6 | -6.2 | -52.387 | 9.4 | -33.528 | 3.8 |
| maug CBS | -7.3 | -5.4 | -6.0 | -52.650 | 9.7 | -33.793 | 4.1 |
| **1,2,4-triazolidine (3a)** | | | | | | | |
| maug-cc-pvDz | -57.5 | -52.6 | -53.2 | -55.019 | -36.8 | -36.161 | -42.4 |
| maug-cc-pvTz | -55.9 | -50.9 | -51.5 | -54.599 | -35.2 | -35.742 | -40.8 |
| maug-cc-pvQz | -55.9 | -51.0 | -51.6 | -54.785 | -35.2 | -35.927 | -40.9 |
| maug CBS | -56.0 | -51.1 | -51.7 | -54.966 | -35.3 | -36.108 | -41.0 |
| ***Triple states*** | | | | | | | |
| **Separated ground state** | | | | | | | |
| maug-cc-pvDz | 32.1 | 30.6 | 30.6 | 2.788 | 29.8 | 2.789 | 29.8 |
| maug-cc-pvTz | 32.5 | 30.8 | 30.8 | 3.221 | 29.9 | 3.220 | 29.9 |
| maug-cc-pvQz | 32.8 | 31.1 | 31.1 | 3.076 | 30.2 | 3.079 | 30.2 |
| maug CBS | 32.9 | 31.2 | 31.2 | 2.955 | 30.4 | 2.955 | 30.4 |
| **Coordinated ground state** | | | | | | | |
| maug-cc-pvDz | 19.7 | 20.5 | 20.0 | -40.220 | 31.9 | -21.361 | 26.3 |
| maug-cc-pvTz | 20.6 | 21.3 | 20.7 | -38.738 | 32.3 | -19.879 | 26.6 |
| maug-cc-pvQz | 21.0 | 21.8 | 21.2 | -38.707 | 32.7 | -19.850 | 27.1 |
| maug CBS | 21.5 | 22.2 | 21.6 | -38.837 | 33.2 | -19.979 | 27.6 |
| **Transition state – single bond formation** | | | | | | | |
| maug-cc-pvDz | 19.8 | 19.9 | 19.3 | -44.184 | 32.5 | -25.326 | 26.8 |
| maug-cc-pvTz | 20.6 | 20.7 | 20.1 | -44.379 | 33.3 | -25.520 | 27.7 |
| maug-cc-pvQz | 21.1 | 21.1 | 20.5 | -44.565 | 33.8 | -25.705 | 28.2 |
| maug CBS | 21.5 | 21.5 | 20.9 | -44.672 | 34.2 | -25.815 | 28.6 |
| **Stepwise addition intermediate** | | | | | | | |
| maug-cc-pvDz | -3.3 | -0.9 | -1.5 | -47.093 | 12.6 | -28.233 | 7.0 |
| maug-cc-pvTz | -2.0 | 0.5 | -0.1 | -45.779 | 13.5 | -26.921 | 7.9 |
| maug-cc-pvQz | -1.9 | 0.6 | 0.0 | -45.742 | 13.6 | -26.884 | 8.0 |
| maug CBS | -1.8 | 0.6 | 0.0 | -45.847 | 13.7 | -26.989 | 8.0 |

## Calculated Energies for Transition and Ground State Structures in the Singlet State

### Azomethine ylide

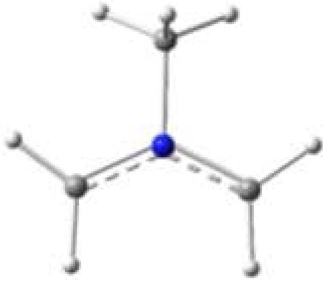

Figure S82. Representative ball and stick model for azomethine ylide.

### Azomethine ylide, maug-cc-pvDz

# m062x/maug-cc-pvdz opt=(calcfc,noeigen) optcyc=50 freq scrf=(solvent=thf,pcm) nosymm

EE = -173.125552214 (Hartree)
ZPE = -173.029914
$E_{298}$ = -173.024571
$H_{298}$ = -173.023627
$G_{298}$ = -173.057204
S = 70.670 (cal/mol·K)

| | | | |
|---|---|---|---|
| 7 | 1.007574 | 1.885935 | -0.577941 |
| 6 | 1.180839 | 1.981552 | 0.740993 |
| 6 | 2.241401 | 1.832781 | -1.385735 |
| 6 | -0.136315 | 1.887982 | -1.263710 |
| 1 | 2.545745 | 2.857487 | -1.630871 |
| 1 | 3.025472 | 1.336330 | -0.805904 |
| 1 | 2.043020 | 1.269825 | -2.302858 |
| 1 | 2.191189 | 1.963037 | 1.131532 |
| 1 | 0.304305 | 2.086460 | 1.374325 |
| 1 | -0.091703 | 1.800619 | -2.342600 |
| 1 | -1.069976 | 1.988725 | -0.717218 |

**Azomethine ylide, maug-cc-pvTz**

# m062x/maug-cc-pvtz opt=(calcfc,noeigen) optcyc=50 freq scrf=(solvent=thf,pcm) nosymm

EE = -173.178488699 (Hartree)
ZPE = -173.082286
$E_{298}$ = -173.077031
$H_{298}$ = -173.076087
$G_{298}$ = -173.109556
S = 70.442 (cal/mol·K)

| | | | |
|---|---|---|---|
| 7 | 1.007018 | 1.887025 | -0.577583 |
| 6 | 1.179455 | 1.981656 | 0.736184 |
| 6 | 2.241068 | 1.832292 | -1.385423 |
| 6 | -0.132281 | 1.889181 | -1.260707 |
| 1 | 2.542233 | 2.847538 | -1.628593 |
| 1 | 3.018143 | 1.340764 | -0.810102 |
| 1 | 2.043870 | 1.274235 | -2.294329 |
| 1 | 2.179484 | 1.964393 | 1.125823 |
| 1 | 0.311490 | 2.083370 | 1.364851 |
| 1 | -0.090714 | 1.803653 | -2.329868 |
| 1 | -1.058217 | 1.986625 | -0.720240 |

**Azomethine ylide, maug-cc-pvQz**

# m062x/maug-cc-pvqz opt=(calcfc,noeigen) optcyc=50 freq scrf=(solvent=thf,pcm) nosymm

EE = -173.190091993 (Hartree)
ZPE = -173.093878
$E_{298}$ = -173.088618
$H_{298}$ = -173.087674
$G_{298}$ = -173.121186
S = 70.533 (cal/mol·K)

| | | | |
|---|---|---|---|
| 7 | 1.007120 | 1.887039 | -0.577647 |
| 6 | 1.179242 | 1.981949 | 0.734885 |
| 6 | 2.240029 | 1.832258 | -1.384781 |
| 6 | -0.131200 | 1.889917 | -1.259980 |
| 1 | 2.541250 | 2.846429 | -1.627975 |
| 1 | 3.017262 | 1.341685 | -0.810693 |
| 1 | 2.043963 | 1.275161 | -2.293359 |

| | | | |
|---|---|---|---|
| 1 | 2.178255 | 1.962880 | 1.124613 |
| 1 | 0.312465 | 2.083448 | 1.363708 |
| 1 | -0.090075 | 1.802860 | -2.328152 |
| 1 | -1.056761 | 1.987106 | -0.720606 |

***trans*-Azobenzene**

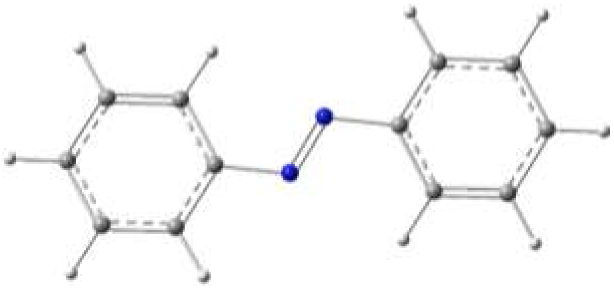

Figure S83. Representative ball and stick model for *trans*-azobenzene.

***trans*-Azobenzene, maug-cc-pvDz**

# m062x/maug-cc-pvdz opt=(calcfc,z-matrix,noeigen) optcyc=50 freq scrf=(solvent=thf,pcm) nosymm

EE = -572.594400813 (Hartree)
ZPE = -572.402719
$E_{298}$ = -572.391781
$H_{298}$ = -572.390836
$G_{298}$ = -572.441963
S = 107.606 (cal/mol·K)

| | | | |
|---|---|---|---|
| 7 | 0.000000 | 0.000000 | 0.000000 |
| 7 | 0.000000 | 0.000000 | 1.243134 |
| 6 | 1.294818 | 0.000000 | -0.600239 |
| 6 | 1.305067 | 0.040142 | -1.996913 |
| 6 | 2.518097 | 0.043522 | -2.685401 |
| 6 | 3.718012 | 0.003809 | -1.975624 |
| 6 | 3.704318 | -0.038885 | -0.576204 |
| 6 | 2.498938 | -0.040888 | 0.117359 |
| 6 | -1.294807 | -0.006060 | 1.843363 |
| 6 | -2.498692 | -0.054505 | 1.125824 |
| 6 | -3.704068 | -0.058175 | 1.819395 |
| 6 | -3.718006 | -0.013638 | 3.218752 |
| 6 | -2.518319 | 0.033610 | 3.928471 |
| 6 | -1.305288 | 0.035864 | 3.239984 |

| | | | |
|---|---|---|---|
| 1 | 0.351678 | 0.069291 | -2.526447 |
| 1 | 2.525254 | 0.076009 | -3.775350 |
| 1 | 4.668977 | 0.004932 | -2.510114 |
| 1 | 4.644844 | -0.071832 | -0.024606 |
| 1 | 2.474753 | -0.074982 | 1.205820 |
| 1 | -2.474355 | -0.090027 | 0.037409 |
| 1 | -4.644398 | -0.097098 | 1.267845 |
| 1 | -4.668962 | -0.017048 | 3.753248 |
| 1 | -2.525660 | 0.067536 | 5.018375 |
| 1 | -0.352075 | 0.070868 | 3.769486 |

***trans*-Azobenzene, maug-cc-pvTz**

#m062x/maug-cc-pvtz opt=(calcfc,z-matrix,noeigen) optcyc=50 freq scrf=(pcm,solvent=THF) nosymm

EE = -572.722257927 (Hartree)
ZPE = -572.530086
$E_{298}$ = -572.519201
$H_{298}$ = -572.518257
$G_{298}$ = -572.569046
S = 106.895 (cal/mol·K)

| | | | |
|---|---|---|---|
| 7 | 0.000000 | 0.000000 | 0.000000 |
| 7 | 0.000000 | 0.000000 | 1.236647 |
| 6 | 1.289603 | 0.000000 | -0.606457 |
| 6 | 1.295665 | 0.080970 | -1.993945 |
| 6 | 2.499753 | 0.089052 | -2.683575 |
| 6 | 3.695295 | 0.010485 | -1.983836 |
| 6 | 3.686159 | -0.075809 | -0.593416 |
| 6 | 2.489466 | -0.081106 | 0.100431 |
| 6 | -1.289553 | -0.008510 | 1.843163 |
| 6 | -2.489086 | -0.093100 | 1.136099 |
| 6 | -3.685714 | -0.095900 | 1.830071 |
| 6 | -3.695131 | -0.014223 | 3.220770 |
| 6 | -2.499915 | 0.067929 | 3.920661 |
| 6 | -1.295882 | 0.068162 | 3.230889 |
| 1 | 0.349196 | 0.138730 | -2.514292 |
| 1 | 2.503846 | 0.154650 | -3.762625 |
| 1 | 4.635976 | 0.014031 | -2.517170 |
| 1 | 4.620093 | -0.140644 | -0.052075 |
| 1 | 2.469773 | -0.149202 | 1.177958 |

| | | | |
|---|---|---|---|
| 1 | -2.469203 | -0.157418 | 0.058341 |
| 1 | -4.619386 | -0.163333 | 1.288591 |
| 1 | -4.635767 | -0.016998 | 3.754191 |
| 1 | -2.504227 | 0.130043 | 4.999917 |
| 1 | -0.349656 | 0.129077 | 3.751323 |

***trans*-Azobenzene, maug-cc-pvQz**

# m062x/maug-cc-pvqz opt=(calcfc,z-matrix,noeigen) optcyc=50 freq
scrf=(solvent=thf,pcm) nosymm

EE = -572.756237755 (Hartree)
ZPE = -572.563997
$E_{298}$ = -572.553130
$H_{298}$ = -572.552186
$G_{298}$ = -572.602924
S = 106.787 (cal/mol·K)

| | | | |
|---|---|---|---|
| 7 | 0.000000 | 0.000000 | 0.000000 |
| 7 | 0.000000 | 0.000000 | 1.235384 |
| 6 | 1.287752 | 0.000000 | -0.607951 |
| 6 | 1.292621 | 0.067926 | -1.995658 |
| 6 | 2.495858 | 0.074506 | -2.685873 |
| 6 | 3.691764 | 0.008309 | -1.986562 |
| 6 | 3.683948 | -0.064165 | -0.595820 |
| 6 | 2.488146 | -0.068302 | 0.098475 |
| 6 | -1.287718 | -0.007652 | 1.843373 |
| 6 | -2.487866 | -0.078975 | 1.136813 |
| 6 | -3.683610 | -0.082381 | 1.831206 |
| 6 | -3.691628 | -0.014524 | 3.222184 |
| 6 | -2.495972 | 0.054840 | 3.921615 |
| 6 | -1.292784 | 0.056024 | 3.231282 |
| 1 | 0.346459 | 0.116500 | -2.515740 |
| 1 | 2.498990 | 0.129520 | -3.764665 |
| 1 | 4.631302 | 0.011052 | -2.520195 |
| 1 | 4.617822 | -0.118866 | -0.054968 |
| 1 | 2.469012 | -0.125524 | 1.175793 |
| 1 | -2.468592 | -0.132534 | 0.059308 |
| 1 | -4.617282 | -0.139378 | 1.290242 |
| 1 | -4.631121 | -0.017769 | 3.755893 |
| 1 | -2.499257 | 0.106369 | 5.000579 |
| 1 | -0.346805 | 0.107312 | 3.751441 |

***cis*-Azobenzene**

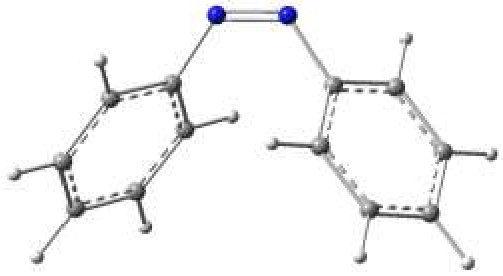

Figure S84. Representative ball and stick model for *cis*-azobenzene.

***cis*-Azobenzene, maug-cc-pvDz**

# m062x/maug-cc-pvdz opt=(calcfc,noeigen) optcyc=50 freq scrf=(solvent=thf,pcm) nosymm

EE = -572.576189039 (Hartree)
ZPE = -572.384640
$E_{298}$ = -572.373891
$H_{298}$ = -572.372946
$G_{298}$ = -572.422497
S = 104.289 (cal/mol·K)

| | | | |
|---|---|---|---|
| 7 | -0.099170 | -0.032413 | 0.000177 |
| 7 | -0.097039 | 0.030484 | 1.237985 |
| 6 | 1.115542 | -0.042388 | -0.774602 |
| 6 | 1.211452 | -0.999325 | -1.786424 |
| 6 | 2.329996 | -1.010268 | -2.618694 |
| 6 | 3.320451 | -0.036946 | -2.474025 |
| 6 | 3.191345 | 0.947954 | -1.491354 |
| 6 | 2.093847 | 0.947273 | -0.633012 |
| 6 | 1.114658 | -0.053867 | 2.012953 |
| 6 | 1.289800 | 0.898852 | 3.018133 |
| 6 | 2.406808 | 0.823915 | 3.849036 |
| 6 | 3.314002 | -0.228153 | 3.710133 |
| 6 | 3.103280 | -1.205868 | 2.734498 |
| 6 | 2.008399 | -1.121118 | 1.877049 |
| 1 | 2.418998 | -1.772187 | -3.394003 |
| 1 | 3.952506 | 1.722519 | -1.389565 |
| 1 | 2.559448 | 1.581297 | 4.618829 |
| 1 | 3.798274 | -2.040834 | 2.637317 |
| 1 | 1.990947 | 1.712094 | 0.137681 |

| | | | |
|---|---|---|---|
| 1 | 4.186832 | -0.036245 | -3.136484 |
| 1 | 0.413513 | -1.734215 | -1.903806 |
| 1 | 1.841806 | -1.879648 | 1.111202 |
| 1 | 4.178448 | -0.295434 | 4.371657 |
| 1 | 0.555310 | 1.697815 | 3.131037 |

**_cis_-Azobenzene, maug-cc-pvTz**

# m062x/maug-cc-pvtz opt=(calcfc,noeigen) optcyc=50 freq scrf=(solvent=thf,pcm) nosymm

EE = -572.704688440 (Hartree)
ZPE = -572.512587
$E_{298}$ = -572.501906
$H_{298}$ = -572.500962
$G_{298}$ = -572.550440
S = 104.136 (cal/mol·K)

| | | | |
|---|---|---|---|
| 7 | -0.088892 | -0.030398 | 0.003376 |
| 7 | -0.086833 | 0.029291 | 1.234684 |
| 6 | 1.121547 | -0.039798 | -0.773182 |
| 6 | 1.223841 | -0.997896 | -1.773146 |
| 6 | 2.336295 | -1.006253 | -2.601345 |
| 6 | 3.315107 | -0.030226 | -2.463496 |
| 6 | 3.180323 | 0.954486 | -1.491848 |
| 6 | 2.088294 | 0.951150 | -0.637942 |
| 6 | 1.120499 | -0.056196 | 2.011423 |
| 6 | 1.301774 | 0.895981 | 3.005828 |
| 6 | 2.412342 | 0.818249 | 3.832826 |
| 6 | 3.308039 | -0.235127 | 3.699664 |
| 6 | 3.092017 | -1.211089 | 2.733940 |
| 6 | 2.002894 | -1.123410 | 1.880842 |
| 1 | 2.430237 | -1.765707 | -3.365142 |
| 1 | 3.930176 | 1.727557 | -1.395767 |
| 1 | 2.569154 | 1.571843 | 4.592091 |
| 1 | 3.776120 | -2.043338 | 2.641671 |
| 1 | 1.980552 | 1.712959 | 0.121904 |
| 1 | 4.173619 | -0.027550 | -3.120501 |
| 1 | 0.437452 | -1.732299 | -1.884803 |
| 1 | 1.832140 | -1.877727 | 1.124983 |
| 1 | 4.164413 | -0.304197 | 4.355827 |
| 1 | 0.578340 | 1.692987 | 3.113831 |

***cis*-Azobenzene, maug-cc-pvQz**

# m062x/maug-cc-pvqz opt=(calcfc,noeigen) optcyc=50 freq scrf=(solvent=thf,pcm) nosymm

EE = -572.738683177 (Hartree)
ZPE = -572.546544
$E_{298}$ = -572.535874
$H_{298}$ = -572.534929
$G_{298}$ = -572.584388
S = 104.095 (cal/mol·K)

| | | | |
|---|---|---|---|
| 7 | -0.083964 | -0.031873 | 0.004082 |
| 7 | -0.081852 | 0.029661 | 1.234005 |
| 6 | 1.123896 | -0.041581 | -0.773996 |
| 6 | 1.224311 | -0.998334 | -1.774925 |
| 6 | 2.333885 | -1.004987 | -2.606122 |
| 6 | 3.312216 | -0.028907 | -2.470169 |
| 6 | 3.180063 | 0.953799 | -1.496921 |
| 6 | 2.090839 | 0.948727 | -0.640181 |
| 6 | 1.122948 | -0.054888 | 2.012284 |
| 6 | 1.302416 | 0.896555 | 3.007208 |
| 6 | 2.410027 | 0.817600 | 3.837163 |
| 6 | 3.305107 | -0.235949 | 3.706334 |
| 6 | 3.091709 | -1.210522 | 2.739431 |
| 6 | 2.005484 | -1.121587 | 1.883514 |
| 1 | 2.425902 | -1.762863 | -3.370503 |
| 1 | 3.929298 | 1.726400 | -1.401957 |
| 1 | 2.564987 | 1.570116 | 4.596653 |
| 1 | 3.775124 | -2.042369 | 2.648584 |
| 1 | 1.985044 | 1.708576 | 0.120690 |
| 1 | 4.167985 | -0.024910 | -3.129340 |
| 1 | 0.438737 | -1.732477 | -1.885522 |
| 1 | 1.836692 | -1.874382 | 1.126917 |
| 1 | 4.158659 | -0.305936 | 4.364661 |
| 1 | 0.579936 | 1.693424 | 3.113830 |

**Coordinated ground state (azomethine ylide and *trans*-azobenzene)**

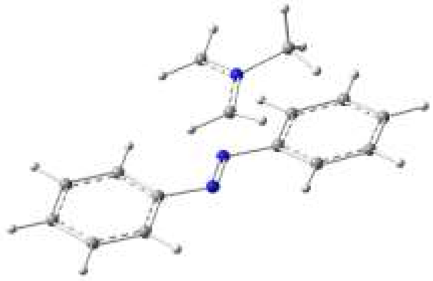

Figure S85. Representative ball and stick model for the coordinated ground state of azomethine ylide and azobenzene.

**Coordinated ground state, maug-cc-pvDz**

# m062x/maug-cc-pvdz opt=(calcfc,noeigen) optcyc=50 freq scrf=(solvent=thf,pcm) nosymm

EE = -745.735459184 (Hartree)
ZPE = -745.445786
$E_{298}$ = -745.428692
$H_{298}$ = -745.427748
$G_{298}$ = -745.491024
S = 133.176 (cal/mol·K)

| | | | |
|---|---|---|---|
| 7 | 1.023061 | 1.908170 | -0.578385 |
| 6 | 1.170891 | 1.894773 | 0.746613 |
| 6 | 2.250389 | 1.849966 | -1.395298 |
| 6 | -0.127466 | 1.941240 | -1.250533 |
| 1 | 2.410316 | 2.833295 | -1.853971 |
| 1 | 3.096933 | 1.587512 | -0.754683 |
| 1 | 2.125264 | 1.086694 | -2.173214 |
| 1 | 2.174136 | 1.857964 | 1.154840 |
| 1 | 0.289749 | 2.029877 | 1.367230 |
| 1 | -0.089657 | 1.933864 | -2.334424 |
| 1 | -1.052814 | 2.064093 | -0.694704 |
| 7 | -0.313642 | -0.564823 | 0.809417 |
| 7 | -0.415160 | -0.722337 | -0.429067 |
| 6 | -1.509059 | -0.097991 | 1.431652 |
| 6 | 0.796225 | -1.147516 | -1.050130 |
| 6 | -1.450733 | 0.060317 | 2.820658 |
| 6 | -2.563183 | 0.524797 | 3.519679 |

| | | | |
|---|---|---|---|
| 6 | -3.737699 | 0.837199 | 2.831410 |
| 6 | -3.794573 | 0.681021 | 1.443056 |
| 6 | -2.687180 | 0.214859 | 0.738034 |
| 6 | 2.012625 | -1.288143 | -0.368685 |
| 6 | 3.152194 | -1.675682 | -1.074278 |
| 6 | 3.085752 | -1.922456 | -2.447176 |
| 6 | 1.867022 | -1.790872 | -3.122002 |
| 6 | 0.725936 | -1.403821 | -2.424880 |
| 1 | -0.519230 | -0.183704 | 3.334578 |
| 1 | -2.513865 | 0.645127 | 4.602615 |
| 1 | -4.609610 | 1.202855 | 3.375613 |
| 1 | -4.712636 | 0.924670 | 0.906096 |
| 1 | -2.718799 | 0.085725 | -0.343355 |
| 1 | 2.047475 | -1.087535 | 0.701069 |
| 1 | 4.102144 | -1.783592 | -0.548017 |
| 1 | 3.982021 | -2.222123 | -2.992382 |
| 1 | 1.810001 | -1.988095 | -4.193239 |
| 1 | -0.233472 | -1.279772 | -2.930197 |

**Coordinated ground state, maug-cc-pvTz**

#m062x/maug-cc-pvtz opt=(calcfc,noeigen) optcyc=50 freq scrf=(pcm,solvent=THF) nosymm

EE = -745.914731865 (Hartree)
ZPE = -745.623923
$E_{298}$ = -745.606973
$H_{298}$ = -745.606029
$G_{298}$ = -745.669058
S = 132.655 (cal/mol·K)

| | | | |
|---|---|---|---|
| 7 | 1.019301 | 1.903202 | -0.579242 |
| 6 | 1.165467 | 1.897100 | 0.740559 |
| 6 | 2.247566 | 1.836965 | -1.394107 |
| 6 | -0.124802 | 1.934222 | -1.251410 |
| 1 | 2.418786 | 2.812841 | -1.840945 |
| 1 | 3.081639 | 1.560335 | -0.759746 |
| 1 | 2.114762 | 1.087644 | -2.170575 |
| 1 | 2.157903 | 1.860104 | 1.148993 |
| 1 | 0.292485 | 2.032042 | 1.355733 |
| 1 | -0.087278 | 1.912745 | -2.325228 |
| 1 | -1.044279 | 2.053533 | -0.704019 |

| | | | |
|---|---|---|---|
| 7 | -0.316591 | -0.566057 | 0.809233 |
| 7 | -0.412149 | -0.728514 | -0.422155 |
| 6 | -1.509194 | -0.098243 | 1.431021 |
| 6 | 0.797343 | -1.149521 | -1.044586 |
| 6 | -1.451893 | 0.058106 | 2.812790 |
| 6 | -2.557882 | 0.521680 | 3.508258 |
| 6 | -3.724955 | 0.835896 | 2.822832 |
| 6 | -3.780829 | 0.683069 | 1.441136 |
| 6 | -2.679454 | 0.217843 | 0.740574 |
| 6 | 2.008905 | -1.286006 | -0.369514 |
| 6 | 3.143305 | -1.664543 | -1.074008 |
| 6 | 3.076025 | -1.906134 | -2.440544 |
| 6 | 1.862210 | -1.777481 | -3.109993 |
| 6 | 0.727081 | -1.398984 | -2.413103 |
| 1 | -0.528952 | -0.185718 | 3.322360 |
| 1 | -2.509827 | 0.640019 | 4.581985 |
| 1 | -4.588900 | 1.199602 | 3.362022 |
| 1 | -4.689783 | 0.928588 | 0.908654 |
| 1 | -2.710459 | 0.092738 | -0.331843 |
| 1 | 2.046182 | -1.088855 | 0.691255 |
| 1 | 4.086394 | -1.768958 | -0.554307 |
| 1 | 3.964847 | -2.198214 | -2.983042 |
| 1 | 1.805385 | -1.969183 | -4.172661 |
| 1 | -0.225004 | -1.276307 | -2.912436 |

**Coordinated ground state, maug-cc-pvQz**

# m062x/maug-cc-pvqz opt=(calcfc,noeigen) optcyc=50 freq scrf=(solvent=thf,pcm) nosymm

EE = -745.959959859 (Hartree)
ZPE = -745.669143
$E_{298}$ = -745.652185
$H_{298}$ = -745.651241
$G_{298}$ = -745.714329
S = 132.782 (cal/mol·K)

| | | | |
|---|---|---|---|
| 7 | 1.020160 | 1.901938 | -0.580694 |
| 6 | 1.168369 | 1.896566 | 0.737632 |
| 6 | 2.246123 | 1.830926 | -1.396353 |
| 6 | -0.123711 | 1.936828 | -1.250734 |
| 1 | 2.411965 | 2.800470 | -1.856299 |

| | | | |
|---|---|---|---|
| 1 | 3.082361 | 1.567898 | -0.760791 |
| 1 | 2.116927 | 1.071242 | -2.162045 |
| 1 | 2.160244 | 1.855500 | 1.144479 |
| 1 | 0.297502 | 2.030834 | 1.354436 |
| 1 | -0.088127 | 1.915178 | -2.323686 |
| 1 | -1.042011 | 2.055938 | -0.703043 |
| 7 | -0.317921 | -0.566278 | 0.810114 |
| 7 | -0.411847 | -0.730466 | -0.419536 |
| 6 | -1.509215 | -0.098489 | 1.431851 |
| 6 | 0.796481 | -1.149897 | -1.042587 |
| 6 | -1.452804 | 0.057373 | 2.813215 |
| 6 | -2.558978 | 0.520603 | 3.507705 |
| 6 | -3.725040 | 0.835086 | 2.821788 |
| 6 | -3.779840 | 0.683200 | 1.440417 |
| 6 | -2.678392 | 0.218298 | 0.740866 |
| 6 | 2.007651 | -1.286805 | -0.367871 |
| 6 | 3.142208 | -1.662335 | -1.072694 |
| 6 | 3.075401 | -1.900739 | -2.439279 |
| 6 | 1.862050 | -1.771852 | -3.108449 |
| 6 | 0.726732 | -1.396369 | -2.411235 |
| 1 | -0.531144 | -0.186513 | 3.323272 |
| 1 | -2.511841 | 0.638426 | 4.580664 |
| 1 | -4.588641 | 1.198409 | 3.360085 |
| 1 | -4.687586 | 0.929174 | 0.907830 |
| 1 | -2.707791 | 0.094166 | -0.330876 |
| 1 | 2.044102 | -1.091603 | 0.692454 |
| 1 | 4.084611 | -1.766776 | -0.553573 |
| 1 | 3.963954 | -2.190182 | -2.981923 |
| 1 | 1.805810 | -1.960820 | -4.170757 |
| 1 | -0.224406 | -1.273373 | -2.910443 |

**Transition State – [3+2] cycloaddition**

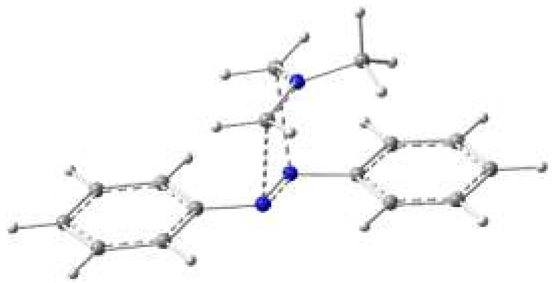

Figure S86. Representative ball and stick model for [3+2] cycloaddition transition state.

**[3+2] cycloaddition transition state, maug-cc-pvDz**

# m062x/maug-cc-pvdz opt=(calcfc,ts,noeigen,recalcfc=25) optcyc=100 freq scrf=(solvent=thf,pcm) nosymm

EE = -745.733956727 (Hartree)
ZPE = -745.443130
$E_{298}$ = -745.427457
$H_{298}$ = -745.426513
$G_{298}$ = -745.486186
S = 125.593 (cal/mol·K)

| | | | |
|---|---|---|---|
| 7 | 0.363331 | 0.154942 | 0.400216 |
| 6 | -1.818035 | 1.828989 | 1.450211 |
| 6 | -2.402106 | 1.276380 | 2.599469 |
| 6 | -3.646514 | 0.660457 | 2.527163 |
| 6 | -4.326342 | 0.592196 | 1.304030 |
| 6 | -3.754835 | 1.159128 | 0.165268 |
| 6 | -2.505138 | 1.781192 | 0.230701 |
| 1 | -1.851342 | 1.328466 | 3.540778 |
| 1 | -4.092366 | 0.230189 | 3.425231 |
| 1 | -5.301574 | 0.106761 | 1.245950 |
| 1 | -4.286246 | 1.118307 | -0.787336 |
| 1 | -2.049590 | 2.230258 | -0.650773 |
| 6 | -0.652563 | -0.882269 | 0.152659 |
| 1 | -1.138116 | -0.685268 | -0.808214 |
| 1 | -0.154540 | -1.859635 | 0.131000 |
| 1 | -1.402112 | -0.857239 | 0.952210 |
| 6 | 1.128123 | 3.646869 | 0.696601 |
| 6 | 1.661116 | 4.256621 | -0.448352 |
| 6 | 1.809895 | 3.760236 | 1.917603 |

| | | | |
|---|---|---|---|
| 6 | 2.855749 | 4.967496 | -0.378634 |
| 1 | 1.118208 | 4.155653 | -1.390392 |
| 6 | 3.008319 | 4.472494 | 1.979935 |
| 1 | 1.383955 | 3.299914 | 2.808544 |
| 6 | 3.536954 | 5.077148 | 0.837891 |
| 1 | 3.258897 | 5.439447 | -1.275948 |
| 1 | 3.532434 | 4.559812 | 2.933308 |
| 1 | 4.473389 | 5.633759 | 0.894627 |
| 6 | 0.784213 | 0.900802 | -0.623942 |
| 1 | 1.692122 | 1.482574 | -0.497836 |
| 1 | 0.390284 | 0.676834 | -1.611001 |
| 6 | 0.694692 | 0.409999 | 1.671960 |
| 1 | 0.290414 | -0.243231 | 2.440684 |
| 1 | 1.581907 | 1.010864 | 1.855975 |
| 7 | -0.525921 | 2.416399 | 1.606295 |
| 7 | -0.102838 | 2.952121 | 0.526759 |

**[3+2] cycloaddition transition state, maug-cc-pvTz**

# m062x/maug-cc-pvtz opt=(calcfc,ts,noeigen,recalcfc=25) optcyc=100 freq scrf=(solvent=thf,pcm) nosymm

EE = -745.913066948 (Hartree)
ZPE = -745.621119
$E_{298}$ = -745.605573
$H_{298}$ = -745.604629
$G_{298}$ = -745.664126
S = 125.222 (cal/mol·K)

| | | | |
|---|---|---|---|
| 7 | 0.357312 | 0.168737 | 0.405250 |
| 6 | -1.817313 | 1.830947 | 1.445423 |
| 6 | -2.393889 | 1.272395 | 2.586953 |
| 6 | -3.628003 | 0.652896 | 2.514721 |
| 6 | -4.305181 | 0.585279 | 1.297866 |
| 6 | -3.740974 | 1.155908 | 0.166411 |
| 6 | -2.500990 | 1.781728 | 0.232929 |
| 1 | -1.846154 | 1.322613 | 3.519352 |
| 1 | -4.066790 | 0.220024 | 3.403572 |
| 1 | -5.269253 | 0.098867 | 1.239621 |
| 1 | -4.268095 | 1.114542 | -0.777747 |
| 1 | -2.052765 | 2.229870 | -0.641179 |
| 6 | -0.659695 | -0.868407 | 0.163244 |

| | | | |
|---|---|---|---|
| 1 | -1.154404 | -0.665863 | -0.780660 |
| 1 | -0.165734 | -1.836471 | 0.128166 |
| 1 | -1.391790 | -0.849984 | 0.965967 |
| 6 | 1.127607 | 3.646024 | 0.697810 |
| 6 | 1.661546 | 4.249198 | -0.441418 |
| 6 | 1.806953 | 3.756317 | 1.911997 |
| 6 | 2.851980 | 4.952577 | -0.372446 |
| 1 | 1.124063 | 4.148882 | -1.375798 |
| 6 | 3.001166 | 4.461605 | 1.974605 |
| 1 | 1.384317 | 3.301358 | 2.795821 |
| 6 | 3.529242 | 5.060831 | 0.838322 |
| 1 | 3.254455 | 5.417779 | -1.262170 |
| 1 | 3.521136 | 4.546842 | 2.919657 |
| 1 | 4.459104 | 5.609848 | 0.894722 |
| 6 | 0.775412 | 0.905480 | -0.619701 |
| 1 | 1.673479 | 1.485633 | -0.502149 |
| 1 | 0.381596 | 0.683072 | -1.596171 |
| 6 | 0.689288 | 0.426577 | 1.670091 |
| 1 | 0.283649 | -0.210396 | 2.437394 |
| 1 | 1.564920 | 1.027030 | 1.852444 |
| 7 | -0.529631 | 2.420813 | 1.600887 |
| 7 | -0.102742 | 2.956119 | 0.528854 |

**[3+2] cycloaddition transition state, maug-cc-pvQz**

# m062x/maug-cc-pvqz opt=(calcfc,ts,noeigen,recalcfc=25) optcyc=100 freq scrf=(solvent=thf,pcm) nosymm

EE = -745.958247084 (Hartree)
ZPE = -745.666189
$E_{298}$ = -745.650687
$H_{298}$ = -745.649743
$G_{298}$ = -745.709103
S = 124.933 (cal/mol·K)

| | | | |
|---|---|---|---|
| 7 | 0.355628 | 0.170538 | 0.405956 |
| 6 | -1.816481 | 1.831782 | 1.445827 |
| 6 | -2.392410 | 1.271898 | 2.586657 |
| 6 | -3.625118 | 0.650971 | 2.513709 |
| 6 | -4.301904 | 0.583326 | 1.297154 |
| 6 | -3.738735 | 1.155411 | 0.166647 |

| | | | |
|---|---|---|---|
| 6 | -2.500099 | 1.782758 | 0.233831 |
| 1 | -1.845421 | 1.321510 | 3.518546 |
| 1 | -4.063022 | 0.216971 | 3.401402 |
| 1 | -5.264394 | 0.095713 | 1.238536 |
| 1 | -4.265303 | 1.113946 | -0.776825 |
| 1 | -2.052073 | 2.231411 | -0.639103 |
| 6 | -0.661616 | -0.864569 | 0.164031 |
| 1 | -1.148593 | -0.668087 | -0.784002 |
| 1 | -0.170951 | -1.833480 | 0.138892 |
| 1 | -1.399344 | -0.839407 | 0.960156 |
| 6 | 1.127821 | 3.644780 | 0.697019 |
| 6 | 1.661864 | 4.247568 | -0.441883 |
| 6 | 1.806633 | 3.755377 | 1.910912 |
| 6 | 2.851638 | 4.951019 | -0.372492 |
| 1 | 1.125505 | 4.147217 | -1.375959 |
| 6 | 3.000203 | 4.460700 | 1.973870 |
| 1 | 1.383925 | 3.300029 | 2.793502 |
| 6 | 3.528148 | 5.059755 | 0.838084 |
| 1 | 3.254072 | 5.415790 | -1.261421 |
| 1 | 3.519567 | 4.546076 | 2.918269 |
| 1 | 4.457120 | 5.608602 | 0.894563 |
| 6 | 0.773270 | 0.907457 | -0.617532 |
| 1 | 1.671002 | 1.486541 | -0.500612 |
| 1 | 0.378798 | 0.687415 | -1.593174 |
| 6 | 0.688552 | 0.426636 | 1.669727 |
| 1 | 0.283650 | -0.210014 | 2.436341 |
| 1 | 1.563369 | 1.026522 | 1.852500 |
| 7 | -0.529931 | 2.421311 | 1.600020 |
| 7 | -0.101548 | 2.955193 | 0.529489 |

**1,2,4-triazolidine product (3a)**

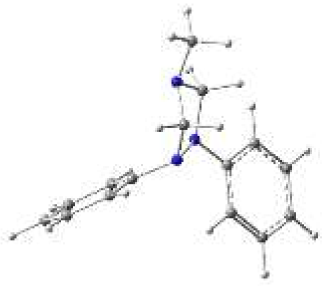

Figure S87. Representative ball and stick model for 1,2,4-triazolidine.

**1,2,4-triazolidine 3a, maug-cc-pvDz**

# m062x/maug-cc-pvdz opt=(calcfc,noeigen) optcyc=50 freq scrf=(solvent=thf,pcm) nosymm

EE = -745.811515493 (Hartree)
ZPE = -745.514914
$E_{298}$ = -745.500115
$H_{298}$ = -745.499171
$G_{298}$ = -745.557734
S = 123.257 (cal/mol·K)

| | | | |
|---|---|---|---|
| 7 | 0.011694 | -0.007578 | 0.009172 |
| 6 | 0.015185 | 0.012205 | 1.468468 |
| 7 | 1.437331 | 0.003248 | 1.789968 |
| 7 | 2.025822 | 0.774186 | 0.752815 |
| 6 | 1.035300 | 0.970374 | -0.314320 |
| 6 | -1.279353 | 0.309670 | -0.576424 |
| 1 | -1.661123 | 1.296523 | -0.243251 |
| 1 | -1.193037 | 0.319341 | -1.671350 |
| 1 | -2.006795 | -0.463798 | -0.295879 |
| 6 | 2.047227 | -1.271722 | 1.975146 |
| 6 | 3.252454 | -1.610238 | 1.348929 |
| 6 | 3.856212 | -2.840427 | 1.615970 |
| 6 | 3.277707 | -3.744175 | 2.505958 |
| 6 | 2.085062 | -3.395678 | 3.144537 |
| 6 | 1.478229 | -2.167830 | 2.893848 |
| 6 | 2.826990 | 1.849753 | 1.152541 |
| 6 | 3.379341 | 1.899283 | 2.443516 |
| 6 | 4.227481 | 2.946342 | 2.797797 |
| 6 | 4.548113 | 3.953596 | 1.885321 |

| | | | |
|---|---|---|---|
| 6 | 4.006670 | 3.896694 | 0.600420 |
| 6 | 3.155339 | 2.857439 | 0.228625 |
| 1 | 3.709148 | -0.908463 | 0.652887 |
| 1 | 4.792842 | -3.090659 | 1.114966 |
| 1 | 3.753585 | -4.704002 | 2.708894 |
| 1 | 1.624259 | -4.081503 | 3.857239 |
| 1 | 0.567373 | -1.896789 | 3.429180 |
| 1 | 3.135255 | 1.116190 | 3.160044 |
| 1 | 4.643804 | 2.971166 | 3.806331 |
| 1 | 5.213743 | 4.768675 | 2.170209 |
| 1 | 4.247928 | 4.670588 | -0.130165 |
| 1 | 2.757975 | 2.824921 | -0.786260 |
| 1 | -0.501033 | -0.859686 | 1.883875 |
| 1 | -0.439777 | 0.943154 | 1.865192 |
| 1 | 1.461760 | 0.781023 | -1.310294 |
| 1 | 0.642536 | 2.011680 | -0.275496 |

**1,2,4-triazolidine 3a, maug-cc-pvTz**

#m062x/maug-cc-pvtz opt=(calcfc,z-matrix,noeigen) optcyc=50 freq scrf=(pcm,solvent=THF) nosymm

EE = -745.989814442 (Hartree)
ZPE = -745.692051
$E_{298}$ = -745.677351
$H_{298}$ = -745.676407
$G_{298}$ = -745.734723
S = 122.738 (cal/mol·K)

| | | | |
|---|---|---|---|
| 7 | 0.000000 | 0.000000 | 0.000000 |
| 6 | 0.000000 | 0.000000 | 1.457137 |
| 7 | 1.418198 | 0.000000 | 1.781867 |
| 7 | 2.002289 | 0.784955 | 0.753853 |
| 6 | 1.016574 | 0.984874 | -0.311249 |
| 6 | -1.290600 | 0.312854 | -0.584056 |
| 1 | -1.673046 | 1.285256 | -0.241976 |
| 1 | -1.204418 | 0.336949 | -1.669097 |
| 1 | -2.008096 | -0.460068 | -0.314403 |
| 6 | 2.042609 | -1.259756 | 1.979299 |
| 6 | 3.263296 | -1.573551 | 1.386100 |
| 6 | 3.877993 | -2.788547 | 1.660364 |
| 6 | 3.295769 | -3.702242 | 2.525958 |

| | | | |
|---|---|---|---|
| 6 | 2.087888 | -3.378997 | 3.132906 |
| 6 | 1.469634 | -2.167062 | 2.874240 |
| 6 | 2.821765 | 1.839356 | 1.148418 |
| 6 | 3.397281 | 1.866332 | 2.422711 |
| 6 | 4.256750 | 2.895280 | 2.773864 |
| 6 | 4.567780 | 3.907195 | 1.874197 |
| 6 | 4.004329 | 3.873528 | 0.605712 |
| 6 | 3.140808 | 2.853030 | 0.237614 |
| 1 | 3.723605 | -0.868080 | 0.710890 |
| 1 | 4.823125 | -3.018075 | 1.186552 |
| 1 | 3.778304 | -4.646706 | 2.734219 |
| 1 | 1.625312 | -4.070776 | 3.824025 |
| 1 | 0.549753 | -1.916974 | 3.384501 |
| 1 | 3.163323 | 1.082397 | 3.127615 |
| 1 | 4.689163 | 2.902269 | 3.765676 |
| 1 | 5.240382 | 4.705036 | 2.155260 |
| 1 | 4.237121 | 4.649220 | -0.111627 |
| 1 | 2.725820 | 2.838893 | -0.760535 |
| 1 | -0.507027 | -0.873185 | 1.854880 |
| 1 | -0.462581 | 0.913988 | 1.857608 |
| 1 | 1.441694 | 0.811669 | -1.300175 |
| 1 | 0.620452 | 2.014441 | -0.263940 |

**1,2,4-triazolidine 3a, maug-cc-pvQz**

# m062x/maug-cc-pvqz opt=(calcfc,noeigen) optcyc=50 freq scrf=(solvent=thf,pcm) nosymm

EE = -746.035454847
ZPE = -745.737668
$E_{298}$ = -745.722993
$H_{298}$ = -745.722049
$G_{298}$ = -745.780269
S = 122.535 (cal/mol·K)

| | | | |
|---|---|---|---|
| 7 | -0.003088 | 0.002693 | 0.001392 |
| 6 | -0.001813 | 0.002336 | 1.457550 |
| 7 | 1.415886 | 0.000023 | 1.779644 |
| 7 | 1.999008 | 0.784920 | 0.753435 |
| 6 | 1.014228 | 0.984830 | -0.311062 |
| 6 | -1.292430 | 0.312941 | -0.583211 |
| 1 | -1.676300 | 1.284805 | -0.243898 |

| | | | |
|---|---|---|---|
| 1 | -1.206796 | 0.335207 | -1.667386 |
| 1 | -2.009269 | -0.459051 | -0.312931 |
| 6 | 2.040944 | -1.257709 | 1.979176 |
| 6 | 3.266369 | -1.565537 | 1.393537 |
| 6 | 3.883852 | -2.778210 | 1.668868 |
| 6 | 3.299706 | -3.695778 | 2.528241 |
| 6 | 2.087009 | -3.378799 | 3.127626 |
| 6 | 1.465715 | -2.169179 | 2.867748 |
| 6 | 2.820607 | 1.836922 | 1.146995 |
| 6 | 3.399643 | 1.859975 | 2.419306 |
| 6 | 4.261235 | 2.886387 | 2.770535 |
| 6 | 4.570831 | 3.899812 | 1.872832 |
| 6 | 4.004029 | 3.870213 | 0.606346 |
| 6 | 3.138442 | 2.852125 | 0.238160 |
| 1 | 3.727646 | -0.856812 | 0.723741 |
| 1 | 4.832246 | -3.002794 | 1.201162 |
| 1 | 3.784163 | -4.638099 | 2.737390 |
| 1 | 1.622935 | -4.073555 | 3.813402 |
| 1 | 0.541685 | -1.924701 | 3.371344 |
| 1 | 3.166748 | 1.074765 | 3.121873 |
| 1 | 4.696209 | 2.890398 | 3.760310 |
| 1 | 5.244721 | 4.695469 | 2.153793 |
| 1 | 4.235564 | 4.646922 | -0.109026 |
| 1 | 2.720341 | 2.841388 | -0.757814 |
| 1 | -0.509792 | -0.869381 | 1.855240 |
| 1 | -0.463141 | 0.915657 | 1.859393 |
| 1 | 1.438201 | 0.810714 | -1.299570 |
| 1 | 0.619911 | 2.014608 | -0.265733 |

**THF solvent**

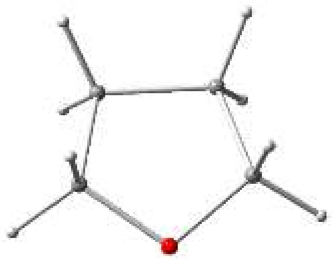

Figure S88. Representative ball and stick model for THF.

**THF, maug-cc-pvQz**

# m062x/maug-cc-pvqz opt=(calcfc,noeigen) optcyc=50 freq scrf=(solvent=thf,pcm) nosymm volume=tight

EE = -232.448282177 (Hartree)
ZPE = -232.330389
$E_{298}$ = -232.325505
$H_{298}$ = -232.324561
$G_{298}$ = -232.358933
S = 72.342 (cal/mol·K)
Volume = 761.603 ($bohr^3$/mol)

| | | | |
|---|---|---|---|
| 8 | -0.001075 | -1.193415 | 0.300354 |
| 6 | 1.126500 | -0.467121 | -0.160379 |
| 6 | 0.773310 | 1.009858 | 0.050639 |
| 6 | -0.772025 | 1.011053 | 0.050608 |
| 6 | -1.127487 | -0.465371 | -0.160450 |
| 1 | 1.996968 | -0.798331 | 0.398857 |
| 1 | 1.287487 | -0.677466 | -1.222943 |
| 1 | 1.158368 | 1.362521 | 1.003810 |
| 1 | 1.192106 | 1.636315 | -0.732218 |
| 1 | -1.156575 | 1.364302 | 1.003770 |
| 1 | -1.189817 | 1.638166 | -0.732255 |
| 1 | -1.998514 | -0.795232 | 0.398715 |
| 1 | -1.288725 | -0.675444 | -1.223032 |

## Calculated Energies for Transition and Ground State Structures in the Triplet State

## Diradical compound

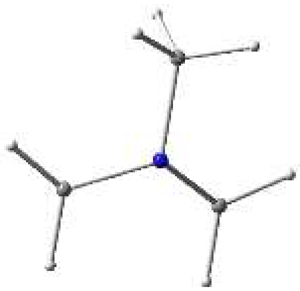

Figure S89. Representative ball and stick model for diradical compound.

## Diradical compound, maug-cc-pvDz

# um062x/maug-cc-pvdz opt=(calcfc,noeigen) optcyc=50 freq scrf=(solvent=thf,pcm) nosymm guess=mix

EE = -173.074330993 (Hartree)
ZPE = -172.981497
$E_{298}$ = -172.975826
$H_{298}$ = -172.974882
$G_{298}$ = -173.009784
S = 73.458
$<S^2>$ = 2.0096

| | | | |
|---|---|---|---|
| 7 | 1.061225 | 1.649539 | -0.602069 |
| 6 | 1.152582 | 2.105888 | 0.723095 |
| 6 | 2.267375 | 1.779909 | -1.402716 |
| 6 | -0.137068 | 2.020818 | -1.234529 |
| 1 | 2.503560 | 2.841621 | -1.608945 |
| 1 | 3.109521 | 1.324100 | -0.866539 |
| 1 | 2.129091 | 1.252832 | -2.355520 |
| 1 | 2.132870 | 2.053665 | 1.195834 |
| 1 | 0.248259 | 2.023914 | 1.325540 |
| 1 | -0.180941 | 1.901927 | -2.316915 |
| 1 | -1.044924 | 1.936520 | -0.637222 |

**Diradical compound, maug-cc-pvTz**

# um062x/maug-cc-pvtz opt=(calcfc,noeigen) optcyc=50 freq scrf=(solvent=thf,pcm) nosymm guess=mix

EE = -173.126622494 (Hartree)
ZPE = -173.033598
$E_{298}$ = -173.027870
$H_{298}$ = -173.026926
$G_{298}$ = -173.061925
S = 73.663
$\langle S^2 \rangle$ = 2.0100

| | | | |
|---|---|---|---|
| 7 | 1.061698 | 1.650859 | -0.602052 |
| 6 | 1.147627 | 2.097033 | 0.722168 |
| 6 | 2.268074 | 1.777988 | -1.399272 |
| 6 | -0.135890 | 2.003186 | -1.235831 |
| 1 | 2.501025 | 2.829535 | -1.604965 |
| 1 | 3.102199 | 1.329167 | -0.864508 |
| 1 | 2.132672 | 1.254707 | -2.343244 |
| 1 | 2.120321 | 2.083780 | 1.187471 |
| 1 | 0.253109 | 2.020680 | 1.319769 |
| 1 | -0.171710 | 1.918474 | -2.310244 |
| 1 | -1.037574 | 1.925322 | -0.649280 |

**Diradical compound, maug-cc-pvQz**

# um062x/maug-cc-pvqz opt=(calcfc,noeigen) optcyc=50 freq scrf=(solvent=thf,pcm) nosymm guess=mix

EE = -173.137844204 (Hartree)
ZPE = -173.044770
$E_{298}$ = -173.039058
$H_{298}$ = -173.038113
$G_{298}$ = -173.073088
S = 73.609
$\langle S^2 \rangle$ = 2.0096

| | | | |
|---|---|---|---|
| 7 | 1.061832 | 1.655949 | -0.601784 |
| 6 | 1.147771 | 2.102428 | 0.720573 |
| 6 | 2.267651 | 1.775519 | -1.398822 |
| 6 | -0.135020 | 2.004621 | -1.235499 |
| 1 | 2.504790 | 2.824217 | -1.608914 |
| 1 | 3.099913 | 1.326372 | -0.863498 |
| 1 | 2.131184 | 1.249660 | -2.340063 |
| 1 | 2.118711 | 2.084480 | 1.187444 |

| | | | |
|---|---|---|---|
| 1 | 0.253084 | 2.028350 | 1.316827 |
| 1 | -0.173028 | 1.910960 | -2.308342 |
| 1 | -1.035337 | 1.928177 | -0.647910 |

**Coordinated ground state (triplet diradical compound coordinated with *trans*-azobenzene)**

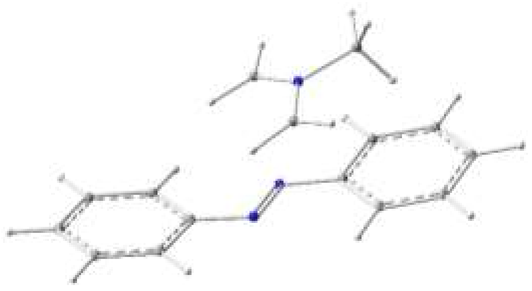

Figure S90. Representative ball and stick model for the coordinated ground state.

**Coordinated ground state, maug-cc-pvDz**

# um062x/maug-cc-pvdz opt=(calcfc,noeigen) optcyc=50 freq scrf=(solvent=thf,pcm) nosymm guess=mix

EE = -745.688496512 (Hartree)
ZPE = -745.400955
$E_{298}$ = -745.383606
$H_{298}$ = -745.382662
$G_{298}$ = -745.448257
S = 138.056
$\langle S^2 \rangle$ = 2.0485

| | | | |
|---|---|---|---|
| 7 | 0.459372 | -2.188601 | -0.608104 |
| 6 | -1.629606 | -0.152362 | 0.517766 |
| 6 | -2.196136 | -0.866939 | 1.602648 |
| 6 | -3.344172 | -1.634073 | 1.441811 |
| 6 | -3.980478 | -1.716160 | 0.195269 |
| 6 | -3.445592 | -0.996675 | -0.881172 |
| 6 | -2.294053 | -0.227263 | -0.735483 |
| 1 | -1.697085 | -0.806748 | 2.572541 |
| 1 | -3.749733 | -2.179281 | 2.296786 |
| 1 | -4.880954 | -2.318179 | 0.068957 |
| 1 | -3.938776 | -1.037913 | -1.855422 |
| 1 | -1.884536 | 0.326184 | -1.579553 |
| 6 | -0.502609 | -3.283748 | -0.352632 |
| 1 | -1.516763 | -2.863533 | -0.391724 |
| 1 | -0.366227 | -4.048506 | -1.124436 |
| 1 | -0.302293 | -3.705081 | 0.635805 |

| | | | |
|---|---|---|---|
| 6 | 1.315993 | 1.656820 | -0.110429 |
| 6 | 1.993486 | 2.166912 | -1.246955 |
| 6 | 1.941204 | 1.813654 | 1.154770 |
| 6 | 3.226268 | 2.795823 | -1.129399 |
| 1 | 1.513159 | 2.057942 | -2.222320 |
| 6 | 3.177689 | 2.447120 | 1.256031 |
| 1 | 1.432996 | 1.438120 | 2.041723 |
| 6 | 3.835187 | 2.943244 | 0.125163 |
| 1 | 3.721172 | 3.180324 | -2.023710 |
| 1 | 3.638472 | 2.560198 | 2.240267 |
| 1 | 4.802214 | 3.438963 | 0.218059 |
| 6 | 0.500126 | -1.660225 | -1.842713 |
| 1 | 1.141383 | -0.799799 | -2.016790 |
| 1 | -0.128843 | -2.101836 | -2.610103 |
| 6 | 1.191385 | -1.704922 | 0.385596 |
| 1 | 1.083299 | -2.145982 | 1.372514 |
| 1 | 1.878099 | -0.884707 | 0.182951 |
| 7 | -0.452505 | 0.528242 | 0.759397 |
| 7 | 0.108951 | 1.024209 | -0.333044 |

**Coordinated ground state, maug-cc-pvTz**

# um062x/maug-cc-pvtz opt=(calcfc,noeigen) optcyc=50 freq scrf=(solvent=thf,pcm) nosymm guess=mix

EE = -745.867979334 (Hartree)
ZPE = -745.579605
$E_{298}$ = -745.562278
$H_{298}$ = -745.561334
$G_{298}$ = -745.627187
S = 138.599
$<S^2>$ = 2.0493

| | | | |
|---|---|---|---|
| 7 | 0.452828 | -2.181890 | -0.575379 |
| 6 | -1.622669 | -0.153446 | 0.504385 |
| 6 | -2.176223 | -0.879888 | 1.579102 |
| 6 | -3.316196 | -1.645770 | 1.418815 |
| 6 | -3.956032 | -1.717531 | 0.181747 |
| 6 | -3.432801 | -0.989424 | -0.885488 |
| 6 | -2.290152 | -0.220555 | -0.739574 |
| 1 | -1.675837 | -0.827820 | 2.538169 |
| 1 | -3.711507 | -2.196352 | 2.263017 |
| 1 | -4.847057 | -2.316436 | 0.055689 |
| 1 | -3.926308 | -1.024962 | -1.849125 |
| 1 | -1.890629 | 0.334736 | -1.574924 |
| 6 | -0.513017 | -3.275776 | -0.332189 |

| | | | |
|---|---|---|---|
| 1 | -1.517605 | -2.866143 | -0.422132 |
| 1 | -0.343484 | -4.050682 | -1.073445 |
| 1 | -0.354804 | -3.667427 | 0.665626 |
| 6 | 1.307325 | 1.672012 | -0.117393 |
| 6 | 1.961729 | 2.217237 | -1.243121 |
| 6 | 1.961134 | 1.773604 | 1.131597 |
| 6 | 3.195921 | 2.826723 | -1.130630 |
| 1 | 1.463209 | 2.148738 | -2.202157 |
| 6 | 3.198879 | 2.388886 | 1.228439 |
| 1 | 1.475309 | 1.372604 | 2.008350 |
| 6 | 3.831678 | 2.919592 | 0.108195 |
| 1 | 3.670705 | 3.237258 | -2.012703 |
| 1 | 3.679267 | 2.459306 | 2.196678 |
| 1 | 4.796534 | 3.399072 | 0.196778 |
| 6 | 0.528641 | -1.680950 | -1.818279 |
| 1 | 1.154028 | -0.820551 | -1.988320 |
| 1 | -0.060268 | -2.144382 | -2.590242 |
| 6 | 1.137120 | -1.671329 | 0.426970 |
| 1 | 0.997244 | -2.082844 | 1.411794 |
| 1 | 1.827454 | -0.865150 | 0.234563 |
| 7 | -0.446886 | 0.520650 | 0.743414 |
| 7 | 0.092563 | 1.064110 | -0.334160 |

**Coordinated ground state, maug-cc-pvQz**

# um062x/maug-cc-pvqz opt=(calcfc,noeigen) optcyc=50 freq scrf=(solvent=thf,pcm) nosymm guess=mix

EE = -745.912790919 (Hartree)
ZPE = -745.624328
$E_{298}$ = -745.607015
$H_{298}$ = -745.606071
$G_{298}$ = -745.671930
S = 138.613
$\langle S^2 \rangle$ = 2.0482

| | | | |
|---|---|---|---|
| 7 | 0.451709 | -2.177702 | -0.564650 |
| 6 | -1.622713 | -0.154090 | 0.498716 |
| 6 | -2.173563 | -0.883663 | 1.572130 |
| 6 | -3.313401 | -1.648858 | 1.411871 |
| 6 | -3.955005 | -1.717453 | 0.176194 |
| 6 | -3.433804 | -0.987136 | -0.889920 |
| 6 | -2.291846 | -0.218239 | -0.743927 |
| 1 | -1.671515 | -0.834882 | 2.529625 |
| 1 | -3.706757 | -2.201520 | 2.254534 |
| 1 | -4.845183 | -2.316107 | 0.050117 |

| | | | |
|---|---|---|---|
| 1 | -3.928008 | -1.021346 | -1.852285 |
| 1 | -1.893034 | 0.338029 | -1.577985 |
| 6 | -0.508254 | -3.274797 | -0.322455 |
| 1 | -1.514107 | -2.873900 | -0.425561 |
| 1 | -0.326697 | -4.054218 | -1.054672 |
| 1 | -0.358400 | -3.657184 | 0.679131 |
| 6 | 1.304791 | 1.674801 | -0.120588 |
| 6 | 1.955461 | 2.227972 | -1.244087 |
| 6 | 1.964192 | 1.763332 | 1.125947 |
| 6 | 3.191704 | 2.831982 | -1.131641 |
| 1 | 1.453320 | 2.169161 | -2.200957 |
| 6 | 3.203843 | 2.373568 | 1.222711 |
| 1 | 1.481090 | 1.356132 | 2.000419 |
| 6 | 3.833056 | 2.911709 | 0.104686 |
| 1 | 3.663805 | 3.248137 | -2.011487 |
| 1 | 3.688432 | 2.433970 | 2.188595 |
| 1 | 4.799250 | 3.386677 | 0.192952 |
| 6 | 0.525084 | -1.676488 | -1.807287 |
| 1 | 1.144334 | -0.812630 | -1.976702 |
| 1 | -0.060056 | -2.142848 | -2.578980 |
| 6 | 1.133553 | -1.664648 | 0.435966 |
| 1 | 0.996510 | -2.075925 | 1.420291 |
| 1 | 1.820911 | -0.857182 | 0.243170 |
| 7 | -0.446281 | 0.517378 | 0.735873 |
| 7 | 0.087675 | 1.073189 | -0.335677 |

**Transition state – single bond formation**

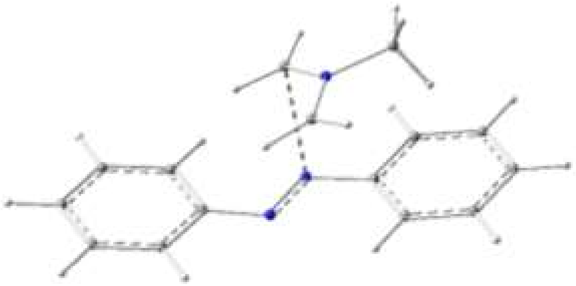

Figure S91. Representative ball and stick model for the single bond formation transition state.

**Single bond formation transition state, maug-cc-pvDz**

# um062x/maug-cc-pvdz opt=(calcfc,ts,noeigen,recalcfc=25) optcyc=100 freq scrf=(solvent=thf,pcm) nosymm guess=mix

EE = -745.688364950 (Hartree)
ZPE = -745.401363
$E_{298}$ = -745.384687
$H_{298}$ = -745.383743
$G_{298}$ = -745.447454
S = 134.092
$\langle S^2 \rangle$ = 2.0410

| | | | |
|---|---|---|---|
| 7 | 0.237381 | -0.035468 | 0.371964 |
| 6 | -1.814577 | 2.004721 | 1.388454 |
| 6 | -2.370860 | 1.273677 | 2.465693 |
| 6 | -3.534524 | 0.529643 | 2.307467 |
| 6 | -4.189010 | 0.484993 | 1.069255 |
| 6 | -3.660836 | 1.220197 | 0.000615 |
| 6 | -2.497084 | 1.971309 | 0.146221 |
| 1 | -1.855424 | 1.304124 | 3.428578 |
| 1 | -3.936191 | -0.027454 | 3.156465 |
| 1 | -5.099172 | -0.102338 | 0.942814 |
| 1 | -4.165371 | 1.202000 | -0.968301 |
| 1 | -2.084849 | 2.529466 | -0.693433 |
| 6 | -0.727295 | -1.120538 | 0.643354 |
| 1 | -1.739810 | -0.743066 | 0.445216 |
| 1 | -0.490286 | -1.963083 | -0.015758 |
| 1 | -0.635931 | -1.421178 | 1.690521 |
| 6 | 1.103684 | 3.876478 | 0.787099 |
| 6 | 1.708710 | 4.528300 | -0.317138 |
| 6 | 1.819043 | 3.855170 | 2.013878 |
| 6 | 2.961646 | 5.115994 | -0.207770 |
| 1 | 1.157666 | 4.556726 | -1.259696 |

| | | | |
|---|---|---|---|
| 6 | 3.075002 | 4.450493 | 2.107802 |
| 1 | 1.363478 | 3.378179 | 2.880998 |
| 6 | 3.662109 | 5.082786 | 1.007033 |
| 1 | 3.401482 | 5.610197 | -1.076518 |
| 1 | 3.605475 | 4.426431 | 3.062583 |
| 1 | 4.645028 | 5.547404 | 1.093395 |
| 6 | 0.374261 | 0.375940 | -0.916115 |
| 1 | 0.994210 | 1.246458 | -1.115096 |
| 1 | -0.162742 | -0.169747 | -1.685516 |
| 6 | 0.863759 | 0.566716 | 1.362812 |
| 1 | 0.696565 | 0.214646 | 2.377254 |
| 1 | 1.590689 | 1.344116 | 1.134078 |
| 7 | -0.605443 | 2.636752 | 1.617979 |
| 7 | -0.136957 | 3.308623 | 0.576449 |

**Single bond formation transition state, maug-cc-pvTz**

# um062x/maug-cc-pvtz opt=(calcfc,ts,noeigen,recalcfc=25) optcyc=100 freq scrf=(solvent=thf,pcm) nosymm guess=mix

EE = -745.867945540 (Hartree)
ZPE = -745.579736
$E_{298}$ = -745.563250
$H_{298}$ = -745.562306
$G_{298}$ = -745.625479
S = 132.958
$\langle S^2 \rangle$ = 2.0435

| | | | |
|---|---|---|---|
| 7 | 0.233519 | -0.029034 | 0.379796 |
| 6 | -1.811403 | 2.009248 | 1.385445 |
| 6 | -2.363271 | 1.277774 | 2.456187 |
| 6 | -3.516420 | 0.531495 | 2.297824 |
| 6 | -4.165900 | 0.482952 | 1.065136 |
| 6 | -3.641534 | 1.215058 | 0.001623 |
| 6 | -2.487769 | 1.967575 | 0.147454 |
| 1 | -1.853643 | 1.311154 | 3.411354 |
| 1 | -3.913181 | -0.022568 | 3.138980 |
| 1 | -5.065550 | -0.103002 | 0.939215 |
| 1 | -4.140311 | 1.193036 | -0.959558 |
| 1 | -2.081613 | 2.521098 | -0.685921 |
| 6 | -0.736893 | -1.110547 | 0.646156 |
| 1 | -1.737666 | -0.728654 | 0.451506 |
| 1 | -0.507581 | -1.943117 | -0.012538 |
| 1 | -0.649134 | -1.413599 | 1.682957 |
| 6 | 1.103932 | 3.872396 | 0.784604 |
| 6 | 1.704263 | 4.527123 | -0.312208 |

| | | | |
|---|---|---|---|
| 6 | 1.821163 | 3.843776 | 2.002359 |
| 6 | 2.949396 | 5.113507 | -0.203063 |
| 1 | 1.156009 | 4.559831 | -1.245296 |
| 6 | 3.069399 | 4.437996 | 2.096580 |
| 1 | 1.374508 | 3.363077 | 2.860088 |
| 6 | 3.649153 | 5.075379 | 1.003982 |
| 1 | 3.383023 | 5.609096 | -1.062260 |
| 1 | 3.598719 | 4.408227 | 3.041012 |
| 1 | 4.622550 | 5.537743 | 1.089941 |
| 6 | 0.369487 | 0.384013 | -0.901656 |
| 1 | 0.987609 | 1.243340 | -1.099811 |
| 1 | -0.164302 | -0.151668 | -1.666177 |
| 6 | 0.855629 | 0.564737 | 1.370159 |
| 1 | 0.681257 | 0.218971 | 2.374765 |
| 1 | 1.575370 | 1.337844 | 1.151817 |
| 7 | -0.613076 | 2.648826 | 1.616908 |
| 7 | -0.131913 | 3.305584 | 0.575276 |

**Single bond formation transition state, maug-cc-pvQz**

# um062x/maug-cc-pvqz opt=(calcfc,ts,noeigen,recalcfc=25) optcyc=100 freq scrf=(solvent=thf,pcm) nosymm guess=mix

EE = -745.912775274 (Hartree)
ZPE = -745.624525
$E_{298}$ = -745.608063
$H_{298}$ = -745.607118
$G_{298}$ = -745.670195
S = 132.755
$\langle S^2 \rangle$ = 2.0446

| | | | |
|---|---|---|---|
| 7 | 0.232352 | -0.024593 | 0.370716 |
| 6 | -1.812807 | 2.013233 | 1.387896 |
| 6 | -2.364717 | 1.282628 | 2.459241 |
| 6 | -3.514510 | 0.532244 | 2.300525 |
| 6 | -4.161821 | 0.478465 | 1.067359 |
| 6 | -3.637852 | 1.209159 | 0.003144 |
| 6 | -2.487278 | 1.965465 | 0.148840 |
| 1 | -1.856989 | 1.319221 | 3.414379 |
| 1 | -3.910273 | -0.021042 | 3.141598 |
| 1 | -5.058642 | -0.110276 | 0.941515 |
| 1 | -4.135031 | 1.183591 | -0.957866 |
| 1 | -2.082952 | 2.519174 | -0.684290 |
| 6 | -0.733556 | -1.108092 | 0.640660 |
| 1 | -1.736190 | -0.722298 | 0.470071 |
| 1 | -0.518602 | -1.931185 | -0.032635 |

| | | | |
|---|---|---|---|
| 1 | -0.626811 | -1.425321 | 1.670272 |
| 6 | 1.107111 | 3.864074 | 0.784197 |
| 6 | 1.720446 | 4.497267 | -0.317567 |
| 6 | 1.808935 | 3.860720 | 2.010678 |
| 6 | 2.962157 | 5.089036 | -0.204000 |
| 1 | 1.185380 | 4.509508 | -1.257905 |
| 6 | 3.053944 | 4.459822 | 2.109015 |
| 1 | 1.353504 | 3.393942 | 2.870385 |
| 6 | 3.645893 | 5.077277 | 1.012105 |
| 1 | 3.405497 | 5.567856 | -1.066716 |
| 1 | 3.571159 | 4.449426 | 3.059624 |
| 1 | 4.616267 | 5.543460 | 1.101306 |
| 6 | 0.341347 | 0.408854 | -0.902525 |
| 1 | 0.955134 | 1.270597 | -1.098938 |
| 1 | -0.211042 | -0.110347 | -1.664065 |
| 6 | 0.879211 | 0.548029 | 1.357130 |
| 1 | 0.721435 | 0.188912 | 2.358783 |
| 1 | 1.587524 | 1.330248 | 1.137855 |
| 7 | -0.621040 | 2.660728 | 1.620482 |
| 7 | -0.123361 | 3.288886 | 0.571364 |

**Stepwise addition intermediate**

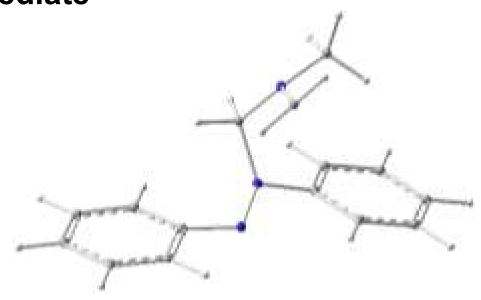

Figure S92. Representative ball and stick model for azomethine ylide.

**Stepwise addition intermediate, maug-cc-pvDz**

# um062x/maug-cc-pvdz opt=(calcfc,noeigen,recalcfc=45) optcyc=90 freq scrf=(solvent=thf,pcm) nosymm guess=mix

EE = -745.725279034 (Hartree)
ZPE = -745.433943
$E_{298}$ = -745.417721
$H_{298}$ = -745.416777
$G_{298}$ = -745.479107
S = 131.183
$\langle S^2 \rangle$ = 2.0156

| | | | |
|---|---|---|---|
| 7 | 0.608575 | -2.217827 | -0.463791 |
| 6 | -1.561061 | -0.450661 | 0.340181 |
| 6 | -2.094382 | -1.083271 | 1.466633 |
| 6 | -3.416225 | -1.526826 | 1.450437 |
| 6 | -4.209061 | -1.331576 | 0.318368 |
| 6 | -3.673945 | -0.682895 | -0.797187 |
| 6 | -2.351552 | -0.242260 | -0.793117 |
| 1 | -1.477174 | -1.221577 | 2.355867 |
| 1 | -3.829410 | -2.019044 | 2.331733 |
| 1 | -5.242875 | -1.678656 | 0.307057 |
| 1 | -4.288666 | -0.526764 | -1.684799 |
| 1 | -1.913490 | 0.252878 | -1.658927 |
| 6 | -0.286207 | -3.329330 | -0.181609 |
| 1 | -1.329295 | -3.110888 | -0.471881 |
| 1 | 0.058161 | -4.211013 | -0.741407 |
| 1 | -0.254709 | -3.563415 | 0.890372 |
| 6 | 1.337516 | 1.619163 | -0.147409 |
| 6 | 1.863187 | 2.394308 | -1.201670 |
| 6 | 2.047104 | 1.567746 | 1.072851 |
| 6 | 3.074933 | 3.059853 | -1.054209 |
| 1 | 1.300690 | 2.450222 | -2.134928 |
| 6 | 3.253447 | 2.248309 | 1.211722 |

| | | | |
|---|---|---|---|
| 1 | 1.625980 | 1.019495 | 1.917264 |
| 6 | 3.781560 | 2.989170 | 0.150917 |
| 1 | 3.471553 | 3.644152 | -1.886039 |
| 1 | 3.783684 | 2.206467 | 2.164630 |
| 1 | 4.728872 | 3.516786 | 0.266264 |
| 6 | 0.788654 | -1.871135 | -1.788565 |
| 1 | 1.396504 | -0.989592 | -1.994023 |
| 1 | 0.716244 | -2.673674 | -2.521928 |
| 6 | 0.783830 | -1.174465 | 0.509447 |
| 1 | 0.695414 | -1.599888 | 1.516451 |
| 1 | 1.782342 | -0.740983 | 0.388598 |
| 7 | -0.189559 | -0.050021 | 0.366041 |
| 7 | 0.091389 | 1.041982 | -0.357090 |

**Stepwise addition intermediate, maug-cc-pvTz**

# um062x/maug-cc-pvtz opt=(calcfc,noeigen,recalcfc=45) optcyc=90 freq scrf=(solvent=thf,pcm) nosymm guess=mix

EE = -745.903935598 (Hartree)
ZPE = -745.611674
$E_{298}$ = -745.595472
$H_{298}$ = -745.594528
$G_{298}$ = -745.657035
S = 131.558
$\langle S^2 \rangle$ = 2.0167

| | | | |
|---|---|---|---|
| 7 | 0.603087 | -2.225466 | -0.444560 |
| 6 | -1.556301 | -0.449339 | 0.323245 |
| 6 | -2.083280 | -1.106307 | 1.430066 |
| 6 | -3.400839 | -1.539044 | 1.413137 |
| 6 | -4.196819 | -1.312348 | 0.297985 |
| 6 | -3.667953 | -0.643411 | -0.799398 |
| 6 | -2.350144 | -0.211523 | -0.793110 |
| 1 | -1.466730 | -1.271262 | 2.303657 |
| 1 | -3.806776 | -2.047303 | 2.276949 |
| 1 | -5.223750 | -1.649961 | 0.285671 |
| 1 | -4.282237 | -0.464461 | -1.671361 |
| 1 | -1.920644 | 0.297641 | -1.643122 |
| 6 | -0.268420 | -3.346001 | -0.136751 |
| 1 | -1.306800 | -3.150257 | -0.422432 |
| 1 | 0.082924 | -4.223888 | -0.679073 |
| 1 | -0.229263 | -3.557286 | 0.929781 |
| 6 | 1.340742 | 1.618233 | -0.150999 |
| 6 | 1.855450 | 2.406576 | -1.192469 |
| 6 | 2.055301 | 1.555232 | 1.058129 |

| | | | |
|---|---|---|---|
| 6 | 3.057839 | 3.072761 | -1.043979 |
| 1 | 1.292211 | 2.472172 | -2.113661 |
| 6 | 3.251790 | 2.237778 | 1.198964 |
| 1 | 1.646335 | 0.999026 | 1.891022 |
| 6 | 3.767723 | 2.991132 | 0.150511 |
| 1 | 3.443724 | 3.664991 | -1.862831 |
| 1 | 3.782932 | 2.187420 | 2.140159 |
| 1 | 4.704255 | 3.518117 | 0.266719 |
| 6 | 0.766862 | -1.897872 | -1.770286 |
| 1 | 1.339386 | -1.011993 | -2.000219 |
| 1 | 0.668944 | -2.692629 | -2.493148 |
| 6 | 0.784860 | -1.171709 | 0.511953 |
| 1 | 0.701141 | -1.578902 | 1.516144 |
| 1 | 1.773173 | -0.742844 | 0.380335 |
| 7 | -0.188188 | -0.052019 | 0.351009 |
| 7 | 0.101494 | 1.039515 | -0.361783 |

**Stepwise addition intermediate, maug-cc-pvQz**

# um062x/maug-cc-pvqz opt=(calcfc,noeigen,recalcfc=45) optcyc=90 freq scrf=(solvent=thf,pcm) nosymm guess=mix

EE = -745.949348538 (Hartree)
ZPE = -745.657029
$E_{298}$ = -745.640849
$H_{298}$ = -745.639905
$G_{298}$ = -745.702422
S = 131.578
$\langle S^2 \rangle$ = 2.0167

| | | | |
|---|---|---|---|
| 7 | 0.603251 | -2.223785 | -0.442550 |
| 6 | -1.556344 | -0.449263 | 0.323618 |
| 6 | -2.083355 | -1.110258 | 1.427519 |
| 6 | -3.400631 | -1.542231 | 1.409032 |
| 6 | -4.196498 | -1.310821 | 0.295446 |
| 6 | -3.667751 | -0.638051 | -0.798987 |
| 6 | -2.350323 | -0.206758 | -0.791147 |
| 1 | -1.467226 | -1.279728 | 2.299473 |
| 1 | -3.806222 | -2.053476 | 2.270157 |
| 1 | -5.222730 | -1.647793 | 0.282018 |
| 1 | -4.281641 | -0.455575 | -1.669431 |
| 1 | -1.920906 | 0.305328 | -1.638338 |
| 6 | -0.264839 | -3.345289 | -0.134885 |
| 1 | -1.304088 | -3.150477 | -0.414833 |
| 1 | 0.084281 | -4.220532 | -0.680846 |
| 1 | -0.221149 | -3.560478 | 0.929705 |

| | | | |
|---|---|---|---|
| 6 | 1.340364 | 1.616208 | -0.151166 |
| 6 | 1.854559 | 2.403048 | -1.193552 |
| 6 | 2.055213 | 1.555016 | 1.057371 |
| 6 | 3.056262 | 3.069603 | -1.046150 |
| 1 | 1.291867 | 2.467206 | -2.114182 |
| 6 | 3.251173 | 2.237891 | 1.196938 |
| 1 | 1.647244 | 0.999788 | 1.890349 |
| 6 | 3.766259 | 2.989846 | 0.147841 |
| 1 | 3.441534 | 3.660343 | -1.865248 |
| 1 | 3.782209 | 2.188881 | 2.137289 |
| 1 | 4.701946 | 3.516840 | 0.262944 |
| 6 | 0.765775 | -1.895057 | -1.766313 |
| 1 | 1.338891 | -1.010239 | -1.995487 |
| 1 | 0.669444 | -2.688511 | -2.489674 |
| 6 | 0.783046 | -1.170744 | 0.514524 |
| 1 | 0.698342 | -1.578495 | 1.517558 |
| 1 | 1.771023 | -0.742349 | 0.385356 |
| 7 | -0.188919 | -0.052579 | 0.351691 |
| 7 | 0.101964 | 1.037259 | -0.359786 |

References

(1) Love, B. E.; Jones, E. G. The Use of Salicylaldehyde Phenylhydrazone as an Indicator for the Titration of Organometallic Reagents. *J. Org. Chem.* **1999**, *64* (10), 3755-3756. DOI: 10.1021/jo982433e.
(2) Durka, J.; Zielinska, B.; Gryko, D. Aliphatic Amines Unlocked for Selective Transformations through Diazotization. *Angew. Chem. Int. Ed.* **2025**, *64* (7), e202419450. DOI: 10.1002/anie.202419450.
(3) Li, M. M., Jun-An; Liao, Saihu. Atom-Transfer Radical Polymerization of a SuFExable Vinyl Monomer and Polymer Library Construction via SuFEx Click Reaction. *Macromolecules* **2023**, *56* (3), 806-814. DOI: 10.1021/acs.macromol.2c01492.
(4) Liu, J.; Song, Y.; Wu, X.; Ma, L. *N*-Dimethylation and *N*-Functionalization of Amines Using Ru Nanoparticle Catalysts and Formaldehyde or Functional Aldehydes as the Carbon Source. *ACS Omega* **2021**, *6* (35), 22504-22513. DOI: 10.1021/acsomega.1c01961.
(5) Meiners, A. F.; Bolze, C.; Scherer, A. L.; Morriss, F. V. An Application of Statistical Design to Organic Synthesis. The Reductive Alkylation of t-Butylamine (Leuckart Reaction). *J. Org. Chem.* **1958**, *23* (8), 1122-1125. DOI: 10.1021/jo01102a010.
(6) Chakrabarti, K.; Dutta, K.; Kundu, S. Synthesis of *N*-methylated amines from acyl azides using methanol. *Organic & Biomolecular Chemistry* **2020**, *18* (30), 5891-5896. DOI: 10.1039/d0ob01303j.
(7) Nibbs, A. E.; Montgomery, T. D.; Zhu, Y.; Rawal, V. H. Access to Spirocyclized Oxindoles and Indolenines via Palladium-Catalyzed Cascade Reactions of Propargyl Carbonates with 2-Oxotryptamines and Tryptamines. *J. Org. Chem.* **2015**, *80* (10), 4928-4941. DOI: 10.1021/acs.joc.5b00277.
(8) Tavakolian, M.; Saki, S.; Hosseini-Sarvari, M. Selective photocatalytic reduction of nitrobenzene to anilines, azoxybenzene, and azobenzene: a solvent-dependent and light-induced process mediated by a CdS/$NH_2$-MIL-125 nanocomposite. *Org. Biomol. Chem.* **2025**, *23* (27), 6625-6636. DOI: 10.1039/d5ob00705d.
(9) Ma, Y.; Zhang, R.; Ma, R.; Qiu, H.; Xie, J. Nitro Compounds/Alcohols as Oxidant/Reductant Pairs: A Practical Synthesis of Azo Compounds and Ketones. *Synthesis* **2023**, *56* (05), 851-859. DOI: 10.1055/a-2226-4152.
(10) Damiano, C.; Cavalleri, M.; Panza, N.; Gallo, E. Cobalt Porphyrin‐Catalysed Synthesis of Azobenzenes by Dehydrogenative Coupling of Anilines. *Eur. J. Org. Chem.* **2022**, *2022* (34). DOI: 10.1002/ejoc.202200791.
(11) Wang, Z. Q.; Yu, J. X.; Bai, S. Q.; Liu, B.; Wang, C. Y.; Li, J. H. Oxidative Dehydrogenation of Hydrazobenzenes toward Azo Compounds Catalyzed by *tert*-Butyl Nitrite in EtOH. *ACS Omega* **2020**, *5* (44), 28856-28862. DOI: 10.1021/acsomega.0c04348.
(12) Sitter, J. D.; Vannucci, A. K. Photocatalytic Oxidative Coupling of Arylamines for the Synthesis of Azoaromatics and the Role of $O_2$ in the Mechanism. *J. Am. Chem. Soc.* **2021**, *143* (7), 2938-2943. DOI: 10.1021/jacs.0c13101.
(13) Vesamaki, S.; Meteling, H.; Nasare, R.; Siiskonen, A.; Patrakka, J.; Roas-Escalona, N.; Linder, M.; Virkki, M.; Priimagi, A. Strategies to control humidity sensitivity of azobenzene isomerisation kinetics in polymer thin films. *Commun. Mater.* **2024**, *5* (1), 209. DOI: 10.1038/s43246-024-00642-w.

(14) Rowshanpour, R.; Vemulapalli, S.; Dudding, T. A Photo-Dual Catalytic Strategy for Azo Compound Synthesis. *Adv. Synth. Catal.* **2025**, *367* (11). DOI: 10.1002/adsc.202500008.
(15) Ma, Y.; Wu, S.; Jiang, S.; Xiao, F.; Deng, G. J. Electrosynthesis of Azobenzenes Directly from Nitrobenzenes. *Chin. J. Chem.* **2021**, *39* (12), 3334-3338. DOI: 10.1002/cjoc.202100470.
(16) Lv, H.; Laishram, R. D.; Yang, Y.; Li, J.; Xu, D.; Zhan, Y.; Luo, Y.; Su, Z.; More, S.; Fan, B. TEMPO catalyzed oxidative dehydrogenation of hydrazobenzenes to azobenzenes. *Org. Biomol. Chem.* **2020**, *18* (18), 3471-3474. DOI: 10.1039/d0ob00103a.
(17) Wang, Z.; Yin, Z.; Zhu, F.; Li, Y.; Wu, X. F. Palladium-Catalyzed Carbonylative Cyclization of Azoarenes. *ChemCatChem* **2017**, *9* (19), 3637-3640. DOI: 10.1002/cctc.201700679.
(18) Durie, K.; Yatvin, J.; Kovaliov, M.; Crane, G. H.; Horn, J.; Averick, S.; Locklin, J. SuFEx Postpolymerization Modification Kinetics and Reactivity in Polymer Brushes. *Macromolecules* **2018**, *51* (2), 297-305. DOI: 10.1021/acs.macromol.7b02372.
(19) Chen, D.; Burford, W. B.; Pham, G.; Zhang, L.; Alto, L. T.; Ertelt, J. M.; Winter, M. G.; Winter, S. E.; Way, S. S.; Alto, N. M. Systematic reconstruction of an effector-gene network reveals determinants of Salmonella cellular and tissue tropism. *Cell Host Microbe* **2021**, *29* (10), 1531-1544 e1539. DOI: 10.1016/j.chom.2021.08.012.
(20) de Jong, M. F.; Liu, Z.; Chen, D.; Alto, N. M. Shigella flexneri suppresses NF-kappaB activation by inhibiting linear ubiquitin chain ligation. *Nat. Microbiol.* **2016**, *1* (7), 16084. DOI: 10.1038/nmicrobiol.2016.84.
(21) Jones, S.; Portnoy, D. A. Characterization of Listeria monocytogenes pathogenesis in a strain expressing perfringolysin O in place of listeriolysin O. *Infect. Immun.* **1994**, *62* (12), 5608-5613. DOI: 10.1128/iai.62.12.5608-5613.1994.
(22) Hudzicki, J. Kirby-Bauer Disk Diffusion Susceptibility Test Protocol. In ASM Conference for Undergraduate Educators, **2009**.
(23) Verma, P.; Truhlar, D. G. Status and Challenges of Density Functional Theory. *Trends in Chemistry* **2020**, *2* (4), 302-318. DOI: 10.1016/j.trechm.2020.02.005.
(24) Frisch, M. J. T., G. W.; Schlegel, H. B.; Scuseria, G. E.; Robb, M. A.; Cheeseman, J. R.; Scalmani, G.; Barone, V.; Petersson, G. A.; Nakatsuji, H.; Li, X.; Caricato, M.; Marenich, A. V.; Bloino, J.; Janesko, B. G.; Gomperts, R.; Mennucci, B.; Hratchian, H. P.; Ortiz, J. V.; Izmaylov, A. F.; Sonnenberg, J. L.; Williams-Young, D.; Ding, F.; Lipparini, F.; Egidi, F.; Goings, J.; Peng, B.; Petrone, A.; Henderson, T.; Ranasinghe, D.; Zakrzewski, V. G.; Gao, J.; Rega, N.; Zheng, G.; Liang, W.; Hada, M.; Ehara, M.; Toyota, K.; Fukuda, R.; Hasegawa, J.; Ishida, M.; Nakajima, T.; Honda, Y.; Kitao, O.; Nakai, H.; Vreven, T.; Throssell, K.; Montgomery, J. A. J.; Peralta, J. E.; Ogliaro, F.; Bearpark, M. J.; Heyd, J. J.; Brothers, E. N.; Kudin, K. N.; Staroverov, V. N.; Keith, T. A.; Kobayashi, R.; Normand, J.; Raghavachari, K.; Rendell, A. P.; Burant, J. C.; Iyengar, S. S.; Tomasi, J.; Cossi, M.; Millam, J. M.; Klene, M.; Adamo, C.; Cammi, R.; Ochterski, J. W.; Martin, R. L.; Morokuma, K.; Farkas, O.; Foresman, J. B.; Fox, D. J., Gaussian 16, Revision C.01; Gaussian, Inc.: Wallingford CT, **2016**.
(25) Zhao, Y.; Truhlar, D. G. The M06 suite of density functionals for main group thermochemistry, thermochemical kinetics, noncovalent interactions, excited states, and transition elements: two new functionals and systematic testing of four M06-class

functionals and 12 other functionals. *Theor. Chem. Acc.* **2007**, *120* (1-3), 215-241. DOI: 10.1007/s00214-007-0310-x.
(26) Dunning, T. H., Jr. Gaussian basis sets for use in correlated molecular calculations. I. The atoms boron through neon and hydrogen. *J. Chem. Phys.* **1989**, *90* (2), 1007-1023. DOI: 10.1063/1.456153 (acccessed 8/17/2025).
(27) Jensen, J. H. *Molecular Modeling Basics*; CRC Press, **2010**. DOI: 10.1201/9781420075274
(28) Mammen, M.; Shakhnovich, E. I.; Deutch, J. M.; Whitesides, G. M. Estimating the Entropic Cost of Self-Assembly of Multiparticle Hydrogen-Bonded Aggregates Based on the Cyanuric Acid·Melamine Lattice. *J. Org. Chem.* **1998**, *63* (12), 3821-3830. DOI: 10.1021/jo970944f.
